# Supplementary material for: Effect of Dihydroquercetin During Long-Last Growth of Yarrowia lipolytica Yeast: Anti-Aging Potential and Hormetic Properties
Source: Int J Mol Sci. 2024 Nov 22;25(23):12574. doi: 10.3390/ijms252312574 (PMC11641753; doi:10.3390/ijms252312574)
Supplement: Supplementary file 1 [file ijms-3312670-supplementary.pdf › ijms-3312670-supplementary.pdf]

*ANOVAOneWay (20.10.2024 14:08:26)*

## Input Data

| | Data | Range |
|---|---|---|
| Control, 1 day | [Book1]"Amount of cells"!A"Control, 1 day" | [1*:5*] |
| Control, 7 days | [Book1]"Amount of cells"!B"Control, 7 days" | [1*:10*] |
| Control, 14 days | [Book1]"Amount of cells"!C"Control, 14 days" | [1*:9*] |
| Control, 28 days | [Book1]"Amount of cells"!D"Control, 28 days" | [1*:6*] |
| Control, 42 days | [Book1]"Amount of cells"!E"Control, 42 days" | [1*:3*] |
| Control, 56 days | [Book1]"Amount of cells"!F"Control, 56 days" | [1*:3*] |
| DHQ, 1 day | [Book1]"Amount of cells"!G"DHQ, 1 day" | [1*:4*] |
| DHQ, 7 days | [Book1]"Amount of cells"!H"DHQ, 7 days" | [1*:10*] |
| DHQ, 14 days | [Book1]"Amount of cells"!I"DHQ, 14 days" | [1*:9*] |
| DHQ, 28 days | [Book1]"Amount of cells"!J"DHQ, 28 days" | [1*:6*] |
| DHQ, 42 days | [Book1]"Amount of cells"!K"DHQ, 42 days" | [1*:3*] |
| DHQ, 56 days | [Book1]"Amount of cells"!L"DHQ, 56 days" | [1*:3*] |

There exist missing values in the input data.

## Descriptive Statistics

| | N Analysis | N Missing | Mean | Standard Deviation | SE of Mean |
|---|---|---|---|---|---|
| Control, 1 day | 5 | 0 | 4,97105E11 | 2,0624E11 | 9,22333E10 |
| Control, 7 days | 10 | 0 | 1,01482E11 | 7,90298E10 | 2,49914E10 |
| Control, 14 days | 9 | 0 | 6,36087E10 | 9,56E9 | 3,18667E9 |
| Control, 28 days | 6 | 0 | 6,43143E10 | 2,42412E10 | 9,89642E9 |
| Control, 42 days | 3 | 0 | 6,31298E10 | 8,36275E9 | 4,82823E9 |
| Control, 56 days | 3 | 0 | 5,51388E10 | 1,09164E10 | 6,3026E9 |
| DHQ, 1 day | 4 | 0 | 4,31678E11 | 9,38902E10 | 4,69451E10 |
| DHQ, 7 days | 3 | 7 | 7,35776E10 | 1,0906E10 | 6,29659E9 |
| DHQ, 14 days | 3 | 6 | 6,47271E10 | 1,21678E10 | 7,02508E9 |
| DHQ, 28 days | 4 | 2 | 5,05429E10 | 6,47457E9 | 3,23729E9 |
| DHQ, 42 days | 3 | 0 | 4,14021E10 | 1,37857E8 | 7,95919E7 |
| DHQ, 56 days | 3 | 0 | 5,20625E10 | 3,7625E9 | 2,17228E9 |

## ANOVA

### Overall ANOVA

| | DF | Sum of Squares | Mean Square | F Value | Prob>F |
|---|---|---|---|---|---|
| Model | 11 | 1,2307E24 | 1,11882E23 | 19,11524 | 2,728E-13 |
| Error | 44 | 2,57533E23 | 5,85301E21 | | |
| Total | 55 | 1,48823E24 | | | |

Null Hypothesis: The means of all levels are equal.
Alternative Hypothesis: The means of one or more levels are different.
At the 0.05 level, the population means are significantly different.

### Fit Statistics

| | R-Square | Coeff Var | Root MSE | Data Mean |
|---|---|---|---|---|
| | 0,82695 | 0,57602 | 7,6505E10 | 1,32816E11 |

### Means Comparisons

#### Bonferroni Test

| | MeanDiff | SEM | t Value | Prob | Alpha | Sig | LCL | UCL |
|---|---|---|---|---|---|---|---|---|
| Control, 7 days Control, 1 day | -3,95623E11 | 4,19035E10 | -9,44128 | 2,51879E-10 | 0,05 | 1 | -5,47304E11 | -2,43942E11 |
| Control, 14 days Control, 1 day | -4,33496E11 | 4,26724E10 | -10,15869 | 2,70722E-11 | 0,05 | 1 | -5,8796E11 | -2,79032E11 |
| Control, 14 days Control, 7 days | -3,78731E10 | 3,51516E10 | -1,07742 | 1 | 0,05 | 0 | -1,65114E11 | 8,93677E10 |
| Control, 28 days Control, 1 day | -4,3279E11 | 4,63261E10 | -9,34226 | 3,4438E-10 | 0,05 | 1 | -6,0048E11 | -2,65101E11 |
| Control, 28 days Control, 7 days | -3,71675E10 | 3,9507E10 | -0,94078 | 1 | 0,05 | 0 | -1,80174E11 | 1,05839E11 |
| Control, 28 days Control, 14 da | 7,05602E8 | 4,03217E10 | 0,0175 | 1 | 0,05 | 0 | -1,4525E11 | 1,46661E11 |
| Control, 42 days Control, 1 day | -4,33975E11 | 5,58713E10 | -7,7674 | 5,75856E-8 | 0,05 | 1 | -6,36216E11 | -2,31733E11 |
| Control, 42 days Control, 7 days | -3,83521E10 | 5,03618E10 | -0,76153 | 1 | 0,05 | 0 | -2,2065E11 | 1,43946E11 |
| Control, 42 days Control, 14 da | -4,78922E8 | 5,10033E10 | -0,00939 | 1 | 0,05 | 0 | -1,85099E11 | 1,84141E11 |
| Control, 42 days Control, 28 da | -1,18452E9 | 5,40972E10 | -0,0219 | 1 | 0,05 | 0 | -1,97004E11 | 1,94635E11 |
| Control, 56 days Control, 1 day | -4,41966E11 | 5,58713E10 | -7,91042 | 3,58152E-8 | 0,05 | 1 | -6,44207E11 | -2,39724E11 |
| Control, 56 days Control, 7 days | -4,63431E10 | 5,03618E10 | -0,9202 | 1 | 0,05 | 0 | -2,28641E11 | 1,35955E11 |
| Control, 56 days Control, 14 da | -8,46993E9 | 5,10033E10 | -0,16607 | 1 | 0,05 | 0 | -1,9309E11 | 1,7615E11 |
| Control, 56 days Control, 28 da | -9,17554E9 | 5,40972E10 | -0,16961 | 1 | 0,05 | 0 | -2,04995E11 | 1,86644E11 |
| Control, 56 days Control, 42 da | -7,99101E9 | 6,24661E10 | -0,12793 | 1 | 0,05 | 0 | -2,34104E11 | 2,18122E11 |
| DHQ, 1 day Control, 1 day | -6,54264E10 | 5,13211E10 | -1,27484 | 1 | 0,05 | 0 | -2,51197E11 | 1,20344E11 |
| DHQ, 1 day Control, 7 days | 3,30196E11 | 4,5261E10 | 7,29539 | 2,7906E-7 | 0,05 | 1 | 1,66362E11 | 4,94031E11 |
| DHQ, 1 day Control, 14 days | 3,6807E11 | 4,59738E10 | 8,00608 | 2,60945E-8 | 0,05 | 1 | 2,01655E11 | 5,34484E11 |
| DHQ, 1 day Control, 28 days | 3,67364E11 | 4,93838E10 | 7,43896 | 1,72393E-7 | 0,05 | 1 | 1,88606E11 | 5,46122E11 |
| DHQ, 1 day Control, 42 days | 3,68548E11 | 5,84317E10 | 6,30734 | 7,86495E-6 | 0,05 | 1 | 1,57039E11 | 5,80058E11 |
| DHQ, 1 day Control, 56 days | 3,7654E11 | 5,84317E10 | 6,4441 | 4,94792E-6 | 0,05 | 1 | 1,6503E11 | 5,88049E11 |
| DHQ, 7 days Control, 1 day | -4,23527E11 | 5,58713E10 | -7,5804 | 1,07407E-7 | 0,05 | 1 | -6,25769E11 | -2,21286E11 |
| DHQ, 7 days Control, 7 days | -2,79042E10 | 5,03618E10 | -0,55408 | 1 | 0,05 | 0 | -2,10202E11 | 1,54394E11 |
| DHQ, 7 days Control, 14 days | 9,96894E9 | 5,10033E10 | 0,19546 | 1 | 0,05 | 0 | -1,74651E11 | 1,94589E11 |
| DHQ, 7 days Control, 28 days | 9,26333E9 | 5,40972E10 | 0,17123 | 1 | 0,05 | 0 | -1,86556E11 | 2,05083E11 |
| DHQ, 7 days Control, 42 days | 1,04479E10 | 6,24661E10 | 0,16726 | 1 | 0,05 | 0 | -2,15665E11 | 2,36561E11 |
| DHQ, 7 days Control, 56 days | 1,84389E10 | 6,24661E10 | 0,29518 | 1 | 0,05 | 0 | -2,07674E11 | 2,44552E11 |
| DHQ, 7 days DHQ, 1 day | -3,58101E11 | 5,84317E10 | -6,12854 | 1,44142E-5 | 0,05 | 1 | -5,6961E11 | -1,46591E11 |
| DHQ, 14 days Control, 1 day | -4,32378E11 | 5,58713E10 | -7,73881 | 6,33328E-8 | 0,05 | 1 | -6,34619E11 | -2,30136E11 |
| DHQ, 14 days Control, 7 days | -3,67547E10 | 5,03618E10 | -0,72981 | 1 | 0,05 | 0 | -2,19053E11 | 1,45543E11 |
| DHQ, 14 days Control, 14 days | 1,11846E9 | 5,10033E10 | 0,02193 | 1 | 0,05 | 0 | -1,83502E11 | 1,85739E11 |
| DHQ, 14 days Control, 28 days | 4,12857E8 | 5,40972E10 | 0,00763 | 1 | 0,05 | 0 | -1,95407E11 | 1,96232E11 |
| DHQ, 14 days Control, 42 days | 1,59738E9 | 6,24661E10 | 0,02557 | 1 | 0,05 | 0 | -2,24515E11 | 2,2771E11 |
| DHQ, 14 days Control, 56 days | 9,58839E9 | 6,24661E10 | 0,1535 | 1 | 0,05 | 0 | -2,16524E11 | 2,35701E11 |
| DHQ, 14 days DHQ, 1 day | -3,66951E11 | 5,84317E10 | -6,28001 | 8,62838E-6 | 0,05 | 1 | -5,7846E11 | -1,55442E11 |
| DHQ, 14 days DHQ, 7 days | -8,85048E9 | 6,24661E10 | -0,14168 | 1 | 0,05 | 0 | -2,34963E11 | 2,17262E11 |
| DHQ, 28 days Control, 1 day | -4,46562E11 | 5,13211E10 | -8,70133 | 2,68094E-9 | 0,05 | 1 | -6,32332E11 | -2,60791E11 |
| DHQ, 28 days Control, 7 days | -5,0939E10 | 4,5261E10 | -1,12545 | 1 | 0,05 | 0 | -2,14773E11 | 1,12895E11 |
| DHQ, 28 days Control, 14 days | -1,30658E10 | 4,59738E10 | -0,2842 | 1 | 0,05 | 0 | -1,7948E11 | 1,53349E11 |
| DHQ, 28 days Control, 28 days | -1,37714E10 | 4,93838E10 | -0,27887 | 1 | 0,05 | 0 | -1,92529E11 | 1,64986E11 |
| DHQ, 28 days Control, 42 days | -1,25869E10 | 5,84317E10 | -0,21541 | 1 | 0,05 | 0 | -2,24096E11 | 1,98922E11 |
| DHQ, 28 days Control, 56 days | -4,59589E9 | 5,84317E10 | -0,07865 | 1 | 0,05 | 0 | -2,16105E11 | 2,06913E11 |
| DHQ, 28 days DHQ, 1 day | -3,81135E11 | 5,40972E10 | -7,04538 | 6,47394E-7 | 0,05 | 1 | -5,76955E11 | -1,85316E11 |
| DHQ, 28 days DHQ, 7 days | -2,30348E10 | 5,84317E10 | -0,39422 | 1 | 0,05 | 0 | -2,34544E11 | 1,88474E11 |
| DHQ, 28 days DHQ, 14 days | -1,41843E10 | 5,84317E10 | -0,24275 | 1 | 0,05 | 0 | -2,25693E11 | 1,97325E11 |
| DHQ, 42 days Control, 1 day | -4,55703E11 | 5,58713E10 | -8,15628 | 1,5897E-8 | 0,05 | 1 | -6,57944E11 | -2,53461E11 |
| DHQ, 42 days Control, 7 days | -6,00797E10 | 5,03618E10 | -1,19296 | 1 | 0,05 | 0 | -2,42378E11 | 1,22218E11 |
| DHQ, 42 days Control, 14 days | -2,22065E10 | 5,10033E10 | -0,43539 | 1 | 0,05 | 0 | -2,06827E11 | 1,62414E11 |
| DHQ, 42 days Control, 28 days | -2,29121E10 | 5,40972E10 | -0,42354 | 1 | 0,05 | 0 | -2,18732E11 | 1,72907E11 |
| DHQ, 42 days Control, 42 days | -2,17276E10 | 6,24661E10 | -0,34783 | 1 | 0,05 | 0 | -2,4784E11 | 2,04385E11 |
| DHQ, 42 days Control, 56 days | -1,37366E10 | 6,24661E10 | -0,21991 | 1 | 0,05 | 0 | -2,39849E11 | 2,12376E11 |
| DHQ, 42 days DHQ, 1 day | -3,90276E11 | 5,84317E10 | -6,67919 | 2,23153E-6 | 0,05 | 1 | -6,01785E11 | -1,78767E11 |
| DHQ, 42 days DHQ, 7 days | -3,21755E10 | 6,24661E10 | -0,51509 | 1 | 0,05 | 0 | -2,58288E11 | 1,93937E11 |
| DHQ, 42 days DHQ, 14 days | -2,3325E10 | 6,24661E10 | -0,3734 | 1 | 0,05 | 0 | -2,49438E11 | 2,02788E11 |
| DHQ, 42 days DHQ, 28 days | -9,14071E9 | 5,84317E10 | -0,15643 | 1 | 0,05 | 0 | -2,2065E11 | 2,02368E11 |
| DHQ, 56 days Control, 1 day | -4,45042E11 | 5,58713E10 | -7,96548 | 2,98448E-8 | 0,05 | 1 | -6,47284E11 | -2,42801E11 |
| DHQ, 56 days Control, 7 days | -4,94193E10 | 5,03618E10 | -0,98129 | 1 | 0,05 | 0 | -2,31717E11 | 1,32879E11 |
| DHQ, 56 days Control, 14 days | -1,15462E10 | 5,10033E10 | -0,22638 | 1 | 0,05 | 0 | -1,96167E11 | 1,73074E11 |
| DHQ, 56 days Control, 28 days | -1,22518E10 | 5,40972E10 | -0,22648 | 1 | 0,05 | 0 | -2,08071E11 | 1,83568E11 |
| DHQ, 56 days Control, 42 days | -1,10673E10 | 6,24661E10 | -0,17717 | 1 | 0,05 | 0 | -2,3718E11 | 2,15046E11 |
| DHQ, 56 days Control, 56 days | -3,07625E9 | 6,24661E10 | -0,04925 | 1 | 0,05 | 0 | -2,29189E11 | 2,23037E11 |
| DHQ, 56 days DHQ, 1 day | -3,79616E11 | 5,84317E10 | -6,49675 | 4,13951E-6 | 0,05 | 1 | -5,91125E11 | -1,68107E11 |
| DHQ, 56 days DHQ, 7 days | -2,15151E10 | 6,24661E10 | -0,34443 | 1 | 0,05 | 0 | -2,47628E11 | 2,04598E11 |
| DHQ, 56 days DHQ, 14 days | -1,26646E10 | 6,24661E10 | -0,20274 | 1 | 0,05 | 0 | -2,38777E11 | 2,13448E11 |
| DHQ, 56 days DHQ, 28 days | 1,51964E9 | 5,84317E10 | 0,02601 | 1 | 0,05 | 0 | -2,0999E11 | 2,13029E11 |
| DHQ, 56 days DHQ, 42 days | 1,06604E10 | 6,24661E10 | 0,17066 | 1 | 0,05 | 0 | -2,15452E11 | 2,36773E11 |

#### Scheffe Test

| | MeanDiff | SEM | F Value | Prob | Alpha | Sig | LCL | UCL |
|---|---|---|---|---|---|---|---|---|
| Control, 7 days Control, 1 day | -3,95623E11 | 4,19035E10 | 8,10344 | 1,69762E-7 | 0,05 | 1 | -5,92857E11 | -1,98389E11 |
| Control, 14 days Control, 1 day | -4,33496E11 | 4,26724E10 | 9,38172 | 2,26284E-8 | 0,05 | 1 | -6,34349E11 | -2,32643E11 |
| Control, 14 days Control, 7 days | -3,78731E10 | 3,51516E10 | 0,10553 | 0,99984 | 0,05 | 0 | -2,03327E11 | 1,27581E11 |
| Control, 28 days Control, 1 day | -4,3279E11 | 4,63261E10 | 7,93435 | 2,24635E-7 | 0,05 | 1 | -6,50841E11 | -2,1474E11 |
| Control, 28 days Control, 7 days | -3,71675E10 | 3,9507E10 | 0,08046 | 0,99996 | 0,05 | 0 | -2,23121E11 | 1,48786E11 |
| Control, 28 days Control, 14 da | 7,05602E8 | 4,03217E10 | 2,78388E-5 | 1 | 0,05 | 0 | -1,89083E11 | 1,90494E11 |
| Control, 42 days Control, 1 day | -4,33975E11 | 5,58713E10 | 5,48477 | 1,96826E-5 | 0,05 | 1 | -6,96953E11 | -1,70996E11 |
| Control, 42 days Control, 7 days | -3,83521E10 | 5,03618E10 | 0,05272 | 1 | 0,05 | 0 | -2,75398E11 | 1,98694E11 |
| Control, 42 days Control, 14 da | -4,78922E8 | 5,10033E10 | 8,01566E-6 | 1 | 0,05 | 0 | -2,40544E11 | 2,39586E11 |
| Control, 42 days Control, 28 da | -1,18452E9 | 5,40972E10 | 4,35858E-5 | 1 | 0,05 | 0 | -2,55812E11 | 2,53443E11 |
| Control, 56 days Control, 1 day | -4,41966E11 | 5,58713E10 | 5,68862 | 1,31312E-5 | 0,05 | 1 | -7,04944E11 | -1,78987E11 |
| Control, 56 days Control, 7 days | -4,63431E10 | 5,03618E10 | 0,07698 | 0,99997 | 0,05 | 0 | -2,83389E11 | 1,90703E11 |
| Control, 56 days Control, 14 da | -8,46993E9 | 5,10033E10 | 0,00251 | 1 | 0,05 | 0 | -2,48535E11 | 2,31595E11 |
| Control, 56 days Control, 28 da | -9,17554E9 | 5,40972E10 | 0,00262 | 1 | 0,05 | 0 | -2,63803E11 | 2,45452E11 |
| Control, 56 days Control, 42 da | -7,99101E9 | 6,24661E10 | 0,00149 | 1 | 0,05 | 0 | -3,0201E11 | 2,86028E11 |
| DHQ, 1 day Control, 1 day | -6,54264E10 | 5,13211E10 | 0,14775 | 0,99919 | 0,05 | 0 | -3,06988E11 | 1,76135E11 |
| DHQ, 1 day Control, 7 days | 3,30196E11 | 4,5261E10 | 4,83843 | 7,41616E-5 | 0,05 | 1 | 1,1716E11 | 5,43233E11 |
| DHQ, 1 day Control, 14 days | 3,6807E11 | 4,59738E10 | 5,82702 | 1,00121E-5 | 0,05 | 1 | 1,51678E11 | 5,84462E11 |
| DHQ, 1 day Control, 28 days | 3,67364E11 | 4,93838E10 | 5,03074 | 4,96291E-5 | 0,05 | 1 | 1,34922E11 | 5,99806E11 |
| DHQ, 1 day Control, 42 days | 3,68548E11 | 5,84317E10 | 3,6166 | 0,0011 | 0,05 | 1 | 9,3519E10 | 6,43578E11 |
| DHQ, 1 day Control, 56 days | 3,7654E11 | 5,84317E10 | 3,77513 | 7,61866E-4 | 0,05 | 1 | 1,0151E11 | 6,51569E11 |
| DHQ, 7 days Control, 1 day | -4,23527E11 | 5,58713E10 | 5,22386 | 3,33552E-5 | 0,05 | 1 | -6,86506E11 | -1,60549E11 |
| DHQ, 7 days Control, 7 days | -2,79042E10 | 5,03618E10 | 0,02791 | 1 | 0,05 | 0 | -2,6495E11 | 2,09141E11 |
| DHQ, 7 days Control, 14 days | 9,96894E9 | 5,10033E10 | 0,00347 | 1 | 0,05 | 0 | -2,30096E11 | 2,50034E11 |
| DHQ, 7 days Control, 28 days | 9,26333E9 | 5,40972E10 | 0,00267 | 1 | 0,05 | 0 | -2,45364E11 | 2,63891E11 |
| DHQ, 7 days Control, 42 days | 1,04479E10 | 6,24661E10 | 0,00254 | 1 | 0,05 | 0 | -2,83571E11 | 3,04467E11 |
| DHQ, 7 days Control, 56 days | 1,84389E10 | 6,24661E10 | 0,00792 | 1 | 0,05 | 0 | -2,7558E11 | 3,12458E11 |
| DHQ, 7 days DHQ, 1 day | -3,58101E11 | 5,84317E10 | 3,41445 | 0,00175 | 0,05 | 1 | -6,3313E11 | -8,30712E10 |
| DHQ, 14 days Control, 1 day | -4,32378E11 | 5,58713E10 | 5,44447 | 2,13385E-5 | 0,05 | 1 | -6,95356E11 | -1,69399E11 |
| DHQ, 14 days Control, 7 days | -3,67547E10 | 5,03618E10 | 0,04842 | 1 | 0,05 | 0 | -2,738E11 | 2,00291E11 |
| DHQ, 14 days Control, 14 days | 1,11846E9 | 5,10033E10 | 4,3717E-5 | 1 | 0,05 | 0 | -2,38947E11 | 2,41184E11 |
| DHQ, 14 days Control, 28 days | 4,12857E8 | 5,40972E10 | 5,29489E-6 | 1 | 0,05 | 0 | -2,54215E11 | 2,55041E11 |
| DHQ, 14 days Control, 42 days | 1,59738E9 | 6,24661E10 | 5,94478E-5 | 1 | 0,05 | 0 | -2,92421E11 | 2,95616E11 |
| DHQ, 14 days Control, 56 days | 9,58839E9 | 6,24661E10 | 0,00214 | 1 | 0,05 | 0 | -2,8443E11 | 3,03607E11 |
| DHQ, 14 days DHQ, 1 day | -3,66951E11 | 5,84317E10 | 3,58532 | 0,00118 | 0,05 | 1 | -6,41981E11 | -9,19216E10 |
| DHQ, 14 days DHQ, 7 days | -8,85048E9 | 6,24661E10 | 0,00182 | 1 | 0,05 | 0 | -3,02869E11 | 2,85168E11 |
| DHQ, 28 days Control, 1 day | -4,46562E11 | 5,13211E10 | 6,88301 | 1,38636E-6 | 0,05 | 1 | -6,88123E11 | -2,05001E11 |
| DHQ, 28 days Control, 7 days | -5,0939E10 | 4,5261E10 | 0,11515 | 0,99975 | 0,05 | 0 | -2,63976E11 | 1,62098E11 |
| DHQ, 28 days Control, 14 days | -1,30658E10 | 4,59738E10 | 0,00734 | 1 | 0,05 | 0 | -2,29458E11 | 2,03326E11 |
| DHQ, 28 days Control, 28 days | -1,37714E10 | 4,93838E10 | 0,00707 | 1 | 0,05 | 0 | -2,46214E11 | 2,18671E11 |
| DHQ, 28 days Control, 42 days | -1,25869E10 | 5,84317E10 | 0,00422 | 1 | 0,05 | 0 | -2,87616E11 | 2,62443E11 |
| DHQ, 28 days Control, 56 days | -4,59589E9 | 5,84317E10 | 5,62407E-4 | 1 | 0,05 | 0 | -2,79625E11 | 2,70434E11 |
| DHQ, 28 days DHQ, 1 day | -3,81135E11 | 5,40972E10 | 4,51249 | 1,48524E-4 | 0,05 | 1 | -6,35763E11 | -1,26508E11 |
| DHQ, 28 days DHQ, 7 days | -2,30348E10 | 5,84317E10 | 0,01413 | 1 | 0,05 | 0 | -2,98064E11 | 2,51995E11 |
| DHQ, 28 days DHQ, 14 days | -1,41843E10 | 5,84317E10 | 0,00536 | 1 | 0,05 | 0 | -2,89214E11 | 2,60845E11 |
| DHQ, 42 days Control, 1 day | -4,55703E11 | 5,58713E10 | 6,04772 | 6,53593E-6 | 0,05 | 1 | -7,18681E11 | -1,92724E11 |
| DHQ, 42 days Control, 7 days | -6,00797E10 | 5,03618E10 | 0,12938 | 0,99957 | 0,05 | 0 | -2,97125E11 | 1,76966E11 |
| DHQ, 42 days Control, 14 days | -2,22065E10 | 5,10033E10 | 0,01723 | 1 | 0,05 | 0 | -2,62272E11 | 2,17859E11 |
| DHQ, 42 days Control, 28 days | -2,29121E10 | 5,40972E10 | 0,01631 | 1 | 0,05 | 0 | -2,7754E11 | 2,31716E11 |
| DHQ, 42 days Control, 42 days | -2,17276E10 | 6,24661E10 | 0,011 | 1 | 0,05 | 0 | -3,15746E11 | 2,72291E11 |
| DHQ, 42 days Control, 56 days | -1,37366E10 | 6,24661E10 | 0,0044 | 1 | 0,05 | 0 | -3,07755E11 | 2,80282E11 |
| DHQ, 42 days DHQ, 1 day | -3,90276E11 | 5,84317E10 | 4,0556 | 4,04846E-4 | 0,05 | 1 | -6,65306E11 | -1,15247E11 |
| DHQ, 42 days DHQ, 7 days | -3,21755E10 | 6,24661E10 | 0,02412 | 1 | 0,05 | 0 | -3,26194E11 | 2,61843E11 |
| DHQ, 42 days DHQ, 14 days | -2,3325E10 | 6,24661E10 | 0,01268 | 1 | 0,05 | 0 | -3,17344E11 | 2,70694E11 |
| DHQ, 42 days DHQ, 28 days | -9,14071E9 | 5,84317E10 | 0,00222 | 1 | 0,05 | 0 | -2,8417E11 | 2,65889E11 |
| DHQ, 56 days Control, 1 day | -4,45042E11 | 5,58713E10 | 5,76808 | 1,12338E-5 | 0,05 | 1 | -7,08021E11 | -1,82064E11 |
| DHQ, 56 days Control, 7 days | -4,94193E10 | 5,03618E10 | 0,08754 | 0,99994 | 0,05 | 0 | -2,86465E11 | 1,87626E11 |
| DHQ, 56 days Control, 14 days | -1,15462E10 | 5,10033E10 | 0,00466 | 1 | 0,05 | 0 | -2,51612E11 | 2,28519E11 |
| DHQ, 56 days Control, 28 days | -1,22518E10 | 5,40972E10 | 0,00466 | 1 | 0,05 | 0 | -2,6688E11 | 2,42376E11 |
| DHQ, 56 days Control, 42 days | -1,10673E10 | 6,24661E10 | 0,00285 | 1 | 0,05 | 0 | -3,05086E11 | 2,82952E11 |
| DHQ, 56 days Control, 56 days | -3,07625E9 | 6,24661E10 | 2,20476E-4 | 1 | 0,05 | 0 | -2,97095E11 | 2,90943E11 |
| DHQ, 56 days DHQ, 1 day | -3,79616E11 | 5,84317E10 | 3,83707 | 6,61873E-4 | 0,05 | 1 | -6,54645E11 | -1,04586E11 |
| DHQ, 56 days DHQ, 7 days | -2,15151E10 | 6,24661E10 | 0,01078 | 1 | 0,05 | 0 | -3,15534E11 | 2,72504E11 |
| DHQ, 56 days DHQ, 14 days | -1,26646E10 | 6,24661E10 | 0,00374 | 1 | 0,05 | 0 | -3,06684E11 | 2,81354E11 |
| DHQ, 56 days DHQ, 28 days | 1,51964E9 | 5,84317E10 | 6,14885E-5 | 1 | 0,05 | 0 | -2,7351E11 | 2,76549E11 |
| DHQ, 56 days DHQ, 42 days | 1,06604E10 | 6,24661E10 | 0,00265 | 1 | 0,05 | 0 | -2,83359E11 | 3,04679E11 |

#### Tukey Test

| | MeanDiff | SEM | q Value | Prob | Alpha | Sig | LCL | UCL |
|---|---|---|---|---|---|---|---|---|
| Control, 7 days Control, 1 day | -3,95623E11 | 4,19035E10 | 13,35199 | 2,50188E-9 | 0,05 | 1 | -5,40153E11 | -2,51092E11 |
| Control, 14 days Control, 1 day | -4,33496E11 | 4,26724E10 | 14,36655 | 0 | 0,05 | 1 | -5,80679E11 | -2,86313E11 |
| Control, 14 days Control, 7 days | -3,78731E10 | 3,51516E10 | 1,52371 | 0,99422 | 0,05 | 0 | -1,59116E11 | 8,33692E10 |
| Control, 28 days Control, 1 day | -4,3279E11 | 4,63261E10 | 13,21196 | 3,19937E-9 | 0,05 | 1 | -5,92575E11 | -2,73006E11 |
| Control, 28 days Control, 7 days | -3,71675E10 | 3,9507E10 | 1,33047 | 0,9982 | 0,05 | 0 | -1,73432E11 | 9,90971E10 |
| Control, 28 days Control, 14 da | 7,05602E8 | 4,03217E10 | 0,02475 | 1 | 0,05 | 0 | -1,38369E11 | 1,3978E11 |
| Control, 42 days Control, 1 day | -4,33975E11 | 5,58713E10 | 10,98476 | 1,66955E-7 | 0,05 | 1 | -6,26682E11 | -2,41268E11 |
| Control, 42 days Control, 7 days | -3,83521E10 | 5,03618E10 | 1,07697 | 0,99974 | 0,05 | 0 | -2,12056E11 | 1,35352E11 |
| Control, 42 days Control, 14 da | -4,78922E8 | 5,10033E10 | 0,01328 | 1 | 0,05 | 0 | -1,76396E11 | 1,75438E11 |
| Control, 42 days Control, 28 da | -1,18452E9 | 5,40972E10 | 0,03097 | 1 | 0,05 | 0 | -1,87773E11 | 1,85404E11 |
| Control, 56 days Control, 1 day | -4,41966E11 | 5,58713E10 | 11,18703 | 1,44116E-7 | 0,05 | 1 | -6,34673E11 | -2,49259E11 |
| Control, 56 days Control, 7 days | -4,63431E10 | 5,03618E10 | 1,30136 | 0,99852 | 0,05 | 0 | -2,20047E11 | 1,27361E11 |
| Control, 56 days Control, 14 da | -8,46993E9 | 5,10033E10 | 0,23485 | 1 | 0,05 | 0 | -1,84387E11 | 1,67447E11 |
| Control, 56 days Control, 28 da | -9,17554E9 | 5,40972E10 | 0,23987 | 1 | 0,05 | 0 | -1,95764E11 | 1,77413E11 |
| Control, 56 days Control, 42 da | -7,99101E9 | 6,24661E10 | 0,18091 | 1 | 0,05 | 0 | -2,23444E11 | 2,07462E11 |
| DHQ, 1 day Control, 1 day | -6,54264E10 | 5,13211E10 | 1,8029 | 0,97819 | 0,05 | 0 | -2,42439E11 | 1,11587E11 |
| DHQ, 1 day Control, 7 days | 3,30196E11 | 4,5261E10 | 10,31724 | 3,62189E-7 | 0,05 | 1 | 1,74086E11 | 4,86307E11 |
| DHQ, 1 day Control, 14 days | 3,6807E11 | 4,59738E10 | 11,3223 | 1,33453E-7 | 0,05 | 1 | 2,095E11 | 5,26639E11 |
| DHQ, 1 day Control, 28 days | 3,67364E11 | 4,93838E10 | 10,52028 | 2,80611E-7 | 0,05 | 1 | 1,97033E11 | 5,37695E11 |
| DHQ, 1 day Control, 42 days | 3,68548E11 | 5,84317E10 | 8,91993 | 7,27557E-6 | 0,05 | 1 | 1,6701E11 | 5,70087E11 |
| DHQ, 1 day Control, 56 days | 3,7654E11 | 5,84317E10 | 9,11334 | 4,60383E-6 | 0,05 | 1 | 1,75001E11 | 5,78078E11 |
| DHQ, 7 days Control, 1 day | -4,23527E11 | 5,58713E10 | 10,7203 | 2,17016E-7 | 0,05 | 1 | -6,16234E11 | -2,3082E11 |
| DHQ, 7 days Control, 7 days | -2,79042E10 | 5,03618E10 | 0,78358 | 0,99999 | 0,05 | 0 | -2,01608E11 | 1,458E11 |
| DHQ, 7 days Control, 14 days | 9,96894E9 | 5,10033E10 | 0,27642 | 1 | 0,05 | 0 | -1,65948E11 | 1,85886E11 |
| DHQ, 7 days Control, 28 days | 9,26333E9 | 5,40972E10 | 0,24216 | 1 | 0,05 | 0 | -1,77325E11 | 1,95851E11 |
| DHQ, 7 days Control, 42 days | 1,04479E10 | 6,24661E10 | 0,23654 | 1 | 0,05 | 0 | -2,05006E11 | 2,25901E11 |
| DHQ, 7 days Control, 56 days | 1,84389E10 | 6,24661E10 | 0,41745 | 1 | 0,05 | 0 | -1,97015E11 | 2,33892E11 |
| DHQ, 7 days DHQ, 1 day | -3,58101E11 | 5,84317E10 | 8,66706 | 1,32302E-5 | 0,05 | 1 | -5,59639E11 | -1,56562E11 |
| DHQ, 14 days Control, 1 day | -4,32378E11 | 5,58713E10 | 10,94432 | 1,72835E-7 | 0,05 | 1 | -6,25085E11 | -2,3967E11 |
| DHQ, 14 days Control, 7 days | -3,67547E10 | 5,03618E10 | 1,03211 | 0,99983 | 0,05 | 0 | -2,10459E11 | 1,36949E11 |
| DHQ, 14 days Control, 14 days | 1,11846E9 | 5,10033E10 | 0,03101 | 1 | 0,05 | 0 | -1,74798E11 | 1,77035E11 |
| DHQ, 14 days Control, 28 days | 4,12857E8 | 5,40972E10 | 0,01079 | 1 | 0,05 | 0 | -1,86175E11 | 1,87001E11 |
| DHQ, 14 days Control, 42 days | 1,59738E9 | 6,24661E10 | 0,03616 | 1 | 0,05 | 0 | -2,13856E11 | 2,17051E11 |
| DHQ, 14 days Control, 56 days | 9,58839E9 | 6,24661E10 | 0,21708 | 1 | 0,05 | 0 | -2,05865E11 | 2,25042E11 |
| DHQ, 14 days DHQ, 1 day | -3,66951E11 | 5,84317E10 | 8,88127 | 7,9725E-6 | 0,05 | 1 | -5,68489E11 | -1,65413E11 |
| DHQ, 14 days DHQ, 7 days | -8,85048E9 | 6,24661E10 | 0,20037 | 1 | 0,05 | 0 | -2,24304E11 | 2,06603E11 |
| DHQ, 28 days Control, 1 day | -4,46562E11 | 5,13211E10 | 12,30554 | 9,92787E-9 | 0,05 | 1 | -6,23575E11 | -2,69549E11 |
| DHQ, 28 days Control, 7 days | -5,0939E10 | 4,5261E10 | 1,59163 | 0,99173 | 0,05 | 0 | -2,0705E11 | 1,05172E11 |
| DHQ, 28 days Control, 14 days | -1,30658E10 | 4,59738E10 | 0,40192 | 1 | 0,05 | 0 | -1,71635E11 | 1,45504E11 |
| DHQ, 28 days Control, 28 days | -1,37714E10 | 4,93838E10 | 0,39438 | 1 | 0,05 | 0 | -1,84102E11 | 1,56559E11 |
| DHQ, 28 days Control, 42 days | -1,25869E10 | 5,84317E10 | 0,30464 | 1 | 0,05 | 0 | -2,14125E11 | 1,88951E11 |
| DHQ, 28 days Control, 56 days | -4,59589E9 | 5,84317E10 | 0,11123 | 1 | 0,05 | 0 | -2,06134E11 | 1,96942E11 |
| DHQ, 28 days DHQ, 1 day | -3,81135E11 | 5,40972E10 | 9,96367 | 7,10588E-7 | 0,05 | 1 | -5,67723E11 | -1,94547E11 |
| DHQ, 28 days DHQ, 7 days | -2,30348E10 | 5,84317E10 | 0,55751 | 1 | 0,05 | 0 | -2,24573E11 | 1,78503E11 |
| DHQ, 28 days DHQ, 14 days | -1,41843E10 | 5,84317E10 | 0,3433 | 1 | 0,05 | 0 | -2,15722E11 | 1,87354E11 |
| DHQ, 42 days Control, 1 day | -4,55703E11 | 5,58713E10 | 11,53473 | 1,21573E-7 | 0,05 | 1 | -6,4841E11 | -2,62995E11 |
| DHQ, 42 days Control, 7 days | -6,00797E10 | 5,03618E10 | 1,6871 | 0,98685 | 0,05 | 0 | -2,33784E11 | 1,13624E11 |
| DHQ, 42 days Control, 14 days | -2,22065E10 | 5,10033E10 | 0,61574 | 1 | 0,05 | 0 | -1,98123E11 | 1,5371E11 |
| DHQ, 42 days Control, 28 days | -2,29121E10 | 5,40972E10 | 0,59897 | 1 | 0,05 | 0 | -2,095E11 | 1,63676E11 |
| DHQ, 42 days Control, 42 days | -2,17276E10 | 6,24661E10 | 0,49191 | 1 | 0,05 | 0 | -2,37181E11 | 1,93726E11 |
| DHQ, 42 days Control, 56 days | -1,37366E10 | 6,24661E10 | 0,31099 | 1 | 0,05 | 0 | -2,2919E11 | 2,01717E11 |
| DHQ, 42 days DHQ, 1 day | -3,90276E11 | 5,84317E10 | 9,4458 | 2,1945E-6 | 0,05 | 1 | -5,91814E11 | -1,88738E11 |
| DHQ, 42 days DHQ, 7 days | -3,21755E10 | 6,24661E10 | 0,72844 | 0,99999 | 0,05 | 0 | -2,47629E11 | 1,83278E11 |
| DHQ, 42 days DHQ, 14 days | -2,3325E10 | 6,24661E10 | 0,52807 | 1 | 0,05 | 0 | -2,38778E11 | 1,92128E11 |
| DHQ, 42 days DHQ, 28 days | -9,14071E9 | 5,84317E10 | 0,22123 | 1 | 0,05 | 0 | -2,10679E11 | 1,92397E11 |
| DHQ, 56 days Control, 1 day | -4,45042E11 | 5,58713E10 | 11,26489 | 1,37617E-7 | 0,05 | 1 | -6,3775E11 | -2,52335E11 |
| DHQ, 56 days Control, 7 days | -4,94193E10 | 5,03618E10 | 1,38775 | 0,99739 | 0,05 | 0 | -2,23123E11 | 1,24285E11 |
| DHQ, 56 days Control, 14 days | -1,15462E10 | 5,10033E10 | 0,32015 | 1 | 0,05 | 0 | -1,87463E11 | 1,64371E11 |
| DHQ, 56 days Control, 28 days | -1,22518E10 | 5,40972E10 | 0,32029 | 1 | 0,05 | 0 | -1,9884E11 | 1,74336E11 |
| DHQ, 56 days Control, 42 days | -1,10673E10 | 6,24661E10 | 0,25056 | 1 | 0,05 | 0 | -2,26521E11 | 2,04386E11 |
| DHQ, 56 days Control, 56 days | -3,07625E9 | 6,24661E10 | 0,06965 | 1 | 0,05 | 0 | -2,1853E11 | 2,12377E11 |
| DHQ, 56 days DHQ, 1 day | -3,79616E11 | 5,84317E10 | 9,18779 | 3,94613E-6 | 0,05 | 1 | -5,81154E11 | -1,78078E11 |
| DHQ, 56 days DHQ, 7 days | -2,15151E10 | 6,24661E10 | 0,4871 | 1 | 0,05 | 0 | -2,36968E11 | 1,93938E11 |
| DHQ, 56 days DHQ, 14 days | -1,26646E10 | 6,24661E10 | 0,28672 | 1 | 0,05 | 0 | -2,28118E11 | 2,02789E11 |
| DHQ, 56 days DHQ, 28 days | 1,51964E9 | 5,84317E10 | 0,03678 | 1 | 0,05 | 0 | -2,00019E11 | 2,03058E11 |
| DHQ, 56 days DHQ, 42 days | 1,06604E10 | 6,24661E10 | 0,24135 | 1 | 0,05 | 0 | -2,04793E11 | 2,26114E11 |

### Grouping Letters

#### Bonferroni Test

| | Mean | Groups | |
|---|---|---|---|
| Control, 1 day | 4,97105E11 | A | |
| DHQ, 1 day | 4,31678E11 | A | |
| Control, 7 days | 1,01482E11 | | B |
| DHQ, 7 days | 7,35776E10 | | B |
| DHQ, 14 days | 6,47271E10 | | B |
| Control, 28 days | 6,43143E10 | | B |
| Control, 14 days | 6,36087E10 | | B |
| Control, 42 days | 6,31298E10 | | B |
| Control, 56 days | 5,51388E10 | | B |
| DHQ, 56 days | 5,20625E10 | | B |
| DHQ, 28 days | 5,05429E10 | | B |
| DHQ, 42 days | 4,14021E10 | | B |

Means that do not share a letter are significantly different.

#### Scheffe Test

| | Mean | Groups | |
|---|---|---|---|
| Control, 1 day | 4,97105E11 | A | |
| DHQ, 1 day | 4,31678E11 | A | |
| Control, 7 days | 1,01482E11 | | B |
| DHQ, 7 days | 7,35776E10 | | B |
| DHQ, 14 days | 6,47271E10 | | B |
| Control, 28 days | 6,43143E10 | | B |
| Control, 14 days | 6,36087E10 | | B |
| Control, 42 days | 6,31298E10 | | B |
| Control, 56 days | 5,51388E10 | | B |
| DHQ, 56 days | 5,20625E10 | | B |
| DHQ, 28 days | 5,05429E10 | | B |
| DHQ, 42 days | 4,14021E10 | | B |

Means that do not share a letter are significantly different.

#### Tukey Test

| | Mean | Groups | |
|---|---|---|---|
| Control, 1 day | 4,97105E11 | A | |
| DHQ, 1 day | 4,31678E11 | A | |
| Control, 7 days | 1,01482E11 | | B |
| DHQ, 7 days | 7,35776E10 | | B |
| DHQ, 14 days | 6,47271E10 | | B |
| Control, 28 days | 6,43143E10 | | B |
| Control, 14 days | 6,36087E10 | | B |
| Control, 42 days | 6,31298E10 | | B |
| Control, 56 days | 5,51388E10 | | B |
| DHQ, 56 days | 5,20625E10 | | B |
| DHQ, 28 days | 5,05429E10 | | B |
| DHQ, 42 days | 4,14021E10 | | B |

Means that do not share a letter are significantly different.

Sig equals 1 indicates that the difference of the means is significant at the 0,05 level.
Sig equals 0 indicates that the difference of the means is not significant at the 0,05 level.

## Powers

| | Alpha | Sample Size | Power |
|---|---|---|---|
| Actual Power | 0,05 | 56 | 1 |

*ANOVAOneWay (27.10.2024 21:46:54)*

## Input Data

| | Data | Range |
|---|---|---|
| Control, 1 day | [Book1]"Budding cells"!A"Control, 1 day" | [1*:6*] |
| Control, 7 days | [Book1]"Budding cells"!B"Control, 7 days" | [1*:7*] |
| Control, 14 days | [Book1]"Budding cells"!C"Control, 14 days" | [1*:8*] |
| Control, 28 days | [Book1]"Budding cells"!D"Control, 28 days" | [1*:6*] |
| Control, 42 days | [Book1]"Budding cells"!E"Control, 42 days" | [1*:3*] |
| Control, 56 days | [Book1]"Budding cells"!F"Control, 56 days" | [1*:3*] |
| DHQ, 1 day | [Book1]"Budding cells"!G"DHQ, 1 d | [1*:5*] |
| DHQ, 7 days | [Book1]"Budding cells"!H"DHQ, 7 days" | [1*:3*] |
| DHQ, 14 days | [Book1]"Budding cells"!I"DHQ, 14 days" | [1*:3*] |
| DHQ, 28 days | [Book1]"Budding cells"!J"DHQ, 28 days" | [1*:3*] |
| DHQ, 42 days | [Book1]"Budding cells"!K"DHQ, 42 days" | [1*:3*] |
| DHQ, 56 days | [Book1]"Budding cells"!L"DHQ, 56 days" | [1*:3*] |

## Descriptive Statistics

| | N Analysis | N Missing | Mean | Standard Deviation | SE of Mean |
|---|---|---|---|---|---|
| Control, 1 day | 6 | 0 | 0,19544 | 0,07741 | 0,0316 |
| Control, 7 days | 7 | 0 | 0,22942 | 0,16541 | 0,06252 |
| Control, 14 days | 8 | 0 | 0,24789 | 0,15503 | 0,05481 |
| Control, 28 days | 6 | 0 | 0,18491 | 0,12425 | 0,05072 |
| Control, 42 days | 3 | 0 | 0,21412 | 0,02633 | 0,0152 |
| Control, 56 days | 3 | 0 | 0,30514 | 0,11963 | 0,06907 |
| DHQ, 1 day | 5 | 0 | 0,21962 | 0,06598 | 0,02951 |
| DHQ, 7 days | 3 | 0 | 0,04331 | 0,06214 | 0,03588 |
| DHQ, 14 days | 3 | 0 | 0,1499 | 0,15247 | 0,08803 |
| DHQ, 28 days | 3 | 0 | 0,2006 | 0,08199 | 0,04734 |
| DHQ, 42 days | 3 | 0 | 0,17245 | 0,0569 | 0,03285 |
| DHQ, 56 days | 3 | 0 | 0,28163 | 0,06459 | 0,03729 |

## ANOVA

### Overall ANOVA

| | DF | Sum of Squares | Mean Square | F Value | Prob>F |
|---|---|---|---|---|---|
| Model | 11 | 0,16087 | 0,01462 | 1,05291 | 0,42088 |
| Error | 41 | 0,56946 | 0,01389 | | |
| Total | 52 | 0,73033 | | | |

Null Hypothesis: The means of all levels are equal.
Alternative Hypothesis: The means of one or more levels are different.
At the 0.05 level, the population means are not significantly different.

### Fit Statistics

| | R-Square | Coeff Var | Root MSE | Data Mean |
|---|---|---|---|---|
| | 0,22027 | 0,56421 | 0,11785 | 0,20888 |

### Means Comparisons

#### Bonferroni Test

| | MeanDiff | SEM | t Value | Prob | Alpha | Sig | LCL | UCL |
|---|---|---|---|---|---|---|---|---|
| Control, 7 days  Control, 1 day | 0,03399 | 0,06557 | 0,51838 | 1 | 0,05 | 0 | -0,20464 | 0,27262 |
| Control, 14 days  Control, 1 day | 0,05246 | 0,06365 | 0,82422 | 1 | 0,05 | 0 | -0,17919 | 0,28411 |
| Control, 14 days  Control, 7 days | 0,01847 | 0,06099 | 0,30283 | 1 | 0,05 | 0 | -0,20352 | 0,24046 |
| Control, 28 days  Control, 1 day | -0,01053 | 0,06804 | -0,15472 | 1 | 0,05 | 0 | -0,25817 | 0,23711 |
| Control, 28 days  Control, 7 days | -0,04452 | 0,06557 | -0,67894 | 1 | 0,05 | 0 | -0,28315 | 0,19412 |
| Control, 28 days  Control, 14 da | -0,06299 | 0,06365 | -0,98963 | 1 | 0,05 | 0 | -0,29463 | 0,16866 |
| Control, 42 days  Control, 1 day | 0,01869 | 0,08333 | 0,22423 | 1 | 0,05 | 0 | -0,28461 | 0,32198 |
| Control, 42 days  Control, 7 days | -0,0153 | 0,08133 | -0,18816 | 1 | 0,05 | 0 | -0,31129 | 0,28068 |
| Control, 42 days  Control, 14 da | -0,03377 | 0,07979 | -0,4233 | 1 | 0,05 | 0 | -0,32416 | 0,25661 |
| Control, 42 days  Control, 28 da | 0,02921 | 0,08333 | 0,35056 | 1 | 0,05 | 0 | -0,27408 | 0,33251 |
| Control, 56 days  Control, 1 day | 0,10971 | 0,08333 | 1,31646 | 1 | 0,05 | 0 | -0,19359 | 0,413 |
| Control, 56 days  Control, 7 days | 0,07572 | 0,08133 | 0,93104 | 1 | 0,05 | 0 | -0,22027 | 0,37171 |
| Control, 56 days  Control, 14 da | 0,05725 | 0,07979 | 0,7175 | 1 | 0,05 | 0 | -0,23314 | 0,34763 |
| Control, 56 days  Control, 28 da | 0,12023 | 0,08333 | 1,4428 | 1 | 0,05 | 0 | -0,18306 | 0,42353 |
| Control, 56 days  Control, 42 da | 0,09102 | 0,09623 | 0,9459 | 1 | 0,05 | 0 | -0,2592 | 0,44124 |
| DHQ, 1 day  Control, 1 day | 0,02419 | 0,07136 | 0,3389 | 1 | 0,05 | 0 | -0,23554 | 0,28391 |
| DHQ, 1 day  Control, 7 days | -0,0098 | 0,06901 | -0,14206 | 1 | 0,05 | 0 | -0,26096 | 0,24135 |
| DHQ, 1 day  Control, 14 days | -0,02827 | 0,06719 | -0,42084 | 1 | 0,05 | 0 | -0,2728 | 0,21625 |
| DHQ, 1 day  Control, 28 days | 0,03471 | 0,07136 | 0,48643 | 1 | 0,05 | 0 | -0,22501 | 0,29444 |
| DHQ, 1 day  Control, 42 days | 0,0055 | 0,08607 | 0,06389 | 1 | 0,05 | 0 | -0,30774 | 0,31874 |
| DHQ, 1 day  Control, 56 days | -0,08552 | 0,08607 | -0,99366 | 1 | 0,05 | 0 | -0,39876 | 0,22772 |
| DHQ, 7 days  Control, 1 day | -0,15213 | 0,08333 | -1,82554 | 1 | 0,05 | 0 | -0,45543 | 0,15117 |
| DHQ, 7 days  Control, 7 days | -0,18612 | 0,08133 | -2,28855 | 1 | 0,05 | 0 | -0,48211 | 0,10987 |
| DHQ, 7 days  Control, 14 days | -0,20459 | 0,07979 | -2,56421 | 0,93075 | 0,05 | 0 | -0,49497 | 0,08579 |
| DHQ, 7 days  Control, 28 days | -0,1416 | 0,08333 | -1,6992 | 1 | 0,05 | 0 | -0,4449 | 0,16169 |
| DHQ, 7 days  Control, 42 days | -0,17082 | 0,09623 | -1,77515 | 1 | 0,05 | 0 | -0,52103 | 0,1794 |
| DHQ, 7 days  Control, 56 days | -0,26184 | 0,09623 | -2,72105 | 0,6274 | 0,05 | 0 | -0,61205 | 0,08838 |
| DHQ, 7 days  DHQ, 1 day | -0,17632 | 0,08607 | -2,04857 | 1 | 0,05 | 0 | -0,48956 | 0,13693 |
| DHQ, 14 days  Control, 1 day | -0,04553 | 0,08333 | -0,54641 | 1 | 0,05 | 0 | -0,34883 | 0,25776 |
| DHQ, 14 days  Control, 7 days | -0,07952 | 0,08133 | -0,97783 | 1 | 0,05 | 0 | -0,37551 | 0,21646 |
| DHQ, 14 days  Control, 14 days | -0,09799 | 0,07979 | -1,2282 | 1 | 0,05 | 0 | -0,38838 | 0,19239 |
| DHQ, 14 days  Control, 28 days | -0,03501 | 0,08333 | -0,42007 | 1 | 0,05 | 0 | -0,3383 | 0,26829 |
| DHQ, 14 days  Control, 42 days | -0,06422 | 0,09623 | -0,66739 | 1 | 0,05 | 0 | -0,41444 | 0,286 |
| DHQ, 14 days  Control, 56 days | -0,15524 | 0,09623 | -1,61329 | 1 | 0,05 | 0 | -0,50546 | 0,19498 |
| DHQ, 14 days  DHQ, 1 day | -0,06972 | 0,08607 | -0,81006 | 1 | 0,05 | 0 | -0,38296 | 0,24352 |
| DHQ, 14 days  DHQ, 7 days | 0,1066 | 0,09623 | 1,10776 | 1 | 0,05 | 0 | -0,24362 | 0,45681 |
| DHQ, 28 days  Control, 1 day | 0,00516 | 0,08333 | 0,06192 | 1 | 0,05 | 0 | -0,29814 | 0,30846 |
| DHQ, 28 days  Control, 7 days | -0,02883 | 0,08133 | -0,35448 | 1 | 0,05 | 0 | -0,32482 | 0,26716 |
| DHQ, 28 days  Control, 14 days | -0,0473 | 0,07979 | -0,59282 | 1 | 0,05 | 0 | -0,33768 | 0,24308 |
| DHQ, 28 days  Control, 28 days | 0,01569 | 0,08333 | 0,18825 | 1 | 0,05 | 0 | -0,28761 | 0,31898 |
| DHQ, 28 days  Control, 42 days | -0,01353 | 0,09623 | -0,14056 | 1 | 0,05 | 0 | -0,36374 | 0,33669 |
| DHQ, 28 days  Control, 56 days | -0,10455 | 0,09623 | -1,08646 | 1 | 0,05 | 0 | -0,45476 | 0,24567 |
| DHQ, 28 days  DHQ, 1 day | -0,01902 | 0,08607 | -0,22105 | 1 | 0,05 | 0 | -0,33227 | 0,29422 |
| DHQ, 28 days  DHQ, 7 days | 0,15729 | 0,09623 | 1,63459 | 1 | 0,05 | 0 | -0,19293 | 0,50751 |
| DHQ, 28 days  DHQ, 14 days | 0,05069 | 0,09623 | 0,52683 | 1 | 0,05 | 0 | -0,29952 | 0,40091 |
| DHQ, 42 days  Control, 1 day | -0,02298 | 0,08333 | -0,27576 | 1 | 0,05 | 0 | -0,32628 | 0,28032 |
| DHQ, 42 days  Control, 7 days | -0,05697 | 0,08133 | -0,7005 | 1 | 0,05 | 0 | -0,35296 | 0,23902 |
| DHQ, 42 days  Control, 14 days | -0,07544 | 0,07979 | -0,94553 | 1 | 0,05 | 0 | -0,36582 | 0,21494 |
| DHQ, 42 days  Control, 28 days | -0,01245 | 0,08333 | -0,14943 | 1 | 0,05 | 0 | -0,31575 | 0,29084 |
| DHQ, 42 days  Control, 42 days | -0,04167 | 0,09623 | -0,43301 | 1 | 0,05 | 0 | -0,39188 | 0,30855 |
| DHQ, 42 days  Control, 56 days | -0,13269 | 0,09623 | -1,37891 | 1 | 0,05 | 0 | -0,4829 | 0,21753 |
| DHQ, 42 days  DHQ, 1 day | -0,04717 | 0,08607 | -0,54801 | 1 | 0,05 | 0 | -0,36041 | 0,26608 |
| DHQ, 42 days  DHQ, 7 days | 0,12915 | 0,09623 | 1,34214 | 1 | 0,05 | 0 | -0,22107 | 0,47937 |
| DHQ, 42 days  DHQ, 14 days | 0,02255 | 0,09623 | 0,23438 | 1 | 0,05 | 0 | -0,32766 | 0,37277 |
| DHQ, 42 days  DHQ, 28 days | -0,02814 | 0,09623 | -0,29245 | 1 | 0,05 | 0 | -0,37836 | 0,32208 |
| DHQ, 56 days  Control, 1 day | 0,08619 | 0,08333 | 1,03429 | 1 | 0,05 | 0 | -0,2171 | 0,38949 |
| DHQ, 56 days  Control, 7 days | 0,0522 | 0,08133 | 0,6419 | 1 | 0,05 | 0 | -0,24378 | 0,34819 |
| DHQ, 56 days  Control, 14 days | 0,03373 | 0,07979 | 0,42278 | 1 | 0,05 | 0 | -0,25665 | 0,32412 |
| DHQ, 56 days  Control, 28 days | 0,09672 | 0,08333 | 1,16062 | 1 | 0,05 | 0 | -0,20658 | 0,40002 |
| DHQ, 56 days  Control, 42 days | 0,06751 | 0,09623 | 0,70153 | 1 | 0,05 | 0 | -0,28271 | 0,41772 |
| DHQ, 56 days  Control, 56 days | -0,02352 | 0,09623 | -0,24437 | 1 | 0,05 | 0 | -0,37373 | 0,3267 |
| DHQ, 56 days  DHQ, 1 day | 0,06201 | 0,08607 | 0,72044 | 1 | 0,05 | 0 | -0,25124 | 0,37525 |
| DHQ, 56 days  DHQ, 7 days | 0,23832 | 0,09623 | 2,47668 | 1 | 0,05 | 0 | -0,11189 | 0,58854 |
| DHQ, 56 days  DHQ, 14 days | 0,13173 | 0,09623 | 1,36892 | 1 | 0,05 | 0 | -0,21849 | 0,48194 |
| DHQ, 56 days  DHQ, 28 days | 0,08103 | 0,09623 | 0,84209 | 1 | 0,05 | 0 | -0,26919 | 0,43125 |
| DHQ, 56 days  DHQ, 42 days | 0,10917 | 0,09623 | 1,13454 | 1 | 0,05 | 0 | -0,24104 | 0,45939 |

#### Scheffe Test

| | MeanDiff | SEM | F Value | Prob | Alpha | Sig | LCL | UCL |
|---|---|---|---|---|---|---|---|---|
| Control, 7 days  Control, 1 day | 0,03399 | 0,06557 | 0,02443 | 1 | 0,05 | 0 | -0,27594 | 0,34392 |
| Control, 14 days  Control, 1 day | 0,05246 | 0,06365 | 0,06176 | 0,99999 | 0,05 | 0 | -0,2484 | 0,35332 |
| Control, 14 days  Control, 7 days | 0,01847 | 0,06099 | 0,00834 | 1 | 0,05 | 0 | -0,26985 | 0,30679 |
| Control, 28 days  Control, 1 day | -0,01053 | 0,06804 | 0,00218 | 1 | 0,05 | 0 | -0,33216 | 0,3111 |
| Control, 28 days  Control, 7 days | -0,04452 | 0,06557 | 0,04191 | 1 | 0,05 | 0 | -0,35445 | 0,26541 |
| Control, 28 days  Control, 14 da | -0,06299 | 0,06365 | 0,08903 | 0,99993 | 0,05 | 0 | -0,36385 | 0,23787 |
| Control, 42 days  Control, 1 day | 0,01869 | 0,08333 | 0,00457 | 1 | 0,05 | 0 | -0,37523 | 0,4126 |
| Control, 42 days  Control, 7 days | -0,0153 | 0,08133 | 0,00322 | 1 | 0,05 | 0 | -0,39972 | 0,36912 |
| Control, 42 days  Control, 14 da | -0,03377 | 0,07979 | 0,01629 | 1 | 0,05 | 0 | -0,41092 | 0,34337 |
| Control, 42 days  Control, 28 da | 0,02921 | 0,08333 | 0,01117 | 1 | 0,05 | 0 | -0,3647 | 0,42313 |
| Control, 56 days  Control, 1 day | 0,10971 | 0,08333 | 0,15755 | 0,99888 | 0,05 | 0 | -0,28421 | 0,50362 |
| Control, 56 days  Control, 7 days | 0,07572 | 0,08133 | 0,0788 | 0,99996 | 0,05 | 0 | -0,3087 | 0,46014 |
| Control, 56 days  Control, 14 da | 0,05725 | 0,07979 | 0,0468 | 1 | 0,05 | 0 | -0,3199 | 0,43439 |
| Control, 56 days  Control, 28 da | 0,12023 | 0,08333 | 0,18924 | 0,99742 | 0,05 | 0 | -0,27368 | 0,51415 |
| Control, 56 days  Control, 42 da | 0,09102 | 0,09623 | 0,08134 | 0,99995 | 0,05 | 0 | -0,36383 | 0,54587 |
| DHQ, 1 day  Control, 1 day | 0,02419 | 0,07136 | 0,01044 | 1 | 0,05 | 0 | -0,31314 | 0,36151 |
| DHQ, 1 day  Control, 7 days | -0,0098 | 0,06901 | 0,00183 | 1 | 0,05 | 0 | -0,336 | 0,31639 |
| DHQ, 1 day  Control, 14 days | -0,02827 | 0,06719 | 0,0161 | 1 | 0,05 | 0 | -0,34586 | 0,28931 |
| DHQ, 1 day  Control, 28 days | 0,03471 | 0,07136 | 0,02151 | 1 | 0,05 | 0 | -0,30262 | 0,37204 |
| DHQ, 1 day  Control, 42 days | 0,0055 | 0,08607 | 3,71137E-4 | 1 | 0,05 | 0 | -0,40133 | 0,41233 |
| DHQ, 1 day  Control, 56 days | -0,08552 | 0,08607 | 0,08976 | 0,99993 | 0,05 | 0 | -0,49236 | 0,32131 |
| DHQ, 7 days  Control, 1 day | -0,15213 | 0,08333 | 0,30296 | 0,98139 | 0,05 | 0 | -0,54605 | 0,24179 |
| DHQ, 7 days  Control, 7 days | -0,18612 | 0,08133 | 0,47613 | 0,90734 | 0,05 | 0 | -0,57054 | 0,1983 |
| DHQ, 7 days  Control, 14 days | -0,20459 | 0,07979 | 0,59774 | 0,81947 | 0,05 | 0 | -0,58173 | 0,17256 |
| DHQ, 7 days  Control, 28 days | -0,1416 | 0,08333 | 0,26248 | 0,98952 | 0,05 | 0 | -0,53552 | 0,25231 |
| DHQ, 7 days  Control, 42 days | -0,17082 | 0,09623 | 0,28647 | 0,98508 | 0,05 | 0 | -0,62567 | 0,28404 |
| DHQ, 7 days  Control, 56 days | -0,26184 | 0,09623 | 0,6731 | 0,75508 | 0,05 | 0 | -0,71669 | 0,19302 |
| DHQ, 7 days  DHQ, 1 day | -0,17632 | 0,08607 | 0,38151 | 0,95599 | 0,05 | 0 | -0,58315 | 0,23052 |
| DHQ, 14 days  Control, 1 day | -0,04553 | 0,08333 | 0,02714 | 1 | 0,05 | 0 | -0,43945 | 0,34838 |
| DHQ, 14 days  Control, 7 days | -0,07952 | 0,08133 | 0,08692 | 0,99994 | 0,05 | 0 | -0,46394 | 0,3049 |
| DHQ, 14 days  Control, 14 days | -0,09799 | 0,07979 | 0,13713 | 0,99941 | 0,05 | 0 | -0,47514 | 0,27915 |
| DHQ, 14 days  Control, 28 days | -0,03501 | 0,08333 | 0,01604 | 1 | 0,05 | 0 | -0,42892 | 0,35891 |
| DHQ, 14 days  Control, 42 days | -0,06422 | 0,09623 | 0,04049 | 1 | 0,05 | 0 | -0,51907 | 0,39063 |
| DHQ, 14 days  Control, 56 days | -0,15524 | 0,09623 | 0,23661 | 0,9932 | 0,05 | 0 | -0,6101 | 0,29961 |
| DHQ, 14 days  DHQ, 1 day | -0,06972 | 0,08607 | 0,05965 | 0,99999 | 0,05 | 0 | -0,47655 | 0,33711 |
| DHQ, 14 days  DHQ, 7 days | 0,1066 | 0,09623 | 0,11156 | 0,99978 | 0,05 | 0 | -0,34826 | 0,56145 |
| DHQ, 28 days  Control, 1 day | 0,00516 | 0,08333 | 3,48582E-4 | 1 | 0,05 | 0 | -0,38875 | 0,39908 |
| DHQ, 28 days  Control, 7 days | -0,02883 | 0,08133 | 0,01142 | 1 | 0,05 | 0 | -0,41325 | 0,35559 |
| DHQ, 28 days  Control, 14 days | -0,0473 | 0,07979 | 0,03195 | 1 | 0,05 | 0 | -0,42444 | 0,32985 |
| DHQ, 28 days  Control, 28 days | 0,01569 | 0,08333 | 0,00322 | 1 | 0,05 | 0 | -0,37823 | 0,4096 |
| DHQ, 28 days  Control, 42 days | -0,01353 | 0,09623 | 0,0018 | 1 | 0,05 | 0 | -0,46838 | 0,44133 |
| DHQ, 28 days  Control, 56 days | -0,10455 | 0,09623 | 0,10731 | 0,99982 | 0,05 | 0 | -0,5594 | 0,35031 |
| DHQ, 28 days  DHQ, 1 day | -0,01902 | 0,08607 | 0,00444 | 1 | 0,05 | 0 | -0,42586 | 0,38781 |
| DHQ, 28 days  DHQ, 7 days | 0,15729 | 0,09623 | 0,2429 | 0,9924 | 0,05 | 0 | -0,29756 | 0,61214 |
| DHQ, 28 days  DHQ, 14 days | 0,05069 | 0,09623 | 0,02523 | 1 | 0,05 | 0 | -0,40416 | 0,50555 |
| DHQ, 42 days  Control, 1 day | -0,02298 | 0,08333 | 0,00691 | 1 | 0,05 | 0 | -0,4169 | 0,37093 |
| DHQ, 42 days  Control, 7 days | -0,05697 | 0,08133 | 0,04461 | 1 | 0,05 | 0 | -0,44139 | 0,32745 |
| DHQ, 42 days  Control, 14 days | -0,07544 | 0,07979 | 0,08127 | 0,99995 | 0,05 | 0 | -0,45259 | 0,3017 |
| DHQ, 42 days  Control, 28 days | -0,01245 | 0,08333 | 0,00203 | 1 | 0,05 | 0 | -0,40637 | 0,38146 |
| DHQ, 42 days  Control, 42 days | -0,04167 | 0,09623 | 0,01704 | 1 | 0,05 | 0 | -0,49652 | 0,41319 |
| DHQ, 42 days  Control, 56 days | -0,13269 | 0,09623 | 0,17285 | 0,99829 | 0,05 | 0 | -0,58754 | 0,32217 |
| DHQ, 42 days  DHQ, 1 day | -0,04717 | 0,08607 | 0,0273 | 1 | 0,05 | 0 | -0,454 | 0,35967 |
| DHQ, 42 days  DHQ, 7 days | 0,12915 | 0,09623 | 0,16376 | 0,99866 | 0,05 | 0 | -0,3257 | 0,584 |
| DHQ, 42 days  DHQ, 14 days | 0,02255 | 0,09623 | 0,00499 | 1 | 0,05 | 0 | -0,4323 | 0,47741 |
| DHQ, 42 days  DHQ, 28 days | -0,02814 | 0,09623 | 0,00777 | 1 | 0,05 | 0 | -0,483 | 0,42671 |
| DHQ, 56 days  Control, 1 day | 0,08619 | 0,08333 | 0,09725 | 0,99989 | 0,05 | 0 | -0,30772 | 0,48011 |
| DHQ, 56 days  Control, 7 days | 0,0522 | 0,08133 | 0,03746 | 1 | 0,05 | 0 | -0,33222 | 0,43662 |
| DHQ, 56 days  Control, 14 days | 0,03373 | 0,07979 | 0,01625 | 1 | 0,05 | 0 | -0,34341 | 0,41088 |
| DHQ, 56 days  Control, 28 days | 0,09672 | 0,08333 | 0,12246 | 0,99966 | 0,05 | 0 | -0,2972 | 0,49063 |
| DHQ, 56 days  Control, 42 days | 0,06751 | 0,09623 | 0,04474 | 1 | 0,05 | 0 | -0,38735 | 0,52236 |
| DHQ, 56 days  Control, 56 days | -0,02352 | 0,09623 | 0,00543 | 1 | 0,05 | 0 | -0,47837 | 0,43134 |
| DHQ, 56 days  DHQ, 1 day | 0,06201 | 0,08607 | 0,04719 | 1 | 0,05 | 0 | -0,34483 | 0,46884 |
| DHQ, 56 days  DHQ, 7 days | 0,23832 | 0,09623 | 0,55763 | 0,85108 | 0,05 | 0 | -0,21653 | 0,69318 |
| DHQ, 56 days  DHQ, 14 days | 0,13173 | 0,09623 | 0,17036 | 0,9984 | 0,05 | 0 | -0,32313 | 0,58658 |
| DHQ, 56 days  DHQ, 28 days | 0,08103 | 0,09623 | 0,06447 | 0,99999 | 0,05 | 0 | -0,37382 | 0,53589 |
| DHQ, 56 days  DHQ, 42 days | 0,10917 | 0,09623 | 0,11702 | 0,99973 | 0,05 | 0 | -0,34568 | 0,56403 |

#### Tukey Test

| | MeanDiff | SEM | q Value | Prob | Alpha | Sig | LCL | UCL |
|---|---|---|---|---|---|---|---|---|
| Control, 7 days  Control, 1 day | 0,03399 | 0,06557 | 0,7331 | 0,99999 | 0,05 | 0 | -0,19305 | 0,26103 |
| Control, 14 days  Control, 1 day | 0,05246 | 0,06365 | 1,16562 | 0,99945 | 0,05 | 0 | -0,16793 | 0,27285 |
| Control, 14 days  Control, 7 days | 0,01847 | 0,06099 | 0,42827 | 1 | 0,05 | 0 | -0,19273 | 0,22967 |
| Control, 28 days  Control, 1 day | -0,01053 | 0,06804 | 0,21881 | 1 | 0,05 | 0 | -0,24613 | 0,22508 |
| Control, 28 days  Control, 7 days | -0,04452 | 0,06557 | 0,96017 | 0,99991 | 0,05 | 0 | -0,27155 | 0,18252 |
| Control, 28 days  Control, 14 da | -0,06299 | 0,06365 | 1,39954 | 0,99713 | 0,05 | 0 | -0,28338 | 0,1574 |
| Control, 42 days  Control, 1 day | 0,01869 | 0,08333 | 0,31711 | 1 | 0,05 | 0 | -0,26987 | 0,30724 |
| Control, 42 days  Control, 7 days | -0,0153 | 0,08133 | 0,26611 | 1 | 0,05 | 0 | -0,29691 | 0,2663 |
| Control, 42 days  Control, 14 da | -0,03377 | 0,07979 | 0,59864 | 1 | 0,05 | 0 | -0,31005 | 0,2425 |
| Control, 42 days  Control, 28 da | 0,02921 | 0,08333 | 0,49577 | 1 | 0,05 | 0 | -0,25934 | 0,31777 |
| Control, 56 days  Control, 1 day | 0,10971 | 0,08333 | 1,86176 | 0,97205 | 0,05 | 0 | -0,17885 | 0,39827 |
| Control, 56 days  Control, 7 days | 0,07572 | 0,08133 | 1,3167 | 0,99832 | 0,05 | 0 | -0,20589 | 0,35732 |
| Control, 56 days  Control, 14 da | 0,05725 | 0,07979 | 1,0147 | 0,99985 | 0,05 | 0 | -0,21903 | 0,33352 |
| Control, 56 days  Control, 28 da | 0,12023 | 0,08333 | 2,04042 | 0,94729 | 0,05 | 0 | -0,16832 | 0,40879 |
| Control, 56 days  Control, 42 da | 0,09102 | 0,09623 | 1,33771 | 0,99807 | 0,05 | 0 | -0,24218 | 0,42422 |
| DHQ, 1 day  Control, 1 day | 0,02419 | 0,07136 | 0,47928 | 1 | 0,05 | 0 | -0,22292 | 0,27129 |
| DHQ, 1 day  Control, 7 days | -0,0098 | 0,06901 | 0,20091 | 1 | 0,05 | 0 | -0,24875 | 0,22915 |
| DHQ, 1 day  Control, 14 days | -0,02827 | 0,06719 | 0,59515 | 1 | 0,05 | 0 | -0,26092 | 0,20437 |
| DHQ, 1 day  Control, 28 days | 0,03471 | 0,07136 | 0,68791 | 1 | 0,05 | 0 | -0,21239 | 0,28182 |
| DHQ, 1 day  Control, 42 days | 0,0055 | 0,08607 | 0,09036 | 1 | 0,05 | 0 | -0,29252 | 0,30352 |
| DHQ, 1 day  Control, 56 days | -0,08552 | 0,08607 | 1,40525 | 0,99703 | 0,05 | 0 | -0,38354 | 0,2125 |
| DHQ, 7 days  Control, 1 day | -0,15213 | 0,08333 | 2,5817 | 0,79488 | 0,05 | 0 | -0,44069 | 0,13643 |
| DHQ, 7 days  Control, 7 days | -0,18612 | 0,08133 | 3,23649 | 0,50014 | 0,05 | 0 | -0,46772 | 0,09549 |
| DHQ, 7 days  Control, 14 days | -0,20459 | 0,07979 | 3,62634 | 0,33193 | 0,05 | 0 | -0,48086 | 0,07168 |
| DHQ, 7 days  Control, 28 days | -0,1416 | 0,08333 | 2,40304 | 0,85829 | 0,05 | 0 | -0,43016 | 0,14696 |
| DHQ, 7 days  Control, 42 days | -0,17082 | 0,09623 | 2,51044 | 0,82159 | 0,05 | 0 | -0,50401 | 0,16238 |
| DHQ, 7 days  Control, 56 days | -0,26184 | 0,09623 | 3,84815 | 0,25242 | 0,05 | 0 | -0,59504 | 0,07136 |
| DHQ, 7 days  DHQ, 1 day | -0,17632 | 0,08607 | 2,89711 | 0,65937 | 0,05 | 0 | -0,47434 | 0,12171 |
| DHQ, 14 days  Control, 1 day | -0,04553 | 0,08333 | 0,77273 | 0,99999 | 0,05 | 0 | -0,33409 | 0,24302 |
| DHQ, 14 days  Control, 7 days | -0,07952 | 0,08133 | 1,38286 | 0,99742 | 0,05 | 0 | -0,36113 | 0,20208 |
| DHQ, 14 days  Control, 14 days | -0,09799 | 0,07979 | 1,73694 | 0,98329 | 0,05 | 0 | -0,37427 | 0,17828 |
| DHQ, 14 days  Control, 28 days | -0,03501 | 0,08333 | 0,59407 | 1 | 0,05 | 0 | -0,32357 | 0,25355 |
| DHQ, 14 days  Control, 42 days | -0,06422 | 0,09623 | 0,94383 | 0,99993 | 0,05 | 0 | -0,39742 | 0,26898 |
| DHQ, 14 days  Control, 56 days | -0,15524 | 0,09623 | 2,28154 | 0,89423 | 0,05 | 0 | -0,48844 | 0,17796 |
| DHQ, 14 days  DHQ, 1 day | -0,06972 | 0,08607 | 1,14559 | 0,99953 | 0,05 | 0 | -0,36774 | 0,2283 |
| DHQ, 14 days  DHQ, 7 days | 0,1066 | 0,09623 | 1,56661 | 0,9926 | 0,05 | 0 | -0,2266 | 0,43979 |
| DHQ, 28 days  Control, 1 day | 0,00516 | 0,08333 | 0,08757 | 1 | 0,05 | 0 | -0,2834 | 0,29372 |
| DHQ, 28 days  Control, 7 days | -0,02883 | 0,08133 | 0,50131 | 1 | 0,05 | 0 | -0,31043 | 0,25278 |
| DHQ, 28 days  Control, 14 days | -0,0473 | 0,07979 | 0,83838 | 0,99998 | 0,05 | 0 | -0,32357 | 0,22897 |
| DHQ, 28 days  Control, 28 days | 0,01569 | 0,08333 | 0,26623 | 1 | 0,05 | 0 | -0,27287 | 0,30425 |
| DHQ, 28 days  Control, 42 days | -0,01353 | 0,09623 | 0,19878 | 1 | 0,05 | 0 | -0,34672 | 0,31967 |
| DHQ, 28 days  Control, 56 days | -0,10455 | 0,09623 | 1,53649 | 0,99369 | 0,05 | 0 | -0,43775 | 0,22865 |
| DHQ, 28 days  DHQ, 1 day | -0,01902 | 0,08607 | 0,31261 | 1 | 0,05 | 0 | -0,31705 | 0,279 |
| DHQ, 28 days  DHQ, 7 days | 0,15729 | 0,09623 | 2,31165 | 0,88589 | 0,05 | 0 | -0,17591 | 0,49049 |
| DHQ, 28 days  DHQ, 14 days | 0,05069 | 0,09623 | 0,74505 | 0,99999 | 0,05 | 0 | -0,2825 | 0,38389 |
| DHQ, 42 days  Control, 1 day | -0,02298 | 0,08333 | 0,38999 | 1 | 0,05 | 0 | -0,31154 | 0,26558 |
| DHQ, 42 days  Control, 7 days | -0,05697 | 0,08133 | 0,99066 | 0,99988 | 0,05 | 0 | -0,33857 | 0,22464 |
| DHQ, 42 days  Control, 14 days | -0,07544 | 0,07979 | 1,33717 | 0,99808 | 0,05 | 0 | -0,35171 | 0,20083 |
| DHQ, 42 days  Control, 28 days | -0,01245 | 0,08333 | 0,21133 | 1 | 0,05 | 0 | -0,30101 | 0,27611 |
| DHQ, 42 days  Control, 42 days | -0,04167 | 0,09623 | 0,61236 | 1 | 0,05 | 0 | -0,37487 | 0,29153 |
| DHQ, 42 days  Control, 56 days | -0,13269 | 0,09623 | 1,95007 | 0,96121 | 0,05 | 0 | -0,46589 | 0,20051 |
| DHQ, 42 days  DHQ, 1 day | -0,04717 | 0,08607 | 0,775 | 0,99999 | 0,05 | 0 | -0,34519 | 0,25086 |
| DHQ, 42 days  DHQ, 7 days | 0,12915 | 0,09623 | 1,89807 | 0,96791 | 0,05 | 0 | -0,20405 | 0,46235 |
| DHQ, 42 days  DHQ, 14 days | 0,02255 | 0,09623 | 0,33147 | 1 | 0,05 | 0 | -0,31065 | 0,35575 |
| DHQ, 42 days  DHQ, 28 days | -0,02814 | 0,09623 | 0,41358 | 1 | 0,05 | 0 | -0,36134 | 0,30506 |
| DHQ, 56 days  Control, 1 day | 0,08619 | 0,08333 | 1,4627 | 0,99582 | 0,05 | 0 | -0,20237 | 0,37475 |
| DHQ, 56 days  Control, 7 days | 0,0522 | 0,08133 | 0,90778 | 0,99995 | 0,05 | 0 | -0,2294 | 0,33381 |
| DHQ, 56 days  Control, 14 days | 0,03373 | 0,07979 | 0,5979 | 1 | 0,05 | 0 | -0,24254 | 0,31001 |
| DHQ, 56 days  Control, 28 days | 0,09672 | 0,08333 | 1,64136 | 0,98924 | 0,05 | 0 | -0,19184 | 0,38528 |
| DHQ, 56 days  Control, 42 days | 0,06751 | 0,09623 | 0,99212 | 0,99988 | 0,05 | 0 | -0,26569 | 0,4007 |
| DHQ, 56 days  Control, 56 days | -0,02352 | 0,09623 | 0,34559 | 1 | 0,05 | 0 | -0,35671 | 0,30968 |
| DHQ, 56 days  DHQ, 1 day | 0,06201 | 0,08607 | 1,01886 | 0,99985 | 0,05 | 0 | -0,23602 | 0,36003 |
| DHQ, 56 days  DHQ, 7 days | 0,23832 | 0,09623 | 3,50255 | 0,38196 | 0,05 | 0 | -0,09488 | 0,57152 |
| DHQ, 56 days  DHQ, 14 days | 0,13173 | 0,09623 | 1,93594 | 0,96312 | 0,05 | 0 | -0,20147 | 0,46493 |
| DHQ, 56 days  DHQ, 28 days | 0,08103 | 0,09623 | 1,1909 | 0,99933 | 0,05 | 0 | -0,25217 | 0,41423 |
| DHQ, 56 days  DHQ, 42 days | 0,10917 | 0,09623 | 1,60448 | 0,99102 | 0,05 | 0 | -0,22403 | 0,44237 |

### Grouping Letters

#### Bonferroni Test

| | Mean | Groups |
|---|---|---|
| Control, 56 days | 0,30514 | A |
| DHQ, 56 days | 0,28163 | A |
| Control, 14 days | 0,24789 | A |
| Control, 7 days | 0,22942 | A |
| DHQ, 1 day | 0,21962 | A |
| Control, 42 days | 0,21412 | A |
| DHQ, 28 days | 0,2006 | A |
| Control, 1 day | 0,19544 | A |
| Control, 28 days | 0,18491 | A |
| DHQ, 42 days | 0,17245 | A |
| DHQ, 14 days | 0,1499 | A |
| DHQ, 7 days | 0,04331 | A |

Means that do not share a letter are significantly different.

#### Scheffe Test

| | Mean | Groups |
|---|---|---|
| Control, 56 days | 0,30514 | A |
| DHQ, 56 days | 0,28163 | A |
| Control, 14 days | 0,24789 | A |
| Control, 7 days | 0,22942 | A |
| DHQ, 1 day | 0,21962 | A |
| Control, 42 days | 0,21412 | A |
| DHQ, 28 days | 0,2006 | A |
| Control, 1 day | 0,19544 | A |
| Control, 28 days | 0,18491 | A |
| DHQ, 42 days | 0,17245 | A |
| DHQ, 14 days | 0,1499 | A |
| DHQ, 7 days | 0,04331 | A |

Means that do not share a letter are significantly different.

#### Tukey Test

| | Mean | Groups |
|---|---|---|
| Control, 56 days | 0,30514 | A |
| DHQ, 56 days | 0,28163 | A |
| Control, 14 days | 0,24789 | A |
| Control, 7 days | 0,22942 | A |
| DHQ, 1 day | 0,21962 | A |
| Control, 42 days | 0,21412 | A |
| DHQ, 28 days | 0,2006 | A |
| Control, 1 day | 0,19544 | A |
| Control, 28 days | 0,18491 | A |
| DHQ, 42 days | 0,17245 | A |
| DHQ, 14 days | 0,1499 | A |
| DHQ, 7 days | 0,04331 | A |

Means that do not share a letter are significantly different.

Sig equals 1 indicates that the difference of the means is significant at the 0,05 level.
Sig equals 0 indicates that the difference of the means is not significant at the 0,05 level.

## Powers

| | Alpha | Sample Size | Power |
|---|---|---|---|
| Actual Power | 0,05 | 53 | 0,48628 |

*ANOVAOneWay (27.10.2024 21:52:28)*

## Notes

| | |
|---|---|
| Description | Perform One-Way ANOVA |
| User Name | yulia |
| Operation Time | 27.10.2024 21:52:28 |
| Report Status | New Analysis Report |
| Data Filter | No |

## Input Data

| | Data | Range |
|---|---|---|
| Control, 1 day | [Book1]"FSC Cytometry"!A"Control, 1 day" | [1*:4*] |
| Control, 7 days | [Book1]"FSC Cytometry"!B"Control, 7 days" | [1*:4*] |
| Control, 14 days | [Book1]"FSC Cytometry"!C"Control, 14 days" | [1*:4*] |
| Control, 28 days | [Book1]"FSC Cytometry"!D"Control, 28 days" | [1*:5*] |
| Control, 42 days | [Book1]"FSC Cytometry"!E"Control, 42 days" | [1*:3*] |
| Control, 56 days | [Book1]"FSC Cytometry"!F"Control, 56 days" | [1*:3*] |
| DHQ, 1 day | [Book1]"FSC Cytometry"!G"DHQ, 1 day" | [1*:3*] |
| DHQ, 7 days | [Book1]"FSC Cytometry"!H"DHQ, 7 days" | [1*:3*] |
| DHQ, 14 days | [Book1]"FSC Cytometry"!I"DHQ, 14 days" | [1*:3*] |
| DHQ, 28 days | [Book1]"FSC Cytometry"!J"DHQ, 28 days" | [1*:4*] |
| DHQ, 42 days | [Book1]"FSC Cytometry"!K"DHQ, 42 days" | [1*:3*] |
| DHQ, 56 days | [Book1]"FSC Cytometry"!L"DHQ, 56 days" | [1*:3*] |

## Descriptive Statistics

| | N Analysis | N Missing | Mean | Standard Deviation | SE of Mean |
|---|---|---|---|---|---|
| Control, 1 day | 4 | 0 | 187637,09167 | 20300,18446 | 10150,09223 |
| Control, 7 days | 4 | 0 | 172434,83333 | 25960,61105 | 12980,30552 |
| Control, 14 days | 4 | 0 | 163197,58333 | 19652,20019 | 9826,10009 |
| Control, 28 days | 5 | 0 | 142952,15333 | 20997,12123 | 9390,19808 |
| Control, 42 days | 3 | 0 | 157114,37778 | 18696,14215 | 10794,2227 |
| Control, 56 days | 3 | 0 | 142232,52222 | 22241,02555 | 12840,86209 |
| DHQ, 1 day | 3 | 0 | 189120,6 | 26194,42874 | 15123,36049 |
| DHQ, 7 days | 3 | 0 | 165571,76667 | 20162,09376 | 11640,59026 |
| DHQ, 14 days | 3 | 0 | 153796,93333 | 21963,23243 | 12680,47816 |
| DHQ, 28 days | 4 | 0 | 143657,525 | 22392,90221 | 11196,45111 |
| DHQ, 42 days | 3 | 0 | 162224,5 | 14680,4 | 8475,73289 |
| DHQ, 56 days | 3 | 0 | 153104,95 | 19569,25 | 11298,31176 |

## ANOVA

### Overall ANOVA

| | DF | Sum of Squares | Mean Square | F Value | Prob>F |
|---|---|---|---|---|---|
| Model | 11 | 1,00939E10 | 9,17623E8 | 2,00646 | 0,06418 |
| Error | 30 | 1,37201E10 | 4,57335E8 | | |
| Total | 41 | 2,38139E10 | | | |

Null Hypothesis: The means of all levels are equal.
Alternative Hypothesis: The means of one or more levels are different.
At the 0.05 level, the population means are not significantly different.

### Fit Statistics

| | R-Square | Coeff Var | Root MSE | Data Mean |
|---|---|---|---|---|
| | 0,42386 | 0,13303 | 21385,40207 | 160761,09167 |

### Means Comparisons

#### Bonferroni Test

| | MeanDiff | SEM | t Value | Prob | Alpha | Sig | LCL | UCL |
|---|---|---|---|---|---|---|---|---|
| Control, 7 days Control, 1 day | -15202,25833 | 15121,76282 | -1,00532 | 1 | 0,05 | 0 | -71892,26404 | 41487,74737 |
| Control, 14 days Control, 1 day | -24439,50833 | 15121,76282 | -1,61618 | 1 | 0,05 | 0 | -81129,51404 | 32250,49737 |
| Control, 14 days Control, 7 days | -9237,25 | 15121,76282 | -0,61086 | 1 | 0,05 | 0 | -65927,2557 | 47452,7557 |
| Control, 28 days Control, 1 day | -44684,93833 | 14345,76382 | -3,11485 | 0,26591 | 0,05 | 0 | -98465,79991 | 9095,92324 |
| Control, 28 days Control, 7 days | -29482,68 | 14345,76382 | -2,05515 | 1 | 0,05 | 0 | -83263,54158 | 24298,18158 |
| Control, 28 days Control, 14 da | -20245,43 | 14345,76382 | -1,41125 | 1 | 0,05 | 0 | -74026,29158 | 33535,43158 |
| Control, 42 days Control, 1 day | -30522,71389 | 16333,37062 | -1,86873 | 1 | 0,05 | 0 | -91754,91841 | 30709,49064 |
| Control, 42 days Control, 7 days | -15320,45556 | 16333,37062 | -0,93798 | 1 | 0,05 | 0 | -76552,66008 | 45911,74897 |
| Control, 42 days Control, 14 da | -6083,20556 | 16333,37062 | -0,37244 | 1 | 0,05 | 0 | -67315,41008 | 55148,99897 |
| Control, 42 days Control, 28 da | 14162,22444 | 15617,68949 | 0,90681 | 1 | 0,05 | 0 | -44386,96168 | 72711,41057 |
| Control, 56 days Control, 1 day | -45404,56944 | 16333,37062 | -2,77987 | 0,61375 | 0,05 | 0 | -106636,7739 | 15827,63508 |
| Control, 56 days Control, 7 days | -30202,31111 | 16333,37062 | -1,84912 | 1 | 0,05 | 0 | -91434,51564 | 31029,89341 |
| Control, 56 days Control, 14 da | -20965,06111 | 16333,37062 | -1,28357 | 1 | 0,05 | 0 | -82197,26564 | 40267,14341 |
| Control, 56 days Control, 28 da | -719,63111 | 15617,68949 | -0,04608 | 1 | 0,05 | 0 | -59268,81724 | 57829,55502 |
| Control, 56 days Control, 42 da | -14881,85556 | 17461,10767 | -0,85229 | 1 | 0,05 | 0 | -80341,83566 | 50578,12455 |
| DHQ, 1 day Control, 1 day | 1483,50833 | 16333,37062 | 0,09083 | 1 | 0,05 | 0 | -59748,69619 | 62715,71286 |
| DHQ, 1 day Control, 7 days | 16685,76667 | 16333,37062 | 1,02158 | 1 | 0,05 | 0 | -44546,43786 | 77917,97119 |
| DHQ, 1 day Control, 14 days | 25923,01667 | 16333,37062 | 1,58712 | 1 | 0,05 | 0 | -35309,18786 | 87155,22119 |
| DHQ, 1 day Control, 28 days | 46168,44667 | 15617,68949 | 2,95616 | 0,39708 | 0,05 | 0 | -12380,73946 | 104717,63279 |
| DHQ, 1 day Control, 42 days | 32006,22222 | 17461,10767 | 1,833 | 1 | 0,05 | 0 | -33453,75788 | 97466,20233 |
| DHQ, 1 day Control, 56 days | 46888,07778 | 17461,10767 | 2,68529 | 0,77159 | 0,05 | 0 | -18571,90233 | 112348,05788 |
| DHQ, 7 days Control, 1 day | -22065,325 | 16333,37062 | -1,35094 | 1 | 0,05 | 0 | -83297,52952 | 39166,87952 |
| DHQ, 7 days Control, 7 days | -6863,06667 | 16333,37062 | -0,42019 | 1 | 0,05 | 0 | -68095,27119 | 54369,13786 |
| DHQ, 7 days Control, 14 days | 2374,18333 | 16333,37062 | 0,14536 | 1 | 0,05 | 0 | -58858,02119 | 63606,38786 |
| DHQ, 7 days Control, 28 days | 22619,61333 | 15617,68949 | 1,44833 | 1 | 0,05 | 0 | -35929,57279 | 81168,79946 |
| DHQ, 7 days Control, 42 days | 8457,38889 | 17461,10767 | 0,48436 | 1 | 0,05 | 0 | -57002,59122 | 73917,36899 |
| DHQ, 7 days Control, 56 days | 23339,24444 | 17461,10767 | 1,33664 | 1 | 0,05 | 0 | -42120,73566 | 88799,22455 |
| DHQ, 7 days DHQ, 1 day | -23548,83333 | 17461,10767 | -1,34864 | 1 | 0,05 | 0 | -89008,81344 | 41911,14677 |
| DHQ, 14 days Control, 1 day | -33840,15833 | 16333,37062 | -2,07184 | 1 | 0,05 | 0 | -95072,36286 | 27392,04619 |
| DHQ, 14 days Control, 7 days | -18637,9 | 16333,37062 | -1,14109 | 1 | 0,05 | 0 | -79870,10452 | 42594,30452 |
| DHQ, 14 days Control, 14 days | -9400,65 | 16333,37062 | -0,57555 | 1 | 0,05 | 0 | -70632,85452 | 51831,55452 |
| DHQ, 14 days Control, 28 days | 10844,78 | 15617,68949 | 0,69439 | 1 | 0,05 | 0 | -47704,40613 | 69393,96613 |
| DHQ, 14 days Control, 42 days | -3317,44444 | 17461,10767 | -0,18999 | 1 | 0,05 | 0 | -68777,42455 | 62142,53566 |
| DHQ, 14 days Control, 56 days | 11564,41111 | 17461,10767 | 0,6623 | 1 | 0,05 | 0 | -53895,56899 | 77024,39122 |
| DHQ, 14 days DHQ, 1 day | -35323,66667 | 17461,10767 | -2,02299 | 1 | 0,05 | 0 | -100783,6467 | 30136,31344 |
| DHQ, 14 days DHQ, 7 days | -11774,83333 | 17461,10767 | -0,67435 | 1 | 0,05 | 0 | -77234,81344 | 53685,14677 |
| DHQ, 28 days Control, 1 day | -43979,56667 | 15121,76282 | -2,90836 | 0,44733 | 0,05 | 0 | -100669,5723 | 12710,43904 |
| DHQ, 28 days Control, 7 days | -28777,30833 | 15121,76282 | -1,90304 | 1 | 0,05 | 0 | -85467,31404 | 27912,69737 |
| DHQ, 28 days Control, 14 days | -19540,05833 | 15121,76282 | -1,29218 | 1 | 0,05 | 0 | -76230,06404 | 37149,94737 |
| DHQ, 28 days Control, 28 days | 705,37167 | 14345,76382 | 0,04917 | 1 | 0,05 | 0 | -53075,48991 | 54486,23324 |
| DHQ, 28 days Control, 42 days | -13456,85278 | 16333,37062 | -0,82389 | 1 | 0,05 | 0 | -74689,0573 | 47775,35175 |
| DHQ, 28 days Control, 56 days | 1425,00278 | 16333,37062 | 0,08724 | 1 | 0,05 | 0 | -59807,20175 | 62657,2073 |
| DHQ, 28 days DHQ, 1 day | -45463,075 | 16333,37062 | -2,78345 | 0,60841 | 0,05 | 0 | -106695,2795 | 15769,12952 |
| DHQ, 28 days DHQ, 7 days | -21914,24167 | 16333,37062 | -1,34169 | 1 | 0,05 | 0 | -83146,44619 | 39317,96286 |
| DHQ, 28 days DHQ, 14 days | -10139,40833 | 16333,37062 | -0,62078 | 1 | 0,05 | 0 | -71371,61286 | 51092,79619 |
| DHQ, 42 days Control, 1 day | -25412,59167 | 16333,37062 | -1,55587 | 1 | 0,05 | 0 | -86644,79619 | 35819,61286 |
| DHQ, 42 days Control, 7 days | -10210,33333 | 16333,37062 | -0,62512 | 1 | 0,05 | 0 | -71442,53786 | 51021,87119 |
| DHQ, 42 days Control, 14 days | -973,08333 | 16333,37062 | -0,05958 | 1 | 0,05 | 0 | -62205,28786 | 60259,12119 |
| DHQ, 42 days Control, 28 days | 19272,34667 | 15617,68949 | 1,23401 | 1 | 0,05 | 0 | -39276,83946 | 77821,53279 |
| DHQ, 42 days Control, 42 days | 5110,12222 | 17461,10767 | 0,29266 | 1 | 0,05 | 0 | -60349,85788 | 70570,10233 |
| DHQ, 42 days Control, 56 days | 19991,97778 | 17461,10767 | 1,14494 | 1 | 0,05 | 0 | -45468,00233 | 85451,95788 |
| DHQ, 42 days DHQ, 1 day | -26896,1 | 17461,10767 | -1,54034 | 1 | 0,05 | 0 | -92356,0801 | 38563,8801 |
| DHQ, 42 days DHQ, 7 days | -3347,26667 | 17461,10767 | -0,1917 | 1 | 0,05 | 0 | -68807,24677 | 62112,71344 |
| DHQ, 42 days DHQ, 14 days | 8427,56667 | 17461,10767 | 0,48265 | 1 | 0,05 | 0 | -57032,41344 | 73887,54677 |
| DHQ, 42 days DHQ, 28 days | 18566,975 | 16333,37062 | 1,13675 | 1 | 0,05 | 0 | -42665,22952 | 79799,17952 |
| DHQ, 56 days Control, 1 day | -34532,14167 | 16333,37062 | -2,11421 | 1 | 0,05 | 0 | -95764,34619 | 26700,06286 |
| DHQ, 56 days Control, 7 days | -19329,88333 | 16333,37062 | -1,18346 | 1 | 0,05 | 0 | -80562,08786 | 41902,32119 |
| DHQ, 56 days Control, 14 days | -10092,63333 | 16333,37062 | -0,61791 | 1 | 0,05 | 0 | -71324,83786 | 51139,57119 |
| DHQ, 56 days Control, 28 days | 10152,79667 | 15617,68949 | 0,65008 | 1 | 0,05 | 0 | -48396,38946 | 68701,98279 |
| DHQ, 56 days Control, 42 days | -4009,42778 | 17461,10767 | -0,22962 | 1 | 0,05 | 0 | -69469,40788 | 61450,55233 |
| DHQ, 56 days Control, 56 days | 10872,42778 | 17461,10767 | 0,62267 | 1 | 0,05 | 0 | -54587,55233 | 76332,40788 |
| DHQ, 56 days DHQ, 1 day | -36015,65 | 17461,10767 | -2,06262 | 1 | 0,05 | 0 | -101475,6301 | 29444,3301 |
| DHQ, 56 days DHQ, 7 days | -12466,81667 | 17461,10767 | -0,71398 | 1 | 0,05 | 0 | -77926,79677 | 52993,16344 |
| DHQ, 56 days DHQ, 14 days | -691,98333 | 17461,10767 | -0,03963 | 1 | 0,05 | 0 | -66151,96344 | 64767,99677 |
| DHQ, 56 days DHQ, 28 days | 9447,425 | 16333,37062 | 0,57841 | 1 | 0,05 | 0 | -51784,77952 | 70679,62952 |
| DHQ, 56 days DHQ, 42 days | -9119,55 | 17461,10767 | -0,52228 | 1 | 0,05 | 0 | -74579,5301 | 56340,4301 |

#### Scheffe Test

| | MeanDiff | SEM | F Value | Prob | Alpha | Sig | LCL | UCL |
|---|---|---|---|---|---|---|---|---|
| Control, 7 days Control, 1 day | -15202,25833 | 15121,76282 | 0,09188 | 0,9999 | 0,05 | 0 | -88322,11348 | 57917,59681 |
| Control, 14 days Control, 1 day | -24439,50833 | 15121,76282 | 0,23746 | 0,99238 | 0,05 | 0 | -97559,36348 | 48680,34681 |
| Control, 14 days Control, 7 days | -9237,25 | 15121,76282 | 0,03392 | 1 | 0,05 | 0 | -82357,10514 | 63882,60514 |
| Control, 28 days Control, 1 day | -44684,93833 | 14345,76382 | 0,88203 | 0,56686 | 0,05 | 0 | -114052,5236 | 24682,647 |
| Control, 28 days Control, 7 days | -29482,68 | 14345,76382 | 0,38397 | 0,95221 | 0,05 | 0 | -98850,26533 | 39884,90533 |
| Control, 28 days Control, 14 da | -20245,43 | 14345,76382 | 0,18106 | 0,99763 | 0,05 | 0 | -89613,01533 | 49122,15533 |
| Control, 42 days Control, 1 day | -30522,71389 | 16333,37062 | 0,31747 | 0,97595 | 0,05 | 0 | -109501,1840 | 48455,75629 |
| Control, 42 days Control, 7 days | -15320,45556 | 16333,37062 | 0,07998 | 0,99995 | 0,05 | 0 | -94298,92574 | 63658,01463 |
| Control, 42 days Control, 14 da | -6083,20556 | 16333,37062 | 0,01261 | 1 | 0,05 | 0 | -85061,67574 | 72895,26463 |
| Control, 42 days Control, 28 da | 14162,22444 | 15617,68949 | 0,07475 | 0,99997 | 0,05 | 0 | -61355,63722 | 89680,08611 |
| Control, 56 days Control, 1 day | -45404,56944 | 16333,37062 | 0,70251 | 0,72623 | 0,05 | 0 | -124383,0396 | 33573,90074 |
| Control, 56 days Control, 7 days | -30202,31111 | 16333,37062 | 0,31084 | 0,97779 | 0,05 | 0 | -109180,7812 | 48776,15907 |
| Control, 56 days Control, 14 da | -20965,06111 | 16333,37062 | 0,14978 | 0,99899 | 0,05 | 0 | -99943,53129 | 58013,40907 |
| Control, 56 days Control, 28 da | -719,63111 | 15617,68949 | 1,93016E-4 | 1 | 0,05 | 0 | -76237,49278 | 74798,23055 |
| Control, 56 days Control, 42 da | -14881,85556 | 17461,10767 | 0,06604 | 0,99998 | 0,05 | 0 | -99313,39166 | 69549,68054 |
| DHQ, 1 day Control, 1 day | 1483,50833 | 16333,37062 | 7,49956E-4 | 1 | 0,05 | 0 | -77494,96185 | 80461,97852 |
| DHQ, 1 day Control, 7 days | 16685,76667 | 16333,37062 | 0,09487 | 0,99989 | 0,05 | 0 | -62292,70352 | 95664,23685 |
| DHQ, 1 day Control, 14 days | 25923,01667 | 16333,37062 | 0,229 | 0,99345 | 0,05 | 0 | -53055,45352 | 104901,48685 |
| DHQ, 1 day Control, 28 days | 46168,44667 | 15617,68949 | 0,79445 | 0,64412 | 0,05 | 0 | -29349,415 | 121686,30833 |
| DHQ, 1 day Control, 42 days | 32006,22222 | 17461,10767 | 0,30544 | 0,97922 | 0,05 | 0 | -52425,31388 | 116437,75832 |
| DHQ, 1 day Control, 56 days | 46888,07778 | 17461,10767 | 0,65552 | 0,76724 | 0,05 | 0 | -37543,45832 | 131319,61388 |
| DHQ, 7 days Control, 1 day | -22065,325 | 16333,37062 | 0,16591 | 0,9984 | 0,05 | 0 | -101043,7951 | 56913,14518 |
| DHQ, 7 days Control, 7 days | -6863,06667 | 16333,37062 | 0,01605 | 1 | 0,05 | 0 | -85841,53685 | 72115,40352 |
| DHQ, 7 days Control, 14 days | 2374,18333 | 16333,37062 | 0,00192 | 1 | 0,05 | 0 | -76604,28685 | 81352,65352 |
| DHQ, 7 days Control, 28 days | 22619,61333 | 15617,68949 | 0,1907 | 0,99702 | 0,05 | 0 | -52898,24833 | 98137,475 |
| DHQ, 7 days Control, 42 days | 8457,38889 | 17461,10767 | 0,02133 | 1 | 0,05 | 0 | -75974,14721 | 92888,92499 |
| DHQ, 7 days Control, 56 days | 23339,24444 | 17461,10767 | 0,16242 | 0,99854 | 0,05 | 0 | -61092,29166 | 107770,78054 |
| DHQ, 7 days DHQ, 1 day | -23548,83333 | 17461,10767 | 0,16535 | 0,99842 | 0,05 | 0 | -107980,3694 | 60882,70277 |
| DHQ, 14 days Control, 1 day | -33840,15833 | 16333,37062 | 0,39023 | 0,94947 | 0,05 | 0 | -112818,6285 | 45138,31185 |
| DHQ, 14 days Control, 7 days | -18637,9 | 16333,37062 | 0,11837 | 0,99967 | 0,05 | 0 | -97616,37018 | 60340,57018 |
| DHQ, 14 days Control, 14 days | -9400,65 | 16333,37062 | 0,03011 | 1 | 0,05 | 0 | -88379,12018 | 69577,82018 |
| DHQ, 14 days Control, 28 days | 10844,78 | 15617,68949 | 0,04383 | 1 | 0,05 | 0 | -64673,08167 | 86362,64167 |
| DHQ, 14 days Control, 42 days | -3317,44444 | 17461,10767 | 0,00328 | 1 | 0,05 | 0 | -87748,98054 | 81114,09166 |
| DHQ, 14 days Control, 56 days | 11564,41111 | 17461,10767 | 0,03988 | 1 | 0,05 | 0 | -72867,12499 | 95995,94721 |
| DHQ, 14 days DHQ, 1 day | -35323,66667 | 17461,10767 | 0,37204 | 0,95718 | 0,05 | 0 | -119755,2027 | 49107,86943 |
| DHQ, 14 days DHQ, 7 days | -11774,83333 | 17461,10767 | 0,04134 | 1 | 0,05 | 0 | -96206,36943 | 72656,70277 |
| DHQ, 28 days Control, 1 day | -43979,56667 | 15121,76282 | 0,76896 | 0,66694 | 0,05 | 0 | -117099,4218 | 29140,28848 |
| DHQ, 28 days Control, 7 days | -28777,30833 | 15121,76282 | 0,32923 | 0,97245 | 0,05 | 0 | -101897,1634 | 44342,54681 |
| DHQ, 28 days Control, 14 days | -19540,05833 | 15121,76282 | 0,15179 | 0,99893 | 0,05 | 0 | -92659,91348 | 53579,79681 |
| DHQ, 28 days Control, 28 days | 705,37167 | 14345,76382 | 2,19784E-4 | 1 | 0,05 | 0 | -68662,21366 | 70072,957 |
| DHQ, 28 days Control, 42 days | -13456,85278 | 16333,37062 | 0,06171 | 0,99999 | 0,05 | 0 | -92435,32296 | 65521,6174 |
| DHQ, 28 days Control, 56 days | 1425,00278 | 16333,37062 | 6,9197E-4 | 1 | 0,05 | 0 | -77553,4674 | 80403,47296 |
| DHQ, 28 days DHQ, 1 day | -45463,075 | 16333,37062 | 0,70433 | 0,72463 | 0,05 | 0 | -124441,5451 | 33515,39518 |
| DHQ, 28 days DHQ, 7 days | -21914,24167 | 16333,37062 | 0,16365 | 0,99849 | 0,05 | 0 | -100892,7118 | 57064,22852 |
| DHQ, 28 days DHQ, 14 days | -10139,40833 | 16333,37062 | 0,03503 | 1 | 0,05 | 0 | -89117,87852 | 68839,06185 |
| DHQ, 42 days Control, 1 day | -25412,59167 | 16333,37062 | 0,22007 | 0,99446 | 0,05 | 0 | -104391,0618 | 53565,87852 |
| DHQ, 42 days Control, 7 days | -10210,33333 | 16333,37062 | 0,03553 | 1 | 0,05 | 0 | -89188,80352 | 68768,13685 |
| DHQ, 42 days Control, 14 days | -973,08333 | 16333,37062 | 3,22668E-4 | 1 | 0,05 | 0 | -79951,55352 | 78005,38685 |
| DHQ, 42 days Control, 28 days | 19272,34667 | 15617,68949 | 0,13843 | 0,9993 | 0,05 | 0 | -56245,515 | 94790,20833 |
| DHQ, 42 days Control, 42 days | 5110,12222 | 17461,10767 | 0,00779 | 1 | 0,05 | 0 | -79321,41388 | 89541,65832 |
| DHQ, 42 days Control, 56 days | 19991,97778 | 17461,10767 | 0,11917 | 0,99966 | 0,05 | 0 | -64439,55832 | 104423,51388 |
| DHQ, 42 days DHQ, 1 day | -26896,1 | 17461,10767 | 0,2157 | 0,99492 | 0,05 | 0 | -111327,6361 | 57535,4361 |
| DHQ, 42 days DHQ, 7 days | -3347,26667 | 17461,10767 | 0,00334 | 1 | 0,05 | 0 | -87778,80277 | 81084,26943 |
| DHQ, 42 days DHQ, 14 days | 8427,56667 | 17461,10767 | 0,02118 | 1 | 0,05 | 0 | -76003,96943 | 92859,10277 |
| DHQ, 42 days DHQ, 28 days | 18566,975 | 16333,37062 | 0,11747 | 0,99968 | 0,05 | 0 | -60411,49518 | 97545,44518 |
| DHQ, 56 days Control, 1 day | -34532,14167 | 16333,37062 | 0,40635 | 0,94202 | 0,05 | 0 | -113510,6118 | 44446,32852 |
| DHQ, 56 days Control, 7 days | -19329,88333 | 16333,37062 | 0,12733 | 0,99953 | 0,05 | 0 | -98308,35352 | 59648,58685 |
| DHQ, 56 days Control, 14 days | -10092,63333 | 16333,37062 | 0,03471 | 1 | 0,05 | 0 | -89071,10352 | 68885,83685 |
| DHQ, 56 days Control, 28 days | 10152,79667 | 15617,68949 | 0,03842 | 1 | 0,05 | 0 | -65365,065 | 85670,65833 |
| DHQ, 56 days Control, 42 days | -4009,42778 | 17461,10767 | 0,00479 | 1 | 0,05 | 0 | -88440,96388 | 80422,10832 |
| DHQ, 56 days Control, 56 days | 10872,42778 | 17461,10767 | 0,03525 | 1 | 0,05 | 0 | -73559,10832 | 95303,96388 |
| DHQ, 56 days DHQ, 1 day | -36015,65 | 17461,10767 | 0,38676 | 0,951 | 0,05 | 0 | -120447,1861 | 48415,8861 |
| DHQ, 56 days DHQ, 7 days | -12466,81667 | 17461,10767 | 0,04634 | 1 | 0,05 | 0 | -96898,35277 | 71964,71943 |
| DHQ, 56 days DHQ, 14 days | -691,98333 | 17461,10767 | 1,42776E-4 | 1 | 0,05 | 0 | -85123,51943 | 83739,55277 |
| DHQ, 56 days DHQ, 28 days | 9447,425 | 16333,37062 | 0,03041 | 1 | 0,05 | 0 | -69531,04518 | 88425,89518 |
| DHQ, 56 days DHQ, 42 days | -9119,55 | 17461,10767 | 0,0248 | 1 | 0,05 | 0 | -93551,0861 | 75311,9861 |

#### Tukey Test

| | MeanDiff | SEM | q Value | Prob | Alpha | Sig | LCL | UCL |
|---|---|---|---|---|---|---|---|---|
| Control, 7 days Control, 1 day | -15202,25833 | 15121,76282 | 1,42174 | 0,99636 | 0,05 | 0 | -68674,41212 | 38269,89546 |
| Control, 14 days Control, 1 day | -24439,50833 | 15121,76282 | 2,28563 | 0,8897 | 0,05 | 0 | -77911,66212 | 29032,64546 |
| Control, 14 days Control, 7 days | -9237,25 | 15121,76282 | 0,86388 | 0,99996 | 0,05 | 0 | -62709,40379 | 44234,90379 |
| Control, 28 days Control, 1 day | -44684,93833 | 14345,76382 | 4,40507 | 0,12541 | 0,05 | 0 | -95413,07755 | 6043,20088 |
| Control, 28 days Control, 7 days | -29482,68 | 14345,76382 | 2,90642 | 0,65522 | 0,05 | 0 | -80210,81921 | 21245,45921 |
| Control, 28 days Control, 14 da | -20245,43 | 14345,76382 | 1,99581 | 0,95208 | 0,05 | 0 | -70973,56921 | 30482,70921 |
| Control, 42 days Control, 1 day | -30522,71389 | 16333,37062 | 2,64279 | 0,76815 | 0,05 | 0 | -88279,24111 | 27233,81333 |
| Control, 42 days Control, 7 days | -15320,45556 | 16333,37062 | 1,32651 | 0,998 | 0,05 | 0 | -73076,98277 | 42436,07166 |
| Control, 42 days Control, 14 da | -6083,20556 | 16333,37062 | 0,52671 | 1 | 0,05 | 0 | -63839,73277 | 51673,32166 |
| Control, 42 days Control, 28 da | 14162,22444 | 15617,68949 | 1,28242 | 0,99851 | 0,05 | 0 | -41063,57852 | 69388,02741 |
| Control, 56 days Control, 1 day | -45404,56944 | 16333,37062 | 3,93132 | 0,23834 | 0,05 | 0 | -103161,0966 | 12351,95777 |
| Control, 56 days Control, 7 days | -30202,31111 | 16333,37062 | 2,61505 | 0,77912 | 0,05 | 0 | -87958,83833 | 27554,21611 |
| Control, 56 days Control, 14 da | -20965,06111 | 16333,37062 | 1,81525 | 0,97513 | 0,05 | 0 | -78721,58833 | 36791,46611 |
| Control, 56 days Control, 28 da | -719,63111 | 15617,68949 | 0,06516 | 1 | 0,05 | 0 | -55945,43408 | 54506,17185 |
| Control, 56 days Control, 42 da | -14881,85556 | 17461,10767 | 1,20531 | 0,99915 | 0,05 | 0 | -76626,18033 | 46862,46922 |
| DHQ, 1 day Control, 1 day | 1483,50833 | 16333,37062 | 0,12845 | 1 | 0,05 | 0 | -56273,01888 | 59240,03555 |
| DHQ, 1 day Control, 7 days | 16685,76667 | 16333,37062 | 1,44473 | 0,99584 | 0,05 | 0 | -41070,76055 | 74442,29388 |
| DHQ, 1 day Control, 14 days | 25923,01667 | 16333,37062 | 2,24453 | 0,9006 | 0,05 | 0 | -31833,51055 | 83679,54388 |
| DHQ, 1 day Control, 28 days | 46168,44667 | 15617,68949 | 4,18065 | 0,17193 | 0,05 | 0 | -9057,3563 | 101394,24963 |
| DHQ, 1 day Control, 42 days | 32006,22222 | 17461,10767 | 2,59225 | 0,78797 | 0,05 | 0 | -29738,10255 | 93750,54699 |
| DHQ, 1 day Control, 56 days | 46888,07778 | 17461,10767 | 3,79757 | 0,28075 | 0,05 | 0 | -14856,24699 | 108632,40255 |
| DHQ, 7 days Control, 1 day | -22065,325 | 16333,37062 | 1,91051 | 0,96432 | 0,05 | 0 | -79821,85222 | 35691,20222 |
| DHQ, 7 days Control, 7 days | -6863,06667 | 16333,37062 | 0,59423 | 1 | 0,05 | 0 | -64619,59388 | 50893,46055 |
| DHQ, 7 days Control, 14 days | 2374,18333 | 16333,37062 | 0,20557 | 1 | 0,05 | 0 | -55382,34388 | 60130,71055 |
| DHQ, 7 days Control, 28 days | 22619,61333 | 15617,68949 | 2,04825 | 0,94324 | 0,05 | 0 | -32606,18963 | 77845,4163 |
| DHQ, 7 days Control, 42 days | 8457,38889 | 17461,10767 | 0,68498 | 1 | 0,05 | 0 | -53286,93588 | 70201,71366 |
| DHQ, 7 days Control, 56 days | 23339,24444 | 17461,10767 | 1,8903 | 0,96686 | 0,05 | 0 | -38405,08033 | 85083,56922 |
| DHQ, 7 days DHQ, 1 day | -23548,83333 | 17461,10767 | 1,90727 | 0,96474 | 0,05 | 0 | -85293,1581 | 38195,49144 |
| DHQ, 14 days Control, 1 day | -33840,15833 | 16333,37062 | 2,93003 | 0,64458 | 0,05 | 0 | -91596,68555 | 23916,36888 |
| DHQ, 14 days Control, 7 days | -18637,9 | 16333,37062 | 1,61375 | 0,98976 | 0,05 | 0 | -76394,42722 | 39118,62722 |
| DHQ, 14 days Control, 14 days | -9400,65 | 16333,37062 | 0,81395 | 0,99998 | 0,05 | 0 | -67157,17722 | 48355,87722 |
| DHQ, 14 days Control, 28 days | 10844,78 | 15617,68949 | 0,98202 | 0,99988 | 0,05 | 0 | -44381,02296 | 66070,58296 |
| DHQ, 14 days Control, 42 days | -3317,44444 | 17461,10767 | 0,26869 | 1 | 0,05 | 0 | -65061,76922 | 58426,88033 |
| DHQ, 14 days Control, 56 days | 11564,41111 | 17461,10767 | 0,93663 | 0,99992 | 0,05 | 0 | -50179,91366 | 73308,73588 |
| DHQ, 14 days DHQ, 1 day | -35323,66667 | 17461,10767 | 2,86094 | 0,67556 | 0,05 | 0 | -97067,99144 | 26420,6581 |
| DHQ, 14 days DHQ, 7 days | -11774,83333 | 17461,10767 | 0,95367 | 0,99991 | 0,05 | 0 | -73519,1581 | 49969,49144 |
| DHQ, 28 days Control, 1 day | -43979,56667 | 15121,76282 | 4,11305 | 0,18834 | 0,05 | 0 | -97451,72046 | 9492,58712 |
| DHQ, 28 days Control, 7 days | -28777,30833 | 15121,76282 | 2,6913 | 0,74847 | 0,05 | 0 | -82249,46212 | 24694,84546 |
| DHQ, 28 days Control, 14 days | -19540,05833 | 15121,76282 | 1,82742 | 0,9739 | 0,05 | 0 | -73012,21212 | 33932,09546 |
| DHQ, 28 days Control, 28 days | 705,37167 | 14345,76382 | 0,06954 | 1 | 0,05 | 0 | -50022,76755 | 51433,51088 |
| DHQ, 28 days Control, 42 days | -13456,85278 | 16333,37062 | 1,16515 | 0,99938 | 0,05 | 0 | -71213,38 | 44299,67444 |
| DHQ, 28 days Control, 56 days | 1425,00278 | 16333,37062 | 0,12338 | 1 | 0,05 | 0 | -56331,52444 | 59181,53 |
| DHQ, 28 days DHQ, 1 day | -45463,075 | 16333,37062 | 3,93639 | 0,23682 | 0,05 | 0 | -103219,6022 | 12293,45222 |
| DHQ, 28 days DHQ, 7 days | -21914,24167 | 16333,37062 | 1,89743 | 0,96598 | 0,05 | 0 | -79670,76888 | 35842,28555 |
| DHQ, 28 days DHQ, 14 days | -10139,40833 | 16333,37062 | 0,87791 | 0,99996 | 0,05 | 0 | -67895,93555 | 47617,11888 |
| DHQ, 42 days Control, 1 day | -25412,59167 | 16333,37062 | 2,20033 | 0,91155 | 0,05 | 0 | -83169,11888 | 32343,93555 |
| DHQ, 42 days Control, 7 days | -10210,33333 | 16333,37062 | 0,88405 | 0,99996 | 0,05 | 0 | -67966,86055 | 47546,19388 |
| DHQ, 42 days Control, 14 days | -973,08333 | 16333,37062 | 0,08425 | 1 | 0,05 | 0 | -58729,61055 | 56783,44388 |
| DHQ, 42 days Control, 28 days | 19272,34667 | 15617,68949 | 1,74515 | 0,98136 | 0,05 | 0 | -35953,4563 | 74498,14963 |
| DHQ, 42 days Control, 42 days | 5110,12222 | 17461,10767 | 0,41388 | 1 | 0,05 | 0 | -56634,20255 | 66854,44699 |
| DHQ, 42 days Control, 56 days | 19991,97778 | 17461,10767 | 1,61919 | 0,98949 | 0,05 | 0 | -41752,34699 | 81736,30255 |
| DHQ, 42 days DHQ, 1 day | -26896,1 | 17461,10767 | 2,17837 | 0,9167 | 0,05 | 0 | -88640,42477 | 34848,22477 |
| DHQ, 42 days DHQ, 7 days | -3347,26667 | 17461,10767 | 0,2711 | 1 | 0,05 | 0 | -65091,59144 | 58397,0581 |
| DHQ, 42 days DHQ, 14 days | 8427,56667 | 17461,10767 | 0,68257 | 1 | 0,05 | 0 | -53316,7581 | 70171,89144 |
| DHQ, 42 days DHQ, 28 days | 18566,975 | 16333,37062 | 1,60761 | 0,99006 | 0,05 | 0 | -39189,55222 | 76323,50222 |
| DHQ, 56 days Control, 1 day | -34532,14167 | 16333,37062 | 2,98994 | 0,61735 | 0,05 | 0 | -92288,66888 | 23224,38555 |
| DHQ, 56 days Control, 7 days | -19329,88333 | 16333,37062 | 1,67366 | 0,98641 | 0,05 | 0 | -77086,41055 | 38426,64388 |
| DHQ, 56 days Control, 14 days | -10092,63333 | 16333,37062 | 0,87386 | 0,99996 | 0,05 | 0 | -67849,16055 | 47663,89388 |
| DHQ, 56 days Control, 28 days | 10152,79667 | 15617,68949 | 0,91936 | 0,99993 | 0,05 | 0 | -45073,0063 | 65378,59963 |
| DHQ, 56 days Control, 42 days | -4009,42778 | 17461,10767 | 0,32473 | 1 | 0,05 | 0 | -65753,75255 | 57734,89699 |
| DHQ, 56 days Control, 56 days | 10872,42778 | 17461,10767 | 0,88058 | 0,99996 | 0,05 | 0 | -50871,89699 | 72616,75255 |
| DHQ, 56 days DHQ, 1 day | -36015,65 | 17461,10767 | 2,91699 | 0,65047 | 0,05 | 0 | -97759,97477 | 25728,67477 |
| DHQ, 56 days DHQ, 7 days | -12466,81667 | 17461,10767 | 1,00971 | 0,99984 | 0,05 | 0 | -74211,14144 | 49277,5081 |
| DHQ, 56 days DHQ, 14 days | -691,98333 | 17461,10767 | 0,05605 | 1 | 0,05 | 0 | -62436,3081 | 61052,34144 |
| DHQ, 56 days DHQ, 28 days | 9447,425 | 16333,37062 | 0,818 | 0,99998 | 0,05 | 0 | -48309,10222 | 67203,95222 |
| DHQ, 56 days DHQ, 42 days | -9119,55 | 17461,10767 | 0,73861 | 0,99999 | 0,05 | 0 | -70863,87477 | 52624,77477 |

### Grouping Letters

#### Bonferroni Test

| | Mean | Groups |
|---|---|---|
| DHQ, 1 day | 189120,6 | A |
| Control, 1 day | 187637,09167 | A |
| Control, 7 days | 172434,83333 | A |
| DHQ, 7 days | 165571,76667 | A |
| Control, 14 days | 163197,58333 | A |
| DHQ, 42 days | 162224,5 | A |
| Control, 42 days | 157114,37778 | A |
| DHQ, 14 days | 153796,93333 | A |
| DHQ, 56 days | 153104,95 | A |
| DHQ, 28 days | 143657,525 | A |
| Control, 28 days | 142952,15333 | A |
| Control, 56 days | 142232,52222 | A |

Means that do not share a letter are significantly different.

#### Scheffe Test

| | Mean | Groups |
|---|---|---|
| DHQ, 1 day | 189120,6 | A |
| Control, 1 day | 187637,09167 | A |
| Control, 7 days | 172434,83333 | A |
| DHQ, 7 days | 165571,76667 | A |
| Control, 14 days | 163197,58333 | A |
| DHQ, 42 days | 162224,5 | A |
| Control, 42 days | 157114,37778 | A |
| DHQ, 14 days | 153796,93333 | A |
| DHQ, 56 days | 153104,95 | A |
| DHQ, 28 days | 143657,525 | A |
| Control, 28 days | 142952,15333 | A |
| Control, 56 days | 142232,52222 | A |

Means that do not share a letter are significantly different.

#### Tukey Test

| | Mean | Groups |
|---|---|---|
| DHQ, 1 day | 189120,6 | A |
| Control, 1 day | 187637,09167 | A |
| Control, 7 days | 172434,83333 | A |
| DHQ, 7 days | 165571,76667 | A |
| Control, 14 days | 163197,58333 | A |
| DHQ, 42 days | 162224,5 | A |
| Control, 42 days | 157114,37778 | A |
| DHQ, 14 days | 153796,93333 | A |
| DHQ, 56 days | 153104,95 | A |
| DHQ, 28 days | 143657,525 | A |
| Control, 28 days | 142952,15333 | A |
| Control, 56 days | 142232,52222 | A |

Means that do not share a letter are significantly different.

Sig equals 1 indicates that the difference of the means is significant at the 0,05 level.
Sig equals 0 indicates that the difference of the means is not significant at the 0,05 level.

### Powers

| | Alpha | Sample Size | Power |
|---|---|---|---|
| Actual Power | 0,05 | 42 | 0,78709 |

*ANOVAOneWay (27.10.2024 21:58:18)*

## *Notes*

| | |
|---|---|
| Description | Perform One-Way ANOVA |
| User Name | yulia |
| Operation Time | 27.10.2024 21:58:18 |
| Report Status | New Analysis Report |
| Data Filter | No |

## *Input Data*

| | Data | Range |
|---|---|---|
| Control, 1 day | [Book1]"SSC Cytometry"!A"Control, 1 day" | [1*:4*] |
| Control, 7 days | [Book1]"SSC Cytometry"!B"Control, 7 days" | [1*:4*] |
| Control, 14 days | [Book1]"SSC Cytometry"!C"Control, 14 days" | [1*:4*] |
| Control, 28 days | [Book1]"SSC Cytometry"!D"Control, 28 days" | [1*:4*] |
| Control, 42 days | [Book1]"SSC Cytometry"!E"Control, 42 days" | [1*:3*] |
| Control, 56 days | [Book1]"SSC Cytometry"!F"Control, 56 days" | [1*:3*] |
| DHQ, 1 day | [Book1]"SSC Cytometry"!G"DHQ, 1 day" | [1*:3*] |
| DHQ, 7 days | [Book1]"SSC Cytometry"!H"DHQ, 7 days" | [1*:3*] |
| DHQ, 14 days | [Book1]"SSC Cytometry"!I"DHQ, 14 days" | [1*:3*] |
| DHQ, 28 days | [Book1]"SSC Cytometry"!J"DHQ, 28 days" | [1*:4*] |
| DHQ, 42 days | [Book1]"SSC Cytometry"!K"DHQ, 42 days" | [1*:3*] |
| DHQ, 56 days | [Book1]"SSC Cytometry"!L"DHQ, 56 days" | [1*:3*] |

## *Descriptive Statistics*

| | N Analysis | N Missing | Mean | Standard Deviation | SE of Mean |
|---|---|---|---|---|---|
| Control, 1 day | 4 | 0 | 93823,66667 | 14099,03706 | 7049,51853 |
| Control, 7 days | 4 | 0 | 90752,86667 | 13262,80851 | 6631,40426 |
| Control, 14 days | 4 | 0 | 89926,075 | 11344,7066 | 5672,3533 |
| Control, 28 days | 4 | 0 | 94694,875 | 12920,31834 | 6460,15917 |
| Control, 42 days | 3 | 0 | 116936,2 | 16036,31856 | 9258,57284 |
| Control, 56 days | 3 | 0 | 121677,42222 | 18103,24492 | 10451,91333 |
| DHQ, 1 day | 3 | 0 | 91917,23333 | 14076,90118 | 8127,30269 |
| DHQ, 7 days | 3 | 0 | 87570,96667 | 9969,85118 | 5756,09626 |
| DHQ, 14 days | 3 | 0 | 82173,83333 | 8345,47868 | 4818,26436 |
| DHQ, 28 days | 4 | 0 | 92354 | 17558,27231 | 8779,13615 |
| DHQ, 42 days | 3 | 0 | 121861,65 | 12212,25 | 7050,74582 |
| DHQ, 56 days | 3 | 0 | 126727,1 | 16113,1 | 9302,90262 |

## *ANOVA*

### *Overall ANOVA*

| | DF | Sum of Squares | Mean Square | F Value | Prob>F |
|---|---|---|---|---|---|
| Model | 11 | 8,70552E9 | 7,91411E8 | 4,05667 | 0,00121 |
| Error | 29 | 5,65758E9 | 1,95089E8 | | |
| Total | 40 | 1,43631E10 | | | |

Null Hypothesis: The means of all levels are equal.
Alternative Hypothesis: The means of one or more levels are different.
At the 0.05 level, the population means are significantly different.

### *Fit Statistics*

| | R-Square | Coeff Var | Root MSE | Data Mean |
|---|---|---|---|---|
| | 0,6061 | 0,13992 | 13967,42123 | 99824,36951 |

### *Means Comparisons*

#### *Bonferroni Test*

| | MeanDiff | SEM | t Value | Prob | Alpha | Sig | LCL | UCL |
|---|---|---|---|---|---|---|---|---|
| Control, 7 days Control, 1 day | -3070,8 | 9876,45827 | -0,31092 | 1 | 0,05 | 0 | -40239,58075 | 34097,98075 |
| Control, 14 days Control, 1 day | -3897,59167 | 9876,45827 | -0,39463 | 1 | 0,05 | 0 | -41066,37242 | 33271,18909 |
| Control, 14 days Control, 7 days | -826,79167 | 9876,45827 | -0,08371 | 1 | 0,05 | 0 | -37995,57242 | 36341,98909 |
| Control, 28 days Control, 1 day | 871,20833 | 9876,45827 | 0,08821 | 1 | 0,05 | 0 | -36297,57242 | 38039,98909 |
| Control, 28 days Control, 7 days | 3942,00833 | 9876,45827 | 0,39913 | 1 | 0,05 | 0 | -33226,77242 | 41110,78909 |
| Control, 28 days Control, 14 da | 4768,8 | 9876,45827 | 0,48285 | 1 | 0,05 | 0 | -32399,98075 | 41937,58075 |
| Control, 42 days Control, 1 day | 23112,53333 | 10667,79417 | 2,16657 | 1 | 0,05 | 0 | -17034,33836 | 63259,40502 |
| Control, 42 days Control, 7 days | 26183,33333 | 10667,79417 | 2,45443 | 1 | 0,05 | 0 | -13963,53836 | 66330,20502 |
| Control, 42 days Control, 14 da | 27010,125 | 10667,79417 | 2,53193 | 1 | 0,05 | 0 | -13136,74669 | 67156,99669 |
| Control, 42 days Control, 28 da | 22241,325 | 10667,79417 | 2,0849 | 1 | 0,05 | 0 | -17905,54669 | 62388,19669 |
| Control, 56 days Control, 1 day | 27853,75556 | 10667,79417 | 2,61101 | 0,93344 | 0,05 | 0 | -12293,11613 | 68000,62725 |
| Control, 56 days Control, 7 days | 30924,55556 | 10667,79417 | 2,89887 | 0,46635 | 0,05 | 0 | -9222,31613 | 71071,42725 |
| Control, 56 days Control, 14 da | 31751,34722 | 10667,79417 | 2,97637 | 0,38495 | 0,05 | 0 | -8395,52447 | 71898,21891 |
| Control, 56 days Control, 28 da | 26982,54722 | 10667,79417 | 2,52935 | 1 | 0,05 | 0 | -13164,32447 | 67129,41891 |
| Control, 56 days Control, 42 da | 4741,22222 | 11404,35168 | 0,41574 | 1 | 0,05 | 0 | -38177,58893 | 47660,03337 |
| DHQ, 1 day Control, 1 day | -1906,43333 | 10667,79417 | -0,17871 | 1 | 0,05 | 0 | -42053,30502 | 38240,43836 |
| DHQ, 1 day Control, 7 days | 1164,36667 | 10667,79417 | 0,10915 | 1 | 0,05 | 0 | -38982,50502 | 41311,23836 |
| DHQ, 1 day Control, 14 days | 1991,15833 | 10667,79417 | 0,18665 | 1 | 0,05 | 0 | -38155,71336 | 42138,03002 |
| DHQ, 1 day Control, 28 days | -2777,64167 | 10667,79417 | -0,26038 | 1 | 0,05 | 0 | -42924,51336 | 37369,23002 |
| DHQ, 1 day Control, 42 days | -25018,96667 | 11404,35168 | -2,19381 | 1 | 0,05 | 0 | -67937,77781 | 17899,84448 |
| DHQ, 1 day Control, 56 days | -29760,18889 | 11404,35168 | -2,60955 | 0,93667 | 0,05 | 0 | -72679,00004 | 13158,62226 |
| DHQ, 7 days Control, 1 day | -6252,7 | 10667,79417 | -0,58613 | 1 | 0,05 | 0 | -46399,57169 | 33894,17169 |
| DHQ, 7 days Control, 7 days | -3181,9 | 10667,79417 | -0,29827 | 1 | 0,05 | 0 | -43328,77169 | 36964,97169 |
| DHQ, 7 days Control, 14 days | -2355,10833 | 10667,79417 | -0,22077 | 1 | 0,05 | 0 | -42501,98002 | 37791,76336 |
| DHQ, 7 days Control, 28 days | -7123,90833 | 10667,79417 | -0,6678 | 1 | 0,05 | 0 | -47270,78002 | 33022,96336 |
| DHQ, 7 days Control, 42 days | -29365,23333 | 11404,35168 | -2,57491 | 1 | 0,05 | 0 | -72284,04448 | 13553,57781 |
| DHQ, 7 days Control, 56 days | -34106,45556 | 11404,35168 | -2,99065 | 0,37151 | 0,05 | 0 | -77025,2667 | 8812,35559 |
| DHQ, 7 days DHQ, 1 day | -4346,26667 | 11404,35168 | -0,38111 | 1 | 0,05 | 0 | -47265,07781 | 38572,54448 |
| DHQ, 14 days Control, 1 day | -11649,83333 | 10667,79417 | -1,09206 | 1 | 0,05 | 0 | -51796,70502 | 28497,03836 |
| DHQ, 14 days Control, 7 days | -8579,03333 | 10667,79417 | -0,8042 | 1 | 0,05 | 0 | -48725,90502 | 31567,83836 |
| DHQ, 14 days Control, 14 days | -7752,24167 | 10667,79417 | -0,7267 | 1 | 0,05 | 0 | -47899,11336 | 32394,63002 |
| DHQ, 14 days Control, 28 days | -12521,04167 | 10667,79417 | -1,17372 | 1 | 0,05 | 0 | -52667,91336 | 27625,83002 |
| DHQ, 14 days Control, 42 days | -34762,36667 | 11404,35168 | -3,04817 | 0,32173 | 0,05 | 0 | -77681,17781 | 8156,44448 |
| DHQ, 14 days Control, 56 days | -39503,58889 | 11404,35168 | -3,4639 | 0,11061 | 0,05 | 0 | -82422,40004 | 3415,22226 |
| DHQ, 14 days DHQ, 1 day | -9743,4 | 11404,35168 | -0,85436 | 1 | 0,05 | 0 | -52662,21115 | 33175,41115 |
| DHQ, 14 days DHQ, 7 days | -5397,13333 | 11404,35168 | -0,47325 | 1 | 0,05 | 0 | -48315,94448 | 37521,67781 |
| DHQ, 28 days Control, 1 day | -1469,66667 | 9876,45827 | -0,14881 | 1 | 0,05 | 0 | -38638,44742 | 35699,11409 |
| DHQ, 28 days Control, 7 days | 1601,13333 | 9876,45827 | 0,16212 | 1 | 0,05 | 0 | -35567,64742 | 38769,91409 |
| DHQ, 28 days Control, 14 days | 2427,925 | 9876,45827 | 0,24583 | 1 | 0,05 | 0 | -34740,85575 | 39596,70575 |
| DHQ, 28 days Control, 28 days | -2340,875 | 9876,45827 | -0,23702 | 1 | 0,05 | 0 | -39509,65575 | 34827,90575 |
| DHQ, 28 days Control, 42 days | -24582,2 | 10667,79417 | -2,30434 | 1 | 0,05 | 0 | -64729,07169 | 15564,67169 |
| DHQ, 28 days Control, 56 days | -29323,42222 | 10667,79417 | -2,74878 | 0,67221 | 0,05 | 0 | -69470,29391 | 10823,44947 |
| DHQ, 28 days DHQ, 1 day | 436,76667 | 10667,79417 | 0,04094 | 1 | 0,05 | 0 | -39710,10502 | 40583,63836 |
| DHQ, 28 days DHQ, 7 days | 4783,03333 | 10667,79417 | 0,44836 | 1 | 0,05 | 0 | -35363,83836 | 44929,90502 |
| DHQ, 28 days DHQ, 14 days | 10180,16667 | 10667,79417 | 0,95429 | 1 | 0,05 | 0 | -29966,70502 | 50327,03836 |
| DHQ, 42 days Control, 1 day | 28037,98333 | 10667,79417 | 2,62828 | 0,89616 | 0,05 | 0 | -12108,88836 | 68184,85502 |
| DHQ, 42 days Control, 7 days | 31108,78333 | 10667,79417 | 2,91614 | 0,44692 | 0,05 | 0 | -9038,08836 | 71255,65502 |
| DHQ, 42 days Control, 14 days | 31935,575 | 10667,79417 | 2,99364 | 0,36875 | 0,05 | 0 | -8211,29669 | 72082,44669 |
| DHQ, 42 days Control, 28 days | 27166,775 | 10667,79417 | 2,54662 | 1 | 0,05 | 0 | -12980,09669 | 67313,64669 |
| DHQ, 42 days Control, 42 days | 4925,45 | 11404,35168 | 0,43189 | 1 | 0,05 | 0 | -37993,36115 | 47844,26115 |
| DHQ, 42 days Control, 56 days | 184,22778 | 11404,35168 | 0,01615 | 1 | 0,05 | 0 | -42734,58337 | 43103,03893 |
| DHQ, 42 days DHQ, 1 day | 29944,41667 | 11404,35168 | 2,6257 | 0,90165 | 0,05 | 0 | -12974,39448 | 72863,22781 |
| DHQ, 42 days DHQ, 7 days | 34290,68333 | 11404,35168 | 3,00681 | 0,35683 | 0,05 | 0 | -8628,12781 | 77209,49448 |
| DHQ, 42 days DHQ, 14 days | 39687,81667 | 11404,35168 | 3,48006 | 0,10602 | 0,05 | 0 | -3230,99448 | 82606,62781 |
| DHQ, 42 days DHQ, 28 days | 29507,65 | 10667,79417 | 2,76605 | 0,64478 | 0,05 | 0 | -10639,22169 | 69654,52169 |
| DHQ, 56 days Control, 1 day | 32903,43333 | 10667,79417 | 3,08437 | 0,29372 | 0,05 | 0 | -7243,43836 | 73050,30502 |
| DHQ, 56 days Control, 7 days | 35974,23333 | 10667,79417 | 3,37223 | 0,14051 | 0,05 | 0 | -4172,63836 | 76121,10502 |
| DHQ, 56 days Control, 14 days | 36801,025 | 10667,79417 | 3,44973 | 0,11479 | 0,05 | 0 | -3345,84669 | 76947,89669 |
| DHQ, 56 days Control, 28 days | 32032,225 | 10667,79417 | 3,0027 | 0,36051 | 0,05 | 0 | -8114,64669 | 72179,09669 |
| DHQ, 56 days Control, 42 days | 9790,9 | 11404,35168 | 0,85852 | 1 | 0,05 | 0 | -33127,91115 | 52709,71115 |
| DHQ, 56 days Control, 56 days | 5049,67778 | 11404,35168 | 0,44279 | 1 | 0,05 | 0 | -37869,13337 | 47968,48893 |
| DHQ, 56 days DHQ, 1 day | 34809,86667 | 11404,35168 | 3,05233 | 0,31838 | 0,05 | 0 | -8108,94448 | 77728,67781 |
| DHQ, 56 days DHQ, 7 days | 39156,13333 | 11404,35168 | 3,43344 | 0,11979 | 0,05 | 0 | -3762,67781 | 82074,94448 |
| DHQ, 56 days DHQ, 14 days | 44553,26667 | 11404,35168 | 3,90669 | 0,034 | 0,05 | 1 | 1634,45552 | 87472,07781 |
| DHQ, 56 days DHQ, 28 days | 34373,1 | 10667,79417 | 3,22214 | 0,20696 | 0,05 | 0 | -5773,77169 | 74519,97169 |
| DHQ, 56 days DHQ, 42 days | 4865,45 | 11404,35168 | 0,42663 | 1 | 0,05 | 0 | -38053,36115 | 47784,26115 |

#### *Scheffe Test*

| | MeanDiff | SEM | F Value | Prob | Alpha | Sig | LCL | UCL |
|---|---|---|---|---|---|---|---|---|
| Control, 7 days Control, 1 day | -3070,8 | 9876,45827 | 0,00879 | 1 | 0,05 | 0 | -50966,00533 | 44824,40533 |
| Control, 14 days Control, 1 day | -3897,59167 | 9876,45827 | 0,01416 | 1 | 0,05 | 0 | -51792,79699 | 43997,61366 |
| Control, 14 days Control, 7 days | -826,79167 | 9876,45827 | 6,37084E-4 | 1 | 0,05 | 0 | -48721,99699 | 47068,41366 |
| Control, 28 days Control, 1 day | 871,20833 | 9876,45827 | 7,07374E-4 | 1 | 0,05 | 0 | -47023,99699 | 48766,41366 |
| Control, 28 days Control, 7 days | 3942,00833 | 9876,45827 | 0,01448 | 1 | 0,05 | 0 | -43953,19699 | 51837,21366 |
| Control, 28 days Control, 14 da | 4768,8 | 9876,45827 | 0,02119 | 1 | 0,05 | 0 | -43126,40533 | 52664,00533 |
| Control, 42 days Control, 1 day | 23112,53333 | 10667,79417 | 0,42673 | 0,93139 | 0,05 | 0 | -28620,20107 | 74845,26774 |
| Control, 42 days Control, 7 days | 26183,33333 | 10667,79417 | 0,54766 | 0,8539 | 0,05 | 0 | -25549,40107 | 77916,06774 |
| Control, 42 days Control, 14 da | 27010,125 | 10667,79417 | 0,58279 | 0,82696 | 0,05 | 0 | -24722,6094 | 78742,8594 |
| Control, 42 days Control, 28 da | 22241,325 | 10667,79417 | 0,39517 | 0,94688 | 0,05 | 0 | -29491,4094 | 73974,0594 |
| Control, 56 days Control, 1 day | 27853,75556 | 10667,79417 | 0,61976 | 0,79701 | 0,05 | 0 | -23878,97885 | 79586,48996 |
| Control, 56 days Control, 7 days | 30924,55556 | 10667,79417 | 0,76395 | 0,67131 | 0,05 | 0 | -20808,17885 | 82657,28996 |
| Control, 56 days Control, 14 da | 31751,34722 | 10667,79417 | 0,80535 | 0,63441 | 0,05 | 0 | -19981,38718 | 83484,08162 |
| Control, 56 days Control, 28 da | 26982,54722 | 10667,79417 | 0,5816 | 0,82789 | 0,05 | 0 | -24750,18718 | 78715,28162 |
| Control, 56 days Control, 42 da | 4741,22222 | 11404,35168 | 0,01571 | 1 | 0,05 | 0 | -50563,39715 | 60045,8416 |
| DHQ, 1 day Control, 1 day | -1906,43333 | 10667,79417 | 0,0029 | 1 | 0,05 | 0 | -53639,16774 | 49826,30107 |
| DHQ, 1 day Control, 7 days | 1164,36667 | 10667,79417 | 0,00108 | 1 | 0,05 | 0 | -50568,36774 | 52897,10107 |
| DHQ, 1 day Control, 14 days | 1991,15833 | 10667,79417 | 0,00317 | 1 | 0,05 | 0 | -49741,57607 | 53723,89274 |
| DHQ, 1 day Control, 28 days | -2777,64167 | 10667,79417 | 0,00616 | 1 | 0,05 | 0 | -54510,37607 | 48955,09274 |
| DHQ, 1 day Control, 42 days | -25018,96667 | 11404,35168 | 0,43753 | 0,9256 | 0,05 | 0 | -80323,58604 | 30285,65271 |
| DHQ, 1 day Control, 56 days | -29760,18889 | 11404,35168 | 0,61907 | 0,79759 | 0,05 | 0 | -85064,80826 | 25544,43049 |
| DHQ, 7 days Control, 1 day | -6252,7 | 10667,79417 | 0,03123 | 1 | 0,05 | 0 | -57985,4344 | 45480,0344 |
| DHQ, 7 days Control, 7 days | -3181,9 | 10667,79417 | 0,00809 | 1 | 0,05 | 0 | -54914,6344 | 48550,8344 |
| DHQ, 7 days Control, 14 days | -2355,10833 | 10667,79417 | 0,00443 | 1 | 0,05 | 0 | -54087,84274 | 49377,62607 |
| DHQ, 7 days Control, 28 days | -7123,90833 | 10667,79417 | 0,04054 | 1 | 0,05 | 0 | -58856,64274 | 44608,82607 |
| DHQ, 7 days Control, 42 days | -29365,23333 | 11404,35168 | 0,60274 | 0,81098 | 0,05 | 0 | -84669,85271 | 25939,38604 |
| DHQ, 7 days Control, 56 days | -34106,45556 | 11404,35168 | 0,81309 | 0,62753 | 0,05 | 0 | -89411,07493 | 21198,16382 |
| DHQ, 7 days DHQ, 1 day | -4346,26667 | 11404,35168 | 0,0132 | 1 | 0,05 | 0 | -59650,88604 | 50958,35271 |
| DHQ, 14 days Control, 1 day | -11649,83333 | 10667,79417 | 0,10842 | 0,99978 | 0,05 | 0 | -63382,56774 | 40082,90107 |
| DHQ, 14 days Control, 7 days | -8579,03333 | 10667,79417 | 0,05879 | 0,99999 | 0,05 | 0 | -60311,76774 | 43153,70107 |
| DHQ, 14 days Control, 14 days | -7752,24167 | 10667,79417 | 0,04801 | 1 | 0,05 | 0 | -59484,97607 | 43980,49274 |
| DHQ, 14 days Control, 28 days | -12521,04167 | 10667,79417 | 0,12524 | 0,99956 | 0,05 | 0 | -64253,77607 | 39211,69274 |
| DHQ, 14 days Control, 42 days | -34762,36667 | 11404,35168 | 0,84467 | 0,59966 | 0,05 | 0 | -90066,98604 | 20542,25271 |
| DHQ, 14 days Control, 56 days | -39503,58889 | 11404,35168 | 1,09079 | 0,40209 | 0,05 | 0 | -94808,20826 | 15801,03049 |
| DHQ, 14 days DHQ, 1 day | -9743,4 | 11404,35168 | 0,06636 | 0,99998 | 0,05 | 0 | -65048,01938 | 45561,21938 |
| DHQ, 14 days DHQ, 7 days | -5397,13333 | 11404,35168 | 0,02036 | 1 | 0,05 | 0 | -60701,75271 | 49907,48604 |
| DHQ, 28 days Control, 1 day | -1469,66667 | 9876,45827 | 0,00201 | 1 | 0,05 | 0 | -49364,87199 | 46425,53866 |
| DHQ, 28 days Control, 7 days | 1601,13333 | 9876,45827 | 0,00239 | 1 | 0,05 | 0 | -46294,07199 | 49496,33866 |
| DHQ, 28 days Control, 14 days | 2427,925 | 9876,45827 | 0,00549 | 1 | 0,05 | 0 | -45467,28033 | 50323,13033 |
| DHQ, 28 days Control, 28 days | -2340,875 | 9876,45827 | 0,00511 | 1 | 0,05 | 0 | -50236,08033 | 45554,33033 |
| DHQ, 28 days Control, 42 days | -24582,2 | 10667,79417 | 0,48272 | 0,89883 | 0,05 | 0 | -76314,9344 | 27150,5344 |
| DHQ, 28 days Control, 56 days | -29323,42222 | 10667,79417 | 0,68689 | 0,73966 | 0,05 | 0 | -81056,15662 | 22409,31218 |
| DHQ, 28 days DHQ, 1 day | 436,76667 | 10667,79417 | 1,5239E-4 | 1 | 0,05 | 0 | -51295,96774 | 52169,50107 |
| DHQ, 28 days DHQ, 7 days | 4783,03333 | 10667,79417 | 0,01828 | 1 | 0,05 | 0 | -46949,70107 | 56515,76774 |
| DHQ, 28 days DHQ, 14 days | 10180,16667 | 10667,79417 | 0,08279 | 0,99994 | 0,05 | 0 | -41552,56774 | 61912,90107 |
| DHQ, 42 days Control, 1 day | 28037,98333 | 10667,79417 | 0,62799 | 0,79017 | 0,05 | 0 | -23694,75107 | 79770,71774 |
| DHQ, 42 days Control, 7 days | 31108,78333 | 10667,79417 | 0,77308 | 0,66316 | 0,05 | 0 | -20623,95107 | 82841,51774 |
| DHQ, 42 days Control, 14 days | 31935,575 | 10667,79417 | 0,81472 | 0,62609 | 0,05 | 0 | -19797,1594 | 83668,3094 |
| DHQ, 42 days Control, 28 days | 27166,775 | 10667,79417 | 0,58957 | 0,82158 | 0,05 | 0 | -24565,9594 | 78899,5094 |
| DHQ, 42 days Control, 42 days | 4925,45 | 11404,35168 | 0,01696 | 1 | 0,05 | 0 | -50379,16938 | 60230,06938 |
| DHQ, 42 days Control, 56 days | 184,22778 | 11404,35168 | 2,37234E-5 | 1 | 0,05 | 0 | -55120,3916 | 55488,84715 |
| DHQ, 42 days DHQ, 1 day | 29944,41667 | 11404,35168 | 0,62676 | 0,7912 | 0,05 | 0 | -25360,20271 | 85249,03604 |
| DHQ, 42 days DHQ, 7 days | 34290,68333 | 11404,35168 | 0,8219 | 0,61973 | 0,05 | 0 | -21013,93604 | 89595,30271 |
| DHQ, 42 days DHQ, 14 days | 39687,81667 | 11404,35168 | 1,10098 | 0,39492 | 0,05 | 0 | -15616,80271 | 94992,43604 |
| DHQ, 42 days DHQ, 28 days | 29507,65 | 10667,79417 | 0,69555 | 0,73206 | 0,05 | 0 | -22225,0844 | 81240,3844 |
| DHQ, 56 days Control, 1 day | 32903,43333 | 10667,79417 | 0,86485 | 0,58203 | 0,05 | 0 | -18829,30107 | 84636,16774 |
| DHQ, 56 days Control, 7 days | 35974,23333 | 10667,79417 | 1,03381 | 0,44379 | 0,05 | 0 | -15758,50107 | 87706,96774 |
| DHQ, 56 days Control, 14 days | 36801,025 | 10667,79417 | 1,08188 | 0,40842 | 0,05 | 0 | -14931,7094 | 88533,7594 |
| DHQ, 56 days Control, 28 days | 32032,225 | 10667,79417 | 0,81966 | 0,62171 | 0,05 | 0 | -19700,5094 | 83764,9594 |
| DHQ, 56 days Control, 42 days | 9790,9 | 11404,35168 | 0,06701 | 0,99998 | 0,05 | 0 | -45513,71938 | 65095,51938 |
| DHQ, 56 days Control, 56 days | 5049,67778 | 11404,35168 | 0,01782 | 1 | 0,05 | 0 | -50254,9416 | 60354,29715 |
| DHQ, 56 days DHQ, 1 day | 34809,86667 | 11404,35168 | 0,84698 | 0,59763 | 0,05 | 0 | -20494,75271 | 90114,48604 |
| DHQ, 56 days DHQ, 7 days | 39156,13333 | 11404,35168 | 1,07168 | 0,41576 | 0,05 | 0 | -16148,48604 | 94460,75271 |
| DHQ, 56 days DHQ, 14 days | 44553,26667 | 11404,35168 | 1,38748 | 0,23099 | 0,05 | 0 | -10751,35271 | 99857,88604 |
| DHQ, 56 days DHQ, 28 days | 34373,1 | 10667,79417 | 0,94383 | 0,51501 | 0,05 | 0 | -17359,6344 | 86105,8344 |
| DHQ, 56 days DHQ, 42 days | 4865,45 | 11404,35168 | 0,01655 | 1 | 0,05 | 0 | -50439,16938 | 60170,06938 |

#### *Tukey Test*

| | MeanDiff | SEM | q Value | Prob | Alpha | Sig | LCL | UCL |
|---|---|---|---|---|---|---|---|---|
| Control, 7 days Control, 1 day | -3070,8 | 9876,45827 | 0,43971 | 1 | 0,05 | 0 | -38089,09604 | 31947,49604 |
| Control, 14 days Control, 1 day | -3897,59167 | 9876,45827 | 0,5581 | 1 | 0,05 | 0 | -38915,88771 | 31120,70438 |
| Control, 14 days Control, 7 days | -826,79167 | 9876,45827 | 0,11839 | 1 | 0,05 | 0 | -35845,08771 | 34191,50438 |
| Control, 28 days Control, 1 day | 871,20833 | 9876,45827 | 0,12475 | 1 | 0,05 | 0 | -34147,08771 | 35889,50438 |
| Control, 28 days Control, 7 days | 3942,00833 | 9876,45827 | 0,56446 | 1 | 0,05 | 0 | -31076,28771 | 38960,30438 |
| Control, 28 days Control, 14 da | 4768,8 | 9876,45827 | 0,68285 | 1 | 0,05 | 0 | -30249,49604 | 39787,09604 |
| Control, 42 days Control, 1 day | 23112,53333 | 10667,79417 | 3,06399 | 0,58377 | 0,05 | 0 | -14711,54939 | 60936,61606 |
| Control, 42 days Control, 7 days | 26183,33333 | 10667,79417 | 3,47109 | 0,40439 | 0,05 | 0 | -11640,74939 | 64007,41606 |
| Control, 42 days Control, 14 da | 27010,125 | 10667,79417 | 3,58069 | 0,36055 | 0,05 | 0 | -10813,95773 | 64834,20773 |
| Control, 42 days Control, 28 da | 22241,325 | 10667,79417 | 2,9485 | 0,6363 | 0,05 | 0 | -15582,75773 | 60065,40773 |
| Control, 56 days Control, 1 day | 27853,75556 | 10667,79417 | 3,69253 | 0,31866 | 0,05 | 0 | -9970,32717 | 65677,83828 |
| Control, 56 days Control, 7 days | 30924,55556 | 10667,79417 | 4,09962 | 0,1933 | 0,05 | 0 | -6899,52717 | 68748,63828 |
| Control, 56 days Control, 14 da | 31751,34722 | 10667,79417 | 4,20923 | 0,1669 | 0,05 | 0 | -6072,7355 | 69575,42995 |
| Control, 56 days Control, 28 da | 26982,54722 | 10667,79417 | 3,57704 | 0,36197 | 0,05 | 0 | -10841,5355 | 64806,62995 |
| Control, 56 days Control, 42 da | 4741,22222 | 11404,35168 | 0,58794 | 1 | 0,05 | 0 | -35694,42307 | 45176,86752 |
| DHQ, 1 day Control, 1 day | -1906,43333 | 10667,79417 | 0,25273 | 1 | 0,05 | 0 | -39730,51606 | 35917,64939 |
| DHQ, 1 day Control, 7 days | 1164,36667 | 10667,79417 | 0,15436 | 1 | 0,05 | 0 | -36659,71606 | 38988,44939 |
| DHQ, 1 day Control, 14 days | 1991,15833 | 10667,79417 | 0,26396 | 1 | 0,05 | 0 | -35832,92439 | 39815,24106 |
| DHQ, 1 day Control, 28 days | -2777,64167 | 10667,79417 | 0,36823 | 1 | 0,05 | 0 | -40601,72439 | 35046,44106 |
| DHQ, 1 day Control, 42 days | -25018,96667 | 11404,35168 | 3,10251 | 0,5662 | 0,05 | 0 | -65454,61196 | 15416,67863 |
| DHQ, 1 day Control, 56 days | -29760,18889 | 11404,35168 | 3,69046 | 0,31941 | 0,05 | 0 | -70195,83418 | 10675,45641 |
| DHQ, 7 days Control, 1 day | -6252,7 | 10667,79417 | 0,82891 | 0,99998 | 0,05 | 0 | -44076,78273 | 31571,38273 |
| DHQ, 7 days Control, 7 days | -3181,9 | 10667,79417 | 0,42182 | 1 | 0,05 | 0 | -41005,98273 | 34642,18273 |
| DHQ, 7 days Control, 14 days | -2355,10833 | 10667,79417 | 0,31221 | 1 | 0,05 | 0 | -40179,19106 | 35468,97439 |
| DHQ, 7 days Control, 28 days | -7123,90833 | 10667,79417 | 0,94441 | 0,99991 | 0,05 | 0 | -44947,99106 | 30700,17439 |
| DHQ, 7 days Control, 42 days | -29365,23333 | 11404,35168 | 3,64148 | 0,33741 | 0,05 | 0 | -69800,87863 | 11070,41196 |
| DHQ, 7 days Control, 56 days | -34106,45556 | 11404,35168 | 4,22942 | 0,16236 | 0,05 | 0 | -74542,10085 | 6329,18974 |
| DHQ, 7 days DHQ, 1 day | -4346,26667 | 11404,35168 | 0,53897 | 1 | 0,05 | 0 | -44781,91196 | 36089,37863 |
| DHQ, 14 days Control, 1 day | -11649,83333 | 10667,79417 | 1,5444 | 0,9927 | 0,05 | 0 | -49473,91606 | 26174,24939 |
| DHQ, 14 days Control, 7 days | -8579,03333 | 10667,79417 | 1,13731 | 0,99949 | 0,05 | 0 | -46403,11606 | 29245,04939 |
| DHQ, 14 days Control, 14 days | -7752,24167 | 10667,79417 | 1,0277 | 0,9998 | 0,05 | 0 | -45576,32439 | 30071,84106 |
| DHQ, 14 days Control, 28 days | -12521,04167 | 10667,79417 | 1,6599 | 0,98712 | 0,05 | 0 | -50345,12439 | 25303,04106 |
| DHQ, 14 days Control, 42 days | -34762,36667 | 11404,35168 | 4,31076 | 0,14507 | 0,05 | 0 | -75198,01196 | 5673,27863 |
| DHQ, 14 days Control, 56 days | -39503,58889 | 11404,35168 | 4,8987 | 0,06015 | 0,05 | 0 | -79939,23418 | 932,05641 |
| DHQ, 14 days DHQ, 1 day | -9743,4 | 11404,35168 | 1,20824 | 0,99912 | 0,05 | 0 | -50179,04529 | 30692,24529 |
| DHQ, 14 days DHQ, 7 days | -5397,13333 | 11404,35168 | 0,66928 | 1 | 0,05 | 0 | -45832,77863 | 35038,51196 |
| DHQ, 28 days Control, 1 day | -1469,66667 | 9876,45827 | 0,21044 | 1 | 0,05 | 0 | -36487,96271 | 33548,62938 |
| DHQ, 28 days Control, 7 days | 1601,13333 | 9876,45827 | 0,22927 | 1 | 0,05 | 0 | -33417,16271 | 36619,42938 |
| DHQ, 28 days Control, 14 days | 2427,925 | 9876,45827 | 0,34766 | 1 | 0,05 | 0 | -32590,37104 | 37446,22104 |
| DHQ, 28 days Control, 28 days | -2340,875 | 9876,45827 | 0,33519 | 1 | 0,05 | 0 | -37359,17104 | 32677,42104 |
| DHQ, 28 days Control, 42 days | -24582,2 | 10667,79417 | 3,25883 | 0,49561 | 0,05 | 0 | -62406,28273 | 13241,88273 |
| DHQ, 28 days Control, 56 days | -29323,42222 | 10667,79417 | 3,88736 | 0,25322 | 0,05 | 0 | -67147,50495 | 8500,6605 |
| DHQ, 28 days DHQ, 1 day | 436,76667 | 10667,79417 | 0,0579 | 1 | 0,05 | 0 | -37387,31606 | 38260,84939 |
| DHQ, 28 days DHQ, 7 days | 4783,03333 | 10667,79417 | 0,63408 | 1 | 0,05 | 0 | -33041,04939 | 42607,11606 |
| DHQ, 28 days DHQ, 14 days | 10180,16667 | 10667,79417 | 1,34957 | 0,99764 | 0,05 | 0 | -27643,91606 | 48004,24939 |
| DHQ, 42 days Control, 1 day | 28037,98333 | 10667,79417 | 3,71695 | 0,30992 | 0,05 | 0 | -9786,09939 | 65862,06606 |
| DHQ, 42 days Control, 7 days | 31108,78333 | 10667,79417 | 4,12405 | 0,18715 | 0,05 | 0 | -6715,29939 | 68932,86606 |
| DHQ, 42 days Control, 14 days | 31935,575 | 10667,79417 | 4,23365 | 0,16143 | 0,05 | 0 | -5888,50773 | 69759,65773 |
| DHQ, 42 days Control, 28 days | 27166,775 | 10667,79417 | 3,60146 | 0,35255 | 0,05 | 0 | -10657,30773 | 64990,85773 |
| DHQ, 42 days Control, 42 days | 4925,45 | 11404,35168 | 0,61079 | 1 | 0,05 | 0 | -35510,19529 | 45361,09529 |
| DHQ, 42 days Control, 56 days | 184,22778 | 11404,35168 | 0,02285 | 1 | 0,05 | 0 | -40251,41752 | 40619,87307 |
| DHQ, 42 days DHQ, 1 day | 29944,41667 | 11404,35168 | 3,7133 | 0,31122 | 0,05 | 0 | -10491,22863 | 70380,06196 |
| DHQ, 42 days DHQ, 7 days | 34290,68333 | 11404,35168 | 4,25227 | 0,15735 | 0,05 | 0 | -6144,96196 | 74726,32863 |
| DHQ, 42 days DHQ, 14 days | 39687,81667 | 11404,35168 | 4,92155 | 0,05801 | 0,05 | 0 | -747,82863 | 80123,46196 |
| DHQ, 42 days DHQ, 28 days | 29507,65 | 10667,79417 | 3,91179 | 0,24572 | 0,05 | 0 | -8316,43273 | 67331,73273 |
| DHQ, 56 days Control, 1 day | 32903,43333 | 10667,79417 | 4,36196 | 0,13497 | 0,05 | 0 | -4920,64939 | 70727,51606 |
| DHQ, 56 days Control, 7 days | 35974,23333 | 10667,79417 | 4,76905 | 0,07371 | 0,05 | 0 | -1849,84939 | 73798,31606 |
| DHQ, 56 days Control, 14 days | 36801,025 | 10667,79417 | 4,87866 | 0,06209 | 0,05 | 0 | -1023,05773 | 74625,10773 |
| DHQ, 56 days Control, 28 days | 32032,225 | 10667,79417 | 4,24646 | 0,15861 | 0,05 | 0 | -5791,85773 | 69856,30773 |
| DHQ, 56 days Control, 42 days | 9790,9 | 11404,35168 | 1,21414 | 0,99908 | 0,05 | 0 | -30644,74529 | 50226,54529 |
| DHQ, 56 days Control, 56 days | 5049,67778 | 11404,35168 | 0,62619 | 1 | 0,05 | 0 | -35385,96752 | 45485,32307 |
| DHQ, 56 days DHQ, 1 day | 34809,86667 | 11404,35168 | 4,31665 | 0,14388 | 0,05 | 0 | -5625,77863 | 75245,51196 |
| DHQ, 56 days DHQ, 7 days | 39156,13333 | 11404,35168 | 4,85561 | 0,06439 | 0,05 | 0 | -1279,51196 | 79591,77863 |
| DHQ, 56 days DHQ, 14 days | 44553,26667 | 11404,35168 | 5,52489 | 0,0213 | 0,05 | 1 | 4117,62137 | 84988,91196 |
| DHQ, 56 days DHQ, 28 days | 34373,1 | 10667,79417 | 4,55679 | 0,10172 | 0,05 | 0 | -3450,98273 | 72197,18273 |
| DHQ, 56 days DHQ, 42 days | 4865,45 | 11404,35168 | 0,60335 | 1 | 0,05 | 0 | -35570,19529 | 45301,09529 |

### *Grouping Letters*

#### *Bonferroni Test*

| | Mean | Groups | |
|---|---|---|---|
| DHQ, 56 days | 126727,1 | A | |
| DHQ, 42 days | 121861,65 | A | B |
| Control, 56 days | 121677,42222 | A | B |
| Control, 42 days | 116936,2 | A | B |
| Control, 28 days | 94694,875 | A | B |
| Control, 1 day | 93823,66667 | A | B |
| DHQ, 28 days | 92354 | A | B |
| DHQ, 1 day | 91917,23333 | A | B |
| Control, 7 days | 90752,86667 | A | B |
| Control, 14 days | 89926,075 | A | B |
| DHQ, 7 days | 87570,96667 | A | B |
| DHQ, 14 days | 82173,83333 | | B |

Means that do not share a letter are significantly different.

#### *Scheffe Test*

| | Mean | Groups |
|---|---|---|
| DHQ, 56 days | 126727,1 | A |
| DHQ, 42 days | 121861,65 | A |
| Control, 56 days | 121677,42222 | A |
| Control, 42 days | 116936,2 | A |
| Control, 28 days | 94694,875 | A |
| Control, 1 day | 93823,66667 | A |
| DHQ, 28 days | 92354 | A |
| DHQ, 1 day | 91917,23333 | A |
| Control, 7 days | 90752,86667 | A |
| Control, 14 days | 89926,075 | A |
| DHQ, 7 days | 87570,96667 | A |
| DHQ, 14 days | 82173,83333 | A |

Means that do not share a letter are significantly different.

#### *Tukey Test*

| | Mean | Groups | |
|---|---|---|---|
| DHQ, 56 days | 126727,1 | A | |
| DHQ, 42 days | 121861,65 | A | B |
| Control, 56 days | 121677,42222 | A | B |
| Control, 42 days | 116936,2 | A | B |
| Control, 28 days | 94694,875 | A | B |
| Control, 1 day | 93823,66667 | A | B |
| DHQ, 28 days | 92354 | A | B |
| DHQ, 1 day | 91917,23333 | A | B |
| Control, 7 days | 90752,86667 | A | B |
| Control, 14 days | 89926,075 | A | B |
| DHQ, 7 days | 87570,96667 | A | B |
| DHQ, 14 days | 82173,83333 | | B |

Means that do not share a letter are significantly different.

Sig equals 1 indicates that the difference of the means is significant at the 0,05 level.
Sig equals 0 indicates that the difference of the means is not significant at the 0,05 level.

## *Powers*

| | Alpha | Sample Size | Power |
|---|---|---|---|
| Actual Power | 0,05 | 41 | 0,98809 |

*ANOVAOneWay (28.10.2024 14:55:30)*

*Notes*

| | |
|---|---|
| Description | Perform One-Way ANOVA |
| User Name | yulia |
| Operation Time | 28.10.2024 14:55:30 |
| Report Status | New Analysis Report |
| Data Filter | No |

*Input Data*

| | Data | Range |
|---|---|---|
| Control, 1 day, DCFDA | [Book1]ROS!A"Control, 1 day, DCFDA" | [1*:3*] |
| Control, 7 days, DCFDA | [Book1]ROS!B"Control, 7 days, DCFDA" | [1*:3*] |
| Control, 14 days, DCFDA | [Book1]ROS!C"Control, 14 days, DCFDA" | [1*:3*] |
| Control, 28 days, DCFDA | [Book1]ROS!D"Control, 28 days, DCFDA" | [1*:3*] |
| Control, 42 days, DCFDA | [Book1]ROS!E"Control, 42 days, DCFDA" | [1*:3*] |
| Control, 56 days, DCFDA | [Book1]ROS!F"Control, 56 days, DCFDA" | [1*:3*] |
| DHQ, 1 day, DCFDA | [Book1]ROS!G"DHQ, 1 day, DCFDA | [1*:3*] |
| DHQ, 7 days, DCFDA | [Book1]ROS!H"DHQ, 7 days, DCFD | [1*:3*] |
| DHQ, 14 days, DCFDA | [Book1]ROS!I"DHQ, 14 days, DCFD | [1*:3*] |
| DHQ, 28 days, DCFDA | [Book1]ROS!J"DHQ, 28 days, DCFDA" | [1*:3*] |
| DHQ, 42 days, DCFDA | [Book1]ROS!K"DHQ, 42 days, DCFDA" | [1*:3*] |
| DHQ, 56 days, DCFDA | [Book1]ROS!L"DHQ, 56 days, DCFDA" | [1*:3*] |
| Control, 1 day, ABAP | [Book1]ROS!M"Control, 1 day, ABA | [1*:3*] |
| Control, 7 days, ABAP | [Book1]ROS!N"Control, 7 days, ABA | [1*:3*] |
| Control, 14 days, ABAP | [Book1]ROS!O"Control, 14 days, ABAP" | [1*:3*] |
| Control, 28 days, ABAP | [Book1]ROS!P"Control, 28 days, ABAP" | [1*:3*] |
| Control, 42 days, ABAP | [Book1]ROS!Q"Control, 42 days, ABAP" | [1*:3*] |
| Control, 56 days, ABAP | [Book1]ROS!R"Control, 56 days, ABAP" | [1*:3*] |
| DHQ, 1 day, ABAP | [Book1]ROS!S"DHQ, 1 day, ABAP" | [1*:3*] |
| DHQ, 7 days, ABAP | [Book1]ROS!T"DHQ, 7 days, ABAP" | [1*:3*] |
| DHQ, 14 days, ABAP | [Book1]ROS!U"DHQ, 14 days, ABA | [1*:3*] |
| DHQ, 28 days, ABAP | [Book1]ROS!V"DHQ, 28 days, ABA | [1*:3*] |
| DHQ, 42 days, ABAP | [Book1]ROS!W"DHQ, 42 days, ABA | [1*:3*] |
| DHQ, 56 days, ABAP | [Book1]ROS!X"DHQ, 56 days, ABAP | [1*:3*] |

*Descriptive Statistics*

| | N Analysis | N Missing | Mean | Standard Deviation | SE of Mean |
|---|---|---|---|---|---|
| Control, 1 day, DCFDA | 3 | 0 | 18799,6125 | 500,77899 | 289,12488 |
| Control, 7 days, DCFDA | 3 | 0 | 11890,7075 | 182,2879 | 105,24397 |
| Control, 14 days, DCFDA | 3 | 0 | 14501,70611 | 363,02077 | 209,59014 |
| Control, 28 days, DCFDA | 3 | 0 | 56850,3 | 897,05449 | 517,91465 |
| Control, 42 days, DCFDA | 3 | 0 | 27409,45444 | 333,55974 | 192,5808 |
| Control, 56 days, DCFDA | 3 | 0 | 8744,82015 | 261,4225 | 150,93235 |
| DHQ, 1 day, DCFDA | 3 | 0 | 8939,61728 | 1414,13259 | 816,44983 |
| DHQ, 7 days, DCFDA | 3 | 0 | 18459,78033 | 104,85689 | 60,53915 |
| DHQ, 14 days, DCFDA | 3 | 0 | 10956,40266 | 440,38984 | 254,25919 |
| DHQ, 28 days, DCFDA | 3 | 0 | 26405,84188 | 593,47073 | 342,64049 |
| DHQ, 42 days, DCFDA | 3 | 0 | 25678,64167 | 2524,03122 | 1457,2501 |
| DHQ, 56 days, DCFDA | 3 | 0 | 10724,41765 | 98,51007 | 56,87481 |
| Control, 1 day, ABAP | 3 | 0 | 48271,04375 | 1476,93774 | 852,7104 |
| Control, 7 days, ABAP | 3 | 0 | 44289,9975 | 2166,89967 | 1251,06011 |
| Control, 14 days, ABAP | 3 | 0 | 36288,93278 | 3356,29547 | 1937,75809 |
| Control, 28 days, ABAP | 3 | 0 | 133754,725 | 6518,86852 | 3763,67049 |
| Control, 42 days, ABAP | 3 | 0 | 93698,31778 | 2506,24434 | 1446,98085 |
| Control, 56 days, ABAP | 3 | 0 | 30736,67939 | 2026,67775 | 1170,10295 |
| DHQ, 1 day, ABAP | 3 | 0 | 13539,49801 | 329,19628 | 190,06156 |
| DHQ, 7 days, ABAP | 3 | 0 | 31660,62767 | 876,91147 | 506,28507 |
| DHQ, 14 days, ABAP | 3 | 0 | 15067,40044 | 1025,72082 | 592,20019 |
| DHQ, 28 days, ABAP | 3 | 0 | 30658,94563 | 964,00468 | 556,56836 |
| DHQ, 42 days, ABAP | 3 | 0 | 25369,525 | 1269,85221 | 733,14951 |
| DHQ, 56 days, ABAP | 3 | 0 | 15865,1451 | 1200,38083 | 693,0402 |

*ANOVA*

*Overall ANOVA*

| | DF | Sum of Squares | Mean Square | F Value | Prob>F |
|---|---|---|---|---|---|
| Model | 23 | 5,78178E10 | 2,51382E9 | 691,2773 | 6,98985E-53 |
| Error | 48 | 1,74551E8 | 3636479,0520 | | |
| Total | 71 | 5,79923E10 | | | |

Null Hypothesis: The means of all levels are equal.
Alternative Hypothesis: The means of one or more levels are different.
At the 0.05 level, the population means are significantly different.

*Fit Statistics*

| R-Square | Coeff Var | Root MSE | Data Mean |
|---|---|---|---|
| 0,99699 | 0,06033 | 1906,95544 | 31606,75584 |

*Means Comparisons*

*Bonferroni Test*

| | MeanDiff | SEM | t Value | Prob | Alpha | Sig | LCL | UCL |
|---|---|---|---|---|---|---|---|---|
| Control, 7 days, DCFDA Control, 1 day, DCFDA | -6908,905 | 1557,0226 | -4,43725 | 0,01467 | 0,05 | 1 | -13227,8634 | -589,9466 |
| Control, 14 days, DCFDA Control, 1 day, DCFDA | -4297,90639 | 1557,0226 | -2,76034 | 1 | 0,05 | 0 | -10616,86479 | 2021,05201 |
| Control, 14 days, DCFDA Control, 7 days, DCFDA | 2610,99861 | 1557,0226 | 1,67692 | 1 | 0,05 | 0 | -3707,95979 | 8929,95701 |
| Control, 28 days, DCFDA Control, 1 day, DCFDA | 38050,6875 | 1557,0226 | 24,43811 | 2,70912E-26 | 0,05 | 1 | 31731,7291 | 44369,6459 |
| Control, 28 days, DCFDA Control, 7 days, DCFDA | 44959,5925 | 1557,0226 | 28,87536 | 1,48819E-29 | 0,05 | 1 | 38640,6341 | 51278,5509 |
| Control, 28 days, DCFDA Control, 14 days, DCFDA | 42348,59389 | 1557,0226 | 27,19845 | 2,23579E-28 | 0,05 | 1 | 36029,63549 | 48667,55229 |
| Control, 42 days, DCFDA Control, 1 day, DCFDA | 8609,84194 | 1557,0226 | 5,52968 | 3,58778E-4 | 0,05 | 1 | 2290,88354 | 14928,80035 |
| Control, 42 days, DCFDA Control, 7 days, DCFDA | 15518,74694 | 1557,0226 | 9,96694 | 7,79051E-11 | 0,05 | 1 | 9199,78854 | 21837,70535 |
| Control, 42 days, DCFDA Control, 14 days, DCFDA | 12907,74833 | 1557,0226 | 8,29002 | 2,22343E-8 | 0,05 | 1 | 6588,78993 | 19226,70674 |
| Control, 42 days, DCFDA Control, 28 days, DCFDA | -29440,84556 | 1557,0226 | -18,90843 | 1,92059E-21 | 0,05 | 1 | -35759,80396 | -23121,88715 |
| Control, 56 days, DCFDA Control, 1 day, DCFDA | -10054,79235 | 1557,0226 | -6,4577 | 1,37827E-5 | 0,05 | 1 | -16373,75075 | -3735,83395 |
| Control, 56 days, DCFDA Control, 7 days, DCFDA | -3145,88735 | 1557,0226 | -2,02045 | 1 | 0,05 | 0 | -9464,84575 | 3173,07105 |
| Control, 56 days, DCFDA Control, 14 days, DCFDA | -5756,88596 | 1557,0226 | -3,69737 | 0,15435 | 0,05 | 0 | -12075,84436 | 562,07244 |
| Control, 56 days, DCFDA Control, 28 days, DCFDA | -48105,47985 | 1557,0226 | -30,89581 | 6,81361E-31 | 0,05 | 1 | -54424,43825 | -41786,52145 |
| Control, 56 days, DCFDA Control, 42 days, DCFDA | -18664,63429 | 1557,0226 | -11,98739 | 1,33872E-13 | 0,05 | 1 | -24983,59269 | -12345,67589 |
| DHQ, 1 day, DCFDA Control, 1 day, DCFDA | -9859,99522 | 1557,0226 | -6,3326 | 2,14376E-5 | 0,05 | 1 | -16178,95362 | -3541,03682 |
| DHQ, 1 day, DCFDA Control, 7 days, DCFDA | -2951,09022 | 1557,0226 | -1,89534 | 1 | 0,05 | 0 | -9270,04862 | 3367,86818 |
| DHQ, 1 day, DCFDA Control, 14 days, DCFDA | -5562,08883 | 1557,0226 | -3,57226 | 0,22564 | 0,05 | 0 | -11881,04723 | 756,86957 |
| DHQ, 1 day, DCFDA Control, 28 days, DCFDA | -47910,68272 | 1557,0226 | -30,7707 | 8,20312E-31 | 0,05 | 1 | -54229,64112 | -41591,72432 |
| DHQ, 1 day, DCFDA Control, 42 days, DCFDA | -18469,83716 | 1557,0226 | -11,86228 | 1,95566E-13 | 0,05 | 1 | -24788,79556 | -12150,87876 |
| DHQ, 1 day, DCFDA Control, 56 days, DCFDA | 194,79713 | 1557,0226 | 0,12511 | 1 | 0,05 | 0 | -6124,16127 | 6513,75553 |
| DHQ, 7 days, DCFDA Control, 1 day, DCFDA | -339,83217 | 1557,0226 | -0,21826 | 1 | 0,05 | 0 | -6658,79057 | 5979,12624 |
| DHQ, 7 days, DCFDA Control, 7 days, DCFDA | 6569,07283 | 1557,0226 | 4,219 | 0,02987 | 0,05 | 1 | 250,11443 | 12888,03124 |
| DHQ, 7 days, DCFDA Control, 14 days, DCFDA | 3958,07422 | 1557,0226 | 2,54208 | 1 | 0,05 | 0 | -2360,88418 | 10277,03262 |
| DHQ, 7 days, DCFDA Control, 28 days, DCFDA | -38390,51967 | 1557,0226 | -24,65637 | 1,82339E-26 | 0,05 | 1 | -44709,47807 | -32071,56126 |
| DHQ, 7 days, DCFDA Control, 42 days, DCFDA | -8949,67411 | 1557,0226 | -5,74794 | 1,67538E-4 | 0,05 | 1 | -15268,63251 | -2630,71571 |
| DHQ, 7 days, DCFDA Control, 56 days, DCFDA | 9714,96018 | 1557,0226 | 6,23945 | 2,97783E-5 | 0,05 | 1 | 3396,00178 | 16033,91858 |
| DHQ, 7 days, DCFDA DHQ, 1 day, DCFDA | 9520,16305 | 1557,0226 | 6,11434 | 4,62792E-5 | 0,05 | 1 | 3201,20465 | 15839,12145 |
| DHQ, 14 days, DCFDA Control, 1 day, DCFDA | -7843,20984 | 1557,0226 | -5,03731 | 0,00196 | 0,05 | 1 | -14162,16824 | -1524,25144 |
| DHQ, 14 days, DCFDA Control, 7 days, DCFDA | -934,30484 | 1557,0226 | -0,60006 | 1 | 0,05 | 0 | -7253,26324 | 5384,65356 |
| DHQ, 14 days, DCFDA Control, 14 days, DCFDA | -3545,30345 | 1557,0226 | -2,27698 | 1 | 0,05 | 0 | -9864,26185 | 2773,65495 |
| DHQ, 14 days, DCFDA Control, 28 days, DCFDA | -45893,89734 | 1557,0226 | -29,47542 | 5,83886E-30 | 0,05 | 1 | -52212,85574 | -39574,93894 |
| DHQ, 14 days, DCFDA Control, 42 days, DCFDA | -16453,05179 | 1557,0226 | -10,567 | 1,11416E-11 | 0,05 | 1 | -22772,01019 | -10134,09338 |
| DHQ, 14 days, DCFDA Control, 56 days, DCFDA | 2211,58251 | 1557,0226 | 1,42039 | 1 | 0,05 | 0 | -4107,37589 | 8530,54091 |
| DHQ, 14 days, DCFDA DHQ, 1 day, DCFDA | 2016,78538 | 1557,0226 | 1,29528 | 1 | 0,05 | 0 | -4302,17303 | 8335,74378 |
| DHQ, 14 days, DCFDA DHQ, 7 days, DCFDA | -7503,37767 | 1557,0226 | -4,81906 | 0,00411 | 0,05 | 1 | -13822,33608 | -1184,41927 |
| DHQ, 28 days, DCFDA Control, 1 day, DCFDA | 7606,22938 | 1557,0226 | 4,88511 | 0,00329 | 0,05 | 1 | 1287,27097 | 13925,18778 |
| DHQ, 28 days, DCFDA Control, 7 days, DCFDA | 14515,13438 | 1557,0226 | 9,32237 | 6,60359E-10 | 0,05 | 1 | 8196,17597 | 20834,09278 |
| DHQ, 28 days, DCFDA Control, 14 days, DCFDA | 11904,13576 | 1557,0226 | 7,64545 | 2,09547E-7 | 0,05 | 1 | 5585,17736 | 18223,09417 |
| DHQ, 28 days, DCFDA Control, 28 days, DCFDA | -30444,45812 | 1557,0226 | -19,553 | 4,6064E-22 | 0,05 | 1 | -36763,41653 | -24125,49972 |
| DHQ, 28 days, DCFDA Control, 42 days, DCFDA | -1003,61257 | 1557,0226 | -0,64457 | 1 | 0,05 | 0 | -7322,57097 | 5315,34583 |
| DHQ, 28 days, DCFDA Control, 56 days, DCFDA | 17661,02172 | 1557,0226 | 11,34282 | 9,64122E-13 | 0,05 | 1 | 11342,06332 | 23979,98013 |
| DHQ, 28 days, DCFDA DHQ, 1 day, DCFDA | 17466,22459 | 1557,0226 | 11,21771 | 1,42317E-12 | 0,05 | 1 | 11147,26619 | 23785,18299 |
| DHQ, 28 days, DCFDA DHQ, 7 days, DCFDA | 7946,06154 | 1557,0226 | 5,10337 | 0,00156 | 0,05 | 1 | 1627,10314 | 14265,01994 |
| DHQ, 28 days, DCFDA DHQ, 14 days, DCFDA | 15449,43922 | 1557,0226 | 9,92242 | 9,01537E-11 | 0,05 | 1 | 9130,48081 | 21768,39762 |
| DHQ, 42 days, DCFDA Control, 1 day, DCFDA | 6879,02917 | 1557,0226 | 4,41807 | 0,01562 | 0,05 | 1 | 560,07076 | 13197,98757 |
| DHQ, 42 days, DCFDA Control, 7 days, DCFDA | 13787,93417 | 1557,0226 | 8,85532 | 3,19873E-9 | 0,05 | 1 | 7468,97576 | 20106,89257 |
| DHQ, 42 days, DCFDA Control, 14 days, DCFDA | 11176,93556 | 1557,0226 | 7,1784 | 1,0817E-6 | 0,05 | 1 | 4857,97715 | 17495,89396 |
| DHQ, 42 days, DCFDA Control, 28 days, DCFDA | -31171,65833 | 1557,0226 | -20,02004 | 1,6748E-22 | 0,05 | 1 | -37490,61673 | -24852,69993 |
| DHQ, 42 days, DCFDA Control, 42 days, DCFDA | -1730,81278 | 1557,0226 | -1,11162 | 1 | 0,05 | 0 | -8049,77118 | 4588,14562 |
| DHQ, 42 days, DCFDA Control, 56 days, DCFDA | 16933,82152 | 1557,0226 | 10,87577 | 4,16821E-12 | 0,05 | 1 | 10614,86311 | 23252,77992 |
| DHQ, 42 days, DCFDA DHQ, 1 day, DCFDA | 16739,02438 | 1557,0226 | 10,75066 | 6,19906E-12 | 0,05 | 1 | 10420,06598 | 23057,98279 |
| DHQ, 42 days, DCFDA DHQ, 7 days, DCFDA | 7218,86133 | 1557,0226 | 4,63632 | 0,00758 | 0,05 | 1 | 899,90293 | 13537,81974 |
| DHQ, 42 days, DCFDA DHQ, 14 days, DCFDA | 14722,23901 | 1557,0226 | 9,45538 | 4,23183E-10 | 0,05 | 1 | 8403,28061 | 21041,19741 |
| DHQ, 42 days, DCFDA DHQ, 28 days, DCFDA | -727,20021 | 1557,0226 | -0,46705 | 1 | 0,05 | 0 | -7046,15861 | 5591,75819 |
| DHQ, 56 days, DCFDA Control, 1 day, DCFDA | -8075,19485 | 1557,0226 | -5,18631 | 0,00118 | 0,05 | 1 | -14394,15325 | -1756,23645 |
| DHQ, 56 days, DCFDA Control, 7 days, DCFDA | -1166,28985 | 1557,0226 | -0,74905 | 1 | 0,05 | 0 | -7485,24825 | 5152,66855 |
| DHQ, 56 days, DCFDA Control, 14 days, DCFDA | -3777,28846 | 1557,0226 | -2,42597 | 1 | 0,05 | 0 | -10096,24687 | 2541,66994 |
| DHQ, 56 days, DCFDA Control, 28 days, DCFDA | -46125,88235 | 1557,0226 | -29,62441 | 4,64073E-30 | 0,05 | 1 | -52444,84075 | -39806,92395 |
| DHQ, 56 days, DCFDA Control, 42 days, DCFDA | -16685,0368 | 1557,0226 | -10,71599 | 6,92234E-12 | 0,05 | 1 | -23003,9952 | -10366,0784 |
| DHQ, 56 days, DCFDA Control, 56 days, DCFDA | 1979,5975 | 1557,0226 | 1,2714 | 1 | 0,05 | 0 | -4339,3609 | 8298,5559 |
| DHQ, 56 days, DCFDA DHQ, 1 day, DCFDA | 1784,80036 | 1557,0226 | 1,14629 | 1 | 0,05 | 0 | -4534,15804 | 8103,75877 |
| DHQ, 56 days, DCFDA DHQ, 7 days, DCFDA | -7735,36269 | 1557,0226 | -4,96805 | 0,00248 | 0,05 | 1 | -14054,32109 | -1416,40428 |
| DHQ, 56 days, DCFDA DHQ, 14 days, DCFDA | -231,98501 | 1557,0226 | -0,14899 | 1 | 0,05 | 0 | -6550,94341 | 6086,97339 |
| DHQ, 56 days, DCFDA DHQ, 28 days, DCFDA | -15681,42423 | 1557,0226 | -10,07142 | 5,53504E-11 | 0,05 | 1 | -22000,38263 | -9362,46583 |
| DHQ, 56 days, DCFDA DHQ, 42 days, DCFDA | -14954,22402 | 1557,0226 | -9,60437 | 2,5769E-10 | 0,05 | 1 | -21273,18242 | -8635,26562 |
| Control, 1 day, ABAP Control, 1 day, DCFDA | 29471,43125 | 1557,0226 | 18,92807 | 1,8378E-21 | 0,05 | 1 | 23152,47285 | 35790,38965 |
| Control, 1 day, ABAP Control, 7 days, DCFDA | 36380,33625 | 1557,0226 | 23,36532 | 1,98377E-25 | 0,05 | 1 | 30061,37785 | 42699,29465 |
| Control, 1 day, ABAP Control, 14 days, DCFDA | 33769,33764 | 1557,0226 | 21,68841 | 5,2272E-24 | 0,05 | 1 | 27450,37924 | 40088,29604 |
| Control, 1 day, ABAP Control, 28 days, DCFDA | -8579,25625 | 1557,0226 | -5,51004 | 3,84142E-4 | 0,05 | 1 | -14898,21465 | -2260,29785 |
| Control, 1 day, ABAP Control, 42 days, DCFDA | 20861,58931 | 1557,0226 | 13,39839 | 2,1409E-15 | 0,05 | 1 | 14542,6309 | 27180,54771 |
| Control, 1 day, ABAP Control, 56 days, DCFDA | 39526,2236 | 1557,0226 | 25,38577 | 4,96091E-27 | 0,05 | 1 | 33207,2652 | 45845,182 |
| Control, 1 day, ABAP DHQ, 1 day, DCFDA | 39331,42647 | 1557,0226 | 25,26067 | 6,18761E-27 | 0,05 | 1 | 33012,46807 | 45650,38487 |
| Control, 1 day, ABAP DHQ, 7 days, DCFDA | 29811,26342 | 1557,0226 | 19,14633 | 1,12901E-21 | 0,05 | 1 | 23492,30501 | 36130,22182 |
| Control, 1 day, ABAP DHQ, 14 days, DCFDA | 37314,64109 | 1557,0226 | 23,96538 | 6,45347E-26 | 0,05 | 1 | 30995,68269 | 43633,59949 |
| Control, 1 day, ABAP DHQ, 28 days, DCFDA | 21865,20187 | 1557,0226 | 14,04296 | 3,51789E-16 | 0,05 | 1 | 15546,24347 | 28184,16028 |
| Control, 1 day, ABAP DHQ, 42 days, DCFDA | 22592,40208 | 1557,0226 | 14,51 | 9,81116E-17 | 0,05 | 1 | 16273,44368 | 28911,36049 |
| Control, 1 day, ABAP DHQ, 56 days, DCFDA | 37546,6261 | 1557,0226 | 24,11437 | 4,90122E-26 | 0,05 | 1 | 31227,6677 | 43865,5845 |
| Control, 7 days, ABAP Control, 1 day, DCFDA | 25490,385 | 1557,0226 | 16,37124 | 7,77899E-19 | 0,05 | 1 | 19171,4266 | 31809,3434 |
| Control, 7 days, ABAP Control, 7 days, DCFDA | 32399,29 | 1557,0226 | 20,80849 | 3,16436E-23 | 0,05 | 1 | 26080,3316 | 38718,2484 |
| Control, 7 days, ABAP Control, 14 days, DCFDA | 29788,29139 | 1557,0226 | 19,13157 | 1,16665E-21 | 0,05 | 1 | 23469,33299 | 36107,24979 |
| Control, 7 days, ABAP Control, 28 days, DCFDA | -12560,3025 | 1557,0226 | -8,06687 | 4,81718E-8 | 0,05 | 1 | -18879,2609 | -6241,3441 |
| Control, 7 days, ABAP Control, 42 days, DCFDA | 16880,54306 | 1557,0226 | 10,84155 | 4,64526E-12 | 0,05 | 1 | 10561,58465 | 23199,50146 |
| Control, 7 days, ABAP Control, 56 days, DCFDA | 35545,17735 | 1557,0226 | 22,82894 | 5,52646E-25 | 0,05 | 1 | 29226,21895 | 41864,13575 |
| Control, 7 days, ABAP DHQ, 1 day, DCFDA | 35350,38022 | 1557,0226 | 22,70383 | 7,03862E-25 | 0,05 | 1 | 29031,42182 | 41669,33862 |
| Control, 7 days, ABAP DHQ, 7 days, DCFDA | 25830,21717 | 1557,0226 | 16,58949 | 4,52361E-19 | 0,05 | 1 | 19511,25876 | 32149,17557 |
| Control, 7 days, ABAP DHQ, 14 days, DCFDA | 33333,59484 | 1557,0226 | 21,40855 | 9,20762E-24 | 0,05 | 1 | 27014,63644 | 39652,55324 |
| Control, 7 days, ABAP DHQ, 28 days, DCFDA | 17884,15562 | 1557,0226 | 11,48612 | 6,18702E-13 | 0,05 | 1 | 11565,19722 | 24203,11403 |
| Control, 7 days, ABAP DHQ, 42 days, DCFDA | 18611,35583 | 1557,0226 | 11,95317 | 1,48465E-13 | 0,05 | 1 | 12292,39743 | 24930,31424 |
| Control, 7 days, ABAP DHQ, 56 days, DCFDA | 33565,57985 | 1557,0226 | 21,55754 | 6,80644E-24 | 0,05 | 1 | 27246,62145 | 39884,53825 |
| Control, 7 days, ABAP Control, 1 day, ABAP | -3981,04625 | 1557,0226 | -2,55683 | 1 | 0,05 | 0 | -10300,00465 | 2337,91215 |
| Control, 14 days, ABAP Control, 1 day, DCFDA | 17489,32028 | 1557,0226 | 11,23254 | 1,35881E-12 | 0,05 | 1 | 11170,36188 | 23808,27868 |
| Control, 14 days, ABAP Control, 7 days, DCFDA | 24398,22528 | 1557,0226 | 15,66998 | 4,59983E-18 | 0,05 | 1 | 18079,26688 | 30717,18368 |
| Control, 14 days, ABAP Control, 14 days, DCFDA | 21787,22667 | 1557,0226 | 13,99288 | 4,04041E-16 | 0,05 | 1 | 15468,26826 | 28106,18507 |
| Control, 14 days, ABAP Control, 28 days, DCFDA | -20561,36722 | 1557,0226 | -13,20557 | 3,71165E-15 | 0,05 | 1 | -26880,32562 | -14242,40882 |
| Control, 14 days, ABAP Control, 42 days, DCFDA | 8879,47833 | 1557,0226 | 5,70286 | 1,96145E-4 | 0,05 | 1 | 2560,51993 | 15198,43674 |
| Control, 14 days, ABAP Control, 56 days, DCFDA | 27544,11263 | 1557,0226 | 17,69025 | 3,16839E-20 | 0,05 | 1 | 21225,15422 | 33863,07103 |
| Control, 14 days, ABAP DHQ, 1 day, DCFDA | 27349,3155 | 1557,0226 | 17,56514 | 4,25979E-20 | 0,05 | 1 | 21030,35709 | 33668,2739 |
| Control, 14 days, ABAP DHQ, 7 days, DCFDA | 17829,15244 | 1557,0226 | 11,4508 | 6,90023E-13 | 0,05 | 1 | 11510,19404 | 24148,11085 |
| Control, 14 days, ABAP DHQ, 14 days, DCFDA | 25332,53012 | 1557,0226 | 16,26985 | 1,0024E-18 | 0,05 | 1 | 19013,57172 | 31651,48852 |
| Control, 14 days, ABAP DHQ, 28 days, DCFDA | 9883,0909 | 1557,0226 | 6,34743 | 2,03441E-5 | 0,05 | 1 | 3564,1325 | 16202,0493 |
| Control, 14 days, ABAP DHQ, 42 days, DCFDA | 10610,29111 | 1557,0226 | 6,81447 | 3,90754E-6 | 0,05 | 1 | 4291,33271 | 16929,24951 |
| Control, 14 days, ABAP DHQ, 56 days, DCFDA | 25564,51513 | 1557,0226 | 16,41885 | 6,9084E-19 | 0,05 | 1 | 19245,55673 | 31883,47353 |
| Control, 14 days, ABAP Control, 1 day, ABAP | -11982,11097 | 1557,0226 | -7,69553 | 1,75849E-7 | 0,05 | 1 | -18301,06937 | -5663,15257 |
| Control, 14 days, ABAP Control, 7 days, ABAP | -8001,06472 | 1557,0226 | -5,1387 | 0,00139 | 0,05 | 1 | -14320,02312 | -1682,10632 |
| Control, 28 days, ABAP Control, 1 day, DCFDA | 114955,1125 | 1557,0226 | 73,83009 | 1,21432E-48 | 0,05 | 1 | 108636,1541 | 121274,0709 |
| Control, 28 days, ABAP Control, 7 days, DCFDA | 121864,0175 | 1557,0226 | 78,26734 | 7,54073E-50 | 0,05 | 1 | 115545,0591 | 128182,9759 |
| Control, 28 days, ABAP Control, 14 days, DCFDA | 119253,0188 | 1557,0226 | 76,59042 | 2,11549E-49 | 0,05 | 1 | 112934,0604 | 125571,9772 |
| Control, 28 days, ABAP Control, 28 days, DCFDA | 76904,425 | 1557,0226 | 49,39198 | 2,26468E-40 | 0,05 | 1 | 70585,4666 | 83223,3834 |
| Control, 28 days, ABAP Control, 42 days, DCFDA | 106345,2705 | 1557,0226 | 68,3004 | 4,92413E-47 | 0,05 | 1 | 100026,3121 | 112664,2289 |
| Control, 28 days, ABAP Control, 56 days, DCFDA | 125009,9048 | 1557,0226 | 80,28779 | 2,23863E-50 | 0,05 | 1 | 118690,9464 | 131328,8632 |
| Control, 28 days, ABAP DHQ, 1 day, DCFDA | 124815,1077 | 1557,0226 | 80,16268 | 2,41134E-50 | 0,05 | 1 | 118496,1493 | 131134,0661 |
| Control, 28 days, ABAP DHQ, 7 days, DCFDA | 115294,9446 | 1557,0226 | 74,04834 | 1,05516E-48 | 0,05 | 1 | 108975,9862 | 121613,9030 |
| Control, 28 days, ABAP DHQ, 14 days, DCFDA | 122798,3223 | 1557,0226 | 78,8674 | 5,24058E-50 | 0,05 | 1 | 116479,3639 | 129117,2807 |
| Control, 28 days, ABAP DHQ, 28 days, DCFDA | 107348,8831 | 1557,0226 | 68,94497 | 3,15097E-47 | 0,05 | 1 | 101029,9247 | 113667,8415 |
| Control, 28 days, ABAP DHQ, 42 days, DCFDA | 108076,0833 | 1557,0226 | 69,41202 | 2,28594E-47 | 0,05 | 1 | 101757,1249 | 114395,0417 |
| Control, 28 days, ABAP DHQ, 56 days, DCFDA | 123030,3073 | 1557,0226 | 79,01639 | 4,78978E-50 | 0,05 | 1 | 116711,3489 | 129349,2657 |
| Control, 28 days, ABAP Control, 1 day, ABAP | 85483,68125 | 1557,0226 | 54,90202 | 1,54114E-42 | 0,05 | 1 | 79164,72285 | 91802,63965 |
| Control, 28 days, ABAP Control, 7 days, ABAP | 89464,7275 | 1557,0226 | 57,45885 | 1,78998E-43 | 0,05 | 1 | 83145,7691 | 95783,6859 |
| Control, 28 days, ABAP Control, 14 days, ABAP | 97465,79222 | 1557,0226 | 62,59755 | 3,09179E-45 | 0,05 | 1 | 91146,83382 | 103784,7506 |
| Control, 42 days, ABAP Control, 1 day, DCFDA | 74898,70528 | 1557,0226 | 48,1038 | 7,85649E-40 | 0,05 | 1 | 68579,74688 | 81217,66368 |
| Control, 42 days, ABAP Control, 7 days, DCFDA | 81807,61028 | 1557,0226 | 52,54106 | 1,22878E-41 | 0,05 | 1 | 75488,65188 | 88126,56868 |
| Control, 42 days, ABAP Control, 14 days, DCFDA | 79196,61167 | 1557,0226 | 50,86414 | 5,67524E-41 | 0,05 | 1 | 72877,65326 | 85515,57007 |
| Control, 42 days, ABAP Control, 28 days, DCFDA | 36848,01778 | 1557,0226 | 23,66569 | 1,12735E-25 | 0,05 | 1 | 30529,05938 | 43166,97618 |
| Control, 42 days, ABAP Control, 42 days, DCFDA | 66288,86333 | 1557,0226 | 42,57412 | 2,41907E-37 | 0,05 | 1 | 59969,90493 | 72607,82174 |
| Control, 42 days, ABAP Control, 56 days, DCFDA | 84953,49763 | 1557,0226 | 54,56151 | 2,06793E-42 | 0,05 | 1 | 78634,53922 | 91272,45603 |
| Control, 42 days, ABAP DHQ, 1 day, DCFDA | 84758,7005 | 1557,0226 | 54,4364 | 2,30487E-42 | 0,05 | 1 | 78439,74209 | 91077,6589 |
| Control, 42 days, ABAP DHQ, 7 days, DCFDA | 75238,53744 | 1557,0226 | 48,32206 | 6,34932E-40 | 0,05 | 1 | 68919,57904 | 81557,49585 |
| Control, 42 days, ABAP DHQ, 14 days, DCFDA | 82741,91512 | 1557,0226 | 53,14111 | 7,18878E-42 | 0,05 | 1 | 76422,95672 | 89060,87352 |
| Control, 42 days, ABAP DHQ, 28 days, DCFDA | 67292,4759 | 1557,0226 | 43,21869 | 1,1973E-37 | 0,05 | 1 | 60973,5175 | 73611,4343 |
| Control, 42 days, ABAP DHQ, 42 days, DCFDA | 68019,67611 | 1557,0226 | 43,68573 | 7,23727E-38 | 0,05 | 1 | 61700,71771 | 74338,63451 |
| Control, 42 days, ABAP DHQ, 56 days, DCFDA | 82973,90013 | 1557,0226 | 53,29011 | 6,29856E-42 | 0,05 | 1 | 76654,94173 | 89292,85853 |
| Control, 42 days, ABAP Control, 1 day, ABAP | 45427,27403 | 1557,0226 | 29,17573 | 9,29669E-30 | 0,05 | 1 | 39108,31563 | 51746,23243 |
| Control, 42 days, ABAP Control, 7 days, ABAP | 49408,32028 | 1557,0226 | 31,73256 | 2,00351E-31 | 0,05 | 1 | 43089,36188 | 55727,27868 |
| Control, 42 days, ABAP Control, 14 days, ABAP | 57409,385 | 1557,0226 | 36,87126 | 1,96889E-34 | 0,05 | 1 | 51090,4266 | 63728,3434 |
| Control, 42 days, ABAP Control, 28 days, ABAP | -40056,40722 | 1557,0226 | -25,72629 | 2,73173E-27 | 0,05 | 1 | -46375,36562 | -33737,44882 |
| Control, 56 days, ABAP Control, 1 day, DCFDA | 11937,06689 | 1557,0226 | 7,6666 | 1,94589E-7 | 0,05 | 1 | 5618,10849 | 18256,0253 |
| Control, 56 days, ABAP Control, 7 days, DCFDA | 18845,97189 | 1557,0226 | 12,10385 | 9,4243E-14 | 0,05 | 1 | 12527,01349 | 25164,9303 |
| Control, 56 days, ABAP Control, 14 days, DCFDA | 16234,97328 | 1557,0226 | 10,42693 | 1,74725E-11 | 0,05 | 1 | 9916,01488 | 22553,93168 |
| Control, 56 days, ABAP Control, 28 days, DCFDA | -26113,62061 | 1557,0226 | -16,77151 | 2,88946E-19 | 0,05 | 1 | -32432,57901 | -19794,6622 |
| Control, 56 days, ABAP Control, 42 days, DCFDA | 3327,22495 | 1557,0226 | 2,13692 | 1 | 0,05 | 0 | -2991,73345 | 9646,18335 |
| Control, 56 days, ABAP Control, 56 days, DCFDA | 21991,85924 | 1557,0226 | 14,1243 | 2,81108E-16 | 0,05 | 1 | 15672,90084 | 28310,81764 |
| Control, 56 days, ABAP DHQ, 1 day, DCFDA | 21797,06211 | 1557,0226 | 13,99919 | 3,97038E-16 | 0,05 | 1 | 15478,10371 | 28116,02051 |
| Control, 56 days, ABAP DHQ, 7 days, DCFDA | 12276,89906 | 1557,0226 | 7,88486 | 9,07634E-8 | 0,05 | 1 | 5957,94066 | 18595,85746 |
| Control, 56 days, ABAP DHQ, 14 days, DCFDA | 19780,27674 | 1557,0226 | 12,70391 | 1,58795E-14 | 0,05 | 1 | 13461,31833 | 26099,23514 |
| Control, 56 days, ABAP DHQ, 28 days, DCFDA | 4330,83752 | 1557,0226 | 2,78149 | 1 | 0,05 | 0 | -1988,12088 | 10649,79592 |
| Control, 56 days, ABAP DHQ, 42 days, DCFDA | 5058,03773 | 1557,0226 | 3,24853 | 0,58536 | 0,05 | 0 | -1260,92067 | 11376,99613 |
| Control, 56 days, ABAP DHQ, 56 days, DCFDA | 20012,26175 | 1557,0226 | 12,8529 | 1,02779E-14 | 0,05 | 1 | 13693,30335 | 26331,22015 |
| Control, 56 days, ABAP Control, 1 day, ABAP | -17534,36436 | 1557,0226 | -11,26147 | 1,24165E-12 | 0,05 | 1 | -23853,32276 | -11215,40595 |
| Control, 56 days, ABAP Control, 7 days, ABAP | -13553,31811 | 1557,0226 | -8,70464 | 5,34736E-9 | 0,05 | 1 | -19872,27651 | -7234,3597 |
| Control, 56 days, ABAP Control, 14 days, ABAP | -5552,25338 | 1557,0226 | -3,56594 | 0,22998 | 0,05 | 0 | -11871,21179 | 766,70502 |
| Control, 56 days, ABAP Control, 28 days, ABAP | -103018,0456 | 1557,0226 | -66,16349 | 2,22937E-46 | 0,05 | 1 | -109337,004 | -96699,0872 |
| Control, 56 days, ABAP Control, 42 days, ABAP | -62961,63838 | 1557,0226 | -40,43722 | 2,683E-36 | 0,05 | 1 | -69280,59679 | -56642,67998 |
| DHQ, 1 day, ABAP Control, 1 day, DCFDA | -5260,11449 | 1557,0226 | -3,37832 | 0,40157 | 0,05 | 0 | -11579,07289 | 1058,84391 |
| DHQ, 1 day, ABAP Control, 7 days, DCFDA | 1648,79051 | 1557,0226 | 1,05894 | 1 | 0,05 | 0 | -4670,16789 | 7967,74891 |
| DHQ, 1 day, ABAP Control, 14 days, DCFDA | -962,20811 | 1557,0226 | -0,61798 | 1 | 0,05 | 0 | -7281,16651 | 5356,7503 |
| DHQ, 1 day, ABAP Control, 28 days, DCFDA | -43310,80199 | 1557,0226 | -27,81642 | 8,09966E-29 | 0,05 | 1 | -49629,76039 | -36991,84359 |
| DHQ, 1 day, ABAP Control, 42 days, DCFDA | -13869,95644 | 1557,0226 | -8,908 | 2,6742E-9 | 0,05 | 1 | -20188,91484 | -7550,99804 |
| DHQ, 1 day, ABAP Control, 56 days, DCFDA | 4794,67786 | 1557,0226 | 3,07939 | 0,9457 | 0,05 | 0 | -1524,28055 | 11113,63626 |
| DHQ, 1 day, ABAP DHQ, 1 day, DCFDA | 4599,88072 | 1557,0226 | 2,95428 | 1 | 0,05 | 0 | -1719,07768 | 10918,83913 |
| DHQ, 1 day, ABAP DHQ, 7 days, DCFDA | -4920,28233 | 1557,0226 | -3,16006 | 0,75356 | 0,05 | 0 | -11239,24073 | 1398,67608 |
| DHQ, 1 day, ABAP DHQ, 14 days, DCFDA | 2583,09535 | 1557,0226 | 1,659 | 1 | 0,05 | 0 | -3735,86305 | 8902,05375 |
| DHQ, 1 day, ABAP DHQ, 28 days, DCFDA | -12866,34387 | 1557,0226 | -8,26343 | 2,43749E-8 | 0,05 | 1 | -19185,30227 | -6547,38547 |
| DHQ, 1 day, ABAP DHQ, 42 days, DCFDA | -12139,14366 | 1557,0226 | -7,79638 | 1,23597E-7 | 0,05 | 1 | -18458,10206 | -5820,18526 |
| DHQ, 1 day, ABAP DHQ, 56 days, DCFDA | 2815,08036 | 1557,0226 | 1,80799 | 1 | 0,05 | 0 | -3503,87804 | 9134,03876 |
| DHQ, 1 day, ABAP Control, 1 day, ABAP | -34731,54574 | 1557,0226 | -22,30639 | 1,529E-24 | 0,05 | 1 | -41050,50414 | -28412,58734 |
| DHQ, 1 day, ABAP Control, 7 days, ABAP | -30750,49949 | 1557,0226 | -19,74955 | 3,00225E-22 | 0,05 | 1 | -37069,45789 | -24431,54109 |
| DHQ, 1 day, ABAP Control, 14 days, ABAP | -22749,43477 | 1557,0226 | -14,61086 | 7,47231E-17 | 0,05 | 1 | -29068,39317 | -16430,47637 |
| DHQ, 1 day, ABAP Control, 28 days, ABAP | -120215,2269 | 1557,0226 | -77,2084 | 1,44276E-49 | 0,05 | 1 | -126534,1853 | -113896,2685 |
| DHQ, 1 day, ABAP Control, 42 days, ABAP | -80158,81977 | 1557,0226 | -51,48212 | 3,21122E-41 | 0,05 | 1 | -86477,77817 | -73839,86137 |
| DHQ, 1 day, ABAP Control, 56 days, ABAP | -17197,18139 | 1557,0226 | -11,04491 | 2,44501E-12 | 0,05 | 1 | -23516,13979 | -10878,22299 |
| DHQ, 7 days, ABAP Control, 1 day, DCFDA | 12861,01517 | 1557,0226 | 8,26001 | 2,46651E-8 | 0,05 | 1 | 6542,05676 | 19179,97357 |
| DHQ, 7 days, ABAP Control, 7 days, DCFDA | 19769,92017 | 1557,0226 | 12,69726 | 1,6192E-14 | 0,05 | 1 | 13450,96176 | 26088,87857 |
| DHQ, 7 days, ABAP Control, 14 days, DCFDA | 17158,92156 | 1557,0226 | 11,02034 | 2,64142E-12 | 0,05 | 1 | 10839,96315 | 23477,87996 |
| DHQ, 7 days, ABAP Control, 28 days, DCFDA | -25189,67233 | 1557,0226 | -16,1781 | 1,26214E-18 | 0,05 | 1 | -31508,63073 | -18870,71393 |
| DHQ, 7 days, ABAP Control, 42 days, DCFDA | 4251,17322 | 1557,0226 | 2,73032 | 1 | 0,05 | 0 | -2067,78518 | 10570,13162 |
| DHQ, 7 days, ABAP Control, 56 days, DCFDA | 22915,80752 | 1557,0226 | 14,71773 | 5,60693E-17 | 0,05 | 1 | 16596,84911 | 29234,76592 |
| DHQ, 7 days, ABAP DHQ, 1 day, DCFDA | 22721,01038 | 1557,0226 | 14,5926 | 7,84916E-17 | 0,05 | 1 | 16402,05198 | 29039,96879 |
| DHQ, 7 days, ABAP DHQ, 7 days, DCFDA | 13200,84733 | 1557,0226 | 8,47826 | 1,16197E-8 | 0,05 | 1 | 6881,88893 | 19519,80574 |
| DHQ, 7 days, ABAP DHQ, 14 days, DCFDA | 20704,22501 | 1557,0226 | 13,29732 | 2,85498E-15 | 0,05 | 1 | 14385,26661 | 27023,18341 |
| DHQ, 7 days, ABAP DHQ, 28 days, DCFDA | 5254,78579 | 1557,0226 | 3,37489 | 0,40562 | 0,05 | 0 | -1064,17261 | 11573,74419 |
| DHQ, 7 days, ABAP DHQ, 42 days, DCFDA | 5981,986 | 1557,0226 | 3,84194 | 0,09881 | 0,05 | 0 | -336,9724 | 12300,9444 |
| DHQ, 7 days, ABAP DHQ, 56 days, DCFDA | 20936,21002 | 1557,0226 | 13,44631 | 1,86859E-15 | 0,05 | 1 | 14617,25162 | 27255,16842 |
| DHQ, 7 days, ABAP Control, 1 day, ABAP | -16610,41608 | 1557,0226 | -10,66806 | 8,06501E-12 | 0,05 | 1 | -22929,37449 | -10291,45768 |
| DHQ, 7 days, ABAP Control, 7 days, ABAP | -12629,36983 | 1557,0226 | -8,11123 | 4,1296E-8 | 0,05 | 1 | -18948,32824 | -6310,41143 |
| DHQ, 7 days, ABAP Control, 14 days, ABAP | -4628,30511 | 1557,0226 | -2,97254 | 1 | 0,05 | 0 | -10947,26351 | 1690,65329 |
| DHQ, 7 days, ABAP Control, 28 days, ABAP | -102094,0973 | 1557,0226 | -65,57008 | 3,4197E-46 | 0,05 | 1 | -108413,0557 | -95775,13893 |
| DHQ, 7 days, ABAP Control, 42 days, ABAP | -62037,69011 | 1557,0226 | -39,8438 | 5,34647E-36 | 0,05 | 1 | -68356,64851 | -55718,73171 |
| DHQ, 7 days, ABAP Control, 56 days, ABAP | 923,94827 | 1557,0226 | 0,59341 | 1 | 0,05 | 0 | -5395,01013 | 7242,90667 |
| DHQ, 7 days, ABAP DHQ, 1 day, ABAP | 18121,12966 | 1557,0226 | 11,63832 | 3,87385E-13 | 0,05 | 1 | 11802,17126 | 24440,08806 |
| DHQ, 14 days, ABAP Control, 1 day, DCFDA | -3732,21206 | 1557,0226 | -2,39702 | 1 | 0,05 | 0 | -10051,17047 | 2586,74634 |
| DHQ, 14 days, ABAP Control, 7 days, DCFDA | 3176,69294 | 1557,0226 | 2,04024 | 1 | 0,05 | 0 | -3142,26547 | 9495,65134 |
| DHQ, 14 days, ABAP Control, 14 days, DCFDA | 565,69433 | 1557,0226 | 0,36332 | 1 | 0,05 | 0 | -5753,26408 | 6884,65273 |
| DHQ, 14 days, ABAP Control, 28 days, DCFDA | -41782,89956 | 1557,0226 | -26,83513 | 4,10032E-28 | 0,05 | 1 | -48101,85797 | -35463,94116 |
| DHQ, 14 days, ABAP Control, 42 days, DCFDA | -12342,05401 | 1557,0226 | -7,9267 | 7,84455E-8 | 0,05 | 1 | -18661,01241 | -6023,09561 |
| DHQ, 14 days, ABAP Control, 56 days, DCFDA | 6322,58028 | 1557,0226 | 4,06069 | 0,04963 | 0,05 | 1 | 3,62188 | 12641,53869 |
| DHQ, 14 days, ABAP DHQ, 1 day, DCFDA | 6127,78315 | 1557,0226 | 3,93558 | 0,07372 | 0,05 | 0 | -191,17525 | 12446,74156 |
| DHQ, 14 days, ABAP DHQ, 7 days, DCFDA | -3392,3799 | 1557,0226 | -2,17876 | 1 | 0,05 | 0 | -9711,3383 | 2926,5785 |
| DHQ, 14 days, ABAP DHQ, 14 days, DCFDA | 4110,99778 | 1557,0226 | 2,64029 | 1 | 0,05 | 0 | -2207,96062 | 10429,95618 |
| DHQ, 14 days, ABAP DHQ, 28 days, DCFDA | -11338,44144 | 1557,0226 | -7,28213 | 7,50629E-7 | 0,05 | 1 | -17657,39984 | -5019,48304 |
| DHQ, 14 days, ABAP DHQ, 42 days, DCFDA | -10611,24123 | 1557,0226 | -6,81508 | 3,89912E-6 | 0,05 | 1 | -16930,19963 | -4292,28283 |
| DHQ, 14 days, ABAP DHQ, 56 days, DCFDA | 4342,98279 | 1557,0226 | 2,78929 | 1 | 0,05 | 0 | -1975,97561 | 10661,94119 |
| DHQ, 14 days, ABAP Control, 1 day, ABAP | -33203,64331 | 1557,0226 | -21,32509 | 1,0914E-23 | 0,05 | 1 | -39522,60172 | -26884,68491 |
| DHQ, 14 days, ABAP Control, 7 days, ABAP | -29222,59706 | 1557,0226 | -18,76825 | 2,63289E-21 | 0,05 | 1 | -35541,55547 | -22903,63866 |
| DHQ, 14 days, ABAP Control, 14 days, ABAP | -21221,53234 | 1557,0226 | -13,62956 | 1,11364E-15 | 0,05 | 1 | -27540,49074 | -14902,57394 |
| DHQ, 14 days, ABAP Control, 28 days, ABAP | -118687,3245 | 1557,0226 | -76,2277 | 2,65307E-49 | 0,05 | 1 | -125006,2829 | -112368,3661 |
| DHQ, 14 days, ABAP Control, 42 days, ABAP | -78630,91734 | 1557,0226 | -50,50082 | 7,95681E-41 | 0,05 | 1 | -84949,87574 | -72311,95894 |
| DHQ, 14 days, ABAP Control, 56 days, ABAP | -15669,27896 | 1557,0226 | -10,06362 | 5,67785E-11 | 0,05 | 1 | -21988,23736 | -9350,32056 |
| DHQ, 14 days, ABAP DHQ, 1 day, ABAP | 1527,90243 | 1557,0226 | 0,9813 | 1 | 0,05 | 0 | -4791,05597 | 7846,86083 |
| DHQ, 14 days, ABAP DHQ, 7 days, ABAP | -16593,22723 | 1557,0226 | -10,65702 | 8,35425E-12 | 0,05 | 1 | -22912,18563 | -10274,26883 |
| DHQ, 28 days, ABAP Control, 1 day, DCFDA | 11859,33313 | 1557,0226 | 7,61667 | 2,31772E-7 | 0,05 | 1 | 5540,37472 | 18178,29153 |
| DHQ, 28 days, ABAP Control, 7 days, DCFDA | 18768,23813 | 1557,0226 | 12,05393 | 1,09522E-13 | 0,05 | 1 | 12449,27972 | 25087,19653 |
| DHQ, 28 days, ABAP Control, 14 days, DCFDA | 16157,23951 | 1557,0226 | 10,37701 | 2,05242E-11 | 0,05 | 1 | 9838,28111 | 22476,19792 |
| DHQ, 28 days, ABAP Control, 28 days, DCFDA | -26191,35437 | 1557,0226 | -16,82143 | 2,55673E-19 | 0,05 | 1 | -32510,31278 | -19872,39597 |
| DHQ, 28 days, ABAP Control, 42 days, DCFDA | 3249,49118 | 1557,0226 | 2,08699 | 1 | 0,05 | 0 | -3069,46722 | 9568,44958 |
| DHQ, 28 days, ABAP Control, 56 days, DCFDA | 21914,12547 | 1557,0226 | 14,07438 | 3,22563E-16 | 0,05 | 1 | 15595,16707 | 28233,08388 |
| DHQ, 28 days, ABAP DHQ, 1 day, DCFDA | 21719,32834 | 1557,0226 | 13,94927 | 4,55937E-16 | 0,05 | 1 | 15400,36994 | 28038,28674 |
| DHQ, 28 days, ABAP DHQ, 7 days, DCFDA | 12199,16529 | 1557,0226 | 7,83493 | 1,08032E-7 | 0,05 | 1 | 5880,20689 | 18518,12369 |
| DHQ, 28 days, ABAP DHQ, 14 days, DCFDA | 19702,54297 | 1557,0226 | 12,65399 | 1,83832E-14 | 0,05 | 1 | 13383,58456 | 26021,50137 |
| DHQ, 28 days, ABAP DHQ, 28 days, DCFDA | 4253,10375 | 1557,0226 | 2,73156 | 1 | 0,05 | 0 | -2065,85465 | 10572,06215 |
| DHQ, 28 days, ABAP DHQ, 42 days, DCFDA | 4980,30396 | 1557,0226 | 3,19861 | 0,67532 | 0,05 | 0 | -1338,65444 | 11299,26236 |
| DHQ, 28 days, ABAP DHQ, 56 days, DCFDA | 19934,52798 | 1557,0226 | 12,80298 | 1,18871E-14 | 0,05 | 1 | 13615,56958 | 26253,48638 |
| DHQ, 28 days, ABAP Control, 1 day, ABAP | -17612,09812 | 1557,0226 | -11,3114 | 1,06298E-12 | 0,05 | 1 | -23931,05653 | -11293,13972 |
| DHQ, 28 days, ABAP Control, 7 days, ABAP | -13631,05187 | 1557,0226 | -8,75456 | 4,50913E-9 | 0,05 | 1 | -19950,01028 | -7312,09347 |
| DHQ, 28 days, ABAP Control, 14 days, ABAP | -5629,98715 | 1557,0226 | -3,61587 | 0,1978 | 0,05 | 0 | -11948,94555 | 688,97125 |
| DHQ, 28 days, ABAP Control, 28 days, ABAP | -103095,7793 | 1557,0226 | -66,21341 | 2,15092E-46 | 0,05 | 1 | -109414,7377 | -96776,82097 |
| DHQ, 28 days, ABAP Control, 42 days, ABAP | -63039,37215 | 1557,0226 | -40,48713 | 2,53289E-36 | 0,05 | 1 | -69358,33055 | -56720,41375 |
| DHQ, 28 days, ABAP Control, 56 days, ABAP | -77,73377 | 1557,0226 | -0,04992 | 1 | 0,05 | 0 | -6396,69217 | 6241,22463 |
| DHQ, 28 days, ABAP DHQ, 1 day, ABAP | 17119,44762 | 1557,0226 | 10,99499 | 2,86085E-12 | 0,05 | 1 | 10800,48922 | 23438,40602 |
| DHQ, 28 days, ABAP DHQ, 7 days, ABAP | -1001,68204 | 1557,0226 | -0,64333 | 1 | 0,05 | 0 | -7320,64044 | 5317,27636 |
| DHQ, 28 days, ABAP DHQ, 14 days, ABAP | 15591,54519 | 1557,0226 | 10,01369 | 6,68445E-11 | 0,05 | 1 | 9272,58679 | 21910,50359 |
| DHQ, 42 days, ABAP Control, 1 day, DCFDA | 6569,9125 | 1557,0226 | 4,21954 | 0,02982 | 0,05 | 1 | 250,9541 | 12888,8709 |
| DHQ, 42 days, ABAP Control, 7 days, DCFDA | 13478,8175 | 1557,0226 | 8,65679 | 6,29804E-9 | 0,05 | 1 | 7159,8591 | 19797,7759 |
| DHQ, 42 days, ABAP Control, 14 days, DCFDA | 10867,81889 | 1557,0226 | 6,97987 | 2,17889E-6 | 0,05 | 1 | 4548,86049 | 17186,77729 |
| DHQ, 42 days, ABAP Control, 28 days, DCFDA | -31480,775 | 1557,0226 | -20,21857 | 1,09555E-22 | 0,05 | 1 | -37799,7334 | -25161,8166 |
| DHQ, 42 days, ABAP Control, 42 days, DCFDA | -2039,92944 | 1557,0226 | -1,31015 | 1 | 0,05 | 0 | -8358,88785 | 4279,02896 |
| DHQ, 42 days, ABAP Control, 56 days, DCFDA | 16624,70485 | 1557,0226 | 10,67724 | 7,83231E-12 | 0,05 | 1 | 10305,74645 | 22943,66325 |
| DHQ, 42 days, ABAP DHQ, 1 day, DCFDA | 16429,90772 | 1557,0226 | 10,55213 | 1,16851E-11 | 0,05 | 1 | 10110,94932 | 22748,86612 |
| DHQ, 42 days, ABAP DHQ, 7 days, DCFDA | 6909,74467 | 1557,0226 | 4,43779 | 0,01464 | 0,05 | 1 | 590,78626 | 13228,70307 |
| DHQ, 42 days, ABAP DHQ, 14 days, DCFDA | 14413,12234 | 1557,0226 | 9,25685 | 8,22782E-10 | 0,05 | 1 | 8094,16394 | 20732,08074 |
| DHQ, 42 days, ABAP DHQ, 28 days, DCFDA | -1036,31688 | 1557,0226 | -0,66558 | 1 | 0,05 | 0 | -7355,27528 | 5282,64153 |
| DHQ, 42 days, ABAP DHQ, 42 days, DCFDA | -309,11667 | 1557,0226 | -0,19853 | 1 | 0,05 | 0 | -6628,07507 | 6009,84174 |
| DHQ, 42 days, ABAP DHQ, 56 days, DCFDA | 14645,10735 | 1557,0226 | 9,40584 | 4,99344E-10 | 0,05 | 1 | 8326,14895 | 20964,06575 |
| DHQ, 42 days, ABAP Control, 1 day, ABAP | -22901,51875 | 1557,0226 | -14,70853 | 5,74665E-17 | 0,05 | 1 | -29220,47715 | -16582,56035 |
| DHQ, 42 days, ABAP Control, 7 days, ABAP | -18920,4725 | 1557,0226 | -12,1517 | 8,16282E-14 | 0,05 | 1 | -25239,4309 | -12601,5141 |
| DHQ, 42 days, ABAP Control, 14 days, ABAP | -10919,40778 | 1557,0226 | -7,01301 | 1,93841E-6 | 0,05 | 1 | -17238,36618 | -4600,44938 |
| DHQ, 42 days, ABAP Control, 28 days, ABAP | -108385,2 | 1557,0226 | -69,61055 | 1,9957E-47 | 0,05 | 1 | -114704,1584 | -102066,2416 |
| DHQ, 42 days, ABAP Control, 42 days, ABAP | -68328,79278 | 1557,0226 | -43,88427 | 5,85213E-38 | 0,05 | 1 | -74647,75118 | -62009,83438 |
| DHQ, 42 days, ABAP Control, 56 days, ABAP | -5367,15439 | 1557,0226 | -3,44706 | 0,32794 | 0,05 | 0 | -11686,1128 | 951,80401 |
| DHQ, 42 days, ABAP DHQ, 1 day, ABAP | 11830,02699 | 1557,0226 | 7,59785 | 2,47575E-7 | 0,05 | 1 | 5511,06859 | 18148,98539 |
| DHQ, 42 days, ABAP DHQ, 7 days, ABAP | -6291,10267 | 1557,0226 | -4,04047 | 0,05293 | 0,05 | 0 | -12610,06107 | 27,85574 |
| DHQ, 42 days, ABAP DHQ, 14 days, ABAP | 10302,12456 | 1557,0226 | 6,61655 | 7,86341E-6 | 0,05 | 1 | 3983,16616 | 16621,08297 |
| DHQ, 42 days, ABAP DHQ, 28 days, ABAP | -5289,42063 | 1557,0226 | -3,39714 | 0,37998 | 0,05 | 0 | -11608,37903 | 1029,53778 |
| DHQ, 56 days, ABAP Control, 1 day, DCFDA | -2934,4674 | 1557,0226 | -1,88467 | 1 | 0,05 | 0 | -9253,4258 | 3384,491 |
| DHQ, 56 days, ABAP Control, 7 days, DCFDA | 3974,4376 | 1557,0226 | 2,55259 | 1 | 0,05 | 0 | -2344,5208 | 10293,396 |
| DHQ, 56 days, ABAP Control, 14 days, DCFDA | 1363,43899 | 1557,0226 | 0,87567 | 1 | 0,05 | 0 | -4955,51941 | 7682,39739 |
| DHQ, 56 days, ABAP Control, 28 days, DCFDA | -40985,1549 | 1557,0226 | -26,32277 | 9,7637E-28 | 0,05 | 1 | -47304,1133 | -34666,1965 |
| DHQ, 56 days, ABAP Control, 42 days, DCFDA | -11544,30935 | 1557,0226 | -7,41435 | 4,7145E-7 | 0,05 | 1 | -17863,26775 | -5225,35094 |
| DHQ, 56 days, ABAP Control, 56 days, DCFDA | 7120,32495 | 1557,0226 | 4,57304 | 0,00936 | 0,05 | 1 | 801,36654 | 13439,28335 |
| DHQ, 56 days, ABAP DHQ, 1 day, DCFDA | 6925,52782 | 1557,0226 | 4,44793 | 0,01416 | 0,05 | 1 | 606,56941 | 13244,48622 |
| DHQ, 56 days, ABAP DHQ, 7 days, DCFDA | -2594,63524 | 1557,0226 | -1,66641 | 1 | 0,05 | 0 | -8913,59364 | 3724,32317 |
| DHQ, 56 days, ABAP DHQ, 14 days, DCFDA | 4908,74244 | 1557,0226 | 3,15265 | 0,76954 | 0,05 | 0 | -1410,21596 | 11227,70084 |
| DHQ, 56 days, ABAP DHQ, 28 days, DCFDA | -10540,69678 | 1557,0226 | -6,76978 | 4,57597E-6 | 0,05 | 1 | -16859,65518 | -4221,73838 |
| DHQ, 56 days, ABAP DHQ, 42 days, DCFDA | -9813,49657 | 1557,0226 | -6,30273 | 2,38202E-5 | 0,05 | 1 | -16132,45497 | -3494,53817 |
| DHQ, 56 days, ABAP DHQ, 56 days, DCFDA | 5140,72745 | 1557,0226 | 3,30164 | 0,50216 | 0,05 | 0 | -1178,23095 | 11459,68585 |
| DHQ, 56 days, ABAP Control, 1 day, ABAP | -32405,89865 | 1557,0226 | -20,81273 | 3,13653E-23 | 0,05 | 1 | -38724,85705 | -26086,94025 |
| DHQ, 56 days, ABAP Control, 7 days, ABAP | -28424,8524 | 1557,0226 | -18,2559 | 8,47087E-21 | 0,05 | 1 | -34743,8108 | -22105,894 |
| DHQ, 56 days, ABAP Control, 14 days, ABAP | -20423,78768 | 1557,0226 | -13,11721 | 4,7836E-15 | 0,05 | 1 | -26742,74608 | -14104,82928 |
| DHQ, 56 days, ABAP Control, 28 days, ABAP | -117889,5799 | 1557,0226 | -75,71475 | 3,65774E-49 | 0,05 | 1 | -124208,5383 | -111570,6215 |
| DHQ, 56 days, ABAP Control, 42 days, ABAP | -77833,17268 | 1557,0226 | -49,98847 | 1,28657E-40 | 0,05 | 1 | -84152,13108 | -71514,21428 |
| DHQ, 56 days, ABAP Control, 56 days, ABAP | -14871,5343 | 1557,0226 | -9,55126 | 3,07439E-10 | 0,05 | 1 | -21190,4927 | -8552,57589 |
| DHQ, 56 days, ABAP DHQ, 1 day, ABAP | 2325,64709 | 1557,0226 | 1,49365 | 1 | 0,05 | 0 | -3993,31131 | 8644,60549 |
| DHQ, 56 days, ABAP DHQ, 7 days, ABAP | -15795,48257 | 1557,0226 | -10,14467 | 4,35899E-11 | 0,05 | 1 | -22114,44097 | -9476,52417 |
| DHQ, 56 days, ABAP DHQ, 14 days, ABAP | 797,74466 | 1557,0226 | 0,51235 | 1 | 0,05 | 0 | -5521,21374 | 7116,70306 |
| DHQ, 56 days, ABAP DHQ, 28 days, ABAP | -14793,80053 | 1557,0226 | -9,50134 | 3,63041E-10 | 0,05 | 1 | -21112,75893 | -8474,84213 |
| DHQ, 56 days, ABAP DHQ, 42 days, ABAP | -9504,3799 | 1557,0226 | -6,1042 | 4,7961E-5 | 0,05 | 1 | -15823,3383 | -3185,4215 |

*Scheffe Test*

| | MeanDiff | SEM | F Value | Prob | Alpha | Sig | LCL | UCL |
|---|---|---|---|---|---|---|---|---|
| Control, 7 days, DCFDA Control, 1 day, DCFDA | -6908,905 | 1557,0226 | 0,85605 | 0,64944 | 0,05 | 0 | -16806,16487 | 2988,35487 |
| Control, 14 days, DCFDA Control, 1 day, DCFDA | -4297,90639 | 1557,0226 | 0,33128 | 0,99728 | 0,05 | 0 | -14195,16626 | 5599,35348 |
| Control, 14 days, DCFDA Control, 7 days, DCFDA | 2610,99861 | 1557,0226 | 0,12226 | 1 | 0,05 | 0 | -7286,26126 | 12508,25848 |
| Control, 28 days, DCFDA Control, 1 day, DCFDA | 38050,6875 | 1557,0226 | 25,96614 | 8,94383E-20 | 0,05 | 1 | 28153,42763 | 47947,94737 |
| Control, 28 days, DCFDA Control, 7 days, DCFDA | 44959,5925 | 1557,0226 | 36,25159 | 6,14947E-23 | 0,05 | 1 | 35062,33263 | 54856,85237 |
| Control, 28 days, DCFDA Control, 14 days, DCFDA | 42348,59389 | 1557,0226 | 32,16328 | 8,59179E-22 | 0,05 | 1 | 32451,33402 | 52245,85376 |
| Control, 42 days, DCFDA Control, 1 day, DCFDA | 8609,84194 | 1557,0226 | 1,32945 | 0,19975 | 0,05 | 0 | -1287,41793 | 18507,10182 |
| Control, 42 days, DCFDA Control, 7 days, DCFDA | 15518,74694 | 1557,0226 | 4,31912 | 9,55272E-6 | 0,05 | 1 | 5621,48707 | 25416,00682 |
| Control, 42 days, DCFDA Control, 14 days, DCFDA | 12907,74833 | 1557,0226 | 2,98802 | 6,87503E-4 | 0,05 | 1 | 3010,48846 | 22805,00821 |
| Control, 42 days, DCFDA Control, 28 days, DCFDA | -29440,84556 | 1557,0226 | 15,54472 | 3,80397E-15 | 0,05 | 1 | -39338,10543 | -19543,58568 |
| Control, 56 days, DCFDA Control, 1 day, DCFDA | -10054,79235 | 1557,0226 | 1,81313 | 0,04122 | 0,05 | 1 | -19952,05222 | -157,53247 |
| Control, 56 days, DCFDA Control, 7 days, DCFDA | -3145,88735 | 1557,0226 | 0,17749 | 0,99998 | 0,05 | 0 | -13043,14722 | 6751,37253 |
| Control, 56 days, DCFDA Control, 14 days, DCFDA | -5756,88596 | 1557,0226 | 0,59437 | 0,9113 | 0,05 | 0 | -15654,14583 | 4140,37391 |
| Control, 56 days, DCFDA Control, 28 days, DCFDA | -48105,47985 | 1557,0226 | 41,50223 | 3,02806E-24 | 0,05 | 1 | -58002,73972 | -38208,21997 |
| Control, 56 days, DCFDA Control, 42 days, DCFDA | -18664,63429 | 1557,0226 | 6,24772 | 4,89088E-8 | 0,05 | 1 | -28561,89417 | -8767,37442 |
| DHQ, 1 day, DCFDA Control, 1 day, DCFDA | -9859,99522 | 1557,0226 | 1,74356 | 0,0523 | 0,05 | 0 | -19757,25509 | 37,26466 |
| DHQ, 1 day, DCFDA Control, 7 days, DCFDA | -2951,09022 | 1557,0226 | 0,15619 | 0,99999 | 0,05 | 0 | -12848,35009 | 6946,16966 |
| DHQ, 1 day, DCFDA Control, 14 days, DCFDA | -5562,08883 | 1557,0226 | 0,55483 | 0,93636 | 0,05 | 0 | -15459,3487 | 4335,17105 |
| DHQ, 1 day, DCFDA Control, 28 days, DCFDA | -47910,68272 | 1557,0226 | 41,16679 | 3,63063E-24 | 0,05 | 1 | -57807,94259 | -38013,42284 |
| DHQ, 1 day, DCFDA Control, 42 days, DCFDA | -18469,83716 | 1557,0226 | 6,11799 | 6,76375E-8 | 0,05 | 1 | -28367,09704 | -8572,57729 |
| DHQ, 1 day, DCFDA Control, 56 days, DCFDA | 194,79713 | 1557,0226 | 6,8053E-4 | 1 | 0,05 | 0 | -9702,46274 | 10092,057 |
| DHQ, 7 days, DCFDA Control, 1 day, DCFDA | -339,83217 | 1557,0226 | 0,00207 | 1 | 0,05 | 0 | -10237,09204 | 9557,42771 |
| DHQ, 7 days, DCFDA Control, 7 days, DCFDA | 6569,07283 | 1557,0226 | 0,77391 | 0,74401 | 0,05 | 0 | -3328,18704 | 16466,33271 |
| DHQ, 7 days, DCFDA Control, 14 days, DCFDA | 3958,07422 | 1557,0226 | 0,28096 | 0,9992 | 0,05 | 0 | -5939,18565 | 13855,3341 |
| DHQ, 7 days, DCFDA Control, 28 days, DCFDA | -38390,51967 | 1557,0226 | 26,43202 | 6,10333E-20 | 0,05 | 1 | -48287,77954 | -28493,25979 |
| DHQ, 7 days, DCFDA Control, 42 days, DCFDA | -8949,67411 | 1557,0226 | 1,43647 | 0,14371 | 0,05 | 0 | -18846,93398 | 947,58576 |
| DHQ, 7 days, DCFDA Control, 56 days, DCFDA | 9714,96018 | 1557,0226 | 1,69264 | 0,06215 | 0,05 | 0 | -182,29969 | 19612,22006 |
| DHQ, 7 days, DCFDA DHQ, 1 day, DCFDA | 9520,16305 | 1557,0226 | 1,62544 | 0,07785 | 0,05 | 0 | -377,09682 | 19417,42292 |
| DHQ, 14 days, DCFDA Control, 1 day, DCFDA | -7843,20984 | 1557,0226 | 1,10324 | 0,37611 | 0,05 | 0 | -17740,46971 | 2054,05003 |
| DHQ, 14 days, DCFDA Control, 7 days, DCFDA | -934,30484 | 1557,0226 | 0,01566 | 1 | 0,05 | 0 | -10831,56471 | 8962,95503 |
| DHQ, 14 days, DCFDA Control, 14 days, DCFDA | -3545,30345 | 1557,0226 | 0,22542 | 0,99986 | 0,05 | 0 | -13442,56333 | 6351,95642 |
| DHQ, 14 days, DCFDA Control, 28 days, DCFDA | -45893,89734 | 1557,0226 | 37,77393 | 2,46916E-23 | 0,05 | 1 | -55791,15721 | -35996,63747 |
| DHQ, 14 days, DCFDA Control, 42 days, DCFDA | -16453,05179 | 1557,0226 | 4,85484 | 1,98945E-6 | 0,05 | 1 | -26350,31166 | -6555,79191 |
| DHQ, 14 days, DCFDA Control, 56 days, DCFDA | 2211,58251 | 1557,0226 | 0,08772 | 1 | 0,05 | 0 | -7685,67737 | 12108,84238 |
| DHQ, 14 days, DCFDA DHQ, 1 day, DCFDA | 2016,78538 | 1557,0226 | 0,07295 | 1 | 0,05 | 0 | -7880,4745 | 11914,04525 |
| DHQ, 14 days, DCFDA DHQ, 7 days, DCFDA | -7503,37767 | 1557,0226 | 1,00971 | 0,47248 | 0,05 | 0 | -17400,63755 | 2393,8822 |
| DHQ, 28 days, DCFDA Control, 1 day, DCFDA | 7606,22938 | 1557,0226 | 1,03758 | 0,44252 | 0,05 | 0 | -2291,0305 | 17503,48925 |
| DHQ, 28 days, DCFDA Control, 7 days, DCFDA | 14515,13438 | 1557,0226 | 3,77854 | 5,08319E-5 | 0,05 | 1 | 4617,8745 | 24412,39425 |
| DHQ, 28 days, DCFDA Control, 14 days, DCFDA | 11904,13576 | 1557,0226 | 2,54143 | 0,00322 | 0,05 | 1 | 2006,87589 | 21801,39564 |
| DHQ, 28 days, DCFDA Control, 28 days, DCFDA | -30444,45812 | 1557,0226 | 16,6226 | 9,89107E-16 | 0,05 | 1 | -40341,718 | -20547,19825 |
| DHQ, 28 days, DCFDA Control, 42 days, DCFDA | -1003,61257 | 1557,0226 | 0,01806 | 1 | 0,05 | 0 | -10900,87244 | 8893,6473 |
| DHQ, 28 days, DCFDA Control, 56 days, DCFDA | 17661,02172 | 1557,0226 | 5,59389 | 2,61402E-7 | 0,05 | 1 | 7763,76185 | 27558,2816 |
| DHQ, 28 days, DCFDA DHQ, 1 day, DCFDA | 17466,22459 | 1557,0226 | 5,47117 | 3,62407E-7 | 0,05 | 1 | 7568,96472 | 27363,48447 |
| DHQ, 28 days, DCFDA DHQ, 7 days, DCFDA | 7946,06154 | 1557,0226 | 1,13236 | 0,34874 | 0,05 | 0 | -1951,19833 | 17843,32142 |
| DHQ, 28 days, DCFDA DHQ, 14 days, DCFDA | 15449,43922 | 1557,0226 | 4,28063 | 1,07283E-5 | 0,05 | 1 | 5552,17934 | 25346,69909 |
| DHQ, 42 days, DCFDA Control, 1 day, DCFDA | 6879,02917 | 1557,0226 | 0,84867 | 0,65811 | 0,05 | 0 | -3018,23071 | 16776,28904 |
| DHQ, 42 days, DCFDA Control, 7 days, DCFDA | 13787,93417 | 1557,0226 | 3,40942 | 1,67634E-4 | 0,05 | 1 | 3890,67429 | 23685,19404 |
| DHQ, 42 days, DCFDA Control, 14 days, DCFDA | 11176,93556 | 1557,0226 | 2,24041 | 0,00927 | 0,05 | 1 | 1279,67568 | 21074,19543 |
| DHQ, 42 days, DCFDA Control, 28 days, DCFDA | -31171,65833 | 1557,0226 | 17,42618 | 3,79608E-16 | 0,05 | 1 | -41068,91821 | -21274,39846 |
| DHQ, 42 days, DCFDA Control, 42 days, DCFDA | -1730,81278 | 1557,0226 | 0,05373 | 1 | 0,05 | 0 | -11628,07265 | 8166,4471 |
| DHQ, 42 days, DCFDA Control, 56 days, DCFDA | 16933,82152 | 1557,0226 | 5,14271 | 8,86355E-7 | 0,05 | 1 | 7036,56164 | 26831,08139 |
| DHQ, 42 days, DCFDA DHQ, 1 day, DCFDA | 16739,02438 | 1557,0226 | 5,02508 | 1,22987E-6 | 0,05 | 1 | 6841,76451 | 26636,28426 |
| DHQ, 42 days, DCFDA DHQ, 7 days, DCFDA | 7218,86133 | 1557,0226 | 0,93459 | 0,55733 | 0,05 | 0 | -2678,39854 | 17116,12121 |
| DHQ, 42 days, DCFDA DHQ, 14 days, DCFDA | 14722,23901 | 1557,0226 | 3,88714 | 3,60694E-5 | 0,05 | 1 | 4824,97913 | 24619,49888 |
| DHQ, 42 days, DCFDA DHQ, 28 days, DCFDA | -727,20021 | 1557,0226 | 0,00948 | 1 | 0,05 | 0 | -10624,46008 | 9170,05967 |
| DHQ, 56 days, DCFDA Control, 1 day, DCFDA | -8075,19485 | 1557,0226 | 1,16947 | 0,31585 | 0,05 | 0 | -17972,45473 | 1822,06502 |
| DHQ, 56 days, DCFDA Control, 7 days, DCFDA | -1166,28985 | 1557,0226 | 0,02439 | 1 | 0,05 | 0 | -11063,54973 | 8730,97002 |
| DHQ, 56 days, DCFDA Control, 14 days, DCFDA | -3777,28846 | 1557,0226 | 0,25588 | 0,99962 | 0,05 | 0 | -13674,54834 | 6119,97141 |
| DHQ, 56 days, DCFDA Control, 28 days, DCFDA | -46125,88235 | 1557,0226 | 38,15678 | 1,97338E-23 | 0,05 | 1 | -56023,14223 | -36228,62248 |
| DHQ, 56 days, DCFDA Control, 42 days, DCFDA | -16685,0368 | 1557,0226 | 4,99271 | 1,34676E-6 | 0,05 | 1 | -26582,29667 | -6787,77692 |
| DHQ, 56 days, DCFDA Control, 56 days, DCFDA | 1979,5975 | 1557,0226 | 0,07028 | 1 | 0,05 | 0 | -7917,66238 | 11876,85737 |
| DHQ, 56 days, DCFDA DHQ, 1 day, DCFDA | 1784,80036 | 1557,0226 | 0,05713 | 1 | 0,05 | 0 | -8112,45951 | 11682,06024 |
| DHQ, 56 days, DCFDA DHQ, 7 days, DCFDA | -7735,36269 | 1557,0226 | 1,07311 | 0,40581 | 0,05 | 0 | -17632,62256 | 2161,89719 |
| DHQ, 56 days, DCFDA DHQ, 14 days, DCFDA | -231,98501 | 1557,0226 | 9,65166E- | 1 | 0,05 | 0 | -10129,24489 | 9665,27486 |
| DHQ, 56 days, DCFDA DHQ, 28 days, DCFDA | -15681,42423 | 1557,0226 | 4,41015 | 7,27304E-6 | 0,05 | 1 | -25578,6841 | -5784,16435 |
| DHQ, 56 days, DCFDA DHQ, 42 days, DCFDA | -14954,22402 | 1557,0226 | 4,01061 | 2,45281E-5 | 0,05 | 1 | -24851,48389 | -5056,96415 |
| Control, 1 day, ABAP Control, 1 day, DCFDA | 29471,43125 | 1557,0226 | 15,57703 | 3,64937E-15 | 0,05 | 1 | 19574,17138 | 39368,69112 |
| Control, 1 day, ABAP Control, 7 days, DCFDA | 36380,33625 | 1557,0226 | 23,73645 | 6,08689E-19 | 0,05 | 1 | 26483,07638 | 46277,59612 |
| Control, 1 day, ABAP Control, 14 days, DCFDA | 33769,33764 | 1557,0226 | 20,45161 | 1,40066E-17 | 0,05 | 1 | 23872,07777 | 43666,59751 |
| Control, 1 day, ABAP Control, 28 days, DCFDA | -8579,25625 | 1557,0226 | 1,32002 | 0,20547 | 0,05 | 0 | -18476,51612 | 1318,00362 |
| Control, 1 day, ABAP Control, 42 days, DCFDA | 20861,58931 | 1557,0226 | 7,80508 | 1,33112E-9 | 0,05 | 1 | 10964,32943 | 30758,84918 |
| Control, 1 day, ABAP Control, 56 days, DCFDA | 39526,2236 | 1557,0226 | 28,01902 | 1,73468E-20 | 0,05 | 1 | 29628,96372 | 49423,48347 |
| Control, 1 day, ABAP DHQ, 1 day, DCFDA | 39331,42647 | 1557,0226 | 27,74353 | 2,14801E-20 | 0,05 | 1 | 29434,16659 | 49228,68634 |
| Control, 1 day, ABAP DHQ, 7 days, DCFDA | 29811,26342 | 1557,0226 | 15,93834 | 2,30582E-15 | 0,05 | 1 | 19914,00354 | 39708,52329 |
| Control, 1 day, ABAP DHQ, 14 days, DCFDA | 37314,64109 | 1557,0226 | 24,97128 | 2,06527E-19 | 0,05 | 1 | 27417,38122 | 47211,90096 |
| Control, 1 day, ABAP DHQ, 28 days, DCFDA | 21865,20187 | 1557,0226 | 8,57412 | 2,66825E-10 | 0,05 | 1 | 11967,942 | 31762,46175 |
| Control, 1 day, ABAP DHQ, 42 days, DCFDA | 22592,40208 | 1557,0226 | 9,15392 | 8,47439E-11 | 0,05 | 1 | 12695,14221 | 32489,66196 |
| Control, 1 day, ABAP DHQ, 56 days, DCFDA | 37546,6261 | 1557,0226 | 25,28274 | 1,58426E-19 | 0,05 | 1 | 27649,36623 | 47443,88598 |
| Control, 7 days, ABAP Control, 1 day, DCFDA | 25490,385 | 1557,0226 | 11,65293 | 1,0268E-12 | 0,05 | 1 | 15593,12513 | 35387,64487 |
| Control, 7 days, ABAP Control, 7 days, DCFDA | 32399,29 | 1557,0226 | 18,82579 | 7,79895E-17 | 0,05 | 1 | 22502,03013 | 42296,54987 |
| Control, 7 days, ABAP Control, 14 days, DCFDA | 29788,29139 | 1557,0226 | 15,91379 | 2,37824E-15 | 0,05 | 1 | 19891,03152 | 39685,55126 |
| Control, 7 days, ABAP Control, 28 days, DCFDA | -12560,3025 | 1557,0226 | 2,82932 | 0,00118 | 0,05 | 1 | -22457,56227 | -2663,04263 |
| Control, 7 days, ABAP Control, 42 days, DCFDA | 16880,54306 | 1557,0226 | 5,1104 | 9,69416E-7 | 0,05 | 1 | 6983,28318 | 26777,80293 |
| Control, 7 days, ABAP Control, 56 days, DCFDA | 35545,17735 | 1557,0226 | 22,65915 | 1,62873E-18 | 0,05 | 1 | 25647,91747 | 45442,43722 |
| Control, 7 days, ABAP DHQ, 1 day, DCFDA | 35350,38022 | 1557,0226 | 22,41148 | 2,05418E-18 | 0,05 | 1 | 25453,12034 | 45247,64009 |
| Control, 7 days, ABAP DHQ, 7 days, DCFDA | 25830,21717 | 1557,0226 | 11,96571 | 6,22459E-13 | 0,05 | 1 | 15932,95729 | 35727,47704 |
| Control, 7 days, ABAP DHQ, 14 days, DCFDA | 33333,59484 | 1557,0226 | 19,92722 | 2,40499E-17 | 0,05 | 1 | 23436,33497 | 43230,85471 |
| Control, 7 days, ABAP DHQ, 28 days, DCFDA | 17884,15562 | 1557,0226 | 5,73613 | 1,7988E-7 | 0,05 | 1 | 7986,89575 | 27781,4155 |
| Control, 7 days, ABAP DHQ, 42 days, DCFDA | 18611,35583 | 1557,0226 | 6,2121 | 5,34405E-8 | 0,05 | 1 | 8714,09596 | 28508,61571 |
| Control, 7 days, ABAP DHQ, 56 days, DCFDA | 33565,57985 | 1557,0226 | 20,20555 | 1,80237E-17 | 0,05 | 1 | 23668,31998 | 43462,83973 |
| Control, 7 days, ABAP Control, 1 day, ABAP | -3981,04625 | 1557,0226 | 0,28423 | 0,99912 | 0,05 | 0 | -13878,30612 | 5916,21362 |
| Control, 14 days, ABAP Control, 1 day, DCFDA | 17489,32028 | 1557,0226 | 5,48565 | 3,48631E-7 | 0,05 | 1 | 7592,0604 | 27386,58015 |
| Control, 14 days, ABAP Control, 7 days, DCFDA | 24398,22528 | 1557,0226 | 10,67576 | 5,2561E-12 | 0,05 | 1 | 14500,9654 | 34295,48515 |
| Control, 14 days, ABAP Control, 14 days, DCFDA | 21787,22667 | 1557,0226 | 8,51307 | 3,02014E-10 | 0,05 | 1 | 11889,96679 | 31684,48654 |
| Control, 14 days, ABAP Control, 28 days, DCFDA | -20561,36722 | 1557,0226 | 7,58204 | 2,16402E-9 | 0,05 | 1 | -30458,6271 | -10664,10735 |
| Control, 14 days, ABAP Control, 42 days, DCFDA | 8879,47833 | 1557,0226 | 1,41403 | 0,15418 | 0,05 | 0 | -1017,78154 | 18776,73821 |
| Control, 14 days, ABAP Control, 56 days, DCFDA | 27544,11263 | 1557,0226 | 13,6063 | 5,26705E-14 | 0,05 | 1 | 17646,85275 | 37441,3725 |
| Control, 14 days, ABAP DHQ, 1 day, DCFDA | 27349,3155 | 1557,0226 | 13,41452 | 6,94194E-14 | 0,05 | 1 | 17452,05562 | 37246,57537 |
| Control, 14 days, ABAP DHQ, 7 days, DCFDA | 17829,15244 | 1557,0226 | 5,7009 | 1,97231E-7 | 0,05 | 1 | 7931,89257 | 27726,41232 |
| Control, 14 days, ABAP DHQ, 14 days, DCFDA | 25332,53012 | 1557,0226 | 11,50905 | 1,29715E-12 | 0,05 | 1 | 15435,27025 | 35229,78999 |
| Control, 14 days, ABAP DHQ, 28 days, DCFDA | 9883,0909 | 1557,0226 | 1,75173 | 0,05086 | 0,05 | 0 | -14,16897 | 19780,35078 |
| Control, 14 days, ABAP DHQ, 42 days, DCFDA | 10610,29111 | 1557,0226 | 2,019 | 0,02017 | 0,05 | 1 | 713,03124 | 20507,55098 |
| Control, 14 days, ABAP DHQ, 56 days, DCFDA | 25564,51513 | 1557,0226 | 11,72081 | 9,20312E-13 | 0,05 | 1 | 15667,25526 | 35461,775 |
| Control, 14 days, ABAP Control, 1 day, ABAP | -11982,11097 | 1557,0226 | 2,57483 | 0,00286 | 0,05 | 1 | -21879,37085 | -2084,8511 |
| Control, 14 days, ABAP Control, 7 days, ABAP | -8001,06472 | 1557,0226 | 1,1481 | 0,33452 | 0,05 | 0 | -17898,3246 | 1896,19515 |
| Control, 28 days, ABAP Control, 1 day, DCFDA | 114955,1125 | 1557,0226 | 236,99485 | 8,26512E-42 | 0,05 | 1 | 105057,8526 | 124852,3723 |
| Control, 28 days, ABAP Control, 7 days, DCFDA | 121864,0175 | 1557,0226 | 266,33811 | 5,18588E-43 | 0,05 | 1 | 111966,7576 | 131761,2773 |
| Control, 28 days, ABAP Control, 14 days, DCFDA | 119253,0188 | 1557,0226 | 255,04752 | 1,44952E-42 | 0,05 | 1 | 109355,759 | 129150,2787 |
| Control, 28 days, ABAP Control, 28 days, DCFDA | 76904,425 | 1557,0226 | 106,06815 | 1,37517E-33 | 0,05 | 1 | 67007,16513 | 86801,68487 |
| Control, 28 days, ABAP Control, 42 days, DCFDA | 106345,2705 | 1557,0226 | 202,8237 | 3,29904E-40 | 0,05 | 1 | 96448,01068 | 116242,5304 |
| Control, 28 days, ABAP Control, 56 days, DCFDA | 125009,9048 | 1557,0226 | 280,26649 | 1,54608E-43 | 0,05 | 1 | 115112,6449 | 134907,1647 |
| Control, 28 days, ABAP DHQ, 1 day, DCFDA | 124815,1077 | 1557,0226 | 279,39372 | 1,66495E-43 | 0,05 | 1 | 114917,8478 | 134712,3676 |
| Control, 28 days, ABAP DHQ, 7 days, DCFDA | 115294,9446 | 1557,0226 | 238,39814 | 7,1858E-42 | 0,05 | 1 | 105397,6847 | 125192,2045 |
| Control, 28 days, ABAP DHQ, 14 days, DCFDA | 122798,3223 | 1557,0226 | 270,43768 | 3,60872E-43 | 0,05 | 1 | 112901,0624 | 132695,5822 |
| Control, 28 days, ABAP DHQ, 28 days, DCFDA | 107348,8831 | 1557,0226 | 206,66998 | 2,11536E-40 | 0,05 | 1 | 97451,62325 | 117246,143 |
| Control, 28 days, ABAP DHQ, 42 days, DCFDA | 108076,0833 | 1557,0226 | 209,4795 | 1,53684E-40 | 0,05 | 1 | 98178,82346 | 117973,3432 |
| Control, 28 days, ABAP DHQ, 56 days, DCFDA | 123030,3073 | 1557,0226 | 271,46044 | 3,29941E-43 | 0,05 | 1 | 113133,0474 | 132927,5672 |
| Control, 28 days, ABAP Control, 1 day, ABAP | 85483,68125 | 1557,0226 | 131,05354 | 9,73106E-36 | 0,05 | 1 | 75586,42138 | 95380,94112 |
| Control, 28 days, ABAP Control, 7 days, ABAP | 89464,7275 | 1557,0226 | 143,54432 | 1,14673E-36 | 0,05 | 1 | 79567,46763 | 99361,98737 |
| Control, 28 days, ABAP Control, 14 days, ABAP | 97465,79222 | 1557,0226 | 170,36751 | 2,02894E-38 | 0,05 | 1 | 87568,53235 | 107363,0521 |
| Control, 42 days, ABAP Control, 1 day, DCFDA | 74898,70528 | 1557,0226 | 100,60764 | 4,7181E-33 | 0,05 | 1 | 65001,4454 | 84795,96515 |
| Control, 42 days, ABAP Control, 7 days, DCFDA | 81807,61028 | 1557,0226 | 120,02446 | 7,64116E-35 | 0,05 | 1 | 71910,3504 | 91704,87015 |
| Control, 42 days, ABAP Control, 14 days, DCFDA | 79196,61167 | 1557,0226 | 112,48524 | 3,48659E-34 | 0,05 | 1 | 69299,35179 | 89093,87154 |
| Control, 42 days, ABAP Control, 28 days, DCFDA | 36848,01778 | 1557,0226 | 24,35065 | 3,53409E-19 | 0,05 | 1 | 26950,7579 | 46745,27765 |
| Control, 42 days, ABAP Control, 42 days, DCFDA | 66288,86333 | 1557,0226 | 78,80676 | 1,36913E-30 | 0,05 | 1 | 56391,60346 | 76186,12321 |
| Control, 42 days, ABAP Control, 56 days, DCFDA | 84953,49763 | 1557,0226 | 129,43295 | 1,30302E-35 | 0,05 | 1 | 75056,23775 | 94850,7575 |
| Control, 42 days, ABAP DHQ, 1 day, DCFDA | 84758,7005 | 1557,0226 | 128,84006 | 1,45119E-35 | 0,05 | 1 | 74861,44062 | 94655,96037 |
| Control, 42 days, ABAP DHQ, 7 days, DCFDA | 75238,53744 | 1557,0226 | 101,52267 | 3,82039E-33 | 0,05 | 1 | 65341,27757 | 85135,79732 |
| Control, 42 days, ABAP DHQ, 14 days, DCFDA | 82741,91512 | 1557,0226 | 122,78165 | 4,48859E-35 | 0,05 | 1 | 72844,65525 | 92639,17499 |
| Control, 42 days, ABAP DHQ, 28 days, DCFDA | 67292,4759 | 1557,0226 | 81,21109 | 6,8313E-31 | 0,05 | 1 | 57395,21603 | 77189,73578 |
| Control, 42 days, ABAP DHQ, 42 days, DCFDA | 68019,67611 | 1557,0226 | 82,9758 | 4,15262E-31 | 0,05 | 1 | 58122,41624 | 77916,93598 |
| Control, 42 days, ABAP DHQ, 56 days, DCFDA | 82973,90013 | 1557,0226 | 123,47111 | 3,93664E-35 | 0,05 | 1 | 73076,64026 | 92871,16 |
| Control, 42 days, ABAP Control, 1 day, ABAP | 45427,27403 | 1557,0226 | 37,00971 | 3,88694E-23 | 0,05 | 1 | 35530,01415 | 55324,5339 |
| Control, 42 days, ABAP Control, 7 days, ABAP | 49408,32028 | 1557,0226 | 43,78068 | 9,14101E-25 | 0,05 | 1 | 39511,0604 | 59305,58015 |
| Control, 42 days, ABAP Control, 14 days, ABAP | 57409,385 | 1557,0226 | 59,10825 | 1,01805E-27 | 0,05 | 1 | 47512,12513 | 67306,64487 |
| Control, 42 days, ABAP Control, 28 days, ABAP | -40056,40722 | 1557,0226 | 28,77573 | 9,73712E-21 | 0,05 | 1 | -49953,6671 | -30159,14735 |
| Control, 56 days, ABAP Control, 1 day, DCFDA | 11937,06689 | 1557,0226 | 2,55651 | 0,00306 | 0,05 | 1 | 2039,80702 | 21834,32677 |
| Control, 56 days, ABAP Control, 7 days, DCFDA | 18845,97189 | 1557,0226 | 6,36971 | 3,61877E-8 | 0,05 | 1 | 8948,71202 | 28743,23177 |
| Control, 56 days, ABAP Control, 14 days, DCFDA | 16234,97328 | 1557,0226 | 4,727 | 2,87068E-6 | 0,05 | 1 | 6337,71341 | 26132,23316 |
| Control, 56 days, ABAP Control, 28 days, DCFDA | -26113,62061 | 1557,0226 | 12,22972 | 4,11174E-13 | 0,05 | 1 | -36010,88048 | -16216,36073 |
| Control, 56 days, ABAP Control, 42 days, DCFDA | 3327,22495 | 1557,0226 | 0,19854 | 0,99995 | 0,05 | 0 | -6570,03492 | 13224,48482 |
| Control, 56 days, ABAP Control, 56 days, DCFDA | 21991,85924 | 1557,0226 | 8,67374 | 2,1827E-10 | 0,05 | 1 | 12094,59937 | 31889,11912 |
| Control, 56 days, ABAP DHQ, 1 day, DCFDA | 21797,06211 | 1557,0226 | 8,52076 | 2,97328E-10 | 0,05 | 1 | 11899,80224 | 31694,32198 |
| Control, 56 days, ABAP DHQ, 7 days, DCFDA | 12276,89906 | 1557,0226 | 2,70309 | 0,00183 | 0,05 | 1 | 2379,63919 | 22174,15893 |
| Control, 56 days, ABAP DHQ, 14 days, DCFDA | 19780,27674 | 1557,0226 | 7,01693 | 7,74129E-9 | 0,05 | 1 | 9883,01686 | 29677,53661 |
| Control, 56 days, ABAP DHQ, 28 days, DCFDA | 4330,83752 | 1557,0226 | 0,33638 | 0,99696 | 0,05 | 0 | -5566,42235 | 14228,09739 |
| Control, 56 days, ABAP DHQ, 42 days, DCFDA | 5058,03773 | 1557,0226 | 0,45882 | 0,97728 | 0,05 | 0 | -4839,22215 | 14955,2976 |
| Control, 56 days, ABAP DHQ, 56 days, DCFDA | 20012,26175 | 1557,0226 | 7,18248 | 5,29369E-9 | 0,05 | 1 | 10115,00187 | 29909,52162 |
| Control, 56 days, ABAP Control, 1 day, ABAP | -17534,36436 | 1557,0226 | 5,51394 | 3,23256E-7 | 0,05 | 1 | -27431,62423 | -7637,10448 |
| Control, 56 days, ABAP Control, 7 days, ABAP | -13553,31811 | 1557,0226 | 3,29438 | 2,45199E-4 | 0,05 | 1 | -23450,57798 | -3656,05823 |
| Control, 56 days, ABAP Control, 14 days, ABAP | -5552,25338 | 1557,0226 | 0,55287 | 0,93748 | 0,05 | 0 | -15449,51326 | 4345,00649 |
| Control, 56 days, ABAP Control, 28 days, ABAP | -103018,0456 | 1557,0226 | 190,33074 | 1,48297E-39 | 0,05 | 1 | -112915,3054 | -93120,78573 |
| Control, 56 days, ABAP Control, 42 days, ABAP | -62961,63838 | 1557,0226 | 71,09423 | 1,47433E-29 | 0,05 | 1 | -72858,89826 | -53064,37851 |
| DHQ, 1 day, ABAP Control, 1 day, DCFDA | -5260,11449 | 1557,0226 | 0,49622 | 0,96456 | 0,05 | 0 | -15157,37437 | 4637,14538 |
| DHQ, 1 day, ABAP Control, 7 days, DCFDA | 1648,79051 | 1557,0226 | 0,04875 | 1 | 0,05 | 0 | -8248,46937 | 11546,05038 |
| DHQ, 1 day, ABAP Control, 14 days, DCFDA | -962,20811 | 1557,0226 | 0,0166 | 1 | 0,05 | 0 | -10859,46798 | 8935,05177 |
| DHQ, 1 day, ABAP Control, 28 days, DCFDA | -43310,80199 | 1557,0226 | 33,64146 | 3,20166E-22 | 0,05 | 1 | -53208,06187 | -33413,54212 |
| DHQ, 1 day, ABAP Control, 42 days, DCFDA | -13869,95644 | 1557,0226 | 3,45011 | 1,46677E-4 | 0,05 | 1 | -23767,21631 | -3972,69656 |
| DHQ, 1 day, ABAP Control, 56 days, DCFDA | 4794,67786 | 1557,0226 | 0,41229 | 0,98813 | 0,05 | 0 | -5102,58202 | 14691,93773 |
| DHQ, 1 day, ABAP DHQ, 1 day, DCFDA | 4599,88072 | 1557,0226 | 0,37947 | 0,99306 | 0,05 | 0 | -5297,37915 | 14497,1406 |
| DHQ, 1 day, ABAP DHQ, 7 days, DCFDA | -4920,28233 | 1557,0226 | 0,43417 | 0,98365 | 0,05 | 0 | -14817,5422 | 4976,97755 |
| DHQ, 1 day, ABAP DHQ, 14 days, DCFDA | 2583,09535 | 1557,0226 | 0,11966 | 1 | 0,05 | 0 | -7314,16453 | 12480,35522 |
| DHQ, 1 day, ABAP DHQ, 28 days, DCFDA | -12866,34387 | 1557,0226 | 2,96888 | 7,33851E-4 | 0,05 | 1 | -22763,60374 | -2969,08399 |
| DHQ, 1 day, ABAP DHQ, 42 days, DCFDA | -12139,14366 | 1557,0226 | 2,64276 | 0,00226 | 0,05 | 1 | -22036,40353 | -2241,88379 |
| DHQ, 1 day, ABAP DHQ, 56 days, DCFDA | 2815,08036 | 1557,0226 | 0,14212 | 1 | 0,05 | 0 | -7082,17951 | 12712,34023 |
| DHQ, 1 day, ABAP Control, 1 day, ABAP | -34731,54574 | 1557,0226 | 21,63369 | 4,32124E-18 | 0,05 | 1 | -44628,80562 | -24834,28587 |
| DHQ, 1 day, ABAP Control, 7 days, ABAP | -30750,49949 | 1557,0226 | 16,95847 | 6,59783E-16 | 0,05 | 1 | -40647,75937 | -20853,23962 |
| DHQ, 1 day, ABAP Control, 14 days, ABAP | -22749,43477 | 1557,0226 | 9,28162 | 6,62857E-11 | 0,05 | 1 | -32646,69464 | -12852,17489 |
| DHQ, 1 day, ABAP Control, 28 days, ABAP | -120215,2269 | 1557,0226 | 259,17989 | 9,89951E-43 | 0,05 | 1 | -130112,4868 | -110317,9671 |
| DHQ, 1 day, ABAP Control, 42 days, ABAP | -80158,81977 | 1557,0226 | 115,23515 | 1,98193E-34 | 0,05 | 1 | -90056,07964 | -70261,5599 |
| DHQ, 1 day, ABAP Control, 56 days, ABAP | -17197,18139 | 1557,0226 | 5,30392 | 5,69355E-7 | 0,05 | 1 | -27094,44126 | -7299,92151 |
| DHQ, 7 days, ABAP Control, 1 day, DCFDA | 12861,01517 | 1557,0226 | 2,96642 | 7,40033E-4 | 0,05 | 1 | 2963,75529 | 22758,27504 |
| DHQ, 7 days, ABAP Control, 7 days, DCFDA | 19769,92017 | 1557,0226 | 7,00958 | 7,87398E-9 | 0,05 | 1 | 9872,66029 | 29667,18004 |
| DHQ, 7 days, ABAP Control, 14 days, DCFDA | 17158,92156 | 1557,0226 | 5,28035 | 6,07154E-7 | 0,05 | 1 | 7261,66168 | 27056,18143 |
| DHQ, 7 days, ABAP Control, 28 days, DCFDA | -25189,67233 | 1557,0226 | 11,37961 | 1,60377E-12 | 0,05 | 1 | -35086,93221 | -15292,41246 |
| DHQ, 7 days, ABAP Control, 42 days, DCFDA | 4251,17322 | 1557,0226 | 0,32412 | 0,99767 | 0,05 | 0 | -5646,08665 | 14148,4331 |
| DHQ, 7 days, ABAP Control, 56 days, DCFDA | 22915,80752 | 1557,0226 | 9,41787 | 5,11365E-11 | 0,05 | 1 | 13018,54764 | 32813,06739 |
| DHQ, 7 days, ABAP DHQ, 1 day, DCFDA | 22721,01038 | 1557,0226 | 9,25844 | 6,9296E-11 | 0,05 | 1 | 12823,75051 | 32618,27026 |
| DHQ, 7 days, ABAP DHQ, 7 days, DCFDA | 13200,84733 | 1557,0226 | 3,12526 | 4,3183E-4 | 0,05 | 1 | 3303,58746 | 23098,10721 |
| DHQ, 7 days, ABAP DHQ, 14 days, DCFDA | 20704,22501 | 1557,0226 | 7,68777 | 1,71677E-9 | 0,05 | 1 | 10806,96513 | 30601,48488 |
| DHQ, 7 days, ABAP DHQ, 28 days, DCFDA | 5254,78579 | 1557,0226 | 0,49521 | 0,96495 | 0,05 | 0 | -4642,47408 | 15152,04567 |
| DHQ, 7 days, ABAP DHQ, 42 days, DCFDA | 5981,986 | 1557,0226 | 0,64176 | 0,87498 | 0,05 | 0 | -3915,27387 | 15879,24587 |
| DHQ, 7 days, ABAP DHQ, 56 days, DCFDA | 20936,21002 | 1557,0226 | 7,86101 | 1,18009E-9 | 0,05 | 1 | 11038,95015 | 30833,46989 |
| DHQ, 7 days, ABAP Control, 1 day, ABAP | -16610,41608 | 1557,0226 | 4,94816 | 1,52684E-6 | 0,05 | 1 | -26507,67596 | -6713,15621 |
| DHQ, 7 days, ABAP Control, 7 days, ABAP | -12629,36983 | 1557,0226 | 2,86052 | 0,00106 | 0,05 | 1 | -22526,62971 | -2732,10996 |
| DHQ, 7 days, ABAP Control, 14 days, ABAP | -4628,30511 | 1557,0226 | 0,38417 | 0,99247 | 0,05 | 0 | -14525,56498 | 5268,95476 |
| DHQ, 7 days, ABAP Control, 28 days, ABAP | -102094,0973 | 1557,0226 | 186,93198 | 2,26998E-39 | 0,05 | 1 | -111991,3572 | -92196,83746 |
| DHQ, 7 days, ABAP Control, 42 days, ABAP | -62037,69011 | 1557,0226 | 69,02296 | 2,91153E-29 | 0,05 | 1 | -71934,94998 | -52140,43024 |
| DHQ, 7 days, ABAP Control, 56 days, ABAP | 923,94827 | 1557,0226 | 0,01531 | 1 | 0,05 | 0 | -8973,3116 | 10821,20815 |
| DHQ, 7 days, ABAP DHQ, 1 day, ABAP | 18121,12966 | 1557,0226 | 5,88915 | 1,21022E-7 | 0,05 | 1 | 8223,86979 | 28018,38953 |
| DHQ, 14 days, ABAP Control, 1 day, DCFDA | -3732,21206 | 1557,0226 | 0,24981 | 0,99968 | 0,05 | 0 | -13629,47194 | 6165,04781 |
| DHQ, 14 days, ABAP Control, 7 days, DCFDA | 3176,69294 | 1557,0226 | 0,18098 | 0,99998 | 0,05 | 0 | -6720,56694 | 13073,95281 |
| DHQ, 14 days, ABAP Control, 14 days, DCFDA | 565,69433 | 1557,0226 | 0,00574 | 1 | 0,05 | 0 | -9331,56555 | 10462,9542 |
| DHQ, 14 days, ABAP Control, 28 days, DCFDA | -41782,89956 | 1557,0226 | 31,30974 | 1,54841E-21 | 0,05 | 1 | -51680,15944 | -31885,63969 |
| DHQ, 14 days, ABAP Control, 42 days, DCFDA | -12342,05401 | 1557,0226 | 2,73185 | 0,00166 | 0,05 | 1 | -22239,31388 | -2444,79413 |
| DHQ, 14 days, ABAP Control, 56 days, DCFDA | 6322,58028 | 1557,0226 | 0,71692 | 0,80495 | 0,05 | 0 | -3574,67959 | 16219,84016 |
| DHQ, 14 days, ABAP DHQ, 1 day, DCFDA | 6127,78315 | 1557,0226 | 0,67342 | 0,84718 | 0,05 | 0 | -3769,47672 | 16025,04303 |
| DHQ, 14 days, ABAP DHQ, 7 days, DCFDA | -3392,3799 | 1557,0226 | 0,20639 | 0,99994 | 0,05 | 0 | -13289,63977 | 6504,87998 |
| DHQ, 14 days, ABAP DHQ, 14 days, DCFDA | 4110,99778 | 1557,0226 | 0,30309 | 0,99858 | 0,05 | 0 | -5786,2621 | 14008,25765 |
| DHQ, 14 days, ABAP DHQ, 28 days, DCFDA | -11338,44144 | 1557,0226 | 2,30563 | 0,00737 | 0,05 | 1 | -21235,70131 | -1441,18156 |
| DHQ, 14 days, ABAP DHQ, 42 days, DCFDA | -10611,24123 | 1557,0226 | 2,01936 | 0,02014 | 0,05 | 1 | -20508,5011 | -713,98136 |
| DHQ, 14 days, ABAP DHQ, 56 days, DCFDA | 4342,98279 | 1557,0226 | 0,33827 | 0,99684 | 0,05 | 0 | -5554,27708 | 14240,24266 |
| DHQ, 14 days, ABAP Control, 1 day, ABAP | -33203,64331 | 1557,0226 | 19,77215 | 2,82853E-17 | 0,05 | 1 | -43100,90319 | -23306,38344 |
| DHQ, 14 days, ABAP Control, 7 days, ABAP | -29222,59706 | 1557,0226 | 15,3515 | 5,11877E-15 | 0,05 | 1 | -39119,85694 | -19325,33719 |
| DHQ, 14 days, ABAP Control, 14 days, ABAP | -21221,53234 | 1557,0226 | 8,07673 | 7,45635E-10 | 0,05 | 1 | -31118,79221 | -11324,27247 |
| DHQ, 14 days, ABAP Control, 28 days, ABAP | -118687,3245 | 1557,0226 | 252,63354 | 1,81634E-42 | 0,05 | 1 | -128584,5844 | -108790,0646 |
| DHQ, 14 days, ABAP Control, 42 days, ABAP | -78630,91734 | 1557,0226 | 110,88403 | 4,87468E-34 | 0,05 | 1 | -88528,17721 | -68733,65747 |
| DHQ, 14 days, ABAP Control, 56 days, ABAP | -15669,27896 | 1557,0226 | 4,40332 | 7,42269E-6 | 0,05 | 1 | -25566,53883 | -5772,01908 |
| DHQ, 14 days, ABAP DHQ, 1 day, ABAP | 1527,90243 | 1557,0226 | 0,04187 | 1 | 0,05 | 0 | -8369,35744 | 11425,1623 |
| DHQ, 14 days, ABAP DHQ, 7 days, ABAP | -16593,22723 | 1557,0226 | 4,93792 | 1,57163E-6 | 0,05 | 1 | -26490,4871 | -6695,96736 |
| DHQ, 28 days, ABAP Control, 1 day, DCFDA | 11859,33313 | 1557,0226 | 2,52234 | 0,00344 | 0,05 | 1 | 1962,07325 | 21756,593 |
| DHQ, 28 days, ABAP Control, 7 days, DCFDA | 18768,23813 | 1557,0226 | 6,31727 | 4,11729E-8 | 0,05 | 1 | 8870,97825 | 28665,498 |
| DHQ, 28 days, ABAP Control, 14 days, DCFDA | 16157,23951 | 1557,0226 | 4,68184 | 3,2714E-6 | 0,05 | 1 | 6259,97964 | 26054,49939 |
| DHQ, 28 days, ABAP Control, 28 days, DCFDA | -26191,35437 | 1557,0226 | 12,30264 | 3,6713E-13 | 0,05 | 1 | -36088,61425 | -16294,0945 |
| DHQ, 28 days, ABAP Control, 42 days, DCFDA | 3249,49118 | 1557,0226 | 0,18937 | 0,99997 | 0,05 | 0 | -6647,76869 | 13146,75105 |
| DHQ, 28 days, ABAP Control, 56 days, DCFDA | 21914,12547 | 1557,0226 | 8,61253 | 2,46893E-10 | 0,05 | 1 | 12016,8656 | 31811,38535 |
| DHQ, 28 days, ABAP DHQ, 1 day, DCFDA | 21719,32834 | 1557,0226 | 8,46009 | 3,36461E-10 | 0,05 | 1 | 11822,06847 | 31616,58822 |
| DHQ, 28 days, ABAP DHQ, 7 days, DCFDA | 12199,16529 | 1557,0226 | 2,66896 | 0,00206 | 0,05 | 1 | 2301,90542 | 22096,42517 |
| DHQ, 28 days, ABAP DHQ, 14 days, DCFDA | 19702,54297 | 1557,0226 | 6,96189 | 8,79502E-9 | 0,05 | 1 | 9805,28309 | 29599,80284 |
| DHQ, 28 days, ABAP DHQ, 28 days, DCFDA | 4253,10375 | 1557,0226 | 0,32441 | 0,99766 | 0,05 | 0 | -5644,15612 | 14150,36362 |
| DHQ, 28 days, ABAP DHQ, 42 days, DCFDA | 4980,30396 | 1557,0226 | 0,44483 | 0,98108 | 0,05 | 0 | -4916,95592 | 14877,56383 |
| DHQ, 28 days, ABAP DHQ, 56 days, DCFDA | 19934,52798 | 1557,0226 | 7,12679 | 6,01183E-9 | 0,05 | 1 | 10037,2681 | 29831,78785 |
| DHQ, 28 days, ABAP Control, 1 day, ABAP | -17612,09812 | 1557,0226 | 5,56294 | 2,83746E-7 | 0,05 | 1 | -27509,358 | -7714,83825 |
| DHQ, 28 days, ABAP Control, 7 days, ABAP | -13631,05187 | 1557,0226 | 3,33228 | 2,16235E-4 | 0,05 | 1 | -23528,31175 | -3733,792 |
| DHQ, 28 days, ABAP Control, 14 days, ABAP | -5629,98715 | 1557,0226 | 0,56846 | 0,92828 | 0,05 | 0 | -15527,24703 | 4267,27272 |
| DHQ, 28 days, ABAP Control, 28 days, ABAP | -103095,7793 | 1557,0226 | 190,61809 | 1,43103E-39 | 0,05 | 1 | -112993,0392 | -93198,5195 |
| DHQ, 28 days, ABAP Control, 42 days, ABAP | -63039,37215 | 1557,0226 | 71,26989 | 1,39288E-29 | 0,05 | 1 | -72936,63203 | -53142,11228 |
| DHQ, 28 days, ABAP Control, 56 days, ABAP | -77,73377 | 1557,0226 | 1,08368E- | 1 | 0,05 | 0 | -9974,99364 | 9819,5261 |
| DHQ, 28 days, ABAP DHQ, 1 day, ABAP | 17119,44762 | 1557,0226 | 5,25608 | 6,48791E-7 | 0,05 | 1 | 7222,18774 | 27016,74749 |
| DHQ, 28 days, ABAP DHQ, 7 days, ABAP | -1001,68204 | 1557,0226 | 0,01799 | 1 | 0,05 | 0 | -10898,94192 | 8895,57783 |
| DHQ, 28 days, ABAP DHQ, 14 days, ABAP | 15591,54519 | 1557,0226 | 4,35974 | 8,45582E-6 | 0,05 | 1 | 5694,28531 | 25488,80506 |
| DHQ, 42 days, ABAP Control, 1 day, DCFDA | 6569,9125 | 1557,0226 | 0,77411 | 0,74379 | 0,05 | 0 | -3327,34737 | 16467,17237 |
| DHQ, 42 days, ABAP Control, 7 days, DCFDA | 13478,8175 | 1557,0226 | 3,25826 | 2,76513E-4 | 0,05 | 1 | 3581,55763 | 23376,07737 |
| DHQ, 42 days, ABAP Control, 14 days, DCFDA | 10867,81889 | 1557,0226 | 2,1182 | 0,01424 | 0,05 | 1 | 970,55902 | 20765,07876 |
| DHQ, 42 days, ABAP Control, 28 days, DCFDA | -31480,775 | 1557,0226 | 17,77351 | 2,53826E-16 | 0,05 | 1 | -41378,03487 | -21583,51513 |
| DHQ, 42 days, ABAP Control, 42 days, DCFDA | -2039,92944 | 1557,0226 | 0,07463 | 1 | 0,05 | 0 | -11937,18932 | 7857,33043 |
| DHQ, 42 days, ABAP Control, 56 days, DCFDA | 16624,70485 | 1557,0226 | 4,95667 | 1,49059E-6 | 0,05 | 1 | 6727,44497 | 26521,96472 |
| DHQ, 42 days, ABAP DHQ, 1 day, DCFDA | 16429,90772 | 1557,0226 | 4,8412 | 2,06841E-6 | 0,05 | 1 | 6532,64784 | 26327,16759 |
| DHQ, 42 days, ABAP DHQ, 7 days, DCFDA | 6909,74467 | 1557,0226 | 0,85626 | 0,64919 | 0,05 | 0 | -2987,51521 | 16807,00454 |
| DHQ, 42 days, ABAP DHQ, 14 days, DCFDA | 14413,12234 | 1557,0226 | 3,72562 | 6,01624E-5 | 0,05 | 1 | 4515,86247 | 24310,38221 |
| DHQ, 42 days, ABAP DHQ, 28 days, DCFDA | -1036,31688 | 1557,0226 | 0,01926 | 1 | 0,05 | 0 | -10933,57675 | 8860,943 |
| DHQ, 42 days, ABAP DHQ, 42 days, DCFDA | -309,11667 | 1557,0226 | 0,00171 | 1 | 0,05 | 0 | -10206,37654 | 9588,14321 |
| DHQ, 42 days, ABAP DHQ, 56 days, DCFDA | 14645,10735 | 1557,0226 | 3,84652 | 4,09912E-5 | 0,05 | 1 | 4747,84748 | 24542,36723 |
| DHQ, 42 days, ABAP Control, 1 day, ABAP | -22901,51875 | 1557,0226 | 9,40613 | 5,22872E-11 | 0,05 | 1 | -32798,77862 | -13004,25888 |
| DHQ, 42 days, ABAP Control, 7 days, ABAP | -18920,4725 | 1557,0226 | 6,42017 | 3,19805E-8 | 0,05 | 1 | -28817,73237 | -9023,21263 |
| DHQ, 42 days, ABAP Control, 14 days, ABAP | -10919,40778 | 1557,0226 | 2,13836 | 0,01327 | 0,05 | 1 | -20816,66765 | -1022,1479 |
| DHQ, 42 days, ABAP Control, 28 days, ABAP | -108385,2 | 1557,0226 | 210,67951 | 1,34252E-40 | 0,05 | 1 | -118282,4598 | -98487,94013 |
| DHQ, 42 days, ABAP Control, 42 days, ABAP | -68328,79278 | 1557,0226 | 83,73168 | 3,36572E-31 | 0,05 | 1 | -78226,05265 | -58431,5329 |
| DHQ, 42 days, ABAP Control, 56 days, ABAP | -5367,15439 | 1557,0226 | 0,51662 | 0,95593 | 0,05 | 0 | -15264,41427 | 4530,10548 |
| DHQ, 42 days, ABAP DHQ, 1 day, ABAP | 11830,02699 | 1557,0226 | 2,50988 | 0,00359 | 0,05 | 1 | 1932,76712 | 21727,28687 |
| DHQ, 42 days, ABAP DHQ, 7 days, ABAP | -6291,10267 | 1557,0226 | 0,7098 | 0,81215 | 0,05 | 0 | -16188,36254 | 3606,15721 |
| DHQ, 42 days, ABAP DHQ, 14 days, ABAP | 10302,12456 | 1557,0226 | 1,90343 | 0,03018 | 0,05 | 1 | 404,86469 | 20199,38444 |
| DHQ, 42 days, ABAP DHQ, 28 days, ABAP | -5289,42063 | 1557,0226 | 0,50176 | 0,96234 | 0,05 | 0 | -15186,6805 | 4607,83925 |
| DHQ, 56 days, ABAP Control, 1 day, DCFDA | -2934,4674 | 1557,0226 | 0,15443 | 1 | 0,05 | 0 | -12831,72728 | 6962,79247 |
| DHQ, 56 days, ABAP Control, 7 days, DCFDA | 3974,4376 | 1557,0226 | 0,28329 | 0,99915 | 0,05 | 0 | -5922,82228 | 13871,69747 |
| DHQ, 56 days, ABAP Control, 14 days, DCFDA | 1363,43899 | 1557,0226 | 0,03334 | 1 | 0,05 | 0 | -8533,82089 | 11260,69886 |
| DHQ, 56 days, ABAP Control, 28 days, DCFDA | -40985,1549 | 1557,0226 | 30,12559 | 3,59312E-21 | 0,05 | 1 | -50882,41478 | -31087,89503 |
| DHQ, 56 days, ABAP Control, 42 days, DCFDA | -11544,30935 | 1557,0226 | 2,39011 | 0,00547 | 0,05 | 1 | -21441,56922 | -1647,04947 |
| DHQ, 56 days, ABAP Control, 56 days, DCFDA | 7120,32495 | 1557,0226 | 0,90925 | 0,58688 | 0,05 | 0 | -2776,93493 | 17017,58482 |
| DHQ, 56 days, ABAP DHQ, 1 day, DCFDA | 6925,52782 | 1557,0226 | 0,86018 | 0,64459 | 0,05 | 0 | -2971,73206 | 16822,78769 |
| DHQ, 56 days, ABAP DHQ, 7 days, DCFDA | -2594,63524 | 1557,0226 | 0,12074 | 1 | 0,05 | 0 | -12491,89511 | 7302,62464 |
| DHQ, 56 days, ABAP DHQ, 14 days, DCFDA | 4908,74244 | 1557,0226 | 0,43214 | 0,98411 | 0,05 | 0 | -4988,51743 | 14806,00231 |
| DHQ, 56 days, ABAP DHQ, 28 days, DCFDA | -10540,69678 | 1557,0226 | 1,9926 | 0,02212 | 0,05 | 1 | -20437,95665 | -643,4369 |
| DHQ, 56 days, ABAP DHQ, 42 days, DCFDA | -9813,49657 | 1557,0226 | 1,72715 | 0,0553 | 0,05 | 0 | -19710,75644 | 83,76331 |
| DHQ, 56 days, ABAP DHQ, 56 days, DCFDA | 5140,72745 | 1557,0226 | 0,47395 | 0,9726 | 0,05 | 0 | -4756,53242 | 15037,98732 |
| DHQ, 56 days, ABAP Control, 1 day, ABAP | -32405,89865 | 1557,0226 | 18,83348 | 7,73367E-17 | 0,05 | 1 | -42303,15853 | -22508,63878 |
| DHQ, 56 days, ABAP Control, 7 days, ABAP | -28424,8524 | 1557,0226 | 14,49035 | 1,53358E-14 | 0,05 | 1 | -38322,11228 | -18527,59253 |
| DHQ, 56 days, ABAP Control, 14 days, ABAP | -20423,78768 | 1557,0226 | 7,48092 | 2,70584E-9 | 0,05 | 1 | -30321,04755 | -10526,52781 |
| DHQ, 56 days, ABAP Control, 28 days, ABAP | -117889,5799 | 1557,0226 | 249,2886 | 2,50115E-42 | 0,05 | 1 | -127786,8397 | -107992,32 |
| DHQ, 56 days, ABAP Control, 42 days, ABAP | -77833,17268 | 1557,0226 | 108,64551 | 7,85034E-34 | 0,05 | 1 | -87730,43255 | -67935,91281 |
| DHQ, 56 days, ABAP Control, 56 days, ABAP | -14871,5343 | 1557,0226 | 3,96638 | 2,81464E-5 | 0,05 | 1 | -24768,79417 | -4974,27442 |
| DHQ, 56 days, ABAP DHQ, 1 day, ABAP | 2325,64709 | 1557,0226 | 0,097 | 1 | 0,05 | 0 | -7571,61278 | 12222,90696 |
| DHQ, 56 days, ABAP DHQ, 7 days, ABAP | -15795,48257 | 1557,0226 | 4,47454 | 6,00643E-6 | 0,05 | 1 | -25692,74244 | -5898,22269 |
| DHQ, 56 days, ABAP DHQ, 14 days, ABAP | 797,74466 | 1557,0226 | 0,01141 | 1 | 0,05 | 0 | -9099,51521 | 10695,00454 |
| DHQ, 56 days, ABAP DHQ, 28 days, ABAP | -14793,80053 | 1557,0226 | 3,92502 | 3,20286E-5 | 0,05 | 1 | -24691,0604 | -4896,54065 |
| DHQ, 56 days, ABAP DHQ, 42 days, ABAP | -9504,3799 | 1557,0226 | 1,62006 | 0,07926 | 0,05 | 0 | -19401,63978 | 392,87997 |

*Tukey Test*

| | MeanDiff | SEM | q Value | Prob | Alpha | Sig | LCL | UCL |
|---|---|---|---|---|---|---|---|---|
| Control, 7 days, DCFDA Control, 1 day, DCFDA | -6908,905 | 1557,0226 | 6,27523 | 0,00966 | 0,05 | 1 | -12910,36013 | -907,44987 |
| Control, 14 days, DCFDA Control, 1 day, DCFDA | -4297,90639 | 1557,0226 | 3,90371 | 0,48857 | 0,05 | 0 | -10299,36152 | 1703,54874 |
| Control, 14 days, DCFDA Control, 7 days, DCFDA | 2610,99861 | 1557,0226 | 2,37152 | 0,98844 | 0,05 | 0 | -3390,45652 | 8612,45374 |
| Control, 28 days, DCFDA Control, 1 day, DCFDA | 38050,6875 | 1557,0226 | 34,5607 | 0 | 0,05 | 1 | 32049,23237 | 44052,14263 |
| Control, 28 days, DCFDA Control, 7 days, DCFDA | 44959,5925 | 1557,0226 | 40,83593 | 0 | 0,05 | 1 | 38958,13737 | 50961,04763 |
| Control, 28 days, DCFDA Control, 14 days, DCFDA | 42348,59389 | 1557,0226 | 38,46441 | 0 | 0,05 | 1 | 36347,13876 | 48350,04902 |
| Control, 42 days, DCFDA Control, 1 day, DCFDA | 8609,84194 | 1557,0226 | 7,82015 | 2,9343E-4 | 0,05 | 1 | 2608,38681 | 14611,29708 |
| Control, 42 days, DCFDA Control, 7 days, DCFDA | 15518,74694 | 1557,0226 | 14,09538 | 0 | 0,05 | 1 | 9517,29181 | 21520,20208 |
| Control, 42 days, DCFDA Control, 14 days, DCFDA | 12907,74833 | 1557,0226 | 11,72386 | 2,20738E-8 | 0,05 | 1 | 6906,2932 | 18909,20346 |
| Control, 42 days, DCFDA Control, 28 days, DCFDA | -29440,84556 | 1557,0226 | 26,74055 | 1,91406E-7 | 0,05 | 1 | -35442,30069 | -23439,39042 |
| Control, 56 days, DCFDA Control, 1 day, DCFDA | -10054,79235 | 1557,0226 | 9,13257 | 1,22236E-5 | 0,05 | 1 | -16056,24748 | -4053,33722 |
| Control, 56 days, DCFDA Control, 7 days, DCFDA | -3145,88735 | 1557,0226 | 2,85735 | 0,92398 | 0,05 | 0 | -9147,34248 | 2855,56778 |
| Control, 56 days, DCFDA Control, 14 days, DCFDA | -5756,88596 | 1557,0226 | 5,22887 | 0,07478 | 0,05 | 0 | -11758,34109 | 244,56917 |
| Control, 56 days, DCFDA Control, 28 days, DCFDA | -48105,47985 | 1557,0226 | 43,69328 | 0 | 0,05 | 1 | -54106,93498 | -42104,02472 |
| Control, 56 days, DCFDA Control, 42 days, DCFDA | -18664,63429 | 1557,0226 | 16,95273 | 0 | 0,05 | 1 | -24666,08942 | -12663,17916 |
| DHQ, 1 day, DCFDA Control, 1 day, DCFDA | -9859,99522 | 1557,0226 | 8,95564 | 1,88691E-5 | 0,05 | 1 | -15861,45035 | -3858,54009 |
| DHQ, 1 day, DCFDA Control, 7 days, DCFDA | -2951,09022 | 1557,0226 | 2,68042 | 0,9576 | 0,05 | 0 | -8952,54535 | 3050,36491 |
| DHQ, 1 day, DCFDA Control, 14 days, DCFDA | -5562,08883 | 1557,0226 | 5,05194 | 0,10151 | 0,05 | 0 | -11563,54396 | 439,3663 |
| DHQ, 1 day, DCFDA Control, 28 days, DCFDA | -47910,68272 | 1557,0226 | 43,51635 | 0 | 0,05 | 1 | -53912,13785 | -41909,22759 |
| DHQ, 1 day, DCFDA Control, 42 days, DCFDA | -18469,83716 | 1557,0226 | 16,7758 | 0 | 0,05 | 1 | -24471,29229 | -12468,38203 |
| DHQ, 1 day, DCFDA Control, 56 days, DCFDA | 194,79713 | 1557,0226 | 0,17693 | 1 | 0,05 | 0 | -5806,658 | 6196,25226 |
| DHQ, 7 days, DCFDA Control, 1 day, DCFDA | -339,83217 | 1557,0226 | 0,30866 | 1 | 0,05 | 0 | -6341,2873 | 5661,62296 |
| DHQ, 7 days, DCFDA Control, 7 days, DCFDA | 6569,07283 | 1557,0226 | 5,96656 | 0,01833 | 0,05 | 1 | 567,6177 | 12570,52796 |
| DHQ, 7 days, DCFDA Control, 14 days, DCFDA | 3958,07422 | 1557,0226 | 3,59504 | 0,64153 | 0,05 | 0 | -2043,38091 | 9959,52935 |
| DHQ, 7 days, DCFDA Control, 28 days, DCFDA | -38390,51967 | 1557,0226 | 34,86937 | 0 | 0,05 | 1 | -44391,9748 | -32389,06454 |
| DHQ, 7 days, DCFDA Control, 42 days, DCFDA | -8949,67411 | 1557,0226 | 8,12882 | 1,40468E-4 | 0,05 | 1 | -14951,12924 | -2948,21898 |
| DHQ, 7 days, DCFDA Control, 56 days, DCFDA | 9714,96018 | 1557,0226 | 8,82391 | 2,60475E-5 | 0,05 | 1 | 3713,50505 | 15716,41531 |
| DHQ, 7 days, DCFDA DHQ, 1 day, DCFDA | 9520,16305 | 1557,0226 | 8,64698 | 4,01096E-5 | 0,05 | 1 | 3518,70792 | 15521,61818 |
| DHQ, 14 days, DCFDA Control, 1 day, DCFDA | -7843,20984 | 1557,0226 | 7,12384 | 0,00149 | 0,05 | 1 | -13844,66497 | -1841,75471 |
| DHQ, 14 days, DCFDA Control, 7 days, DCFDA | -934,30484 | 1557,0226 | 0,84861 | 1 | 0,05 | 0 | -6935,75997 | 5067,15029 |
| DHQ, 14 days, DCFDA Control, 14 days, DCFDA | -3545,30345 | 1557,0226 | 3,22013 | 0,81052 | 0,05 | 0 | -9546,75858 | 2456,15168 |
| DHQ, 14 days, DCFDA Control, 28 days, DCFDA | -45893,89734 | 1557,0226 | 41,68454 | 0 | 0,05 | 1 | -51895,35247 | -39892,44221 |
| DHQ, 14 days, DCFDA Control, 42 days, DCFDA | -16453,05179 | 1557,0226 | 14,94399 | 0 | 0,05 | 1 | -22454,50692 | -10451,59665 |
| DHQ, 14 days, DCFDA Control, 56 days, DCFDA | 2211,58251 | 1557,0226 | 2,00874 | 0,99861 | 0,05 | 0 | -3789,87262 | 8213,03764 |
| DHQ, 14 days, DCFDA DHQ, 1 day, DCFDA | 2016,78538 | 1557,0226 | 1,83181 | 0,99964 | 0,05 | 0 | -3984,66975 | 8018,24051 |
| DHQ, 14 days, DCFDA DHQ, 7 days, DCFDA | -7503,37767 | 1557,0226 | 6,81517 | 0,00299 | 0,05 | 1 | -13504,83281 | -1501,92254 |
| DHQ, 28 days, DCFDA Control, 1 day, DCFDA | 7606,22938 | 1557,0226 | 6,90859 | 0,00242 | 0,05 | 1 | 1604,77424 | 13607,68451 |
| DHQ, 28 days, DCFDA Control, 7 days, DCFDA | 14515,13438 | 1557,0226 | 13,18382 | 0 | 0,05 | 1 | 8513,67924 | 20516,58951 |
| DHQ, 28 days, DCFDA Control, 14 days, DCFDA | 11904,13576 | 1557,0226 | 10,8123 | 1,01484E-6 | 0,05 | 1 | 5902,68063 | 17905,59089 |
| DHQ, 28 days, DCFDA Control, 28 days, DCFDA | -30444,45812 | 1557,0226 | 27,65211 | 3,54108E-8 | 0,05 | 1 | -36445,91326 | -24443,00299 |
| DHQ, 28 days, DCFDA Control, 42 days, DCFDA | -1003,61257 | 1557,0226 | 0,91156 | 1 | 0,05 | 0 | -7005,0677 | 4997,84256 |
| DHQ, 28 days, DCFDA Control, 56 days, DCFDA | 17661,02172 | 1557,0226 | 16,04117 | 0 | 0,05 | 1 | 11659,56659 | 23662,47685 |
| DHQ, 28 days, DCFDA DHQ, 1 day, DCFDA | 17466,22459 | 1557,0226 | 15,86423 | 0 | 0,05 | 1 | 11464,76946 | 23467,67972 |
| DHQ, 28 days, DCFDA DHQ, 7 days, DCFDA | 7946,06154 | 1557,0226 | 7,21725 | 0,0012 | 0,05 | 1 | 1944,60641 | 13947,51667 |
| DHQ, 28 days, DCFDA DHQ, 14 days, DCFDA | 15449,43922 | 1557,0226 | 14,03243 | 0 | 0,05 | 1 | 9447,98409 | 21450,89435 |
| DHQ, 42 days, DCFDA Control, 1 day, DCFDA | 6879,02917 | 1557,0226 | 6,24809 | 0,01023 | 0,05 | 1 | 877,57404 | 12880,4843 |
| DHQ, 42 days, DCFDA Control, 7 days, DCFDA | 13787,93417 | 1557,0226 | 12,52331 | 1,20222E-1 | 0,05 | 1 | 7786,47904 | 19789,3893 |
| DHQ, 42 days, DCFDA Control, 14 days, DCFDA | 11176,93556 | 1557,0226 | 10,15179 | 1,30756E-6 | 0,05 | 1 | 5175,48042 | 17178,39069 |
| DHQ, 42 days, DCFDA Control, 28 days, DCFDA | -31171,65833 | 1557,0226 | 28,31262 | 3,84206E-8 | 0,05 | 1 | -37173,11346 | -25170,2032 |
| DHQ, 42 days, DCFDA Control, 42 days, DCFDA | -1730,81278 | 1557,0226 | 1,57206 | 0,99997 | 0,05 | 0 | -7732,26791 | 4270,64235 |
| DHQ, 42 days, DCFDA Control, 56 days, DCFDA | 16933,82152 | 1557,0226 | 15,38066 | 0 | 0,05 | 1 | 10932,36638 | 22935,27665 |
| DHQ, 42 days, DCFDA DHQ, 1 day, DCFDA | 16739,02438 | 1557,0226 | 15,20373 | 0 | 0,05 | 1 | 10737,56925 | 22740,47952 |
| DHQ, 42 days, DCFDA DHQ, 7 days, DCFDA | 7218,86133 | 1557,0226 | 6,55675 | 0,00528 | 0,05 | 1 | 1217,4062 | 13220,31646 |
| DHQ, 42 days, DCFDA DHQ, 14 days, DCFDA | 14722,23901 | 1557,0226 | 13,37193 | 0 | 0,05 | 1 | 8720,78388 | 20723,69414 |
| DHQ, 42 days, DCFDA DHQ, 28 days, DCFDA | -727,20021 | 1557,0226 | 0,6605 | 1 | 0,05 | 0 | -6728,65534 | 5274,25492 |
| DHQ, 56 days, DCFDA Control, 1 day, DCFDA | -8075,19485 | 1557,0226 | 7,33454 | 9,16161E-4 | 0,05 | 1 | -14076,64998 | -2073,73972 |
| DHQ, 56 days, DCFDA Control, 7 days, DCFDA | -1166,28985 | 1557,0226 | 1,05932 | 1 | 0,05 | 0 | -7167,74498 | 4835,16528 |
| DHQ, 56 days, DCFDA Control, 14 days, DCFDA | -3777,28846 | 1557,0226 | 3,43084 | 0,72005 | 0,05 | 0 | -9778,7436 | 2224,16667 |
| DHQ, 56 days, DCFDA Control, 28 days, DCFDA | -46125,88235 | 1557,0226 | 41,89525 | 0 | 0,05 | 1 | -52127,33748 | -40124,42722 |
| DHQ, 56 days, DCFDA Control, 42 days, DCFDA | -16685,0368 | 1557,0226 | 15,1547 | 0 | 0,05 | 1 | -22686,49193 | -10683,58167 |
| DHQ, 56 days, DCFDA Control, 56 days, DCFDA | 1979,5975 | 1557,0226 | 1,79803 | 0,99973 | 0,05 | 0 | -4021,85764 | 7981,05263 |
| DHQ, 56 days, DCFDA DHQ, 1 day, DCFDA | 1784,80036 | 1557,0226 | 1,6211 | 0,99995 | 0,05 | 0 | -4216,65477 | 7786,2555 |
| DHQ, 56 days, DCFDA DHQ, 7 days, DCFDA | -7735,36269 | 1557,0226 | 7,02588 | 0,00186 | 0,05 | 1 | -13736,81782 | -1733,90756 |
| DHQ, 56 days, DCFDA DHQ, 14 days, DCFDA | -231,98501 | 1557,0226 | 0,21071 | 1 | 0,05 | 0 | -6233,44014 | 5769,47012 |
| DHQ, 56 days, DCFDA DHQ, 28 days, DCFDA | -15681,42423 | 1557,0226 | 14,24313 | 0 | 0,05 | 1 | -21682,87936 | -9679,9691 |
| DHQ, 56 days, DCFDA DHQ, 42 days, DCFDA | -14954,22402 | 1557,0226 | 13,58263 | 0 | 0,05 | 1 | -20955,67915 | -8952,76889 |
| Control, 1 day, ABAP Control, 1 day, DCFDA | 29471,43125 | 1557,0226 | 26,76833 | 1,92063E-7 | 0,05 | 1 | 23469,97612 | 35472,88638 |
| Control, 1 day, ABAP Control, 7 days, DCFDA | 36380,33625 | 1557,0226 | 33,04356 | 0 | 0,05 | 1 | 30378,88112 | 42381,79138 |
| Control, 1 day, ABAP Control, 14 days, DCFDA | 33769,33764 | 1557,0226 | 30,67204 | 0 | 0,05 | 1 | 27767,88251 | 39770,79277 |
| Control, 1 day, ABAP Control, 28 days, DCFDA | -8579,25625 | 1557,0226 | 7,79237 | 3,13404E-4 | 0,05 | 1 | -14580,71138 | -2577,80112 |
| Control, 1 day, ABAP Control, 42 days, DCFDA | 20861,58931 | 1557,0226 | 18,94818 | 0 | 0,05 | 1 | 14860,13417 | 26863,04444 |
| Control, 1 day, ABAP Control, 56 days, DCFDA | 39526,2236 | 1557,0226 | 35,90091 | 0 | 0,05 | 1 | 33524,76847 | 45527,67873 |
| Control, 1 day, ABAP DHQ, 1 day, DCFDA | 39331,42647 | 1557,0226 | 35,72398 | 0 | 0,05 | 1 | 33329,97134 | 45332,8816 |
| Control, 1 day, ABAP DHQ, 7 days, DCFDA | 29811,26342 | 1557,0226 | 27,07699 | 1,99349E-7 | 0,05 | 1 | 23809,80829 | 35812,71855 |
| Control, 1 day, ABAP DHQ, 14 days, DCFDA | 37314,64109 | 1557,0226 | 33,89217 | 0 | 0,05 | 1 | 31313,18596 | 43316,09622 |
| Control, 1 day, ABAP DHQ, 28 days, DCFDA | 21865,20187 | 1557,0226 | 19,85974 | 0 | 0,05 | 1 | 15863,74674 | 27866,65701 |
| Control, 1 day, ABAP DHQ, 42 days, DCFDA | 22592,40208 | 1557,0226 | 20,52024 | 0 | 0,05 | 1 | 16590,94695 | 28593,85721 |
| Control, 1 day, ABAP DHQ, 56 days, DCFDA | 37546,6261 | 1557,0226 | 34,10288 | 0 | 0,05 | 1 | 31545,17097 | 43548,08123 |
| Control, 7 days, ABAP Control, 1 day, DCFDA | 25490,385 | 1557,0226 | 23,15242 | 0 | 0,05 | 1 | 19488,92987 | 31491,84013 |
| Control, 7 days, ABAP Control, 7 days, DCFDA | 32399,29 | 1557,0226 | 29,42765 | 4,31273E-8 | 0,05 | 1 | 26397,83487 | 38400,74513 |
| Control, 7 days, ABAP Control, 14 days, DCFDA | 29788,29139 | 1557,0226 | 27,05613 | 1,98866E-7 | 0,05 | 1 | 23786,83626 | 35789,74652 |
| Control, 7 days, ABAP Control, 28 days, DCFDA | -12560,3025 | 1557,0226 | 11,40828 | 1,25193E-7 | 0,05 | 1 | -18561,75763 | -6558,84737 |
| Control, 7 days, ABAP Control, 42 days, DCFDA | 16880,54306 | 1557,0226 | 15,33227 | 0 | 0,05 | 1 | 10879,08792 | 22881,99819 |
| Control, 7 days, ABAP Control, 56 days, DCFDA | 35545,17735 | 1557,0226 | 32,285 | 0 | 0,05 | 1 | 29543,72222 | 41546,63248 |
| Control, 7 days, ABAP DHQ, 1 day, DCFDA | 35350,38022 | 1557,0226 | 32,10807 | 0 | 0,05 | 1 | 29348,92509 | 41351,83535 |
| Control, 7 days, ABAP DHQ, 7 days, DCFDA | 25830,21717 | 1557,0226 | 23,46109 | 0 | 0,05 | 1 | 19828,76204 | 31831,6723 |
| Control, 7 days, ABAP DHQ, 14 days, DCFDA | 33333,59484 | 1557,0226 | 30,27626 | 4,64043E-8 | 0,05 | 1 | 27332,13971 | 39335,04997 |
| Control, 7 days, ABAP DHQ, 28 days, DCFDA | 17884,15562 | 1557,0226 | 16,24383 | 0 | 0,05 | 1 | 11882,70049 | 23885,61076 |
| Control, 7 days, ABAP DHQ, 42 days, DCFDA | 18611,35583 | 1557,0226 | 16,90434 | 0 | 0,05 | 1 | 12609,9007 | 24612,81096 |
| Control, 7 days, ABAP DHQ, 56 days, DCFDA | 33565,57985 | 1557,0226 | 30,48697 | 0 | 0,05 | 1 | 27564,12472 | 39567,03498 |
| Control, 7 days, ABAP Control, 1 day, ABAP | -3981,04625 | 1557,0226 | 3,61591 | 0,63126 | 0,05 | 0 | -9982,50138 | 2020,40888 |
| Control, 14 days, ABAP Control, 1 day, DCFDA | 17489,32028 | 1557,0226 | 15,88521 | 0 | 0,05 | 1 | 11487,86515 | 23490,77541 |
| Control, 14 days, ABAP Control, 7 days, DCFDA | 24398,22528 | 1557,0226 | 22,16044 | 0 | 0,05 | 1 | 18396,77015 | 30399,68041 |
| Control, 14 days, ABAP Control, 14 days, DCFDA | 21787,22667 | 1557,0226 | 19,78892 | 0 | 0,05 | 1 | 15785,77154 | 27788,6818 |
| Control, 14 days, ABAP Control, 28 days, DCFDA | -20561,36722 | 1557,0226 | 18,67549 | 0 | 0,05 | 1 | -26562,82235 | -14559,91209 |
| Control, 14 days, ABAP Control, 42 days, DCFDA | 8879,47833 | 1557,0226 | 8,06506 | 1,63667E-4 | 0,05 | 1 | 2878,0232 | 14880,93346 |
| Control, 14 days, ABAP Control, 56 days, DCFDA | 27544,11263 | 1557,0226 | 25,01779 | 0 | 0,05 | 1 | 21542,6575 | 33545,56776 |
| Control, 14 days, ABAP DHQ, 1 day, DCFDA | 27349,3155 | 1557,0226 | 24,84086 | 0 | 0,05 | 1 | 21347,86036 | 33350,77063 |
| Control, 14 days, ABAP DHQ, 7 days, DCFDA | 17829,15244 | 1557,0226 | 16,19387 | 0 | 0,05 | 1 | 11827,69731 | 23830,60758 |
| Control, 14 days, ABAP DHQ, 14 days, DCFDA | 25332,53012 | 1557,0226 | 23,00905 | 0 | 0,05 | 1 | 19331,07499 | 31333,98525 |
| Control, 14 days, ABAP DHQ, 28 days, DCFDA | 9883,0909 | 1557,0226 | 8,97662 | 1,79235E-5 | 0,05 | 1 | 3881,63577 | 15884,54603 |
| Control, 14 days, ABAP DHQ, 42 days, DCFDA | 10610,29111 | 1557,0226 | 9,63712 | 3,57592E-6 | 0,05 | 1 | 4608,83598 | 16611,74624 |
| Control, 14 days, ABAP DHQ, 56 days, DCFDA | 25564,51513 | 1557,0226 | 23,21976 | 0 | 0,05 | 1 | 19563,06 | 31565,97026 |
| Control, 14 days, ABAP Control, 1 day, ABAP | -11982,11097 | 1557,0226 | 10,88312 | 9,968E-7 | 0,05 | 1 | -17983,5661 | -5980,65584 |
| Control, 14 days, ABAP Control, 7 days, ABAP | -8001,06472 | 1557,0226 | 7,26721 | 0,00107 | 0,05 | 1 | -14002,51985 | -1999,60959 |
| Control, 28 days, ABAP Control, 1 day, DCFDA | 114955,1125 | 1557,0226 | 104,4115 | 0 | 0,05 | 1 | 108953,6573 | 120956,5676 |
| Control, 28 days, ABAP Control, 7 days, DCFDA | 121864,0175 | 1557,0226 | 110,6867 | 0 | 0,05 | 1 | 115862,5623 | 127865,4726 |
| Control, 28 days, ABAP Control, 14 days, DCFDA | 119253,0188 | 1557,0226 | 108,3152 | 0 | 0,05 | 1 | 113251,5637 | 125254,474 |
| Control, 28 days, ABAP Control, 28 days, DCFDA | 76904,425 | 1557,0226 | 69,8508 | 0 | 0,05 | 1 | 70902,96987 | 82905,88013 |
| Control, 28 days, ABAP Control, 42 days, DCFDA | 106345,2705 | 1557,0226 | 96,59136 | 0 | 0,05 | 1 | 100343,8154 | 112346,7256 |
| Control, 28 days, ABAP Control, 56 days, DCFDA | 125009,9048 | 1557,0226 | 113,544 | 0 | 0,05 | 1 | 119008,4497 | 131011,3599 |
| Control, 28 days, ABAP DHQ, 1 day, DCFDA | 124815,1077 | 1557,0226 | 113,3671 | 0 | 0,05 | 1 | 118813,6525 | 130816,5628 |
| Control, 28 days, ABAP DHQ, 7 days, DCFDA | 115294,9446 | 1557,0226 | 104,7201 | 0 | 0,05 | 1 | 109293,4895 | 121296,3998 |
| Control, 28 days, ABAP DHQ, 14 days, DCFDA | 122798,3223 | 1557,0226 | 111,5353 | 0 | 0,05 | 1 | 116796,8672 | 128799,7774 |
| Control, 28 days, ABAP DHQ, 28 days, DCFDA | 107348,8831 | 1557,0226 | 97,50292 | 0 | 0,05 | 1 | 101347,4279 | 113350,3382 |
| Control, 28 days, ABAP DHQ, 42 days, DCFDA | 108076,0833 | 1557,0226 | 98,16342 | 0 | 0,05 | 1 | 102074,6282 | 114077,5384 |
| Control, 28 days, ABAP DHQ, 56 days, DCFDA | 123030,3073 | 1557,0226 | 111,746 | 0 | 0,05 | 1 | 117028,8522 | 129031,7624 |
| Control, 28 days, ABAP Control, 1 day, ABAP | 85483,68125 | 1557,0226 | 77,64318 | 0 | 0,05 | 1 | 79482,22612 | 91485,13638 |
| Control, 28 days, ABAP Control, 7 days, ABAP | 89464,7275 | 1557,0226 | 81,25908 | 0 | 0,05 | 1 | 83463,27237 | 95466,18263 |
| Control, 28 days, ABAP Control, 14 days, ABAP | 97465,79222 | 1557,0226 | 88,5263 | 0 | 0,05 | 1 | 91464,33709 | 103467,2473 |
| Control, 42 days, ABAP Control, 1 day, DCFDA | 74898,70528 | 1557,0226 | 68,02905 | 0 | 0,05 | 1 | 68897,25015 | 80900,16041 |
| Control, 42 days, ABAP Control, 7 days, DCFDA | 81807,61028 | 1557,0226 | 74,30427 | 0 | 0,05 | 1 | 75806,15515 | 87809,06541 |
| Control, 42 days, ABAP Control, 14 days, DCFDA | 79196,61167 | 1557,0226 | 71,93275 | 0 | 0,05 | 1 | 73195,15654 | 85198,0668 |
| Control, 42 days, ABAP Control, 28 days, DCFDA | 36848,01778 | 1557,0226 | 33,46834 | 0 | 0,05 | 1 | 30846,56265 | 42849,47291 |
| Control, 42 days, ABAP Control, 42 days, DCFDA | 66288,86333 | 1557,0226 | 60,20889 | 0 | 0,05 | 1 | 60287,4082 | 72290,31846 |
| Control, 42 days, ABAP Control, 56 days, DCFDA | 84953,49763 | 1557,0226 | 77,16162 | 0 | 0,05 | 1 | 78952,0425 | 90954,95276 |
| Control, 42 days, ABAP DHQ, 1 day, DCFDA | 84758,7005 | 1557,0226 | 76,98469 | 0 | 0,05 | 1 | 78757,24536 | 90760,15563 |
| Control, 42 days, ABAP DHQ, 7 days, DCFDA | 75238,53744 | 1557,0226 | 68,33771 | 0 | 0,05 | 1 | 69237,08231 | 81239,99258 |
| Control, 42 days, ABAP DHQ, 14 days, DCFDA | 82741,91512 | 1557,0226 | 75,15288 | 0 | 0,05 | 1 | 76740,45999 | 88743,37025 |
| Control, 42 days, ABAP DHQ, 28 days, DCFDA | 67292,4759 | 1557,0226 | 61,12046 | 0 | 0,05 | 1 | 61291,02077 | 73293,93103 |
| Control, 42 days, ABAP DHQ, 42 days, DCFDA | 68019,67611 | 1557,0226 | 61,78096 | 0 | 0,05 | 1 | 62018,22098 | 74021,13124 |
| Control, 42 days, ABAP DHQ, 56 days, DCFDA | 82973,90013 | 1557,0226 | 75,36359 | 0 | 0,05 | 1 | 76972,445 | 88975,35526 |
| Control, 42 days, ABAP Control, 1 day, ABAP | 45427,27403 | 1557,0226 | 41,26072 | 0 | 0,05 | 1 | 39425,8189 | 51428,72916 |
| Control, 42 days, ABAP Control, 7 days, ABAP | 49408,32028 | 1557,0226 | 44,87662 | 0 | 0,05 | 1 | 43406,86515 | 55409,77541 |
| Control, 42 days, ABAP Control, 14 days, ABAP | 57409,385 | 1557,0226 | 52,14384 | 0 | 0,05 | 1 | 51407,92987 | 63410,84013 |
| Control, 42 days, ABAP Control, 28 days, ABAP | -40056,40722 | 1557,0226 | 36,38246 | 0 | 0,05 | 1 | -46057,86235 | -34054,95209 |
| Control, 56 days, ABAP Control, 1 day, DCFDA | 11937,06689 | 1557,0226 | 10,84221 | 1,00657E-6 | 0,05 | 1 | 5935,61176 | 17938,52203 |
| Control, 56 days, ABAP Control, 7 days, DCFDA | 18845,97189 | 1557,0226 | 17,11743 | 0 | 0,05 | 1 | 12844,51676 | 24847,42703 |
| Control, 56 days, ABAP Control, 14 days, DCFDA | 16234,97328 | 1557,0226 | 14,74591 | 0 | 0,05 | 1 | 10233,51815 | 22236,42841 |
| Control, 56 days, ABAP Control, 28 days, DCFDA | -26113,62061 | 1557,0226 | 23,7185 | 0 | 0,05 | 1 | -32115,07574 | -20112,16547 |
| Control, 56 days, ABAP Control, 42 days, DCFDA | 3327,22495 | 1557,0226 | 3,02205 | 0,87991 | 0,05 | 0 | -2674,23018 | 9328,68008 |
| Control, 56 days, ABAP Control, 56 days, DCFDA | 21991,85924 | 1557,0226 | 19,97478 | 0 | 0,05 | 1 | 15990,40411 | 27993,31437 |
| Control, 56 days, ABAP DHQ, 1 day, DCFDA | 21797,06211 | 1557,0226 | 19,79785 | 0 | 0,05 | 1 | 15795,60698 | 27798,51724 |
| Control, 56 days, ABAP DHQ, 7 days, DCFDA | 12276,89906 | 1557,0226 | 11,15087 | 1,62917E-7 | 0,05 | 1 | 6275,44393 | 18278,35419 |
| Control, 56 days, ABAP DHQ, 14 days, DCFDA | 19780,27674 | 1557,0226 | 17,96604 | 0 | 0,05 | 1 | 13778,8216 | 25781,73187 |
| Control, 56 days, ABAP DHQ, 28 days, DCFDA | 4330,83752 | 1557,0226 | 3,93362 | 0,47405 | 0,05 | 0 | -1670,61716 | 10332,29265 |
| Control, 56 days, ABAP DHQ, 42 days, DCFDA | 5058,03773 | 1557,0226 | 4,59412 | 0,2085 | 0,05 | 0 | -943,41694 | 11059,49286 |
| Control, 56 days, ABAP DHQ, 56 days, DCFDA | 20012,26175 | 1557,0226 | 18,17675 | 0 | 0,05 | 1 | 14010,80662 | 26013,71688 |
| Control, 56 days, ABAP Control, 1 day, ABAP | -17534,36436 | 1557,0226 | 15,92612 | 0 | 0,05 | 1 | -23535,81949 | -11532,90922 |
| Control, 56 days, ABAP Control, 7 days, ABAP | -13553,31811 | 1557,0226 | 12,31022 | 3,1728E-9 | 0,05 | 1 | -19554,77324 | -7551,86297 |
| Control, 56 days, ABAP Control, 14 days, ABAP | -5552,25338 | 1557,0226 | 5,043 | 0,10305 | 0,05 | 0 | -11553,70851 | 449,20175 |
| Control, 56 days, ABAP Control, 28 days, ABAP | -103018,0456 | 1557,0226 | 93,5693 | 0 | 0,05 | 1 | -109019,5007 | -97016,59047 |
| Control, 56 days, ABAP Control, 42 days, ABAP | -62961,63838 | 1557,0226 | 57,18684 | 0 | 0,05 | 1 | -68963,09351 | -56960,18325 |
| DHQ, 1 day, ABAP Control, 1 day, DCFDA | -5260,11449 | 1557,0226 | 4,77766 | 0,15834 | 0,05 | 0 | -11261,56962 | 741,34064 |
| DHQ, 1 day, ABAP Control, 7 days, DCFDA | 1648,79051 | 1557,0226 | 1,49756 | 0,99999 | 0,05 | 0 | -4352,66462 | 7650,24564 |
| DHQ, 1 day, ABAP Control, 14 days, DCFDA | -962,20811 | 1557,0226 | 0,87396 | 1 | 0,05 | 0 | -6963,66323 | 5039,24703 |
| DHQ, 1 day, ABAP Control, 28 days, DCFDA | -43310,80199 | 1557,0226 | 39,33837 | 0 | 0,05 | 1 | -49312,25712 | -37309,34686 |
| DHQ, 1 day, ABAP Control, 42 days, DCFDA | -13869,95644 | 1557,0226 | 12,59781 | 0 | 0,05 | 1 | -19871,41157 | -7868,50131 |
| DHQ, 1 day, ABAP Control, 56 days, DCFDA | 4794,67786 | 1557,0226 | 4,35491 | 0,29009 | 0,05 | 0 | -1206,77728 | 10796,13299 |
| DHQ, 1 day, ABAP DHQ, 1 day, DCFDA | 4599,88072 | 1557,0226 | 4,17798 | 0,36181 | 0,05 | 0 | -1401,57441 | 10601,33586 |
| DHQ, 1 day, ABAP DHQ, 7 days, DCFDA | -4920,28233 | 1557,0226 | 4,469 | 0,24893 | 0,05 | 0 | -10921,73746 | 1081,1728 |
| DHQ, 1 day, ABAP DHQ, 14 days, DCFDA | 2583,09535 | 1557,0226 | 2,34618 | 0,9898 | 0,05 | 0 | -3418,35978 | 8584,55048 |
| DHQ, 1 day, ABAP DHQ, 28 days, DCFDA | -12866,34387 | 1557,0226 | 11,68625 | 1,04913E-7 | 0,05 | 1 | -18867,799 | -6864,88874 |
| DHQ, 1 day, ABAP DHQ, 42 days, DCFDA | -12139,14366 | 1557,0226 | 11,02575 | 1,98114E-7 | 0,05 | 1 | -18140,59879 | -6137,68853 |
| DHQ, 1 day, ABAP DHQ, 56 days, DCFDA | 2815,08036 | 1557,0226 | 2,55688 | 0,97358 | 0,05 | 0 | -3186,37477 | 8816,53549 |
| DHQ, 1 day, ABAP Control, 1 day, ABAP | -34731,54574 | 1557,0226 | 31,54599 | 0 | 0,05 | 1 | -40733,00087 | -28730,09061 |
| DHQ, 1 day, ABAP Control, 7 days, ABAP | -30750,49949 | 1557,0226 | 27,93008 | 3,66979E-8 | 0,05 | 1 | -36751,95462 | -24749,04436 |
| DHQ, 1 day, ABAP Control, 14 days, ABAP | -22749,43477 | 1557,0226 | 20,66287 | 0 | 0,05 | 1 | -28750,88989 | -16747,97964 |
| DHQ, 1 day, ABAP Control, 28 days, ABAP | -120215,2269 | 1557,0226 | 109,1891 | 0 | 0,05 | 1 | -126216,6821 | -114213,7718 |
| DHQ, 1 day, ABAP Control, 42 days, ABAP | -80158,81977 | 1557,0226 | 72,80671 | 0 | 0,05 | 1 | -86160,2749 | -74157,36464 |
| DHQ, 1 day, ABAP Control, 56 days, ABAP | -17197,18139 | 1557,0226 | 15,61987 | 0 | 0,05 | 1 | -23198,63652 | -11195,72626 |
| DHQ, 7 days, ABAP Control, 1 day, DCFDA | 12861,01517 | 1557,0226 | 11,68141 | 1,05151E-7 | 0,05 | 1 | 6859,56004 | 18862,4703 |
| DHQ, 7 days, ABAP Control, 7 days, DCFDA | 19769,92017 | 1557,0226 | 17,95664 | 0 | 0,05 | 1 | 13768,46504 | 25771,3753 |
| DHQ, 7 days, ABAP Control, 14 days, DCFDA | 17158,92156 | 1557,0226 | 15,58512 | 0 | 0,05 | 1 | 11157,46642 | 23160,37669 |
| DHQ, 7 days, ABAP Control, 28 days, DCFDA | -25189,67233 | 1557,0226 | 22,87929 | 0 | 0,05 | 1 | -31191,12746 | -19188,2172 |
| DHQ, 7 days, ABAP Control, 42 days, DCFDA | 4251,17322 | 1557,0226 | 3,86126 | 0,50936 | 0,05 | 0 | -1750,28191 | 10252,62835 |
| DHQ, 7 days, ABAP Control, 56 days, DCFDA | 22915,80752 | 1557,0226 | 20,81399 | 0 | 0,05 | 1 | 16914,35238 | 28917,26265 |
| DHQ, 7 days, ABAP DHQ, 1 day, DCFDA | 22721,01038 | 1557,0226 | 20,63706 | 0 | 0,05 | 1 | 16719,55525 | 28722,46552 |
| DHQ, 7 days, ABAP DHQ, 7 days, DCFDA | 13200,84733 | 1557,0226 | 11,99007 | 1,06877E-8 | 0,05 | 1 | 7199,3922 | 19202,30246 |
| DHQ, 7 days, ABAP DHQ, 14 days, DCFDA | 20704,22501 | 1557,0226 | 18,80525 | 0 | 0,05 | 1 | 14702,76988 | 26705,68014 |
| DHQ, 7 days, ABAP DHQ, 28 days, DCFDA | 5254,78579 | 1557,0226 | 4,77282 | 0,15953 | 0,05 | 0 | -746,66934 | 11256,24092 |
| DHQ, 7 days, ABAP DHQ, 42 days, DCFDA | 5981,986 | 1557,0226 | 5,43332 | 0,05167 | 0,05 | 0 | -19,46913 | 11983,44113 |
| DHQ, 7 days, ABAP DHQ, 56 days, DCFDA | 20936,21002 | 1557,0226 | 19,01596 | 0 | 0,05 | 1 | 14934,75489 | 26937,66515 |
| DHQ, 7 days, ABAP Control, 1 day, ABAP | -16610,41608 | 1557,0226 | 15,08692 | 0 | 0,05 | 1 | -22611,87121 | -10608,96095 |
| DHQ, 7 days, ABAP Control, 7 days, ABAP | -12629,36983 | 1557,0226 | 11,47101 | 1,1922E-7 | 0,05 | 1 | -18630,82496 | -6627,9147 |
| DHQ, 7 days, ABAP Control, 14 days, ABAP | -4628,30511 | 1557,0226 | 4,2038 | 0,35078 | 0,05 | 0 | -10629,76024 | 1373,15002 |
| DHQ, 7 days, ABAP Control, 28 days, ABAP | -102094,0973 | 1557,0226 | 92,7301 | 0 | 0,05 | 1 | -108095,5524 | -96092,6422 |
| DHQ, 7 days, ABAP Control, 42 days, ABAP | -62037,69011 | 1557,0226 | 56,34764 | 0 | 0,05 | 1 | -68039,14524 | -56036,23498 |
| DHQ, 7 days, ABAP Control, 56 days, ABAP | 923,94827 | 1557,0226 | 0,8392 | 1 | 0,05 | 0 | -5077,50688 | 6925,40339 |
| DHQ, 7 days, ABAP DHQ, 1 day, ABAP | 18121,12966 | 1557,0226 | 16,45907 | 0 | 0,05 | 1 | 12119,67453 | 24122,58479 |
| DHQ, 14 days, ABAP Control, 1 day, DCFDA | -3732,21206 | 1557,0226 | 3,38996 | 0,73872 | 0,05 | 0 | -9733,66179 | 2269,32847 |
| DHQ, 14 days, ABAP Control, 7 days, DCFDA | 3176,69294 | 1557,0226 | 2,88533 | 0,91738 | 0,05 | 0 | -2824,76219 | 9178,14807 |
| DHQ, 14 days, ABAP Control, 14 days, DCFDA | 565,69433 | 1557,0226 | 0,51383 | 1 | 0,05 | 0 | -5435,7608 | 6567,14946 |
| DHQ, 14 days, ABAP Control, 28 days, DCFDA | -41782,89956 | 1557,0226 | 37,95066 | 0 | 0,05 | 1 | -47784,35469 | -35781,44443 |
| DHQ, 14 days, ABAP Control, 42 days, DCFDA | -12342,05401 | 1557,0226 | 11,21005 | 1,48629E-7 | 0,05 | 1 | -18343,50914 | -6340,59888 |
| DHQ, 14 days, ABAP Control, 56 days, DCFDA | 6322,58028 | 1557,0226 | 5,74268 | 0,02865 | 0,05 | 1 | 321,12515 | 12324,03542 |
| DHQ, 14 days, ABAP DHQ, 1 day, DCFDA | 6127,78315 | 1557,0226 | 5,56575 | 0,04031 | 0,05 | 1 | 126,32802 | 12129,23828 |
| DHQ, 14 days, ABAP DHQ, 7 days, DCFDA | -3392,3799 | 1557,0226 | 3,08123 | 0,86099 | 0,05 | 0 | -9393,83503 | 2609,07523 |
| DHQ, 14 days, ABAP DHQ, 14 days, DCFDA | 4110,99778 | 1557,0226 | 3,73394 | 0,57258 | 0,05 | 0 | -1890,45735 | 10112,45291 |
| DHQ, 14 days, ABAP DHQ, 28 days, DCFDA | -11338,44144 | 1557,0226 | 10,29849 | 2,55643E-6 | 0,05 | 1 | -17339,89657 | -5336,98631 |
| DHQ, 14 days, ABAP DHQ, 42 days, DCFDA | -10611,24123 | 1557,0226 | 9,63799 | 3,56856E-6 | 0,05 | 1 | -16612,69636 | -4609,7861 |
| DHQ, 14 days, ABAP DHQ, 56 days, DCFDA | 4342,98279 | 1557,0226 | 3,94465 | 0,46873 | 0,05 | 0 | -1658,47234 | 10344,43792 |
| DHQ, 14 days, ABAP Control, 1 day, ABAP | -33203,64331 | 1557,0226 | 30,15823 | 4,59639E-8 | 0,05 | 1 | -39205,09844 | -27202,18818 |
| DHQ, 14 days, ABAP Control, 7 days, ABAP | -29222,59706 | 1557,0226 | 26,54232 | 1,50781E-7 | 0,05 | 1 | -35224,05219 | -23221,14193 |
| DHQ, 14 days, ABAP Control, 14 days, ABAP | -21221,53234 | 1557,0226 | 19,27511 | 0 | 0,05 | 1 | -27222,98747 | -15220,07721 |
| DHQ, 14 days, ABAP Control, 28 days, ABAP | -118687,3245 | 1557,0226 | 107,8014 | 0 | 0,05 | 1 | -124688,7796 | -112685,8694 |
| DHQ, 14 days, ABAP Control, 42 days, ABAP | -78630,91734 | 1557,0226 | 71,41894 | 0 | 0,05 | 1 | -84632,37246 | -72629,46221 |
| DHQ, 14 days, ABAP Control, 56 days, ABAP | -15669,27896 | 1557,0226 | 14,2321 | 0 | 0,05 | 1 | -21670,73409 | -9667,82383 |
| DHQ, 14 days, ABAP DHQ, 1 day, ABAP | 1527,90243 | 1557,0226 | 1,38776 | 1 | 0,05 | 0 | -4473,55270 | 7529,35756 |
| DHQ, 14 days, ABAP DHQ, 7 days, ABAP | -16593,22723 | 1557,0226 | 15,07131 | 0 | 0,05 | 1 | -22594,68236 | -10591,7721 |
| DHQ, 28 days, ABAP Control, 1 day, DCFDA | 11859,33313 | 1557,0226 | 10,77168 | 1,02779E-6 | 0,05 | 1 | 5857,87799 | 17860,78826 |
| DHQ, 28 days, ABAP Control, 7 days, DCFDA | 18768,23813 | 1557,0226 | 17,04683 | 0 | 0,05 | 1 | 12766,78299 | 24769,69326 |
| DHQ, 28 days, ABAP Control, 14 days, DCFDA | 16157,23951 | 1557,0226 | 14,67531 | 0 | 0,05 | 1 | 10155,78438 | 22158,69464 |
| DHQ, 28 days, ABAP Control, 28 days, DCFDA | -26191,35437 | 1557,0226 | 23,7891 | 0 | 0,05 | 1 | -32192,80951 | -20189,89924 |
| DHQ, 28 days, ABAP Control, 42 days, DCFDA | 3249,49118 | 1557,0226 | 2,95145 | 0,90035 | 0,05 | 0 | -2751,96396 | 9250,94631 |
| DHQ, 28 days, ABAP Control, 56 days, DCFDA | 21914,12547 | 1557,0226 | 19,90418 | 0 | 0,05 | 1 | 15912,67034 | 27915,5806 |
| DHQ, 28 days, ABAP DHQ, 1 day, DCFDA | 21719,32834 | 1557,0226 | 19,72725 | 0 | 0,05 | 1 | 15717,87321 | 27720,78347 |
| DHQ, 28 days, ABAP DHQ, 7 days, DCFDA | 12199,16529 | 1557,0226 | 11,08027 | 1,84136E-7 | 0,05 | 1 | 6197,71016 | 18200,62042 |
| DHQ, 28 days, ABAP DHQ, 14 days, DCFDA | 19702,54297 | 1557,0226 | 17,89544 | 0 | 0,05 | 1 | 13701,08784 | 25703,9981 |
| DHQ, 28 days, ABAP DHQ, 28 days, DCFDA | 4253,10375 | 1557,0226 | 3,86301 | 0,50849 | 0,05 | 0 | -1748,35138 | 10254,55888 |
| DHQ, 28 days, ABAP DHQ, 42 days, DCFDA | 4980,30396 | 1557,0226 | 4,52351 | 0,23073 | 0,05 | 0 | -1021,15117 | 10981,75909 |
| DHQ, 28 days, ABAP DHQ, 56 days, DCFDA | 19934,52798 | 1557,0226 | 18,10615 | 0 | 0,05 | 1 | 13933,07285 | 25935,98311 |
| DHQ, 28 days, ABAP Control, 1 day, ABAP | -17612,09812 | 1557,0226 | 15,99673 | 0 | 0,05 | 1 | -23613,55326 | -11610,64299 |
| DHQ, 28 days, ABAP Control, 7 days, ABAP | -13631,05187 | 1557,0226 | 12,38082 | 2,03945E-9 | 0,05 | 1 | -19632,50701 | -7629,59674 |
| DHQ, 28 days, ABAP Control, 14 days, ABAP | -5629,98715 | 1557,0226 | 5,11361 | 0,0914 | 0,05 | 0 | -11631,44228 | 371,46798 |
| DHQ, 28 days, ABAP Control, 28 days, ABAP | -103095,7793 | 1557,0226 | 93,63991 | 0 | 0,05 | 1 | -109097,2345 | -97094,32424 |
| DHQ, 28 days, ABAP Control, 42 days, ABAP | -63039,37215 | 1557,0226 | 57,25744 | 0 | 0,05 | 1 | -69040,82728 | -57037,91702 |
| DHQ, 28 days, ABAP Control, 56 days, ABAP | -77,73377 | 1557,0226 | 0,0706 | 1 | 0,05 | 0 | -6079,1889 | 5923,72136 |
| DHQ, 28 days, ABAP DHQ, 1 day, ABAP | 17119,44762 | 1557,0226 | 15,54926 | 0 | 0,05 | 1 | 11117,99249 | 23120,90275 |
| DHQ, 28 days, ABAP DHQ, 7 days, ABAP | -1001,68204 | 1557,0226 | 0,90981 | 1 | 0,05 | 0 | -7003,13717 | 4999,77309 |
| DHQ, 28 days, ABAP DHQ, 14 days, ABAP | 15591,54519 | 1557,0226 | 14,1615 | 0 | 0,05 | 1 | 9590,09006 | 21593,00032 |
| DHQ, 42 days, ABAP Control, 1 day, DCFDA | 6569,9125 | 1557,0226 | 5,96732 | 0,0183 | 0,05 | 1 | 568,45737 | 12571,36763 |
| DHQ, 42 days, ABAP Control, 7 days, DCFDA | 13478,8175 | 1557,0226 | 12,24255 | 0 | 0,05 | 1 | 7477,36237 | 19480,27263 |
| DHQ, 42 days, ABAP Control, 14 days, DCFDA | 10867,81889 | 1557,0226 | 9,87103 | 1,8735E-6 | 0,05 | 1 | 4866,36376 | 16869,27402 |
| DHQ, 42 days, ABAP Control, 28 days, DCFDA | -31480,775 | 1557,0226 | 28,59338 | 3,96495E-8 | 0,05 | 1 | -37482,23013 | -25479,31987 |
| DHQ, 42 days, ABAP Control, 42 days, DCFDA | -2039,92944 | 1557,0226 | 1,85283 | 0,99957 | 0,05 | 0 | -8041,38458 | 3961,52569 |
| DHQ, 42 days, ABAP Control, 56 days, DCFDA | 16624,70485 | 1557,0226 | 15,0999 | 0 | 0,05 | 1 | 10623,24972 | 22626,15998 |
| DHQ, 42 days, ABAP DHQ, 1 day, DCFDA | 16429,90772 | 1557,0226 | 14,92297 | 0 | 0,05 | 1 | 10428,45259 | 22431,36285 |
| DHQ, 42 days, ABAP DHQ, 7 days, DCFDA | 6909,74467 | 1557,0226 | 6,27599 | 0,00965 | 0,05 | 1 | 908,28954 | 12911,1998 |
| DHQ, 42 days, ABAP DHQ, 14 days, DCFDA | 14413,12234 | 1557,0226 | 13,09116 | 0 | 0,05 | 1 | 8411,66721 | 20414,57747 |
| DHQ, 42 days, ABAP DHQ, 28 days, DCFDA | -1036,31688 | 1557,0226 | 0,94127 | 1 | 0,05 | 0 | -7037,77201 | 4965,13826 |
| DHQ, 42 days, ABAP DHQ, 42 days, DCFDA | -309,11667 | 1557,0226 | 0,28076 | 1 | 0,05 | 0 | -6310,5718 | 5692,33846 |
| DHQ, 42 days, ABAP DHQ, 56 days, DCFDA | 14645,10735 | 1557,0226 | 13,30187 | 0 | 0,05 | 1 | 8643,65222 | 20646,56248 |
| DHQ, 42 days, ABAP Control, 1 day, ABAP | -22901,51875 | 1557,0226 | 20,80101 | 0 | 0,05 | 1 | -28902,97388 | -16900,06362 |
| DHQ, 42 days, ABAP Control, 7 days, ABAP | -18920,4725 | 1557,0226 | 17,1851 | 0 | 0,05 | 1 | -24921,92763 | -12919,01737 |
| DHQ, 42 days, ABAP Control, 14 days, ABAP | -10919,40778 | 1557,0226 | 9,91789 | 1,90137E-6 | 0,05 | 1 | -16920,86291 | -4917,95265 |
| DHQ, 42 days, ABAP Control, 28 days, ABAP | -108385,2 | 1557,0226 | 98,44418 | 0 | 0,05 | 1 | -114386,6551 | -102383,7448 |
| DHQ, 42 days, ABAP Control, 42 days, ABAP | -68328,79278 | 1557,0226 | 62,06172 | 0 | 0,05 | 1 | -74330,24791 | -62327,33765 |
| DHQ, 42 days, ABAP Control, 56 days, ABAP | -5367,15439 | 1557,0226 | 4,87488 | 0,13584 | 0,05 | 0 | -11368,60953 | 634,30074 |
| DHQ, 42 days, ABAP DHQ, 1 day, ABAP | 11830,02699 | 1557,0226 | 10,74499 | 1,03739E-6 | 0,05 | 1 | 5828,57186 | 17831,48212 |
| DHQ, 42 days, ABAP DHQ, 7 days, ABAP | -6291,10267 | 1557,0226 | 5,71409 | 0,0303 | 0,05 | 1 | -12292,5578 | -289,64754 |
| DHQ, 42 days, ABAP DHQ, 14 days, ABAP | 10302,12456 | 1557,0226 | 9,35722 | 7,07774E-6 | 0,05 | 1 | 4300,66943 | 16303,57969 |
| DHQ, 42 days, ABAP DHQ, 28 days, ABAP | -5289,42063 | 1557,0226 | 4,80428 | 0,15191 | 0,05 | 0 | -11290,87576 | 712,03451 |
| DHQ, 56 days, ABAP Control, 1 day, DCFDA | -2934,4674 | 1557,0226 | 2,66532 | 0,95986 | 0,05 | 0 | -8935,92253 | 3066,98773 |
| DHQ, 56 days, ABAP Control, 7 days, DCFDA | 3974,4376 | 1557,0226 | 3,6099 | 0,63422 | 0,05 | 0 | -2027,01753 | 9975,89273 |
| DHQ, 56 days, ABAP Control, 14 days, DCFDA | 1363,43899 | 1557,0226 | 1,23839 | 1 | 0,05 | 0 | -4638,01614 | 7364,89412 |
| DHQ, 56 days, ABAP Control, 28 days, DCFDA | -40985,1549 | 1557,0226 | 37,22602 | 0 | 0,05 | 1 | -46986,61003 | -34983,69977 |
| DHQ, 56 days, ABAP Control, 42 days, DCFDA | -11544,30935 | 1557,0226 | 10,48547 | 6,69492E-7 | 0,05 | 1 | -17545,76448 | -5542,85422 |
| DHQ, 56 days, ABAP Control, 56 days, DCFDA | 7120,32495 | 1557,0226 | 6,46725 | 0,00641 | 0,05 | 1 | 1118,86982 | 13121,78008 |
| DHQ, 56 days, ABAP DHQ, 1 day, DCFDA | 6925,52782 | 1557,0226 | 6,29032 | 0,00936 | 0,05 | 1 | 924,07268 | 12926,98295 |
| DHQ, 56 days, ABAP DHQ, 7 days, DCFDA | -2594,63524 | 1557,0226 | 2,35666 | 0,98926 | 0,05 | 0 | -8596,09037 | 3406,8199 |
| DHQ, 56 days, ABAP DHQ, 14 days, DCFDA | 4908,74244 | 1557,0226 | 4,45852 | 0,25254 | 0,05 | 0 | -1092,71269 | 10910,19757 |
| DHQ, 56 days, ABAP DHQ, 28 days, DCFDA | -10540,69678 | 1557,0226 | 9,57391 | 4,1702E-6 | 0,05 | 1 | -16542,15191 | -4539,24165 |
| DHQ, 56 days, ABAP DHQ, 42 days, DCFDA | -9813,49657 | 1557,0226 | 8,91341 | 2,09255E-5 | 0,05 | 1 | -15814,9517 | -3812,04144 |
| DHQ, 56 days, ABAP DHQ, 56 days, DCFDA | 5140,72745 | 1557,0226 | 4,66922 | 0,18675 | 0,05 | 0 | -860,72768 | 11142,18258 |
| DHQ, 56 days, ABAP Control, 1 day, ABAP | -32405,89865 | 1557,0226 | 29,43365 | 4,31514E-8 | 0,05 | 1 | -38407,35378 | -26404,44352 |
| DHQ, 56 days, ABAP Control, 7 days, ABAP | -28424,8524 | 1557,0226 | 25,81774 | 7,90399E-7 | 0,05 | 1 | -34426,30753 | -22423,39727 |
| DHQ, 56 days, ABAP Control, 14 days, ABAP | -20423,78768 | 1557,0226 | 18,55053 | 0 | 0,05 | 1 | -26425,24281 | -14422,33255 |
| DHQ, 56 days, ABAP Control, 28 days, ABAP | -117889,5799 | 1557,0226 | 107,0768 | 0 | 0,05 | 1 | -123891,0350 | -111888,1247 |
| DHQ, 56 days, ABAP Control, 42 days, ABAP | -77833,17268 | 1557,0226 | 70,69437 | 0 | 0,05 | 1 | -83834,62781 | -71831,71755 |
| DHQ, 56 days, ABAP Control, 56 days, ABAP | -14871,5343 | 1557,0226 | 13,50753 | 0 | 0,05 | 1 | -20872,98943 | -8870,07916 |
| DHQ, 56 days, ABAP DHQ, 1 day, ABAP | 2325,64709 | 1557,0226 | 2,11234 | 0,99725 | 0,05 | 0 | -3675,80804 | 8327,10222 |
| DHQ, 56 days, ABAP DHQ, 7 days, ABAP | -15795,48257 | 1557,0226 | 14,34673 | 0 | 0,05 | 1 | -21796,9377 | -9794,02744 |
| DHQ, 56 days, ABAP DHQ, 14 days, ABAP | 797,74466 | 1557,0226 | 0,72458 | 1 | 0,05 | 0 | -5203,71047 | 6799,19979 |
| DHQ, 56 days, ABAP DHQ, 28 days, ABAP | -14793,80053 | 1557,0226 | 13,43692 | 0 | 0,05 | 1 | -20795,25566 | -8792,3454 |
| DHQ, 56 days, ABAP DHQ, 42 days, ABAP | -9504,3799 | 1557,0226 | 8,63264 | 4,15343E-5 | 0,05 | 1 | -15505,83503 | -3502,92477 |

*Grouping Letters*

*Bonferroni Test*

| | Mean | Groups | | | | | | | | | |
|---|---|---|---|---|---|---|---|---|---|---|---|
| Control, 28 days, ABAP | 133754,725 | A | | | | | | | | | |
| Control, 42 days, ABAP | 93698,31778 | | B | | | | | | | | |
| Control, 28 days, DCFDA | 56850,3 | | | C | | | | | | | |
| Control, 1 day, ABAP | 48271,04375 | | | | D | | | | | | |
| Control, 7 days, ABAP | 44289,9975 | | | | D | | | | | | |
| Control, 14 days, ABAP | 36288,93278 | | | | | E | | | | | |
| DHQ, 7 days, ABAP | 31660,62767 | | | | | E | F | | | | |
| Control, 56 days, ABAP | 30736,67939 | | | | | E | F | | | | |
| DHQ, 28 days, ABAP | 30658,94563 | | | | | E | F | | | | |
| Control, 42 days, DCFDA | 27409,45444 | | | | | | F | | | | |
| DHQ, 28 days, DCFDA | 26405,84188 | | | | | | F | | | | |
| DHQ, 42 days, DCFDA | 25678,64167 | | | | | | F | | | | |
| DHQ, 42 days, ABAP | 25369,525 | | | | | | F | | | | |
| Control, 1 day, DCFDA | 18799,6125 | | | | | | | G | | | |
| DHQ, 7 days, DCFDA | 18459,78033 | | | | | | | G | | | |
| DHQ, 56 days, ABAP | 15865,1451 | | | | | | | G | H | | |
| DHQ, 14 days, ABAP | 15067,40044 | | | | | | | G | H | I | |
| Control, 14 days, DCFDA | 14501,70611 | | | | | | | G | H | I | J |
| DHQ, 1 day, ABAP | 13539,49801 | | | | | | | G | H | I | J |
| Control, 7 days, DCFDA | 11890,7075 | | | | | | | | H | I | J |
| DHQ, 14 days, DCFDA | 10956,40266 | | | | | | | | H | I | J |
| DHQ, 56 days, DCFDA | 10724,41765 | | | | | | | | H | I | J |
| DHQ, 1 day, DCFDA | 8939,61728 | | | | | | | | | I | J |
| Control, 56 days, DCFDA | 8744,82015 | | | | | | | | | | J |

Means that do not share a letter are significantly different.

*Scheffe Test*

| | Mean | Groups | | | | | | | | | | |
|---|---|---|---|---|---|---|---|---|---|---|---|---|
| Control, 28 days, ABAP | 133754,725 | A | | | | | | | | | | |
| Control, 42 days, ABAP | 93698,31778 | | B | | | | | | | | | |
| Control, 28 days, DCFDA | 56850,3 | | | C | | | | | | | | |
| Control, 1 day, ABAP | 48271,04375 | | | C | D | | | | | | | |
| Control, 7 days, ABAP | 44289,9975 | | | | D | E | | | | | | |
| Control, 14 days, ABAP | 36288,93278 | | | | | E | F | | | | | |
| DHQ, 7 days, ABAP | 31660,62767 | | | | | | F | G | | | | |
| Control, 56 days, ABAP | 30736,67939 | | | | | | F | G | | | | |
| DHQ, 28 days, ABAP | 30658,94563 | | | | | | F | G | | | | |
| Control, 42 days, DCFDA | 27409,45444 | | | | | | F | G | H | | | |
| DHQ, 28 days, DCFDA | 26405,84188 | | | | | | F | G | H | | | |
| DHQ, 42 days, DCFDA | 25678,64167 | | | | | | | G | H | I | | |
| DHQ, 42 days, ABAP | 25369,525 | | | | | | | G | H | I | | |
| Control, 1 day, DCFDA | 18799,6125 | | | | | | | | H | I | J | |
| DHQ, 7 days, DCFDA | 18459,78033 | | | | | | | | H | I | J | K |
| DHQ, 56 days, ABAP | 15865,1451 | | | | | | | | | I | J | K |
| DHQ, 14 days, ABAP | 15067,40044 | | | | | | | | | | J | K |
| Control, 14 days, DCFDA | 14501,70611 | | | | | | | | | | J | K |
| DHQ, 1 day, ABAP | 13539,49801 | | | | | | | | | | J | K |
| Control, 7 days, DCFDA | 11890,7075 | | | | | | | | | | J | K |
| DHQ, 14 days, DCFDA | 10956,40266 | | | | | | | | | | J | K |
| DHQ, 56 days, DCFDA | 10724,41765 | | | | | | | | | | J | K |
| DHQ, 1 day, DCFDA | 8939,61728 | | | | | | | | | | J | K |
| Control, 56 days, DCFDA | 8744,82015 | | | | | | | | | | | K |

Means that do not share a letter are significantly different.

*Tukey Test*

| | Mean | Groups | | | | | | | | | |
|---|---|---|---|---|---|---|---|---|---|---|---|
| Control, 28 days, ABAP | 133754,725 | A | | | | | | | | | |
| Control, 42 days, ABAP | 93698,31778 | | B | | | | | | | | |
| Control, 28 days, DCFDA | 56850,3 | | | C | | | | | | | |
| Control, 1 day, ABAP | 48271,04375 | | | | D | | | | | | |
| Control, 7 days, ABAP | 44289,9975 | | | | D | | | | | | |
| Control, 14 days, ABAP | 36288,93278 | | | | | E | | | | | |
| DHQ, 7 days, ABAP | 31660,62767 | | | | | E | F | | | | |
| Control, 56 days, ABAP | 30736,67939 | | | | | E | F | G | | | |
| DHQ, 28 days, ABAP | 30658,94563 | | | | | E | F | G | | | |
| Control, 42 days, DCFDA | 27409,45444 | | | | | | F | G | | | |
| DHQ, 28 days, DCFDA | 26405,84188 | | | | | | F | G | | | |
| DHQ, 42 days, DCFDA | 25678,64167 | | | | | | F | G | | | |
| DHQ, 42 days, ABAP | 25369,525 | | | | | | | G | | | |
| Control, 1 day, DCFDA | 18799,6125 | | | | | | | | H | | |
| DHQ, 7 days, DCFDA | 18459,78033 | | | | | | | | H | | |
| DHQ, 56 days, ABAP | 15865,1451 | | | | | | | | H | I | |
| DHQ, 14 days, ABAP | 15067,40044 | | | | | | | | H | I | |
| Control, 14 days, DCFDA | 14501,70611 | | | | | | | | H | I | J |
| DHQ, 1 day, ABAP | 13539,49801 | | | | | | | | H | I | J |
| Control, 7 days, DCFDA | 11890,7075 | | | | | | | | | I | J |
| DHQ, 14 days, DCFDA | 10956,40266 | | | | | | | | | I | J |
| DHQ, 56 days, DCFDA | 10724,41765 | | | | | | | | | I | J |
| DHQ, 1 day, DCFDA | 8939,61728 | | | | | | | | | | J |
| Control, 56 days, DCFDA | 8744,82015 | | | | | | | | | | J |

Means that do not share a letter are significantly different.

Sig equals 1 indicates that the difference of the means is significant at the 0,05 level.
Sig equals 0 indicates that the difference of the means is not significant at the 0,05 level.

*Powers*

| | Alpha | Sample Size | Power |
|---|---|---|---|
| Actual Power | 0,05 | 72 | 1 |

## ANOVAOneWay (11.11.2021 23:30:4

### Notes

| | |
|---|---|
| Description | Perform One-Way ANOVA |
| User Name | maxim |
| Operation Time | 11.11.2021 23:30:49 |
| Report Status | New Analysis Report |
| Data Filter | No |

### Input Data

| | Data | Range |
|---|---|---|
| DHQ, 1 day, AAPH | [DataANOVALong]"ROS AAPH"!A"DHQ, 1 day, AAPH" | [1:3] |
| DHQ, 7 days, AAPH | [DataANOVALong]"ROS AAPH"!B"DHQ, 7 days, AAPH" | [1:3] |
| DHQ, 14 days, AAPH | [DataANOVALong]"ROS AAPH"!C"DHQ, 14 days, AAPH" | [1:3] |
| DHQ, 28 days, AAPH | [DataANOVALong]"ROS AAPH"!D"DHQ, 28 days, AAPH" | [1:3] |
| DHQ, 42 days, AAPH | [DataANOVALong]"ROS AAPH"!E"DHQ, 42 days, AAPH" | [1:3] |
| DHQ, 56 days, AAPH | [DataANOVALong]"ROS AAPH"!F"DHQ, 56 days, AAPH" | [1:3] |

### Descriptive Statistics

| | N Analysis | N Missing | Mean | Standard Deviation | SE of Mean |
|---|---|---|---|---|---|
| DHQ, 1 day, AAPH | 3 | 0 | 0,28055 | 0,00473 | 0,00273 |
| DHQ, 7 days, AAPH | 3 | 0 | 0,71546 | 0,02179 | 0,01258 |
| DHQ, 14 days, AAPH | 3 | 0 | 0,41771 | 0,04821 | 0,02783 |
| DHQ, 28 days, AAPH | 3 | 0 | 0,22936 | 0,00544 | 0,00314 |
| DHQ, 42 days, AAPH | 3 | 0 | 0,27087 | 0,01522 | 0,00879 |
| DHQ, 56 days, AAPH | 3 | 0 | 0,51762 | 0,05133 | 0,02963 |

### ANOVA

#### Overall ANOVA

| | DF | Sum of Squares | Mean Square | F Value | Prob>F |
|---|---|---|---|---|---|
| Model | 5 | 0,52067 | 0,10413 | 109,28512 | 1,40414E-9 |
| Error | 12 | 0,01143 | 9,52857E-4 | | |
| Total | 17 | 0,5321 | | | |

Null Hypothesis: The means of all levels are equal.
Alternative Hypothesis: The means of one or more levels are different.
At the 0.05 level, the population means are significantly different.

#### Fit Statistics

| | R-Square | Coeff Var | Root MSE | Data Mean |
|---|---|---|---|---|
| | 0,97851 | 0,07617 | 0,03087 | 0,40526 |

#### Means Comparisons

##### Bonferroni Test

| | MeanDiff | SEM | t Value | Prob | Alpha | Sig | LCL | UCL |
|---|---|---|---|---|---|---|---|---|
| DHQ, 7 days, AAPH DHQ, 1 day, AAPH | 0,43491 | 0,0252 | 17,25565 | 1,16331E-8 | 0,05 | 1 | 0,34294 | 0,52688 |
| DHQ, 14 days, AAPH DHQ, 1 day, AAPH | 0,13716 | 0,0252 | 5,44185 | 0,00224 | 0,05 | 1 | 0,04519 | 0,22912 |
| DHQ, 14 days, AAPH DHQ, 7 days, AAPH | -0,29775 | 0,0252 | -11,8138 | 8,63297E-7 | 0,05 | 1 | -0,38972 | -0,20579 |
| DHQ, 28 days, AAPH DHQ, 1 day, AAPH | -0,05119 | 0,0252 | -2,03086 | 0,97545 | 0,05 | 0 | -0,14315 | 0,04078 |
| DHQ, 28 days, AAPH DHQ, 7 days, AAPH | -0,4861 | 0,0252 | -19,28651 | 3,19602E-9 | 0,05 | 1 | -0,57806 | -0,39413 |
| DHQ, 28 days, AAPH DHQ, 14 days, AAPH | -0,18834 | 0,0252 | -7,47271 | 1,12546E-4 | 0,05 | 1 | -0,28031 | -0,09638 |
| DHQ, 42 days, AAPH DHQ, 1 day, AAPH | -0,00968 | 0,0252 | -0,38389 | 1 | 0,05 | 0 | -0,10164 | 0,08229 |
| DHQ, 42 days, AAPH DHQ, 7 days, AAPH | -0,44459 | 0,0252 | -17,63954 | 9,01711E-9 | 0,05 | 1 | -0,53655 | -0,35262 |
| DHQ, 42 days, AAPH DHQ, 14 days, AAPH | -0,14683 | 0,0252 | -5,82574 | 0,00122 | 0,05 | 1 | -0,2388 | -0,05487 |
| DHQ, 42 days, AAPH DHQ, 28 days, AAPH | 0,04151 | 0,0252 | 1,64697 | 1 | 0,05 | 0 | -0,05046 | 0,13348 |
| DHQ, 56 days, AAPH DHQ, 1 day, AAPH | 0,23707 | 0,0252 | 9,40608 | 1,03671E-5 | 0,05 | 1 | 0,1451 | 0,32904 |
| DHQ, 56 days, AAPH DHQ, 7 days, AAPH | -0,19784 | 0,0252 | -7,84957 | 6,84595E-5 | 0,05 | 1 | -0,28981 | -0,10587 |
| DHQ, 56 days, AAPH DHQ, 14 days, AAPH | 0,09991 | 0,0252 | 3,96423 | 0,02818 | 0,05 | 1 | 0,00795 | 0,19188 |
| DHQ, 56 days, AAPH DHQ, 28 days, AAPH | 0,28826 | 0,0252 | 11,43694 | 1,23683E-6 | 0,05 | 1 | 0,19629 | 0,38022 |
| DHQ, 56 days, AAPH DHQ, 42 days, AAPH | 0,24675 | 0,0252 | 9,78997 | 6,75354E-6 | 0,05 | 1 | 0,15478 | 0,33871 |

##### Scheffe Test

| | MeanDiff | SEM | F Value | Prob | Alpha | Sig | LCL | UCL |
|---|---|---|---|---|---|---|---|---|
| DHQ, 7 days, AAPH DHQ, 1 day, AAPH | 0,43491 | 0,0252 | 59,55149 | 4,71181E-8 | 0,05 | 1 | 0,33559 | 0,53423 |
| DHQ, 14 days, AAPH DHQ, 1 day, AAPH | 0,13716 | 0,0252 | 5,92275 | 0,00551 | 0,05 | 1 | 0,03783 | 0,23648 |
| DHQ, 14 days, AAPH DHQ, 7 days, AAPH | -0,29775 | 0,0252 | 27,91317 | 3,25044E-6 | 0,05 | 1 | -0,39708 | -0,19843 |
| DHQ, 28 days, AAPH DHQ, 1 day, AAPH | -0,05119 | 0,0252 | 0,82488 | 0,55546 | 0,05 | 0 | -0,15051 | 0,04814 |
| DHQ, 28 days, AAPH DHQ, 7 days, AAPH | -0,4861 | 0,0252 | 74,39389 | 1,31174E-8 | 0,05 | 1 | -0,58542 | -0,38677 |
| DHQ, 28 days, AAPH DHQ, 14 days, AAPH | -0,18834 | 0,0252 | 11,16828 | 3,51028E-4 | 0,05 | 1 | -0,28766 | -0,08902 |
| DHQ, 42 days, AAPH DHQ, 1 day, AAPH | -0,00968 | 0,0252 | 0,02947 | 0,99945 | 0,05 | 0 | -0,109 | 0,08965 |
| DHQ, 42 days, AAPH DHQ, 7 days, AAPH | -0,44459 | 0,0252 | 62,23065 | 3,66263E-8 | 0,05 | 1 | -0,54391 | -0,34526 |
| DHQ, 42 days, AAPH DHQ, 14 days, AAPH | -0,14683 | 0,0252 | 6,78784 | 0,00319 | 0,05 | 1 | -0,24615 | -0,04751 |
| DHQ, 42 days, AAPH DHQ, 28 days, AAPH | 0,04151 | 0,0252 | 0,5425 | 0,7411 | 0,05 | 0 | -0,05781 | 0,14083 |
| DHQ, 56 days, AAPH DHQ, 1 day, AAPH | 0,23707 | 0,0252 | 17,69486 | 3,61969E-5 | 0,05 | 1 | 0,13775 | 0,33639 |
| DHQ, 56 days, AAPH DHQ, 7 days, AAPH | -0,19784 | 0,0252 | 12,32315 | 2,19551E-4 | 0,05 | 1 | -0,29716 | -0,09852 |
| DHQ, 56 days, AAPH DHQ, 14 days, AAPH | 0,09991 | 0,0252 | 3,14302 | 0,04831 | 0,05 | 1 | 5,92183E-4 | 0,19924 |
| DHQ, 56 days, AAPH DHQ, 28 days, AAPH | 0,28826 | 0,0252 | 26,16071 | 4,61532E-6 | 0,05 | 1 | 0,18893 | 0,38758 |
| DHQ, 56 days, AAPH DHQ, 42 days, AAPH | 0,24675 | 0,0252 | 19,16869 | 2,39491E-5 | 0,05 | 1 | 0,14742 | 0,34607 |

##### Tukey Test

| | MeanDiff | SEM | q Value | Prob | Alpha | Sig | LCL | UCL |
|---|---|---|---|---|---|---|---|---|
| DHQ, 7 days, AAPH DHQ, 1 day, AAPH | 0,43491 | 0,0252 | 24,40317 | 2,39693E-7 | 0,05 | 1 | 0,35025 | 0,51957 |
| DHQ, 14 days, AAPH DHQ, 1 day, AAPH | 0,13716 | 0,0252 | 7,69594 | 0,0016 | 0,05 | 1 | 0,0525 | 0,22181 |
| DHQ, 14 days, AAPH DHQ, 7 days, AAPH | -0,29775 | 0,0252 | 16,70724 | 6,03429E-7 | 0,05 | 1 | -0,38241 | -0,2131 |
| DHQ, 28 days, AAPH DHQ, 1 day, AAPH | -0,05119 | 0,0252 | 2,87207 | 0,38051 | 0,05 | 0 | -0,13584 | 0,03347 |
| DHQ, 28 days, AAPH DHQ, 7 days, AAPH | -0,4861 | 0,0252 | 27,27524 | 0 | 0,05 | 1 | -0,57075 | -0,40144 |
| DHQ, 28 days, AAPH DHQ, 14 days, AAPH | -0,18834 | 0,0252 | 10,56801 | 8,54472E-5 | 0,05 | 1 | -0,273 | -0,10368 |
| DHQ, 42 days, AAPH DHQ, 1 day, AAPH | -0,00968 | 0,0252 | 0,5429 | 0,99862 | 0,05 | 0 | -0,09433 | 0,07498 |
| DHQ, 42 days, AAPH DHQ, 7 days, AAPH | -0,44459 | 0,0252 | 24,94607 | 2,41679E-7 | 0,05 | 1 | -0,52924 | -0,35993 |
| DHQ, 42 days, AAPH DHQ, 14 days, AAPH | -0,14683 | 0,0252 | 8,23884 | 8,86505E-4 | 0,05 | 1 | -0,23149 | -0,06217 |
| DHQ, 42 days, AAPH DHQ, 28 days, AAPH | 0,04151 | 0,0252 | 2,32917 | 0,58645 | 0,05 | 0 | -0,04315 | 0,12617 |
| DHQ, 56 days, AAPH DHQ, 1 day, AAPH | 0,23707 | 0,0252 | 13,3022 | 8,03207E-6 | 0,05 | 1 | 0,15241 | 0,32173 |
| DHQ, 56 days, AAPH DHQ, 7 days, AAPH | -0,19784 | 0,0252 | 11,10097 | 5,23284E-5 | 0,05 | 1 | -0,2825 | -0,11318 |
| DHQ, 56 days, AAPH DHQ, 14 days, AAPH | 0,09991 | 0,0252 | 5,60627 | 0,01801 | 0,05 | 1 | 0,01526 | 0,18457 |
| DHQ, 56 days, AAPH DHQ, 28 days, AAPH | 0,28826 | 0,0252 | 16,17427 | 9,01155E-7 | 0,05 | 1 | 0,2036 | 0,37291 |
| DHQ, 56 days, AAPH DHQ, 42 days, AAPH | 0,24675 | 0,0252 | 13,8451 | 5,22685E-6 | 0,05 | 1 | 0,16209 | 0,3314 |

#### Grouping Letters

##### Bonferroni Test

| | Mean | Groups | | | |
|---|---|---|---|---|---|
| DHQ, 7 days, AAPH | 0,71546 | A | | | |
| DHQ, 56 days, AAPH | 0,51762 | | B | | |
| DHQ, 14 days, AAPH | 0,41771 | | | C | |
| DHQ, 1 day, AAPH | 0,28055 | | | | D |
| DHQ, 42 days, AAPH | 0,27087 | | | | D |
| DHQ, 28 days, AAPH | 0,22936 | | | | D |

Means that do not share a letter are significantly different.

##### Scheffe Test

| | Mean | Groups | | | |
|---|---|---|---|---|---|
| DHQ, 7 days, AAPH | 0,71546 | A | | | |
| DHQ, 56 days, AAPH | 0,51762 | | B | | |
| DHQ, 14 days, AAPH | 0,41771 | | | C | |
| DHQ, 1 day, AAPH | 0,28055 | | | | D |
| DHQ, 42 days, AAPH | 0,27087 | | | | D |
| DHQ, 28 days, AAPH | 0,22936 | | | | D |

Means that do not share a letter are significantly different.

##### Tukey Test

| | Mean | Groups | | | |
|---|---|---|---|---|---|
| DHQ, 7 days, AAPH | 0,71546 | A | | | |
| DHQ, 56 days, AAPH | 0,51762 | | B | | |
| DHQ, 14 days, AAPH | 0,41771 | | | C | |
| DHQ, 1 day, AAPH | 0,28055 | | | | D |
| DHQ, 42 days, AAPH | 0,27087 | | | | D |
| DHQ, 28 days, AAPH | 0,22936 | | | | D |

Means that do not share a letter are significantly different.

Sig equals 1 indicates that the difference of the means is significant at the 0,05 level.
Sig equals 0 indicates that the difference of the means is not significant at the 0,05 level.

### Powers

| | Alpha | Sample Size | Power |
|---|---|---|---|
| Actual Power | 0,05 | 18 | 1 |

# ANOVAOneWay (27.10.2024 22:04:41)

## Notes

| | |
|---|---|
| Description | Perform One-Way ANOVA |
| User Name | yulia |
| Operation Time | 27.10.2024 22:04:41 |
| Report Status | New Analysis Report |
| Data Filter | No |

## Input Data

| | Data | Range |
|---|---|---|
| Control, 7 days | [Book1]CFU!A"Control, 7 days" | [1*:4*] |
| Control, 14 days | [Book1]CFU!B"Control, 14 days | [1*:4*] |
| Control, 28 days | [Book1]CFU!C"Control, 28 days | [1*:4*] |
| Control, 42 days | [Book1]CFU!D"Control, 42 days | [1*:10*] |
| Control, 56 days | [Book1]CFU!E"Control, 56 days | [1*:6*] |
| DHQ, 7 days | [Book1]CFU!F"DHQ, 7 days" | [1*:3*] |
| DHQ, 14 days | [Book1]CFU!G"DHQ, 14 days" | [1*:4*] |
| DHQ, 28 days | [Book1]CFU!H"DHQ, 28 days" | [1*:6*] |
| DHQ, 42 days | [Book1]CFU!I"DHQ, 42 days" | [1*:8*] |
| DHQ, 56 days | [Book1]CFU!J"DHQ, 56 days" | [1*:4*] |

## Descriptive Statistics

| | N Analysis | N Missing | Mean | Standard Deviation | SE of Mean |
|---|---|---|---|---|---|
| Control, 7 days | 4 | 0 | 0,62503 | 0,19392 | 0,09696 |
| Control, 14 days | 4 | 0 | 0,71263 | 0,07604 | 0,03802 |
| Control, 28 days | 4 | 0 | 0,34522 | 0,09571 | 0,04785 |
| Control, 42 days | 10 | 0 | 0,29247 | 0,09726 | 0,03076 |
| Control, 56 days | 6 | 0 | 0,20625 | 0,0443 | 0,01809 |
| DHQ, 7 days | 3 | 0 | 0,44197 | 0,02825 | 0,01631 |
| DHQ, 14 days | 4 | 0 | 0,47843 | 0,26684 | 0,13342 |
| DHQ, 28 days | 6 | 0 | 0,1759 | 0,07508 | 0,03065 |
| DHQ, 42 days | 8 | 0 | 0,31357 | 0,06739 | 0,02383 |
| DHQ, 56 days | 4 | 0 | 0,19392 | 0,06519 | 0,0326 |

## ANOVA

### Overall ANOVA

| | DF | Sum of Squares | Mean Square | F Value | Prob>F |
|---|---|---|---|---|---|
| Model | 9 | 1,36688 | 0,15188 | 12,08234 | 3,12101E-9 |
| Error | 43 | 0,54051 | 0,01257 | | |
| Total | 52 | 1,90739 | | | |

Null Hypothesis: The means of all levels are equal.
Alternative Hypothesis: The means of one or more levels are different.
At the 0.05 level, the population means are significantly different.

### Fit Statistics

| | R-Square | Coeff Var | Root MSE | Data Mean |
|---|---|---|---|---|
| | 0,71662 | 0,32167 | 0,11212 | 0,34855 |

### Means Comparisons

#### Bonferroni Test

| | MeanDiff | SEM | t Value | Prob | Alpha | Sig | LCL | UCL |
|---|---|---|---|---|---|---|---|---|
| Control, 14 days Control, 7 days | 0,0876 | 0,07928 | 1,10498 | 1 | 0,05 | 0 | -0,18952 | 0,36472 |
| Control, 28 days Control, 7 days | -0,2798 | 0,07928 | -3,52939 | 0,0453 | 0,05 | 1 | -0,55692 | -0,00269 |
| Control, 28 days Control, 14 da | -0,3674 | 0,07928 | -4,63437 | 0,00149 | 0,05 | 1 | -0,64452 | -0,09029 |
| Control, 42 days Control, 7 days | -0,33256 | 0,06633 | -5,0138 | 4,35382E-4 | 0,05 | 1 | -0,56441 | -0,10071 |
| Control, 42 days Control, 14 da | -0,42016 | 0,06633 | -6,33451 | 5,36376E-6 | 0,05 | 1 | -0,65201 | -0,18831 |
| Control, 42 days Control, 28 da | -0,05276 | 0,06633 | -0,79537 | 1 | 0,05 | 0 | -0,28461 | 0,1791 |
| Control, 56 days Control, 7 days | -0,41877 | 0,07237 | -5,78649 | 3,36117E-5 | 0,05 | 1 | -0,67174 | -0,1658 |
| Control, 56 days Control, 14 da | -0,50637 | 0,07237 | -6,99693 | 5,84997E-7 | 0,05 | 1 | -0,75935 | -0,2534 |
| Control, 56 days Control, 28 da | -0,13897 | 0,07237 | -1,92023 | 1 | 0,05 | 0 | -0,39194 | 0,114 |
| Control, 56 days Control, 42 da | -0,08621 | 0,0579 | -1,48908 | 1 | 0,05 | 0 | -0,28859 | 0,11617 |
| DHQ, 7 days Control, 7 days | -0,18306 | 0,08563 | -2,13777 | 1 | 0,05 | 0 | -0,48238 | 0,11626 |
| DHQ, 7 days Control, 14 days | -0,27066 | 0,08563 | -3,16078 | 0,12965 | 0,05 | 0 | -0,56998 | 0,02866 |
| DHQ, 7 days Control, 28 days | 0,09675 | 0,08563 | 1,12981 | 1 | 0,05 | 0 | -0,20258 | 0,39607 |
| DHQ, 7 days Control, 42 days | 0,1495 | 0,0738 | 2,02567 | 1 | 0,05 | 0 | -0,10848 | 0,40748 |
| DHQ, 7 days Control, 56 days | 0,23571 | 0,07928 | 2,97326 | 0,21662 | 0,05 | 0 | -0,0414 | 0,51283 |
| DHQ, 14 days Control, 7 days | -0,1466 | 0,07928 | -1,84918 | 1 | 0,05 | 0 | -0,42372 | 0,13052 |
| DHQ, 14 days Control, 14 days | -0,2342 | 0,07928 | -2,95417 | 0,22804 | 0,05 | 0 | -0,51132 | 0,04292 |
| DHQ, 14 days Control, 28 days | 0,1332 | 0,07928 | 1,68021 | 1 | 0,05 | 0 | -0,14391 | 0,41032 |
| DHQ, 14 days Control, 42 days | 0,18596 | 0,06633 | 2,8036 | 0,33987 | 0,05 | 0 | -0,04589 | 0,41781 |
| DHQ, 14 days Control, 56 days | 0,27217 | 0,07237 | 3,76081 | 0,02281 | 0,05 | 1 | 0,0192 | 0,52515 |
| DHQ, 14 days DHQ, 7 days | 0,03646 | 0,08563 | 0,42576 | 1 | 0,05 | 0 | -0,26286 | 0,33578 |
| DHQ, 28 days Control, 7 days | -0,44913 | 0,07237 | -6,20594 | 8,253E-6 | 0,05 | 1 | -0,7021 | -0,19616 |
| DHQ, 28 days Control, 14 days | -0,53673 | 0,07237 | -7,41639 | 1,45213E-7 | 0,05 | 1 | -0,7897 | -0,28376 |
| DHQ, 28 days Control, 28 days | -0,16932 | 0,07237 | -2,33969 | 1 | 0,05 | 0 | -0,4223 | 0,08365 |
| DHQ, 28 days Control, 42 days | -0,11657 | 0,0579 | -2,0134 | 1 | 0,05 | 0 | -0,31895 | 0,08581 |
| DHQ, 28 days Control, 56 days | -0,03036 | 0,06473 | -0,46897 | 1 | 0,05 | 0 | -0,25662 | 0,19591 |
| DHQ, 28 days DHQ, 7 days | -0,26607 | 0,07928 | -3,35617 | 0,07474 | 0,05 | 0 | -0,54319 | 0,01105 |
| DHQ, 28 days DHQ, 14 days | -0,30253 | 0,07237 | -4,18026 | 0,00631 | 0,05 | 1 | -0,5555 | -0,04956 |
| DHQ, 42 days Control, 7 days | -0,31145 | 0,06866 | -4,53638 | 0,00205 | 0,05 | 1 | -0,55144 | -0,07146 |
| DHQ, 42 days Control, 14 days | -0,39905 | 0,06866 | -5,81231 | 3,08329E-5 | 0,05 | 1 | -0,63905 | -0,15906 |
| DHQ, 42 days Control, 28 days | -0,03165 | 0,06866 | -0,46099 | 1 | 0,05 | 0 | -0,27164 | 0,20834 |
| DHQ, 42 days Control, 42 days | 0,02111 | 0,05318 | 0,39686 | 1 | 0,05 | 0 | -0,16479 | 0,207 |
| DHQ, 42 days Control, 56 days | 0,10732 | 0,06055 | 1,7724 | 1 | 0,05 | 0 | -0,10433 | 0,31897 |
| DHQ, 42 days DHQ, 7 days | -0,1284 | 0,0759 | -1,69158 | 1 | 0,05 | 0 | -0,39372 | 0,13692 |
| DHQ, 42 days DHQ, 14 days | -0,16485 | 0,06866 | -2,40113 | 0,93334 | 0,05 | 0 | -0,40485 | 0,07514 |
| DHQ, 42 days DHQ, 28 days | 0,13767 | 0,06055 | 2,27375 | 1 | 0,05 | 0 | -0,07398 | 0,34933 |
| DHQ, 56 days Control, 7 days | -0,4311 | 0,07928 | -5,43783 | 1,07412E-4 | 0,05 | 1 | -0,70822 | -0,15398 |
| DHQ, 56 days Control, 14 days | -0,5187 | 0,07928 | -6,54281 | 2,669E-6 | 0,05 | 1 | -0,79582 | -0,24158 |
| DHQ, 56 days Control, 28 days | -0,1513 | 0,07928 | -1,90844 | 1 | 0,05 | 0 | -0,42842 | 0,12582 |
| DHQ, 56 days Control, 42 days | -0,09854 | 0,06633 | -1,48565 | 1 | 0,05 | 0 | -0,3304 | 0,13331 |
| DHQ, 56 days Control, 56 days | -0,01233 | 0,07237 | -0,17036 | 1 | 0,05 | 0 | -0,2653 | 0,24064 |
| DHQ, 56 days DHQ, 7 days | -0,24804 | 0,08563 | -2,89668 | 0,26591 | 0,05 | 0 | -0,54737 | 0,05128 |
| DHQ, 56 days DHQ, 14 days | -0,2845 | 0,07928 | -3,58865 | 0,03807 | 0,05 | 1 | -0,56162 | -0,00738 |
| DHQ, 56 days DHQ, 28 days | 0,01803 | 0,07237 | 0,2491 | 1 | 0,05 | 0 | -0,23495 | 0,271 |
| DHQ, 56 days DHQ, 42 days | -0,11965 | 0,06866 | -1,74268 | 1 | 0,05 | 0 | -0,35964 | 0,12034 |

#### Scheffe Test

| | MeanDiff | SEM | F Value | Prob | Alpha | Sig | LCL | UCL |
|---|---|---|---|---|---|---|---|---|
| Control, 14 days Control, 7 days | 0,0876 | 0,07928 | 0,13567 | 0,99838 | 0,05 | 0 | -0,25757 | 0,43277 |
| Control, 28 days Control, 7 days | -0,2798 | 0,07928 | 1,38407 | 0,22522 | 0,05 | 0 | -0,62497 | 0,06536 |
| Control, 28 days Control, 14 da | -0,3674 | 0,07928 | 2,38638 | 0,02729 | 0,05 | 1 | -0,71257 | -0,02224 |
| Control, 42 days Control, 7 days | -0,33256 | 0,06633 | 2,79313 | 0,01133 | 0,05 | 1 | -0,62135 | -0,04377 |
| Control, 42 days Control, 14 da | -0,42016 | 0,06633 | 4,45844 | 3,63495E-4 | 0,05 | 1 | -0,70895 | -0,13137 |
| Control, 42 days Control, 28 da | -0,05276 | 0,06633 | 0,07029 | 0,99989 | 0,05 | 0 | -0,34154 | 0,23603 |
| Control, 56 days Control, 7 days | -0,41877 | 0,07237 | 3,72038 | 0,0016 | 0,05 | 1 | -0,73386 | -0,10368 |
| Control, 56 days Control, 14 da | -0,50637 | 0,07237 | 5,43968 | 5,66164E-5 | 0,05 | 1 | -0,82147 | -0,19128 |
| Control, 56 days Control, 28 da | -0,13897 | 0,07237 | 0,4097 | 0,92298 | 0,05 | 0 | -0,45406 | 0,17612 |
| Control, 56 days Control, 42 da | -0,08621 | 0,0579 | 0,24637 | 0,98502 | 0,05 | 0 | -0,33829 | 0,16586 |
| DHQ, 7 days Control, 7 days | -0,18306 | 0,08563 | 0,50778 | 0,86072 | 0,05 | 0 | -0,55588 | 0,18976 |
| DHQ, 7 days Control, 14 days | -0,27066 | 0,08563 | 1,11006 | 0,37639 | 0,05 | 0 | -0,64348 | 0,10216 |
| DHQ, 7 days Control, 28 days | 0,09675 | 0,08563 | 0,14183 | 0,99807 | 0,05 | 0 | -0,27608 | 0,46957 |
| DHQ, 7 days Control, 42 days | 0,1495 | 0,0738 | 0,45592 | 0,89567 | 0,05 | 0 | -0,17183 | 0,47083 |
| DHQ, 7 days Control, 56 days | 0,23571 | 0,07928 | 0,98225 | 0,46819 | 0,05 | 0 | -0,10945 | 0,58088 |
| DHQ, 14 days Control, 7 days | -0,1466 | 0,07928 | 0,37994 | 0,9384 | 0,05 | 0 | -0,49177 | 0,19857 |
| DHQ, 14 days Control, 14 days | -0,2342 | 0,07928 | 0,96968 | 0,47791 | 0,05 | 0 | -0,57937 | 0,11097 |
| DHQ, 14 days Control, 28 days | 0,1332 | 0,07928 | 0,31368 | 0,9662 | 0,05 | 0 | -0,21196 | 0,47837 |
| DHQ, 14 days Control, 42 days | 0,18596 | 0,06633 | 0,87335 | 0,55579 | 0,05 | 0 | -0,10283 | 0,47475 |
| DHQ, 14 days Control, 56 days | 0,27217 | 0,07237 | 1,57152 | 0,15463 | 0,05 | 0 | -0,04292 | 0,58726 |
| DHQ, 14 days DHQ, 7 days | 0,03646 | 0,08563 | 0,02014 | 1 | 0,05 | 0 | -0,33636 | 0,40928 |
| DHQ, 28 days Control, 7 days | -0,44913 | 0,07237 | 4,2793 | 5,1762E-4 | 0,05 | 1 | -0,76422 | -0,13404 |
| DHQ, 28 days Control, 14 days | -0,53673 | 0,07237 | 6,11143 | 1,70528E-5 | 0,05 | 1 | -0,85182 | -0,22164 |
| DHQ, 28 days Control, 28 days | -0,16932 | 0,07237 | 0,60824 | 0,78305 | 0,05 | 0 | -0,48442 | 0,14577 |
| DHQ, 28 days Control, 42 days | -0,11657 | 0,0579 | 0,45042 | 0,89913 | 0,05 | 0 | -0,36864 | 0,13551 |
| DHQ, 28 days Control, 56 days | -0,03036 | 0,06473 | 0,02444 | 1 | 0,05 | 0 | -0,31218 | 0,25147 |
| DHQ, 28 days DHQ, 7 days | -0,26607 | 0,07928 | 1,25154 | 0,29061 | 0,05 | 0 | -0,61124 | 0,0791 |
| DHQ, 28 days DHQ, 14 days | -0,30253 | 0,07237 | 1,94162 | 0,07117 | 0,05 | 0 | -0,61762 | 0,01256 |
| DHQ, 42 days Control, 7 days | -0,31145 | 0,06866 | 2,28653 | 0,03388 | 0,05 | 1 | -0,61038 | -0,01253 |
| DHQ, 42 days Control, 14 days | -0,39905 | 0,06866 | 3,75365 | 0,0015 | 0,05 | 1 | -0,69798 | -0,10013 |
| DHQ, 42 days Control, 28 days | -0,03165 | 0,06866 | 0,02361 | 1 | 0,05 | 0 | -0,33057 | 0,26727 |
| DHQ, 42 days Control, 42 days | 0,02111 | 0,05318 | 0,0175 | 1 | 0,05 | 0 | -0,21044 | 0,25265 |
| DHQ, 42 days Control, 56 days | 0,10732 | 0,06055 | 0,34904 | 0,95251 | 0,05 | 0 | -0,15631 | 0,37094 |
| DHQ, 42 days DHQ, 7 days | -0,1284 | 0,0759 | 0,31794 | 0,96469 | 0,05 | 0 | -0,45887 | 0,20208 |
| DHQ, 42 days DHQ, 14 days | -0,16485 | 0,06866 | 0,6406 | 0,75608 | 0,05 | 0 | -0,46378 | 0,13407 |
| DHQ, 42 days DHQ, 28 days | 0,13767 | 0,06055 | 0,57444 | 0,81037 | 0,05 | 0 | -0,12595 | 0,4013 |
| DHQ, 56 days Control, 7 days | -0,4311 | 0,07928 | 3,28556 | 0,00397 | 0,05 | 1 | -0,77627 | -0,08593 |
| DHQ, 56 days Control, 14 days | -0,5187 | 0,07928 | 4,75649 | 2,03825E-4 | 0,05 | 1 | -0,86387 | -0,17354 |
| DHQ, 56 days Control, 28 days | -0,1513 | 0,07928 | 0,40468 | 0,9257 | 0,05 | 0 | -0,49646 | 0,19387 |
| DHQ, 56 days Control, 42 days | -0,09854 | 0,06633 | 0,24524 | 0,98526 | 0,05 | 0 | -0,38733 | 0,19025 |
| DHQ, 56 days Control, 56 days | -0,01233 | 0,07237 | 0,00322 | 1 | 0,05 | 0 | -0,32742 | 0,30276 |
| DHQ, 56 days DHQ, 7 days | -0,24804 | 0,08563 | 0,93231 | 0,50744 | 0,05 | 0 | -0,62087 | 0,12478 |
| DHQ, 56 days DHQ, 14 days | -0,2845 | 0,07928 | 1,43093 | 0,20532 | 0,05 | 0 | -0,62967 | 0,06067 |
| DHQ, 56 days DHQ, 28 days | 0,01803 | 0,07237 | 0,00689 | 1 | 0,05 | 0 | -0,29707 | 0,33312 |
| DHQ, 56 days DHQ, 42 days | -0,11965 | 0,06866 | 0,33744 | 0,95729 | 0,05 | 0 | -0,41857 | 0,17928 |

#### Tukey Test

| | MeanDiff | SEM | q Value | Prob | Alpha | Sig | LCL | UCL |
|---|---|---|---|---|---|---|---|---|
| Control, 14 days Control, 7 days | 0,0876 | 0,07928 | 1,56268 | 0,98156 | 0,05 | 0 | -0,17677 | 0,35197 |
| Control, 28 days Control, 7 days | -0,2798 | 0,07928 | 4,99131 | 0,03041 | 0,05 | 1 | -0,54417 | -0,01544 |
| Control, 28 days Control, 14 da | -0,3674 | 0,07928 | 6,55399 | 0,00125 | 0,05 | 1 | -0,63177 | -0,10304 |
| Control, 42 days Control, 7 days | -0,33256 | 0,06633 | 7,09058 | 3,78552E-4 | 0,05 | 1 | -0,55375 | -0,11137 |
| Control, 42 days Control, 14 da | -0,42016 | 0,06633 | 8,95834 | 5,03174E-6 | 0,05 | 1 | -0,64135 | -0,19897 |
| Control, 42 days Control, 28 da | -0,05276 | 0,06633 | 1,12482 | 0,9983 | 0,05 | 0 | -0,27394 | 0,16843 |
| Control, 56 days Control, 7 days | -0,41877 | 0,07237 | 8,18333 | 3,07724E-5 | 0,05 | 1 | -0,66011 | -0,17744 |
| Control, 56 days Control, 14 da | -0,50637 | 0,07237 | 9,89516 | 6,80348E-7 | 0,05 | 1 | -0,74771 | -0,26504 |
| Control, 56 days Control, 28 da | -0,13897 | 0,07237 | 2,71562 | 0,65591 | 0,05 | 0 | -0,3803 | 0,10237 |
| Control, 56 days Control, 42 da | -0,08621 | 0,0579 | 2,10588 | 0,8891 | 0,05 | 0 | -0,27928 | 0,10685 |
| DHQ, 7 days Control, 7 days | -0,18306 | 0,08563 | 3,02326 | 0,51261 | 0,05 | 0 | -0,46861 | 0,10249 |
| DHQ, 7 days Control, 14 days | -0,27066 | 0,08563 | 4,47002 | 0,07621 | 0,05 | 0 | -0,55621 | 0,01489 |
| DHQ, 7 days Control, 28 days | 0,09675 | 0,08563 | 1,5978 | 0,97862 | 0,05 | 0 | -0,1888 | 0,3823 |
| DHQ, 7 days Control, 42 days | 0,1495 | 0,0738 | 2,86472 | 0,58664 | 0,05 | 0 | -0,09661 | 0,39562 |
| DHQ, 7 days Control, 56 days | 0,23571 | 0,07928 | 4,20483 | 0,11689 | 0,05 | 0 | -0,02865 | 0,50008 |
| DHQ, 14 days Control, 7 days | -0,1466 | 0,07928 | 2,61514 | 0,70122 | 0,05 | 0 | -0,41097 | 0,11777 |
| DHQ, 14 days Control, 14 days | -0,2342 | 0,07928 | 4,17782 | 0,12189 | 0,05 | 0 | -0,49857 | 0,03017 |
| DHQ, 14 days Control, 28 days | 0,1332 | 0,07928 | 2,37617 | 0,80007 | 0,05 | 0 | -0,13116 | 0,39757 |
| DHQ, 14 days Control, 42 days | 0,18596 | 0,06633 | 3,96489 | 0,16759 | 0,05 | 0 | -0,03523 | 0,40715 |
| DHQ, 14 days Control, 56 days | 0,27217 | 0,07237 | 5,31859 | 0,01633 | 0,05 | 1 | 0,03084 | 0,51351 |
| DHQ, 14 days DHQ, 7 days | 0,03646 | 0,08563 | 0,60211 | 0,99999 | 0,05 | 0 | -0,24909 | 0,32201 |
| DHQ, 28 days Control, 7 days | -0,44913 | 0,07237 | 8,77653 | 7,69702E-6 | 0,05 | 1 | -0,69046 | -0,20779 |
| DHQ, 28 days Control, 14 days | -0,53673 | 0,07237 | 10,48836 | 2,60201E-7 | 0,05 | 1 | -0,77806 | -0,2954 |
| DHQ, 28 days Control, 28 days | -0,16932 | 0,07237 | 3,30882 | 0,38589 | 0,05 | 0 | -0,41066 | 0,07201 |
| DHQ, 28 days Control, 42 days | -0,11657 | 0,0579 | 2,84737 | 0,59477 | 0,05 | 0 | -0,30964 | 0,0765 |
| DHQ, 28 days Control, 56 days | -0,03036 | 0,06473 | 0,66322 | 0,99998 | 0,05 | 0 | -0,24621 | 0,1855 |
| DHQ, 28 days DHQ, 7 days | -0,26607 | 0,07928 | 4,74634 | 0,04739 | 0,05 | 1 | -0,53044 | -0,0017 |
| DHQ, 28 days DHQ, 14 days | -0,30253 | 0,07237 | 5,91178 | 0,00493 | 0,05 | 1 | -0,54386 | -0,06119 |
| DHQ, 42 days Control, 7 days | -0,31145 | 0,06866 | 6,41541 | 0,00169 | 0,05 | 1 | -0,5404 | -0,0825 |
| DHQ, 42 days Control, 14 days | -0,39905 | 0,06866 | 8,21984 | 2,82639E-5 | 0,05 | 1 | -0,628 | -0,17011 |
| DHQ, 42 days Control, 28 days | -0,03165 | 0,06866 | 0,65194 | 0,99998 | 0,05 | 0 | -0,2606 | 0,1973 |
| DHQ, 42 days Control, 42 days | 0,02111 | 0,05318 | 0,56125 | 0,99999 | 0,05 | 0 | -0,15624 | 0,19845 |
| DHQ, 42 days Control, 56 days | 0,10732 | 0,06055 | 2,50655 | 0,74801 | 0,05 | 0 | -0,0946 | 0,30923 |
| DHQ, 42 days DHQ, 7 days | -0,1284 | 0,0759 | 2,39226 | 0,79393 | 0,05 | 0 | -0,38151 | 0,12472 |
| DHQ, 42 days DHQ, 14 days | -0,16485 | 0,06866 | 3,39571 | 0,3504 | 0,05 | 0 | -0,3938 | 0,0641 |
| DHQ, 42 days DHQ, 28 days | 0,13767 | 0,06055 | 3,21556 | 0,42579 | 0,05 | 0 | -0,06424 | 0,33959 |
| DHQ, 56 days Control, 7 days | -0,4311 | 0,07928 | 7,69025 | 9,6433E-5 | 0,05 | 1 | -0,69547 | -0,16673 |
| DHQ, 56 days Control, 14 days | -0,5187 | 0,07928 | 9,25294 | 2,62394E-6 | 0,05 | 1 | -0,78307 | -0,25433 |
| DHQ, 56 days Control, 28 days | -0,1513 | 0,07928 | 2,69894 | 0,66353 | 0,05 | 0 | -0,41567 | 0,11307 |
| DHQ, 56 days Control, 42 days | -0,09854 | 0,06633 | 2,10103 | 0,89044 | 0,05 | 0 | -0,31973 | 0,12264 |
| DHQ, 56 days Control, 56 days | -0,01233 | 0,07237 | 0,24092 | 1 | 0,05 | 0 | -0,25366 | 0,229 |
| DHQ, 56 days DHQ, 7 days | -0,24804 | 0,08563 | 4,09653 | 0,13799 | 0,05 | 0 | -0,53359 | 0,03751 |
| DHQ, 56 days DHQ, 14 days | -0,2845 | 0,07928 | 5,07511 | 0,02601 | 0,05 | 1 | -0,54887 | -0,02013 |
| DHQ, 56 days DHQ, 28 days | 0,01803 | 0,07237 | 0,35228 | 1 | 0,05 | 0 | -0,22331 | 0,25936 |
| DHQ, 56 days DHQ, 42 days | -0,11965 | 0,06866 | 2,46453 | 0,76534 | 0,05 | 0 | -0,3486 | 0,1093 |

### Grouping Letters

#### Bonferroni Test

| | Mean | Groups | | |
|---|---|---|---|---|
| Control, 14 days | 0,71263 | A | | |
| Control, 7 days | 0,62503 | A | | |
| DHQ, 14 days | 0,47843 | A | B | |
| DHQ, 7 days | 0,44197 | A | B | C |
| Control, 28 days | 0,34522 | | B | C |
| DHQ, 42 days | 0,31357 | | B | C |
| Control, 42 days | 0,29247 | | B | C |
| Control, 56 days | 0,20625 | | | C |
| DHQ, 56 days | 0,19392 | | | C |
| DHQ, 28 days | 0,1759 | | | C |

Means that do not share a letter are significantly different.

#### Scheffe Test

| | Mean | Groups | | |
|---|---|---|---|---|
| Control, 14 days | 0,71263 | A | | |
| Control, 7 days | 0,62503 | A | B | |
| DHQ, 14 days | 0,47843 | A | B | C |
| DHQ, 7 days | 0,44197 | A | B | C |
| Control, 28 days | 0,34522 | | B | C |
| DHQ, 42 days | 0,31357 | | | C |
| Control, 42 days | 0,29247 | | | C |
| Control, 56 days | 0,20625 | | | C |
| DHQ, 56 days | 0,19392 | | | C |
| DHQ, 28 days | 0,1759 | | | C |

Means that do not share a letter are significantly different.

#### Tukey Test

| | Mean | Groups | | | |
|---|---|---|---|---|---|
| Control, 14 days | 0,71263 | A | | | |
| Control, 7 days | 0,62503 | A | | | |
| DHQ, 14 days | 0,47843 | A | B | | |
| DHQ, 7 days | 0,44197 | A | B | C | |
| Control, 28 days | 0,34522 | | B | C | D |
| DHQ, 42 days | 0,31357 | | B | C | D |
| Control, 42 days | 0,29247 | | B | C | D |
| Control, 56 days | 0,20625 | | | C | D |
| DHQ, 56 days | 0,19392 | | | C | D |
| DHQ, 28 days | 0,1759 | | | | D |

Means that do not share a letter are significantly different.

Sig equals 1 indicates that the difference of the means is significant at the 0,05 level.
Sig equals 0 indicates that the difference of the means is not significant at the 0,05 level.

## Powers

| | Alpha | Sample Size | Power |
|---|---|---|---|
| Actual Power | 0,05 | 53 | 1 |

# ANOVAOneWay (27.10.2024 22:08:56)

## Notes

| | |
|---|---|
| Description | Perform One-Way ANOVA |
| User Name | yulia |
| Operation Time | 27.10.2024 22:08:56 |
| Report Status | New Analysis Report |
| Data Filter | No |

## Input Data

| | Data | Range |
|---|---|---|
| Control, 1 day | [Book1]"MTT (1)"!A"Control, 1 day" | [1*:4*] |
| Control, 7 days | [Book1]"MTT (1)"!B"Control, 7 days | [1*:4*] |
| Control, 14 days | [Book1]"MTT (1)"!C"Control, 14 day | [1*:8*] |
| Control, 28 days | [Book1]"MTT (1)"!D"Control, 28 day | [1*:4*] |
| Control, 42 days | [Book1]"MTT (1)"!E"Control, 42 day | [1*:3*] |
| Control, 56 days | [Book1]"MTT (1)"!F"Control, 56 day | [1*:3*] |
| DHQ, 1 day | [Book1]"MTT (1)"!G"DHQ, 1 day" | [1*:3*] |
| DHQ, 7 days | [Book1]"MTT (1)"!H"DHQ, 7 days" | [1*:3*] |
| DHQ, 14 days | [Book1]"MTT (1)"!I"DHQ, 14 days" | [1*:3*] |
| DHQ, 28 days | [Book1]"MTT (1)"!J"DHQ, 28 days" | [1*:4*] |
| DHQ, 42 days | [Book1]"MTT (1)"!K"DHQ, 42 days" | [1*:3*] |
| DHQ, 56 days | [Book1]"MTT (1)"!L"DHQ, 56 days" | [1*:3*] |

## Descriptive Statistics

| | N Analysis | N Missing | Mean | Standard Deviation | SE of Mean |
|---|---|---|---|---|---|
| Control, 1 day | 4 | 0 | 0,88625 | 0,44906 | 0,22453 |
| Control, 7 days | 4 | 0 | 0,35209 | 0,1273 | 0,06365 |
| Control, 14 days | 8 | 0 | 0,33778 | 0,03811 | 0,01347 |
| Control, 28 days | 4 | 0 | 0,35669 | 0,10179 | 0,0509 |
| Control, 42 days | 3 | 0 | 0,1804 | 0,0174 | 0,01005 |
| Control, 56 days | 3 | 0 | 0,15977 | 0,04877 | 0,02816 |
| DHQ, 1 day | 3 | 0 | 0,8033 | 0,41259 | 0,23821 |
| DHQ, 7 days | 3 | 0 | 0,40218 | 0,06212 | 0,03587 |
| DHQ, 14 days | 3 | 0 | 0,64698 | 0,12354 | 0,07133 |
| DHQ, 28 days | 4 | 0 | 0,4614 | 0,11881 | 0,0594 |
| DHQ, 42 days | 3 | 0 | 0,22383 | 0,02503 | 0,01445 |
| DHQ, 56 days | 3 | 0 | 0,17807 | 0,05767 | 0,03329 |

## ANOVA

### Overall ANOVA

| | DF | Sum of Squares | Mean Square | F Value | Prob>F |
|---|---|---|---|---|---|
| Model | 11 | 2,22686 | 0,20244 | 5,91645 | 3,2204E-5 |
| Error | 33 | 1,12916 | 0,03422 | | |
| Total | 44 | 3,35602 | | | |

Null Hypothesis: The means of all levels are equal.
Alternative Hypothesis: The means of one or more levels are different.
At the 0.05 level, the population means are significantly different.

### Fit Statistics

| | R-Square | Coeff Var | Root MSE | Data Mean |
|---|---|---|---|---|
| | 0,66354 | 0,44486 | 0,18498 | 0,41581 |

### Means Comparisons

#### Bonferroni Test

| | MeanDiff | SEM | t Value | Prob | Alpha | Sig | LCL | UCL |
|---|---|---|---|---|---|---|---|---|
| Control, 7 days  Control, 1 day | -0,53416 | 0,1308 | -4,08379 | 0,01749 | 0,05 | 1 | -1,01958 | -0,04873 |
| Control, 14 days  Control, 1 day | -0,54847 | 0,11328 | -4,84192 | 0,00194 | 0,05 | 1 | -0,96886 | -0,12808 |
| Control, 14 days  Control, 7 days | -0,01431 | 0,11328 | -0,12637 | 1 | 0,05 | 0 | -0,4347 | 0,40607 |
| Control, 28 days  Control, 1 day | -0,52956 | 0,1308 | -4,04869 | 0,01934 | 0,05 | 1 | -1,01499 | -0,04414 |
| Control, 28 days  Control, 7 days | 0,00459 | 0,1308 | 0,03511 | 1 | 0,05 | 0 | -0,48083 | 0,49001 |
| Control, 28 days  Control, 14 da | 0,01891 | 0,11328 | 0,1669 | 1 | 0,05 | 0 | -0,40148 | 0,43929 |
| Control, 42 days  Control, 1 day | -0,70585 | 0,14128 | -4,9961 | 0,00123 | 0,05 | 1 | -1,23016 | -0,18153 |
| Control, 42 days  Control, 7 days | -0,17169 | 0,14128 | -1,21525 | 1 | 0,05 | 0 | -0,69601 | 0,35263 |
| Control, 42 days  Control, 14 da | -0,15737 | 0,12523 | -1,25668 | 1 | 0,05 | 0 | -0,62213 | 0,30738 |
| Control, 42 days  Control, 28 da | -0,17628 | 0,14128 | -1,24775 | 1 | 0,05 | 0 | -0,7006 | 0,34804 |
| Control, 56 days  Control, 1 day | -0,72648 | 0,14128 | -5,14215 | 8,00653E-4 | 0,05 | 1 | -1,2508 | -0,20216 |
| Control, 56 days  Control, 7 days | -0,19232 | 0,14128 | -1,3613 | 1 | 0,05 | 0 | -0,71664 | 0,33199 |
| Control, 56 days  Control, 14 da | -0,17801 | 0,12523 | -1,42145 | 1 | 0,05 | 0 | -0,64277 | 0,28675 |
| Control, 56 days  Control, 28 da | -0,19692 | 0,14128 | -1,3938 | 1 | 0,05 | 0 | -0,72123 | 0,3274 |
| Control, 56 days  Control, 42 da | -0,02063 | 0,15103 | -0,13662 | 1 | 0,05 | 0 | -0,58115 | 0,53988 |
| DHQ, 1 day  Control, 1 day | -0,08295 | 0,14128 | -0,58714 | 1 | 0,05 | 0 | -0,60727 | 0,44137 |
| DHQ, 1 day  Control, 7 days | 0,45121 | 0,14128 | 3,19372 | 0,20352 | 0,05 | 0 | -0,07311 | 0,97552 |
| DHQ, 1 day  Control, 14 days | 0,46552 | 0,12523 | 3,71731 | 0,04916 | 0,05 | 1 | 7,63775E-4 | 0,93028 |
| DHQ, 1 day  Control, 28 days | 0,44661 | 0,14128 | 3,16122 | 0,2217 | 0,05 | 0 | -0,0777 | 0,97093 |
| DHQ, 1 day  Control, 42 days | 0,6229 | 0,15103 | 4,12421 | 0,01559 | 0,05 | 1 | 0,06238 | 1,18341 |
| DHQ, 1 day  Control, 56 days | 0,64353 | 0,15103 | 4,26083 | 0,01053 | 0,05 | 1 | 0,08301 | 1,20405 |
| DHQ, 7 days  Control, 1 day | -0,48407 | 0,14128 | -3,42631 | 0,1093 | 0,05 | 0 | -1,00838 | 0,04025 |
| DHQ, 7 days  Control, 7 days | 0,05009 | 0,14128 | 0,35455 | 1 | 0,05 | 0 | -0,47423 | 0,57441 |
| DHQ, 7 days  Control, 14 days | 0,0644 | 0,12523 | 0,51429 | 1 | 0,05 | 0 | -0,40035 | 0,52916 |
| DHQ, 7 days  Control, 28 days | 0,0455 | 0,14128 | 0,32205 | 1 | 0,05 | 0 | -0,47882 | 0,56982 |
| DHQ, 7 days  Control, 42 days | 0,22178 | 0,15103 | 1,46841 | 1 | 0,05 | 0 | -0,33874 | 0,7823 |
| DHQ, 7 days  Control, 56 days | 0,24241 | 0,15103 | 1,60503 | 1 | 0,05 | 0 | -0,3181 | 0,80293 |
| DHQ, 7 days  DHQ, 1 day | -0,40112 | 0,15103 | -2,6558 | 0,79792 | 0,05 | 0 | -0,96163 | 0,1594 |
| DHQ, 14 days  Control, 1 day | -0,23927 | 0,14128 | -1,69362 | 1 | 0,05 | 0 | -0,76359 | 0,28504 |
| DHQ, 14 days  Control, 7 days | 0,29488 | 0,14128 | 2,08724 | 1 | 0,05 | 0 | -0,22943 | 0,8192 |
| DHQ, 14 days  Control, 14 days | 0,3092 | 0,12523 | 2,46902 | 1 | 0,05 | 0 | -0,15556 | 0,77395 |
| DHQ, 14 days  Control, 28 days | 0,29029 | 0,14128 | 2,05473 | 1 | 0,05 | 0 | -0,23403 | 0,81461 |
| DHQ, 14 days  Control, 42 days | 0,46657 | 0,15103 | 3,08919 | 0,26764 | 0,05 | 0 | -0,09395 | 1,02709 |
| DHQ, 14 days  Control, 56 days | 0,48721 | 0,15103 | 3,22581 | 0,18698 | 0,05 | 0 | -0,07331 | 1,04772 |
| DHQ, 14 days  DHQ, 1 day | -0,15632 | 0,15103 | -1,03502 | 1 | 0,05 | 0 | -0,71684 | 0,40419 |
| DHQ, 14 days  DHQ, 7 days | 0,24479 | 0,15103 | 1,62078 | 1 | 0,05 | 0 | -0,31573 | 0,80531 |
| DHQ, 28 days  Control, 1 day | -0,42485 | 0,1308 | -3,24811 | 0,17625 | 0,05 | 0 | -0,91027 | 0,06057 |
| DHQ, 28 days  Control, 7 days | 0,10931 | 0,1308 | 0,83568 | 1 | 0,05 | 0 | -0,37612 | 0,59473 |
| DHQ, 28 days  Control, 14 days | 0,12362 | 0,11328 | 1,09133 | 1 | 0,05 | 0 | -0,29677 | 0,54401 |
| DHQ, 28 days  Control, 28 days | 0,10471 | 0,1308 | 0,80057 | 1 | 0,05 | 0 | -0,38071 | 0,59014 |
| DHQ, 28 days  Control, 42 days | 0,281 | 0,14128 | 1,98894 | 1 | 0,05 | 0 | -0,24332 | 0,80531 |
| DHQ, 28 days  Control, 56 days | 0,30163 | 0,14128 | 2,13499 | 1 | 0,05 | 0 | -0,22269 | 0,82595 |
| DHQ, 28 days  DHQ, 1 day | -0,3419 | 0,14128 | -2,42003 | 1 | 0,05 | 0 | -0,86622 | 0,18242 |
| DHQ, 28 days  DHQ, 7 days | 0,05922 | 0,14128 | 0,41914 | 1 | 0,05 | 0 | -0,4651 | 0,58353 |
| DHQ, 28 days  DHQ, 14 days | -0,18558 | 0,14128 | -1,31355 | 1 | 0,05 | 0 | -0,70989 | 0,33874 |
| DHQ, 42 days  Control, 1 day | -0,66242 | 0,14128 | -4,68871 | 0,00304 | 0,05 | 1 | -1,18673 | -0,1381 |
| DHQ, 42 days  Control, 7 days | -0,12826 | 0,14128 | -0,90785 | 1 | 0,05 | 0 | -0,65258 | 0,39606 |
| DHQ, 42 days  Control, 14 days | -0,11395 | 0,12523 | -0,90989 | 1 | 0,05 | 0 | -0,5787 | 0,35081 |
| DHQ, 42 days  Control, 28 days | -0,13285 | 0,14128 | -0,94036 | 1 | 0,05 | 0 | -0,65717 | 0,39146 |
| DHQ, 42 days  Control, 42 days | 0,04343 | 0,15103 | 0,28754 | 1 | 0,05 | 0 | -0,51709 | 0,60395 |
| DHQ, 42 days  Control, 56 days | 0,06406 | 0,15103 | 0,42416 | 1 | 0,05 | 0 | -0,49646 | 0,62458 |
| DHQ, 42 days  DHQ, 1 day | -0,57947 | 0,15103 | -3,83667 | 0,03522 | 0,05 | 1 | -1,13999 | -0,01895 |
| DHQ, 42 days  DHQ, 7 days | -0,17835 | 0,15103 | -1,18087 | 1 | 0,05 | 0 | -0,73887 | 0,38217 |
| DHQ, 42 days  DHQ, 14 days | -0,42314 | 0,15103 | -2,80165 | 0,55706 | 0,05 | 0 | -0,98366 | 0,13737 |
| DHQ, 42 days  DHQ, 28 days | -0,23757 | 0,14128 | -1,68154 | 1 | 0,05 | 0 | -0,76188 | 0,28675 |
| DHQ, 56 days  Control, 1 day | -0,70818 | 0,14128 | -5,01264 | 0,00117 | 0,05 | 1 | -1,2325 | -0,18386 |
| DHQ, 56 days  Control, 7 days | -0,17402 | 0,14128 | -1,23178 | 1 | 0,05 | 0 | -0,69834 | 0,35029 |
| DHQ, 56 days  Control, 14 days | -0,15971 | 0,12523 | -1,27533 | 1 | 0,05 | 0 | -0,62447 | 0,30505 |
| DHQ, 56 days  Control, 28 days | -0,17862 | 0,14128 | -1,26428 | 1 | 0,05 | 0 | -0,70293 | 0,3457 |
| DHQ, 56 days  Control, 42 days | -0,00234 | 0,15103 | -0,01547 | 1 | 0,05 | 0 | -0,56285 | 0,55818 |
| DHQ, 56 days  Control, 56 days | 0,0183 | 0,15103 | 0,12115 | 1 | 0,05 | 0 | -0,54222 | 0,57882 |
| DHQ, 56 days  DHQ, 1 day | -0,62523 | 0,15103 | -4,13968 | 0,01491 | 0,05 | 1 | -1,18575 | -0,06471 |
| DHQ, 56 days  DHQ, 7 days | -0,22412 | 0,15103 | -1,48387 | 1 | 0,05 | 0 | -0,78463 | 0,3364 |
| DHQ, 56 days  DHQ, 14 days | -0,46891 | 0,15103 | -3,10465 | 0,25707 | 0,05 | 0 | -1,02943 | 0,09161 |
| DHQ, 56 days  DHQ, 28 days | -0,28333 | 0,14128 | -2,00547 | 1 | 0,05 | 0 | -0,80765 | 0,24099 |
| DHQ, 56 days  DHQ, 42 days | -0,04576 | 0,15103 | -0,303 | 1 | 0,05 | 0 | -0,60628 | 0,51475 |

#### Scheffe Test

| | MeanDiff | SEM | F Value | Prob | Alpha | Sig | LCL | UCL |
|---|---|---|---|---|---|---|---|---|
| Control, 7 days  Control, 1 day | -0,53416 | 0,1308 | 1,51612 | 0,17251 | 0,05 | 0 | -1,1618 | 0,09349 |
| Control, 14 days  Control, 1 day | -0,54847 | 0,11328 | 2,13129 | 0,04603 | 0,05 | 1 | -1,09203 | -0,00492 |
| Control, 14 days  Control, 7 days | -0,01431 | 0,11328 | 0,00145 | 1 | 0,05 | 0 | -0,55787 | 0,52924 |
| Control, 28 days  Control, 1 day | -0,52956 | 0,1308 | 1,49017 | 0,18207 | 0,05 | 0 | -1,15721 | 0,09808 |
| Control, 28 days  Control, 7 days | 0,00459 | 0,1308 | 1,12046E-4 | 1 | 0,05 | 0 | -0,62305 | 0,63223 |
| Control, 28 days  Control, 14 da | 0,01891 | 0,11328 | 0,00253 | 1 | 0,05 | 0 | -0,52465 | 0,56246 |
| Control, 42 days  Control, 1 day | -0,70585 | 0,14128 | 2,26919 | 0,0341 | 0,05 | 1 | -1,38378 | -0,02791 |
| Control, 42 days  Control, 7 days | -0,17169 | 0,14128 | 0,13426 | 0,99942 | 0,05 | 0 | -0,84962 | 0,50624 |
| Control, 42 days  Control, 14 da | -0,15737 | 0,12523 | 0,14357 | 0,99921 | 0,05 | 0 | -0,7583 | 0,44355 |
| Control, 42 days  Control, 28 da | -0,17628 | 0,14128 | 0,14153 | 0,99926 | 0,05 | 0 | -0,85421 | 0,50165 |
| Control, 56 days  Control, 1 day | -0,72648 | 0,14128 | 2,4038 | 0,02546 | 0,05 | 1 | -1,40441 | -0,04855 |
| Control, 56 days  Control, 7 days | -0,19232 | 0,14128 | 0,16847 | 0,99835 | 0,05 | 0 | -0,87025 | 0,48561 |
| Control, 56 days  Control, 14 da | -0,17801 | 0,12523 | 0,18368 | 0,99756 | 0,05 | 0 | -0,77893 | 0,42291 |
| Control, 56 days  Control, 28 da | -0,19692 | 0,14128 | 0,17661 | 0,99796 | 0,05 | 0 | -0,87485 | 0,48102 |
| Control, 56 days  Control, 42 da | -0,02063 | 0,15103 | 0,0017 | 1 | 0,05 | 0 | -0,74537 | 0,7041 |
| DHQ, 1 day  Control, 1 day | -0,08295 | 0,14128 | 0,03134 | 1 | 0,05 | 0 | -0,76088 | 0,59498 |
| DHQ, 1 day  Control, 7 days | 0,45121 | 0,14128 | 0,92726 | 0,52702 | 0,05 | 0 | -0,22672 | 1,12914 |
| DHQ, 1 day  Control, 14 days | 0,46552 | 0,12523 | 1,25622 | 0,29171 | 0,05 | 0 | -0,1354 | 1,06644 |
| DHQ, 1 day  Control, 28 days | 0,44661 | 0,14128 | 0,90848 | 0,54308 | 0,05 | 0 | -0,23132 | 1,12455 |
| DHQ, 1 day  Control, 42 days | 0,6229 | 0,15103 | 1,54628 | 0,16199 | 0,05 | 0 | -0,10184 | 1,34763 |
| DHQ, 1 day  Control, 56 days | 0,64353 | 0,15103 | 1,65043 | 0,13005 | 0,05 | 0 | -0,08121 | 1,36827 |
| DHQ, 7 days  Control, 1 day | -0,48407 | 0,14128 | 1,06723 | 0,41534 | 0,05 | 0 | -1,162 | 0,19387 |
| DHQ, 7 days  Control, 7 days | 0,05009 | 0,14128 | 0,01143 | 1 | 0,05 | 0 | -0,62784 | 0,72802 |
| DHQ, 7 days  Control, 14 days | 0,0644 | 0,12523 | 0,02404 | 1 | 0,05 | 0 | -0,53652 | 0,66533 |
| DHQ, 7 days  Control, 28 days | 0,0455 | 0,14128 | 0,00943 | 1 | 0,05 | 0 | -0,63243 | 0,72343 |
| DHQ, 7 days  Control, 42 days | 0,22178 | 0,15103 | 0,19602 | 0,99675 | 0,05 | 0 | -0,50296 | 0,94652 |
| DHQ, 7 days  Control, 56 days | 0,24241 | 0,15103 | 0,23419 | 0,99304 | 0,05 | 0 | -0,48233 | 0,96715 |
| DHQ, 7 days  DHQ, 1 day | -0,40112 | 0,15103 | 0,64121 | 0,78062 | 0,05 | 0 | -1,12586 | 0,32362 |
| DHQ, 14 days  Control, 1 day | -0,23927 | 0,14128 | 0,26076 | 0,98916 | 0,05 | 0 | -0,9172 | 0,43866 |
| DHQ, 14 days  Control, 7 days | 0,29488 | 0,14128 | 0,39605 | 0,94783 | 0,05 | 0 | -0,38305 | 0,97281 |
| DHQ, 14 days  Control, 14 days | 0,3092 | 0,12523 | 0,55419 | 0,8509 | 0,05 | 0 | -0,29172 | 0,91012 |
| DHQ, 14 days  Control, 28 days | 0,29029 | 0,14128 | 0,38381 | 0,9532 | 0,05 | 0 | -0,38764 | 0,96822 |
| DHQ, 14 days  Control, 42 days | 0,46657 | 0,15103 | 0,86755 | 0,57877 | 0,05 | 0 | -0,25817 | 1,19131 |
| DHQ, 14 days  Control, 56 days | 0,48721 | 0,15103 | 0,94598 | 0,51122 | 0,05 | 0 | -0,23753 | 1,21195 |
| DHQ, 14 days  DHQ, 1 day | -0,15632 | 0,15103 | 0,09739 | 0,99988 | 0,05 | 0 | -0,88106 | 0,56842 |
| DHQ, 14 days  DHQ, 7 days | 0,24479 | 0,15103 | 0,23881 | 0,99245 | 0,05 | 0 | -0,47995 | 0,96953 |
| DHQ, 28 days  Control, 1 day | -0,42485 | 0,1308 | 0,95911 | 0,50029 | 0,05 | 0 | -1,05249 | 0,20279 |
| DHQ, 28 days  Control, 7 days | 0,10931 | 0,1308 | 0,06349 | 0,99999 | 0,05 | 0 | -0,51834 | 0,73695 |
| DHQ, 28 days  Control, 14 days | 0,12362 | 0,11328 | 0,10827 | 0,99979 | 0,05 | 0 | -0,41993 | 0,66717 |
| DHQ, 28 days  Control, 28 days | 0,10471 | 0,1308 | 0,05827 | 0,99999 | 0,05 | 0 | -0,52293 | 0,73236 |
| DHQ, 28 days  Control, 42 days | 0,281 | 0,14128 | 0,35962 | 0,96283 | 0,05 | 0 | -0,39694 | 0,95893 |
| DHQ, 28 days  Control, 56 days | 0,30163 | 0,14128 | 0,41438 | 0,93918 | 0,05 | 0 | -0,3763 | 0,97956 |
| DHQ, 28 days  DHQ, 1 day | -0,3419 | 0,14128 | 0,53241 | 0,86695 | 0,05 | 0 | -1,01983 | 0,33603 |
| DHQ, 28 days  DHQ, 7 days | 0,05922 | 0,14128 | 0,01597 | 1 | 0,05 | 0 | -0,61872 | 0,73715 |
| DHQ, 28 days  DHQ, 14 days | -0,18558 | 0,14128 | 0,15685 | 0,99881 | 0,05 | 0 | -0,86351 | 0,49235 |
| DHQ, 42 days  Control, 1 day | -0,66242 | 0,14128 | 1,99855 | 0,06144 | 0,05 | 0 | -1,34035 | 0,01551 |
| DHQ, 42 days  Control, 7 days | -0,12826 | 0,14128 | 0,07493 | 0,99997 | 0,05 | 0 | -0,80619 | 0,54967 |
| DHQ, 42 days  Control, 14 days | -0,11395 | 0,12523 | 0,07526 | 0,99997 | 0,05 | 0 | -0,71487 | 0,48698 |
| DHQ, 42 days  Control, 28 days | -0,13285 | 0,14128 | 0,08039 | 0,99995 | 0,05 | 0 | -0,81078 | 0,54508 |
| DHQ, 42 days  Control, 42 days | 0,04343 | 0,15103 | 0,00752 | 1 | 0,05 | 0 | -0,68131 | 0,76817 |
| DHQ, 42 days  Control, 56 days | 0,06406 | 0,15103 | 0,01636 | 1 | 0,05 | 0 | -0,66068 | 0,7888 |
| DHQ, 42 days  DHQ, 1 day | -0,57947 | 0,15103 | 1,33819 | 0,24815 | 0,05 | 0 | -1,30421 | 0,14527 |
| DHQ, 42 days  DHQ, 7 days | -0,17835 | 0,15103 | 0,12677 | 0,99956 | 0,05 | 0 | -0,90309 | 0,54639 |
| DHQ, 42 days  DHQ, 14 days | -0,42314 | 0,15103 | 0,71357 | 0,71716 | 0,05 | 0 | -1,14788 | 0,3016 |
| DHQ, 42 days  DHQ, 28 days | -0,23757 | 0,14128 | 0,25705 | 0,98977 | 0,05 | 0 | -0,9155 | 0,44036 |
| DHQ, 56 days  Control, 1 day | -0,70818 | 0,14128 | 2,28423 | 0,033 | 0,05 | 1 | -1,38611 | -0,03025 |
| DHQ, 56 days  Control, 7 days | -0,17402 | 0,14128 | 0,13793 | 0,99934 | 0,05 | 0 | -0,85196 | 0,50391 |
| DHQ, 56 days  Control, 14 days | -0,15971 | 0,12523 | 0,14786 | 0,99909 | 0,05 | 0 | -0,76063 | 0,44121 |
| DHQ, 56 days  Control, 28 days | -0,17862 | 0,14128 | 0,14531 | 0,99916 | 0,05 | 0 | -0,85655 | 0,49931 |
| DHQ, 56 days  Control, 42 days | -0,00234 | 0,15103 | 2,17439E-5 | 1 | 0,05 | 0 | -0,72707 | 0,7224 |
| DHQ, 56 days  Control, 56 days | 0,0183 | 0,15103 | 0,00133 | 1 | 0,05 | 0 | -0,70644 | 0,74304 |
| DHQ, 56 days  DHQ, 1 day | -0,62523 | 0,15103 | 1,5579 | 0,15809 | 0,05 | 0 | -1,34997 | 0,09951 |
| DHQ, 56 days  DHQ, 7 days | -0,22412 | 0,15103 | 0,20017 | 0,99644 | 0,05 | 0 | -0,94885 | 0,50062 |
| DHQ, 56 days  DHQ, 14 days | -0,46891 | 0,15103 | 0,87626 | 0,57111 | 0,05 | 0 | -1,19365 | 0,25583 |
| DHQ, 56 days  DHQ, 28 days | -0,28333 | 0,14128 | 0,36563 | 0,96056 | 0,05 | 0 | -0,96126 | 0,3946 |
| DHQ, 56 days  DHQ, 42 days | -0,04576 | 0,15103 | 0,00835 | 1 | 0,05 | 0 | -0,7705 | 0,67898 |

#### Tukey Test

| | MeanDiff | SEM | q Value | Prob | Alpha | Sig | LCL | UCL |
|---|---|---|---|---|---|---|---|---|
| Control, 7 days  Control, 1 day | -0,53416 | 0,1308 | 5,77535 | 0,01193 | 0,05 | 1 | -0,9934 | -0,07491 |
| Control, 14 days  Control, 1 day | -0,54847 | 0,11328 | 6,84751 | 0,00151 | 0,05 | 1 | -0,94619 | -0,15075 |
| Control, 14 days  Control, 7 days | -0,01431 | 0,11328 | 0,17871 | 1 | 0,05 | 0 | -0,41203 | 0,38341 |
| Control, 28 days  Control, 1 day | -0,52956 | 0,1308 | 5,72571 | 0,01307 | 0,05 | 1 | -0,98881 | -0,07032 |
| Control, 28 days  Control, 7 days | 0,00459 | 0,1308 | 0,04965 | 1 | 0,05 | 0 | -0,45465 | 0,46384 |
| Control, 28 days  Control, 14 da | 0,01891 | 0,11328 | 0,23604 | 1 | 0,05 | 0 | -0,37881 | 0,41663 |
| Control, 42 days  Control, 1 day | -0,70585 | 0,14128 | 7,06556 | 9,77971E-4 | 0,05 | 1 | -1,20189 | -0,2098 |
| Control, 42 days  Control, 7 days | -0,17169 | 0,14128 | 1,71862 | 0,98379 | 0,05 | 0 | -0,66773 | 0,32435 |
| Control, 42 days  Control, 14 da | -0,15737 | 0,12523 | 1,77721 | 0,97917 | 0,05 | 0 | -0,59707 | 0,28232 |
| Control, 42 days  Control, 28 da | -0,17628 | 0,14128 | 1,76458 | 0,98024 | 0,05 | 0 | -0,67232 | 0,31976 |
| Control, 56 days  Control, 1 day | -0,72648 | 0,14128 | 7,27211 | 6,46203E-4 | 0,05 | 1 | -1,22252 | -0,23044 |
| Control, 56 days  Control, 7 days | -0,19232 | 0,14128 | 1,92517 | 0,96312 | 0,05 | 0 | -0,68837 | 0,30372 |
| Control, 56 days  Control, 14 da | -0,17801 | 0,12523 | 2,01023 | 0,9506 | 0,05 | 0 | -0,6177 | 0,26169 |
| Control, 56 days  Control, 28 da | -0,19692 | 0,14128 | 1,97113 | 0,95668 | 0,05 | 0 | -0,69296 | 0,29913 |
| Control, 56 days  Control, 42 da | -0,02063 | 0,15103 | 0,19321 | 1 | 0,05 | 0 | -0,55093 | 0,50966 |
| DHQ, 1 day  Control, 1 day | -0,08295 | 0,14128 | 0,83033 | 0,99998 | 0,05 | 0 | -0,57899 | 0,41309 |
| DHQ, 1 day  Control, 7 days | 0,45121 | 0,14128 | 4,5166 | 0,10261 | 0,05 | 0 | -0,04484 | 0,94725 |
| DHQ, 1 day  Control, 14 days | 0,46552 | 0,12523 | 5,25707 | 0,03037 | 0,05 | 1 | 0,02583 | 0,90522 |
| DHQ, 1 day  Control, 28 days | 0,44661 | 0,14128 | 4,47064 | 0,11003 | 0,05 | 0 | -0,04943 | 0,94266 |
| DHQ, 1 day  Control, 42 days | 0,6229 | 0,15103 | 5,83252 | 0,01073 | 0,05 | 1 | 0,0926 | 1,15319 |
| DHQ, 1 day  Control, 56 days | 0,64353 | 0,15103 | 6,02572 | 0,00746 | 0,05 | 1 | 0,11324 | 1,17382 |
| DHQ, 7 days  Control, 1 day | -0,48407 | 0,14128 | 4,84553 | 0,06096 | 0,05 | 0 | -0,98011 | 0,01198 |
| DHQ, 7 days  Control, 7 days | 0,05009 | 0,14128 | 0,50141 | 1 | 0,05 | 0 | -0,44595 | 0,54613 |
| DHQ, 7 days  Control, 14 days | 0,0644 | 0,12523 | 0,72731 | 0,99999 | 0,05 | 0 | -0,37529 | 0,5041 |
| DHQ, 7 days  Control, 28 days | 0,0455 | 0,14128 | 0,45544 | 1 | 0,05 | 0 | -0,45054 | 0,54154 |
| DHQ, 7 days  Control, 42 days | 0,22178 | 0,15103 | 2,07664 | 0,93896 | 0,05 | 0 | -0,30851 | 0,75207 |
| DHQ, 7 days  Control, 56 days | 0,24241 | 0,15103 | 2,26985 | 0,89509 | 0,05 | 0 | -0,28788 | 0,77271 |
| DHQ, 7 days  DHQ, 1 day | -0,40112 | 0,15103 | 3,75587 | 0,29127 | 0,05 | 0 | -0,93141 | 0,12918 |
| DHQ, 14 days  Control, 1 day | -0,23927 | 0,14128 | 2,39514 | 0,85851 | 0,05 | 0 | -0,73532 | 0,25677 |
| DHQ, 14 days  Control, 7 days | 0,29488 | 0,14128 | 2,9518 | 0,63446 | 0,05 | 0 | -0,20116 | 0,79093 |
| DHQ, 14 days  Control, 14 days | 0,3092 | 0,12523 | 3,49172 | 0,39213 | 0,05 | 0 | -0,1305 | 0,74889 |
| DHQ, 14 days  Control, 28 days | 0,29029 | 0,14128 | 2,90583 | 0,65543 | 0,05 | 0 | -0,20575 | 0,78633 |
| DHQ, 14 days  Control, 42 days | 0,46657 | 0,15103 | 4,36877 | 0,12808 | 0,05 | 0 | -0,06372 | 0,99686 |
| DHQ, 14 days  Control, 56 days | 0,48721 | 0,15103 | 4,56198 | 0,09571 | 0,05 | 0 | -0,04309 | 1,0175 |
| DHQ, 14 days  DHQ, 1 day | -0,15632 | 0,15103 | 1,46374 | 0,9955 | 0,05 | 0 | -0,68662 | 0,37397 |
| DHQ, 14 days  DHQ, 7 days | 0,24479 | 0,15103 | 2,29213 | 0,88905 | 0,05 | 0 | -0,2855 | 0,77509 |
| DHQ, 28 days  Control, 1 day | -0,42485 | 0,1308 | 4,59352 | 0,09114 | 0,05 | 0 | -0,8841 | 0,0344 |
| DHQ, 28 days  Control, 7 days | 0,10931 | 0,1308 | 1,18183 | 0,99932 | 0,05 | 0 | -0,34994 | 0,56855 |
| DHQ, 28 days  Control, 14 days | 0,12362 | 0,11328 | 1,54337 | 0,99304 | 0,05 | 0 | -0,2741 | 0,52134 |
| DHQ, 28 days  Control, 28 days | 0,10471 | 0,1308 | 1,13218 | 0,99954 | 0,05 | 0 | -0,35453 | 0,56396 |
| DHQ, 28 days  Control, 42 days | 0,281 | 0,14128 | 2,81278 | 0,6971 | 0,05 | 0 | -0,21505 | 0,77704 |
| DHQ, 28 days  Control, 56 days | 0,30163 | 0,14128 | 3,01933 | 0,60337 | 0,05 | 0 | -0,19441 | 0,79767 |
| DHQ, 28 days  DHQ, 1 day | -0,3419 | 0,14128 | 3,42244 | 0,42123 | 0,05 | 0 | -0,83794 | 0,15414 |
| DHQ, 28 days  DHQ, 7 days | 0,05922 | 0,14128 | 0,59275 | 1 | 0,05 | 0 | -0,43683 | 0,55526 |
| DHQ, 28 days  DHQ, 14 days | -0,18558 | 0,14128 | 1,85763 | 0,97129 | 0,05 | 0 | -0,68162 | 0,31047 |
| DHQ, 42 days  Control, 1 day | -0,66242 | 0,14128 | 6,63084 | 0,00232 | 0,05 | 1 | -1,15846 | -0,16637 |
| DHQ, 42 days  Control, 7 days | -0,12826 | 0,14128 | 1,2839 | 0,99855 | 0,05 | 0 | -0,6243 | 0,36778 |
| DHQ, 42 days  Control, 14 days | -0,11395 | 0,12523 | 1,28679 | 0,99853 | 0,05 | 0 | -0,55364 | 0,32575 |
| DHQ, 42 days  Control, 28 days | -0,13285 | 0,14128 | 1,32986 | 0,99803 | 0,05 | 0 | -0,6289 | 0,36319 |
| DHQ, 42 days  Control, 42 days | 0,04343 | 0,15103 | 0,40664 | 1 | 0,05 | 0 | -0,48686 | 0,57372 |
| DHQ, 42 days  Control, 56 days | 0,06406 | 0,15103 | 0,59985 | 1 | 0,05 | 0 | -0,46623 | 0,59435 |
| DHQ, 42 days  DHQ, 1 day | -0,57947 | 0,15103 | 5,42587 | 0,02254 | 0,05 | 1 | -1,10976 | -0,04917 |
| DHQ, 42 days  DHQ, 7 days | -0,17835 | 0,15103 | 1,67 | 0,98698 | 0,05 | 0 | -0,70864 | 0,35194 |
| DHQ, 42 days  DHQ, 14 days | -0,42314 | 0,15103 | 3,96213 | 0,22518 | 0,05 | 0 | -0,95344 | 0,10715 |
| DHQ, 42 days  DHQ, 28 days | -0,23757 | 0,14128 | 2,37806 | 0,86386 | 0,05 | 0 | -0,73361 | 0,25848 |
| DHQ, 56 days  Control, 1 day | -0,70818 | 0,14128 | 7,08894 | 9,33271E-4 | 0,05 | 1 | -1,20422 | -0,21214 |
| DHQ, 56 days  Control, 7 days | -0,17402 | 0,14128 | 1,742 | 0,98205 | 0,05 | 0 | -0,67007 | 0,32202 |
| DHQ, 56 days  Control, 14 days | -0,15971 | 0,12523 | 1,80359 | 0,97679 | 0,05 | 0 | -0,59941 | 0,27998 |
| DHQ, 56 days  Control, 28 days | -0,17862 | 0,14128 | 1,78796 | 0,97822 | 0,05 | 0 | -0,67466 | 0,31743 |
| DHQ, 56 days  Control, 42 days | -0,00234 | 0,15103 | 0,02187 | 1 | 0,05 | 0 | -0,53263 | 0,52796 |
| DHQ, 56 days  Control, 56 days | 0,0183 | 0,15103 | 0,17134 | 1 | 0,05 | 0 | -0,51199 | 0,54859 |
| DHQ, 56 days  DHQ, 1 day | -0,62523 | 0,15103 | 5,85439 | 0,0103 | 0,05 | 1 | -1,15552 | -0,09494 |
| DHQ, 56 days  DHQ, 7 days | -0,22412 | 0,15103 | 2,09852 | 0,93475 | 0,05 | 0 | -0,75441 | 0,30618 |
| DHQ, 56 days  DHQ, 14 days | -0,46891 | 0,15103 | 4,39064 | 0,12401 | 0,05 | 0 | -0,9992 | 0,06138 |
| DHQ, 56 days  DHQ, 28 days | -0,28333 | 0,14128 | 2,83616 | 0,68675 | 0,05 | 0 | -0,77937 | 0,21271 |
| DHQ, 56 days  DHQ, 42 days | -0,04576 | 0,15103 | 0,42851 | 1 | 0,05 | 0 | -0,57606 | 0,48453 |

### Grouping Letters

#### Bonferroni Test

| | Mean | Groups | | |
|---|---|---|---|---|
| Control, 1 day | 0,88625 | A | | |
| DHQ, 1 day | 0,8033 | A | B | |
| DHQ, 14 days | 0,64698 | A | B | C |
| DHQ, 28 days | 0,4614 | A | B | C |
| DHQ, 7 days | 0,40218 | A | B | C |
| Control, 28 days | 0,35669 | | B | C |
| Control, 7 days | 0,35209 | | B | C |
| Control, 14 days | 0,33778 | | | C |
| DHQ, 42 days | 0,22383 | | | C |
| Control, 42 days | 0,1804 | | | C |
| DHQ, 56 days | 0,17807 | | | C |
| Control, 56 days | 0,15977 | | | C |

Means that do not share a letter are significantly different.

#### Scheffe Test

| | Mean | Groups | |
|---|---|---|---|
| Control, 1 day | 0,88625 | A | |
| DHQ, 1 day | 0,8033 | A | B |
| DHQ, 14 days | 0,64698 | A | B |
| DHQ, 28 days | 0,4614 | A | B |
| DHQ, 7 days | 0,40218 | A | B |
| Control, 28 days | 0,35669 | A | B |
| Control, 7 days | 0,35209 | A | B |
| Control, 14 days | 0,33778 | | B |
| DHQ, 42 days | 0,22383 | A | B |
| Control, 42 days | 0,1804 | | B |
| DHQ, 56 days | 0,17807 | | B |
| Control, 56 days | 0,15977 | | B |

Means that do not share a letter are significantly different.

#### Tukey Test

| | Mean | Groups | | |
|---|---|---|---|---|
| Control, 1 day | 0,88625 | A | | |
| DHQ, 1 day | 0,8033 | A | B | |
| DHQ, 14 days | 0,64698 | A | B | C |
| DHQ, 28 days | 0,4614 | A | B | C |
| DHQ, 7 days | 0,40218 | A | B | C |
| Control, 28 days | 0,35669 | | B | C |
| Control, 7 days | 0,35209 | | B | C |
| Control, 14 days | 0,33778 | | | C |
| DHQ, 42 days | 0,22383 | | | C |
| Control, 42 days | 0,1804 | | | C |
| DHQ, 56 days | 0,17807 | | | C |
| Control, 56 days | 0,15977 | | | C |

Means that do not share a letter are significantly different.

Sig equals 1 indicates that the difference of the means is significant at the 0,05 level.
Sig equals 0 indicates that the difference of the means is not significant at the 0,05 level.

## Powers

| | Alpha | Sample Size | Power |
|---|---|---|---|
| Actual Power | 0,05 | 45 | 0,99972 |

## ANOVAOneWay (27.10.2024 22:12:03)

### Notes

| | |
|---|---|
| Description | Perform One-Way ANOVA |
| User Name | yulia |
| Operation Time | 27.10.2024 22:12:03 |
| Report Status | New Analysis Report |
| Data Filter | No |

### Input Data

| | Data | Range |
|---|---|---|
| DHQ, 1 day | [Book1]"MTT, DHQ % of control"!A" DHQ, 1 day" | [1*:3*] |
| DHQ, 7 days | [Book1]"MTT, DHQ % of control"!B" DHQ, 7 days" | [1*:3*] |
| DHQ, 14 days | [Book1]"MTT, DHQ % of control"!C" DHQ, 14 days" | [1*:3*] |
| DHQ, 28 days | [Book1]"MTT, DHQ % of control"!D" DHQ, 28 days" | [1*:4*] |
| DHQ, 42 days | [Book1]"MTT, DHQ % of control"!E" DHQ, 42 days" | [1*:3*] |
| DHQ, 56 days | [Book1]"MTT, DHQ % of control"!F" DHQ, 56 days" | [1*:3*] |

### Descriptive Statistics

| | N Analysis | N Missing | Mean | Standard Deviation | SE of Mean |
|---|---|---|---|---|---|
| DHQ, 1 day | 3 | 0 | 0,75349 | 0,00797 | 0,0046 |
| DHQ, 7 days | 3 | 0 | 1,14413 | 0,27832 | 0,16069 |
| DHQ, 14 days | 3 | 0 | 1,91675 | 0,37685 | 0,21758 |
| DHQ, 28 days | 4 | 0 | 1,36762 | 0,56177 | 0,28088 |
| DHQ, 42 days | 3 | 0 | 1,23887 | 0,01924 | 0,01111 |
| DHQ, 56 days | 3 | 0 | 1,10755 | 0,02286 | 0,0132 |

### ANOVA

#### Overall ANOVA

| | DF | Sum of Squares | Mean Square | F Value | Prob>F |
|---|---|---|---|---|---|
| Model | 5 | 2,22128 | 0,44426 | 4,16204 | 0,01771 |
| Error | 13 | 1,38762 | 0,10674 | | |
| Total | 18 | 3,60891 | | | |

Null Hypothesis: The means of all levels are equal.
Alternative Hypothesis: The means of one or more levels are different.
At the 0.05 level, the population means are significantly different.

#### Fit Statistics

| R-Square | Coeff Var | Root MSE | Data Mean |
|---|---|---|---|
| 0,6155 | 0,25916 | 0,32671 | 1,26068 |

#### Means Comparisons

##### Bonferroni Test

| | | MeanDiff | SEM | t Value | Prob | Alpha | Sig | LCL | UCL |
|---|---|---|---|---|---|---|---|---|---|
| DHQ, 7 days | DHQ, 1 day | 0,39064 | 0,26676 | 1,46439 | 1 | 0,05 | 0 | -0,56538 | 1,34666 |
| DHQ, 14 days | DHQ, 1 day | 1,16326 | 0,26676 | 4,36073 | 0,01157 | 0,05 | 1 | 0,20724 | 2,11928 |
| DHQ, 14 days | DHQ, 7 days | 0,77262 | 0,26676 | 2,89634 | 0,18743 | 0,05 | 0 | -0,1834 | 1,72864 |
| DHQ, 28 days | DHQ, 1 day | 0,61413 | 0,24953 | 2,46115 | 0,4291 | 0,05 | 0 | -0,28014 | 1,50841 |
| DHQ, 28 days | DHQ, 7 days | 0,22349 | 0,24953 | 0,89566 | 1 | 0,05 | 0 | -0,67078 | 1,11777 |
| DHQ, 28 days | DHQ, 14 days | -0,54913 | 0,24953 | -2,20066 | 0,69662 | 0,05 | 0 | -1,4434 | 0,34514 |
| DHQ, 42 days | DHQ, 1 day | 0,48538 | 0,26676 | 1,81956 | 1 | 0,05 | 0 | -0,47064 | 1,4414 |
| DHQ, 42 days | DHQ, 7 days | 0,09474 | 0,26676 | 0,35516 | 1 | 0,05 | 0 | -0,86128 | 1,05076 |
| DHQ, 42 days | DHQ, 14 days | -0,67788 | 0,26676 | -2,54117 | 0,369 | 0,05 | 0 | -1,6339 | 0,27814 |
| DHQ, 42 days | DHQ, 28 days | -0,12875 | 0,24953 | -0,51597 | 1 | 0,05 | 0 | -1,02302 | 0,76553 |
| DHQ, 56 days | DHQ, 1 day | 0,35406 | 0,26676 | 1,32727 | 1 | 0,05 | 0 | -0,60196 | 1,31008 |
| DHQ, 56 days | DHQ, 7 days | -0,03658 | 0,26676 | -0,13712 | 1 | 0,05 | 0 | -0,9926 | 0,91944 |
| DHQ, 56 days | DHQ, 14 days | -0,8092 | 0,26676 | -3,03346 | 0,14401 | 0,05 | 0 | -1,76522 | 0,14682 |
| DHQ, 56 days | DHQ, 28 days | -0,26007 | 0,24953 | -1,04224 | 1 | 0,05 | 0 | -1,15434 | 0,6342 |
| DHQ, 56 days | DHQ, 42 day | -0,13132 | 0,26676 | -0,49228 | 1 | 0,05 | 0 | -1,08734 | 0,8247 |

##### Scheffe Test

| | | MeanDiff | SEM | F Value | Prob | Alpha | Sig | LCL | UCL |
|---|---|---|---|---|---|---|---|---|---|
| DHQ, 7 days | DHQ, 1 day | 0,39064 | 0,26676 | 0,42889 | 0,82058 | 0,05 | 0 | -0,64688 | 1,42816 |
| DHQ, 14 days | DHQ, 1 day | 1,16326 | 0,26676 | 3,80319 | 0,0242 | 0,05 | 1 | 0,12574 | 2,20078 |
| DHQ, 14 days | DHQ, 7 days | 0,77262 | 0,26676 | 1,67775 | 0,20909 | 0,05 | 0 | -0,2649 | 1,81015 |
| DHQ, 28 days | DHQ, 1 day | 0,61413 | 0,24953 | 1,21146 | 0,35741 | 0,05 | 0 | -0,35638 | 1,58464 |
| DHQ, 28 days | DHQ, 7 days | 0,22349 | 0,24953 | 0,16044 | 0,97276 | 0,05 | 0 | -0,74702 | 1,19401 |
| DHQ, 28 days | DHQ, 14 days | -0,54913 | 0,24953 | 0,96858 | 0,47192 | 0,05 | 0 | -1,51964 | 0,42138 |
| DHQ, 42 days | DHQ, 1 day | 0,48538 | 0,26676 | 0,66216 | 0,65851 | 0,05 | 0 | -0,55214 | 1,5229 |
| DHQ, 42 days | DHQ, 7 days | 0,09474 | 0,26676 | 0,02523 | 0,99963 | 0,05 | 0 | -0,94278 | 1,13227 |
| DHQ, 42 days | DHQ, 14 day | -0,67788 | 0,26676 | 1,29151 | 0,32586 | 0,05 | 0 | -1,7154 | 0,35964 |
| DHQ, 42 days | DHQ, 28 day | -0,12875 | 0,24953 | 0,05324 | 0,99778 | 0,05 | 0 | -1,09926 | 0,84176 |
| DHQ, 56 days | DHQ, 1 day | 0,35406 | 0,26676 | 0,35233 | 0,87174 | 0,05 | 0 | -0,68346 | 1,39158 |
| DHQ, 56 days | DHQ, 7 days | -0,03658 | 0,26676 | 0,00376 | 1 | 0,05 | 0 | -1,0741 | 1,00094 |
| DHQ, 56 days | DHQ, 14 day | -0,8092 | 0,26676 | 1,84037 | 0,174 | 0,05 | 0 | -1,84672 | 0,22832 |
| DHQ, 56 days | DHQ, 28 day | -0,26007 | 0,24953 | 0,21725 | 0,94884 | 0,05 | 0 | -1,23058 | 0,71044 |
| DHQ, 56 days | DHQ, 42 day | -0,13132 | 0,26676 | 0,04847 | 0,99822 | 0,05 | 0 | -1,16884 | 0,9062 |

##### Tukey Test

| | | MeanDiff | SEM | q Value | Prob | Alpha | Sig | LCL | UCL |
|---|---|---|---|---|---|---|---|---|---|
| DHQ, 7 days | DHQ, 1 day | 0,39064 | 0,26676 | 2,07096 | 0,69059 | 0,05 | 0 | -0,49396 | 1,27524 |
| DHQ, 14 days | DHQ, 1 day | 1,16326 | 0,26676 | 6,167 | 0,00794 | 0,05 | 1 | 0,27866 | 2,04786 |
| DHQ, 14 days | DHQ, 7 days | 0,77262 | 0,26676 | 4,09604 | 0,10234 | 0,05 | 0 | -0,11198 | 1,65722 |
| DHQ, 28 days | DHQ, 1 day | 0,61413 | 0,24953 | 3,4806 | 0,20525 | 0,05 | 0 | -0,21334 | 1,4416 |
| DHQ, 28 days | DHQ, 7 days | 0,22349 | 0,24953 | 1,26665 | 0,94087 | 0,05 | 0 | -0,60398 | 1,05096 |
| DHQ, 28 days | DHQ, 14 day | -0,54913 | 0,24953 | 3,1122 | 0,30014 | 0,05 | 0 | -1,3766 | 0,27834 |
| DHQ, 42 days | DHQ, 1 day | 0,48538 | 0,26676 | 2,57324 | 0,48698 | 0,05 | 0 | -0,39922 | 1,36998 |
| DHQ, 42 days | DHQ, 7 days | 0,09474 | 0,26676 | 0,50228 | 0,99907 | 0,05 | 0 | -0,78986 | 0,97934 |
| DHQ, 42 days | DHQ, 14 day | -0,67788 | 0,26676 | 3,59376 | 0,1815 | 0,05 | 0 | -1,56248 | 0,20672 |
| DHQ, 42 days | DHQ, 28 day | -0,12875 | 0,24953 | 0,72969 | 0,99455 | 0,05 | 0 | -0,95622 | 0,69872 |
| DHQ, 56 days | DHQ, 1 day | 0,35406 | 0,26676 | 1,87705 | 0,76598 | 0,05 | 0 | -0,53054 | 1,23866 |
| DHQ, 56 days | DHQ, 7 days | -0,03658 | 0,26676 | 0,19392 | 0,99999 | 0,05 | 0 | -0,92118 | 0,84802 |
| DHQ, 56 days | DHQ, 14 day | -0,8092 | 0,26676 | 4,28996 | 0,08128 | 0,05 | 0 | -1,6938 | 0,0754 |
| DHQ, 56 days | DHQ, 28 day | -0,26007 | 0,24953 | 1,47395 | 0,8951 | 0,05 | 0 | -1,08754 | 0,5674 |
| DHQ, 56 days | DHQ, 42 day | -0,13132 | 0,26676 | 0,6962 | 0,99561 | 0,05 | 0 | -1,01592 | 0,75328 |

##### Grouping Letters Table

###### Bonferroni Test

| | Mean | Groups | |
|---|---|---|---|
| DHQ, 14 days | 1,91675 | A | |
| DHQ, 28 days | 1,36762 | A | B |
| DHQ, 42 days | 1,23887 | A | B |
| DHQ, 7 days | 1,14413 | A | B |
| DHQ, 56 days | 1,10755 | A | B |
| DHQ, 1 day | 0,75349 | | B |

Means that do not share a letter are significantly different.

###### Scheffe Test

| | Mean | Groups | |
|---|---|---|---|
| DHQ, 14 days | 1,91675 | A | |
| DHQ, 28 days | 1,36762 | A | B |
| DHQ, 42 days | 1,23887 | A | B |
| DHQ, 7 days | 1,14413 | A | B |
| DHQ, 56 days | 1,10755 | A | B |
| DHQ, 1 day | 0,75349 | | B |

Means that do not share a letter are significantly different.

###### Tukey Test

| | Mean | Groups | |
|---|---|---|---|
| DHQ, 14 days | 1,91675 | A | |
| DHQ, 28 days | 1,36762 | A | B |
| DHQ, 42 days | 1,23887 | A | B |
| DHQ, 7 days | 1,14413 | A | B |
| DHQ, 56 days | 1,10755 | A | B |
| DHQ, 1 day | 0,75349 | | B |

Means that do not share a letter are significantly different.

Sig equals 1 indicates that the difference of the means is significant at the 0,05 level.
Sig equals 0 indicates that the difference of the means is not significant at the 0,05 level.

#### Powers

| | Alpha | Sample Size | Power |
|---|---|---|---|
| Actual Power | 0,05 | 19 | 0,83245 |

*ANOVAOneWay (28.10.2024 12:34:47)*

## Notes

| | |
|---|---|
| Description | Perform One-Way ANOVA |
| User Name | yulia |
| Operation Time | 28.10.2024 12:34:47 |
| Report Status | New Analysis Report |
| Data Filter | No |

## Input Data

| | Data | Range |
|---|---|---|
| Control, 1 day | [Book1]Respiration!A"Control, 1 day | [1*:7*] |
| Control, 7 days | [Book1]Respiration!B"Control, 7 day | [1*:6*] |
| Control, 14 days | [Book1]Respiration!C"Control, 14 d ays" | [1*:6*] |
| Control, 28 days | [Book1]Respiration!D"Control, 28 d ays" | [1*:8*] |
| Control, 42 days | [Book1]Respiration!E"Control, 42 d ays" | [1*:5*] |
| Control, 56 days | [Book1]Respiration!F"Control, 56 d ays" | [1*:4*] |
| DHQ, 1 day | [Book1]Respiration!G"DHQ, 1 day" | [1*:6*] |
| DHQ, 7 days | [Book1]Respiration!H"DHQ, 7 days" | [1*:6*] |
| DHQ, 14 days | [Book1]Respiration!I"DHQ, 14 days" | [1*:7*] |
| DHQ, 28 days | [Book1]Respiration!J"DHQ, 28 days | [1*:8*] |
| DHQ, 42 days | [Book1]Respiration!K"DHQ, 42 days | [1*:5*] |
| DHQ, 56 days | [Book1]Respiration!L"DHQ, 56 days | [1*:4*] |

## Descriptive Statistics

| | N Analysis | N Missing | Mean | Standard Deviation | SE of Mean |
|---|---|---|---|---|---|
| Control, 1 day | 7 | 0 | 26,58161 | 6,46613 | 2,44397 |
| Control, 7 days | 6 | 0 | 16,4949 | 8,98435 | 3,66785 |
| Control, 14 days | 6 | 0 | 15,55272 | 5,76544 | 2,35373 |
| Control, 28 days | 8 | 0 | 19,24303 | 10,5332 | 3,72405 |
| Control, 42 days | 5 | 0 | 9,5173 | 1,80428 | 0,8069 |
| Control, 56 days | 4 | 0 | 6,5202 | 0,88101 | 0,44051 |
| DHQ, 1 day | 6 | 0 | 35,28931 | 13,42638 | 5,4813 |
| DHQ, 7 days | 6 | 0 | 19,92221 | 11,41206 | 4,65896 |
| DHQ, 14 days | 7 | 0 | 14,99683 | 7,10222 | 2,68439 |
| DHQ, 28 days | 8 | 0 | 19,78601 | 10,67839 | 3,77538 |
| DHQ, 42 days | 5 | 0 | 9,69144 | 2,4381 | 1,09035 |
| DHQ, 56 days | 4 | 0 | 6,30025 | 1,34946 | 0,67473 |

## ANOVA

### Overall ANOVA

| | DF | Sum of Squares | Mean Square | F Value | Prob>F |
|---|---|---|---|---|---|
| Model | 11 | 4254,73653 | 386,79423 | 5,4031 | 6,77427E-6 |
| Error | 60 | 4295,24907 | 71,58748 | | |
| Total | 71 | 8549,9856 | | | |

Null Hypothesis: The means of all levels are equal.
Alternative Hypothesis: The means of one or more levels are different.
At the 0.05 level, the population means are significantly different.

### Fit Statistics

| | R-Square | Coeff Var | Root MSE | Data Mean |
|---|---|---|---|---|
| | 0,49763 | 0,47811 | 8,46094 | 17,69669 |

### Means Comparisons

#### Bonferroni Test

| | MeanDiff | SEM | t Value | Prob | Alpha | Sig | LCL | UCL |
|---|---|---|---|---|---|---|---|---|
| Control, 7 days  Control, 1 day | -10,08671 | 4,70723 | -2,14281 | 1 | 0,05 | 0 | -26,79457 | 6,62115 |
| Control, 14 days  Control, 1 day | -11,02889 | 4,70723 | -2,34297 | 1 | 0,05 | 0 | -27,73675 | 5,67897 |
| Control, 14 days  Control, 7 days | -0,94218 | 4,88493 | -0,19287 | 1 | 0,05 | 0 | -18,28074 | 16,39639 |
| Control, 28 days  Control, 1 day | -7,33857 | 4,37895 | -1,67587 | 1 | 0,05 | 0 | -22,88123 | 8,20409 |
| Control, 28 days  Control, 7 days | 2,74814 | 4,56943 | 0,60142 | 1 | 0,05 | 0 | -13,4706 | 18,96688 |
| Control, 28 days  Control, 14 da | 3,69031 | 4,56943 | 0,80761 | 1 | 0,05 | 0 | -12,52843 | 19,90906 |
| Control, 42 days  Control, 1 day | -17,0643 | 4,95422 | -3,4444 | 0,0693 | 0,05 | 0 | -34,64882 | 0,52021 |
| Control, 42 days  Control, 7 days | -6,97759 | 5,12335 | -1,36192 | 1 | 0,05 | 0 | -25,16243 | 11,20725 |
| Control, 42 days  Control, 14 da | -6,03542 | 5,12335 | -1,17802 | 1 | 0,05 | 0 | -24,22026 | 12,14942 |
| Control, 42 days  Control, 28 da | -9,72573 | 4,82348 | -2,01633 | 1 | 0,05 | 0 | -26,84619 | 7,39473 |
| Control, 56 days  Control, 1 day | -20,06141 | 5,30317 | -3,78291 | 0,02375 | 0,05 | 1 | -38,88451 | -1,23831 |
| Control, 56 days  Control, 7 days | -9,9747 | 5,46151 | -1,82636 | 1 | 0,05 | 0 | -29,3598 | 9,41041 |
| Control, 56 days  Control, 14 da | -9,03252 | 5,46151 | -1,65385 | 1 | 0,05 | 0 | -28,41763 | 10,35258 |
| Control, 56 days  Control, 28 da | -12,72284 | 5,18125 | -2,45556 | 1 | 0,05 | 0 | -31,11316 | 5,66749 |
| Control, 56 days  Control, 42 da | -2,99711 | 5,67577 | -0,52805 | 1 | 0,05 | 0 | -23,1427 | 17,14848 |
| DHQ, 1 day  Control, 1 day | 8,7077 | 4,70723 | 1,84986 | 1 | 0,05 | 0 | -8,00015 | 25,41556 |
| DHQ, 1 day  Control, 7 days | 18,79441 | 4,88493 | 3,84743 | 0,01925 | 0,05 | 1 | 1,45585 | 36,13298 |
| DHQ, 1 day  Control, 14 days | 19,73659 | 4,88493 | 4,04031 | 0,01017 | 0,05 | 1 | 2,39803 | 37,07515 |
| DHQ, 1 day  Control, 28 days | 16,04628 | 4,56943 | 3,51166 | 0,05626 | 0,05 | 0 | -0,17247 | 32,26502 |
| DHQ, 1 day  Control, 42 days | 25,77201 | 5,12335 | 5,0303 | 3,12362E-4 | 0,05 | 1 | 7,58717 | 43,95685 |
| DHQ, 1 day  Control, 56 days | 28,76911 | 5,46151 | 5,26761 | 1,30024E-4 | 0,05 | 1 | 9,38401 | 48,15422 |
| DHQ, 7 days  Control, 1 day | -6,6594 | 4,70723 | -1,41472 | 1 | 0,05 | 0 | -23,36726 | 10,04846 |
| DHQ, 7 days  Control, 7 days | 3,42731 | 4,88493 | 0,70161 | 1 | 0,05 | 0 | -13,91125 | 20,76587 |
| DHQ, 7 days  Control, 14 days | 4,36948 | 4,88493 | 0,89448 | 1 | 0,05 | 0 | -12,96908 | 21,70805 |
| DHQ, 7 days  Control, 28 days | 0,67917 | 4,56943 | 0,14863 | 1 | 0,05 | 0 | -15,53957 | 16,89791 |
| DHQ, 7 days  Control, 42 days | 10,4049 | 5,12335 | 2,03088 | 1 | 0,05 | 0 | -7,77994 | 28,58974 |
| DHQ, 7 days  Control, 56 days | 13,40201 | 5,46151 | 2,4539 | 1 | 0,05 | 0 | -5,9831 | 32,78711 |
| DHQ, 7 days  DHQ, 1 day | -15,36711 | 4,88493 | -3,14582 | 0,1701 | 0,05 | 0 | -32,70567 | 1,97146 |
| DHQ, 14 days  Control, 1 day | -11,58478 | 4,52256 | -2,56155 | 0,8548 | 0,05 | 0 | -27,63717 | 4,46762 |
| DHQ, 14 days  Control, 7 days | -1,49806 | 4,70723 | -0,31825 | 1 | 0,05 | 0 | -18,20592 | 15,20979 |
| DHQ, 14 days  Control, 14 days | -0,55589 | 4,70723 | -0,11809 | 1 | 0,05 | 0 | -17,26375 | 16,15197 |
| DHQ, 14 days  Control, 28 days | -4,2462 | 4,37895 | -0,96968 | 1 | 0,05 | 0 | -19,78886 | 11,29646 |
| DHQ, 14 days  Control, 42 days | 5,47953 | 4,95422 | 1,10603 | 1 | 0,05 | 0 | -12,10499 | 23,06404 |
| DHQ, 14 days  Control, 56 days | 8,47663 | 5,30317 | 1,59841 | 1 | 0,05 | 0 | -10,34646 | 27,29973 |
| DHQ, 14 days  DHQ, 1 day | -20,29248 | 4,70723 | -4,31092 | 0,00405 | 0,05 | 1 | -37,00034 | -3,58462 |
| DHQ, 14 days  DHQ, 7 days | -4,92537 | 4,70723 | -1,04634 | 1 | 0,05 | 0 | -21,63323 | 11,78248 |
| DHQ, 28 days  Control, 1 day | -6,7956 | 4,37895 | -1,55188 | 1 | 0,05 | 0 | -22,33826 | 8,74706 |
| DHQ, 28 days  Control, 7 days | 3,29111 | 4,56943 | 0,72025 | 1 | 0,05 | 0 | -12,92763 | 19,50985 |
| DHQ, 28 days  Control, 14 days | 4,23329 | 4,56943 | 0,92644 | 1 | 0,05 | 0 | -11,98546 | 20,45203 |
| DHQ, 28 days  Control, 28 days | 0,54297 | 4,23047 | 0,12835 | 1 | 0,05 | 0 | -14,47266 | 15,55861 |
| DHQ, 28 days  Control, 42 days | 10,2687 | 4,82348 | 2,1289 | 1 | 0,05 | 0 | -6,85176 | 27,38916 |
| DHQ, 28 days  Control, 56 days | 13,26581 | 5,18125 | 2,56035 | 0,85746 | 0,05 | 0 | -5,12452 | 31,65613 |
| DHQ, 28 days  DHQ, 1 day | -15,5033 | 4,56943 | -3,39283 | 0,08119 | 0,05 | 0 | -31,72205 | 0,71544 |
| DHQ, 28 days  DHQ, 7 days | -0,1362 | 4,56943 | -0,02981 | 1 | 0,05 | 0 | -16,35494 | 16,08254 |
| DHQ, 28 days  DHQ, 14 days | 4,78918 | 4,37895 | 1,09368 | 1 | 0,05 | 0 | -10,75348 | 20,33184 |
| DHQ, 42 days  Control, 1 day | -16,89017 | 4,95422 | -3,40925 | 0,07721 | 0,05 | 0 | -34,47468 | 0,69434 |
| DHQ, 42 days  Control, 7 days | -6,80346 | 5,12335 | -1,32793 | 1 | 0,05 | 0 | -24,9883 | 11,38138 |
| DHQ, 42 days  Control, 14 days | -5,86128 | 5,12335 | -1,14403 | 1 | 0,05 | 0 | -24,04612 | 12,32356 |
| DHQ, 42 days  Control, 28 days | -9,5516 | 4,82348 | -1,98023 | 1 | 0,05 | 0 | -26,67206 | 7,56886 |
| DHQ, 42 days  Control, 42 days | 0,17413 | 5,35117 | 0,03254 | 1 | 0,05 | 0 | -18,81931 | 19,16758 |
| DHQ, 42 days  Control, 56 days | 3,17124 | 5,67577 | 0,55873 | 1 | 0,05 | 0 | -16,97435 | 23,31683 |
| DHQ, 42 days  DHQ, 1 day | -25,59787 | 5,12335 | -4,99631 | 3,53747E-4 | 0,05 | 1 | -43,78271 | -7,41303 |
| DHQ, 42 days  DHQ, 7 days | -10,23077 | 5,12335 | -1,99689 | 1 | 0,05 | 0 | -28,41561 | 7,95407 |
| DHQ, 42 days  DHQ, 14 days | -5,30539 | 4,95422 | -1,07088 | 1 | 0,05 | 0 | -22,88991 | 12,27912 |
| DHQ, 42 days  DHQ, 28 days | -10,09457 | 4,82348 | -2,0928 | 1 | 0,05 | 0 | -27,21503 | 7,02589 |
| DHQ, 56 days  Control, 1 day | -20,28136 | 5,30317 | -3,82438 | 0,02076 | 0,05 | 1 | -39,10445 | -1,45826 |
| DHQ, 56 days  Control, 7 days | -10,19465 | 5,46151 | -1,86663 | 1 | 0,05 | 0 | -29,57975 | 9,19046 |
| DHQ, 56 days  Control, 14 days | -9,25247 | 5,46151 | -1,69412 | 1 | 0,05 | 0 | -28,63757 | 10,13263 |
| DHQ, 56 days  Control, 28 days | -12,94278 | 5,18125 | -2,49801 | 1 | 0,05 | 0 | -31,33311 | 5,44754 |
| DHQ, 56 days  Control, 42 days | -3,21705 | 5,67577 | -0,5668 | 1 | 0,05 | 0 | -23,36265 | 16,92854 |
| DHQ, 56 days  Control, 56 days | -0,21995 | 5,98279 | -0,03676 | 1 | 0,05 | 0 | -21,45527 | 21,01537 |
| DHQ, 56 days  DHQ, 1 day | -28,98906 | 5,46151 | -5,30788 | 1,11913E-4 | 0,05 | 1 | -48,37416 | -9,60396 |
| DHQ, 56 days  DHQ, 7 days | -13,62196 | 5,46151 | -2,49417 | 1 | 0,05 | 0 | -33,00706 | 5,76315 |
| DHQ, 56 days  DHQ, 14 days | -8,69658 | 5,30317 | -1,63988 | 1 | 0,05 | 0 | -27,51968 | 10,12652 |
| DHQ, 56 days  DHQ, 28 days | -13,48576 | 5,18125 | -2,6028 | 0,76781 | 0,05 | 0 | -31,87608 | 4,90457 |
| DHQ, 56 days  DHQ, 42 days | -3,39119 | 5,67577 | -0,59748 | 1 | 0,05 | 0 | -23,53678 | 16,7544 |

#### Scheffe Test

| | MeanDiff | SEM | F Value | Prob | Alpha | Sig | LCL | UCL |
|---|---|---|---|---|---|---|---|---|
| Control, 7 days  Control, 1 day | -10,08671 | 4,70723 | 0,41742 | 0,94273 | 0,05 | 0 | -31,90021 | 11,72679 |
| Control, 14 days  Control, 1 day | -11,02889 | 4,70723 | 0,49904 | 0,89644 | 0,05 | 0 | -32,84239 | 10,78462 |
| Control, 14 days  Control, 7 days | -0,94218 | 4,88493 | 0,00338 | 1 | 0,05 | 0 | -23,57912 | 21,69477 |
| Control, 28 days  Control, 1 day | -7,33857 | 4,37895 | 0,25532 | 0,99142 | 0,05 | 0 | -27,63081 | 12,95367 |
| Control, 28 days  Control, 7 days | 2,74814 | 4,56943 | 0,03288 | 1 | 0,05 | 0 | -18,42678 | 23,92306 |
| Control, 28 days  Control, 14 da | 3,69031 | 4,56943 | 0,05929 | 0,99999 | 0,05 | 0 | -17,48461 | 24,86523 |
| Control, 42 days  Control, 1 day | -17,0643 | 4,95422 | 1,07853 | 0,39352 | 0,05 | 0 | -40,02235 | 5,89375 |
| Control, 42 days  Control, 7 days | -6,97759 | 5,12335 | 0,16862 | 0,99863 | 0,05 | 0 | -30,71942 | 16,76423 |
| Control, 42 days  Control, 14 da | -6,03542 | 5,12335 | 0,12616 | 0,99965 | 0,05 | 0 | -29,77724 | 17,70641 |
| Control, 42 days  Control, 28 da | -9,72573 | 4,82348 | 0,3696 | 0,96296 | 0,05 | 0 | -32,07792 | 12,62646 |
| Control, 56 days  Control, 1 day | -20,06141 | 5,30317 | 1,30094 | 0,24635 | 0,05 | 0 | -44,63653 | 4,51371 |
| Control, 56 days  Control, 7 days | -9,9747 | 5,46151 | 0,30324 | 0,98265 | 0,05 | 0 | -35,28357 | 15,33417 |
| Control, 56 days  Control, 14 da | -9,03252 | 5,46151 | 0,24866 | 0,99232 | 0,05 | 0 | -34,34139 | 16,27635 |
| Control, 56 days  Control, 28 da | -12,72284 | 5,18125 | 0,54816 | 0,86207 | 0,05 | 0 | -36,73294 | 11,28727 |
| Control, 56 days  Control, 42 da | -2,99711 | 5,67577 | 0,02535 | 1 | 0,05 | 0 | -29,29886 | 23,30464 |
| DHQ, 1 day  Control, 1 day | 8,7077 | 4,70723 | 0,31109 | 0,98081 | 0,05 | 0 | -13,1058 | 30,52121 |
| DHQ, 1 day  Control, 7 days | 18,79441 | 4,88493 | 1,3457 | 0,22277 | 0,05 | 0 | -3,84253 | 41,43136 |
| DHQ, 1 day  Control, 14 days | 19,73659 | 4,88493 | 1,48401 | 0,16149 | 0,05 | 0 | -2,90035 | 42,37353 |
| DHQ, 1 day  Control, 28 days | 16,04628 | 4,56943 | 1,12107 | 0,36147 | 0,05 | 0 | -5,12864 | 37,2212 |
| DHQ, 1 day  Control, 42 days | 25,77201 | 5,12335 | 2,30036 | 0,01997 | 0,05 | 1 | 2,03018 | 49,51383 |
| DHQ, 1 day  Control, 56 days | 28,76911 | 5,46151 | 2,52252 | 0,01102 | 0,05 | 1 | 3,46024 | 54,07798 |
| DHQ, 7 days  Control, 1 day | -6,6594 | 4,70723 | 0,18195 | 0,99806 | 0,05 | 0 | -28,4729 | 15,1541 |
| DHQ, 7 days  Control, 7 days | 3,42731 | 4,88493 | 0,04475 | 1 | 0,05 | 0 | -19,20963 | 26,06425 |
| DHQ, 7 days  Control, 14 days | 4,36948 | 4,88493 | 0,07274 | 0,99998 | 0,05 | 0 | -18,26746 | 27,00643 |
| DHQ, 7 days  Control, 28 days | 0,67917 | 4,56943 | 0,00201 | 1 | 0,05 | 0 | -20,49575 | 21,85409 |
| DHQ, 7 days  Control, 42 days | 10,4049 | 5,12335 | 0,37495 | 0,96095 | 0,05 | 0 | -13,33692 | 34,14673 |
| DHQ, 7 days  Control, 56 days | 13,40201 | 5,46151 | 0,54742 | 0,86262 | 0,05 | 0 | -11,90686 | 38,71088 |
| DHQ, 7 days  DHQ, 1 day | -15,36711 | 4,88493 | 0,89965 | 0,54604 | 0,05 | 0 | -38,00405 | 7,26984 |
| DHQ, 14 days  Control, 1 day | -11,58478 | 4,52256 | 0,5965 | 0,82421 | 0,05 | 0 | -32,54251 | 9,37296 |
| DHQ, 14 days  Control, 7 days | -1,49806 | 4,70723 | 0,00921 | 1 | 0,05 | 0 | -23,31157 | 20,31544 |
| DHQ, 14 days  Control, 14 days | -0,55589 | 4,70723 | 0,00127 | 1 | 0,05 | 0 | -22,36939 | 21,25761 |
| DHQ, 14 days  Control, 28 days | -4,2462 | 4,37895 | 0,08548 | 0,99995 | 0,05 | 0 | -24,53844 | 16,04604 |
| DHQ, 14 days  Control, 42 days | 5,47953 | 4,95422 | 0,11121 | 0,99981 | 0,05 | 0 | -17,47852 | 28,43758 |
| DHQ, 14 days  Control, 56 days | 8,47663 | 5,30317 | 0,23226 | 0,99426 | 0,05 | 0 | -16,09849 | 33,05176 |
| DHQ, 14 days  DHQ, 1 day | -20,29248 | 4,70723 | 1,68945 | 0,09778 | 0,05 | 0 | -42,10598 | 1,52102 |
| DHQ, 14 days  DHQ, 7 days | -4,92537 | 4,70723 | 0,09953 | 0,99989 | 0,05 | 0 | -26,73888 | 16,88813 |
| DHQ, 28 days  Control, 1 day | -6,7956 | 4,37895 | 0,21894 | 0,99556 | 0,05 | 0 | -27,08784 | 13,49664 |
| DHQ, 28 days  Control, 7 days | 3,29111 | 4,56943 | 0,04716 | 1 | 0,05 | 0 | -17,88381 | 24,46603 |
| DHQ, 28 days  Control, 14 days | 4,23329 | 4,56943 | 0,07803 | 0,99997 | 0,05 | 0 | -16,94163 | 25,40821 |
| DHQ, 28 days  Control, 28 days | 0,54297 | 4,23047 | 0,0015 | 1 | 0,05 | 0 | -19,06119 | 20,14714 |
| DHQ, 28 days  Control, 42 days | 10,2687 | 4,82348 | 0,41202 | 0,94527 | 0,05 | 0 | -12,08349 | 32,62089 |
| DHQ, 28 days  Control, 56 days | 13,26581 | 5,18125 | 0,59595 | 0,82467 | 0,05 | 0 | -10,74429 | 37,27591 |
| DHQ, 28 days  DHQ, 1 day | -15,5033 | 4,56943 | 1,04648 | 0,41884 | 0,05 | 0 | -36,67822 | 5,67162 |
| DHQ, 28 days  DHQ, 7 days | -0,1362 | 4,56943 | 8,07656 | 1 | 0,05 | 0 | -21,31112 | 21,03872 |
| DHQ, 28 days  DHQ, 14 days | 4,78918 | 4,37895 | 0,10874 | 0,99983 | 0,05 | 0 | -15,50306 | 25,08142 |
| DHQ, 42 days  Control, 1 day | -16,89017 | 4,95422 | 1,05664 | 0,41071 | 0,05 | 0 | -39,84822 | 6,06788 |
| DHQ, 42 days  Control, 7 days | -6,80346 | 5,12335 | 0,16031 | 0,99891 | 0,05 | 0 | -30,54528 | 16,93837 |
| DHQ, 42 days  Control, 14 days | -5,86128 | 5,12335 | 0,11898 | 0,99974 | 0,05 | 0 | -29,60311 | 17,88054 |
| DHQ, 42 days  Control, 28 days | -9,5516 | 4,82348 | 0,35648 | 0,96761 | 0,05 | 0 | -31,90379 | 12,80059 |
| DHQ, 42 days  Control, 42 days | 0,17413 | 5,35117 | 9,62655 | 1 | 0,05 | 0 | -24,62339 | 24,97166 |
| DHQ, 42 days  Control, 56 days | 3,17124 | 5,67577 | 0,02838 | 1 | 0,05 | 0 | -23,13051 | 29,47299 |
| DHQ, 42 days  DHQ, 1 day | -25,59787 | 5,12335 | 2,26938 | 0,02169 | 0,05 | 1 | -49,3397 | -1,85605 |
| DHQ, 42 days  DHQ, 7 days | -10,23077 | 5,12335 | 0,36251 | 0,96552 | 0,05 | 0 | -33,97259 | 13,51106 |
| DHQ, 42 days  DHQ, 14 days | -5,30539 | 4,95422 | 0,10425 | 0,99986 | 0,05 | 0 | -28,26344 | 17,65265 |
| DHQ, 42 days  DHQ, 28 days | -10,09457 | 4,82348 | 0,39816 | 0,9515 | 0,05 | 0 | -32,44676 | 12,25762 |
| DHQ, 56 days  Control, 1 day | -20,28136 | 5,30317 | 1,32963 | 0,23101 | 0,05 | 0 | -44,85648 | 4,29377 |
| DHQ, 56 days  Control, 7 days | -10,19465 | 5,46151 | 0,31676 | 0,97939 | 0,05 | 0 | -35,50352 | 15,11422 |
| DHQ, 56 days  Control, 14 days | -9,25247 | 5,46151 | 0,26091 | 0,9906 | 0,05 | 0 | -34,56134 | 16,0564 |
| DHQ, 56 days  Control, 28 days | -12,94278 | 5,18125 | 0,56728 | 0,84754 | 0,05 | 0 | -36,95289 | 11,06732 |
| DHQ, 56 days  Control, 42 days | -3,21705 | 5,67577 | 0,02921 | 1 | 0,05 | 0 | -29,5188 | 23,08469 |
| DHQ, 56 days  Control, 56 days | -0,21995 | 5,98279 | 1,22869 | 1 | 0,05 | 0 | -27,94443 | 27,50453 |
| DHQ, 56 days  DHQ, 1 day | -28,98906 | 5,46151 | 2,56124 | 0,00993 | 0,05 | 1 | -54,29793 | -3,68019 |
| DHQ, 56 days  DHQ, 7 days | -13,62196 | 5,46151 | 0,56554 | 0,84888 | 0,05 | 0 | -38,93083 | 11,68691 |
| DHQ, 56 days  DHQ, 14 days | -8,69658 | 5,30317 | 0,24447 | 0,99285 | 0,05 | 0 | -33,2717 | 15,87854 |
| DHQ, 56 days  DHQ, 28 days | -13,48576 | 5,18125 | 0,61587 | 0,8081 | 0,05 | 0 | -37,49586 | 10,52435 |
| DHQ, 56 days  DHQ, 42 days | -3,39119 | 5,67577 | 0,03245 | 1 | 0,05 | 0 | -29,69294 | 22,91056 |

#### Tukey Test

| | MeanDiff | SEM | q Value | Prob | Alpha | Sig | LCL | UCL |
|---|---|---|---|---|---|---|---|---|
| Control, 7 days  Control, 1 day | -10,08671 | 4,70723 | 3,03039 | 0,59542 | 0,05 | 0 | -26,0918 | 5,91838 |
| Control, 14 days  Control, 1 day | -11,02889 | 4,70723 | 3,31346 | 0,45922 | 0,05 | 0 | -27,03398 | 4,9762 |
| Control, 14 days  Control, 7 days | -0,94218 | 4,88493 | 0,27277 | 1 | 0,05 | 0 | -17,55144 | 15,66709 |
| Control, 28 days  Control, 1 day | -7,33857 | 4,37895 | 2,37004 | 0,87185 | 0,05 | 0 | -22,22748 | 7,55033 |
| Control, 28 days  Control, 7 days | 2,74814 | 4,56943 | 0,85053 | 0,99998 | 0,05 | 0 | -12,78841 | 18,28469 |
| Control, 28 days  Control, 14 da | 3,69031 | 4,56943 | 1,14213 | 0,99959 | 0,05 | 0 | -11,84623 | 19,22686 |
| Control, 42 days  Control, 1 day | -17,0643 | 4,95422 | 4,87112 | 0,04442 | 0,05 | 1 | -33,90917 | -0,21943 |
| Control, 42 days  Control, 7 days | -6,97759 | 5,12335 | 1,92604 | 0,96631 | 0,05 | 0 | -24,39754 | 10,44236 |
| Control, 42 days  Control, 14 da | -6,03542 | 5,12335 | 1,66597 | 0,98877 | 0,05 | 0 | -23,45536 | 11,38453 |
| Control, 42 days  Control, 28 da | -9,72573 | 4,82348 | 2,85152 | 0,6808 | 0,05 | 0 | -26,12607 | 6,67461 |
| Control, 56 days  Control, 1 day | -20,06141 | 5,30317 | 5,34984 | 0,01706 | 0,05 | 1 | -38,09277 | -2,03005 |
| Control, 56 days  Control, 7 days | -9,9747 | 5,46151 | 2,58287 | 0,79711 | 0,05 | 0 | -28,54442 | 8,59503 |
| Control, 56 days  Control, 14 da | -9,03252 | 5,46151 | 2,3389 | 0,88121 | 0,05 | 0 | -27,60225 | 9,5372 |
| Control, 56 days  Control, 28 da | -12,72284 | 5,18125 | 3,47268 | 0,38697 | 0,05 | 0 | -30,33962 | 4,89395 |
| Control, 56 days  Control, 42 da | -2,99711 | 5,67577 | 0,74678 | 0,99999 | 0,05 | 0 | -22,29533 | 16,30112 |
| DHQ, 1 day  Control, 1 day | 8,7077 | 4,70723 | 2,61609 | 0,78388 | 0,05 | 0 | -7,29739 | 24,71279 |
| DHQ, 1 day  Control, 7 days | 18,79441 | 4,88493 | 5,44109 | 0,01408 | 0,05 | 1 | 2,18515 | 35,40368 |
| DHQ, 1 day  Control, 14 days | 19,73659 | 4,88493 | 5,71385 | 0,00781 | 0,05 | 1 | 3,12732 | 36,34586 |
| DHQ, 1 day  Control, 28 days | 16,04628 | 4,56943 | 4,96624 | 0,03699 | 0,05 | 1 | 0,50973 | 31,58282 |
| DHQ, 1 day  Control, 42 days | 25,77201 | 5,12335 | 7,11392 | 2,78097E-4 | 0,05 | 1 | 8,35206 | 43,19195 |
| DHQ, 1 day  Control, 56 days | 28,76911 | 5,46151 | 7,44952 | 1,18099E-4 | 0,05 | 1 | 10,19939 | 47,33884 |
| DHQ, 7 days  Control, 1 day | -6,6594 | 4,70723 | 2,00071 | 0,95599 | 0,05 | 0 | -22,66449 | 9,34569 |
| DHQ, 7 days  Control, 7 days | 3,42731 | 4,88493 | 0,99223 | 0,9999 | 0,05 | 0 | -13,18196 | 20,03658 |
| DHQ, 7 days  Control, 14 days | 4,36948 | 4,88493 | 1,26499 | 0,99895 | 0,05 | 0 | -12,23978 | 20,97875 |
| DHQ, 7 days  Control, 28 days | 0,67917 | 4,56943 | 0,2102 | 1 | 0,05 | 0 | -14,85738 | 16,21572 |
| DHQ, 7 days  Control, 42 days | 10,4049 | 5,12335 | 2,87209 | 0,67119 | 0,05 | 0 | -7,01505 | 27,82485 |
| DHQ, 7 days  Control, 56 days | 13,40201 | 5,46151 | 3,47034 | 0,388 | 0,05 | 0 | -5,16772 | 31,97173 |
| DHQ, 7 days  DHQ, 1 day | -15,36711 | 4,88493 | 4,44886 | 0,09534 | 0,05 | 0 | -31,97637 | 1,24216 |
| DHQ, 14 days  Control, 1 day | -11,58478 | 4,52256 | 3,62258 | 0,32419 | 0,05 | 0 | -26,96197 | 3,79242 |
| DHQ, 14 days  Control, 7 days | -1,49806 | 4,70723 | 0,45007 | 1 | 0,05 | 0 | -17,50316 | 14,50703 |
| DHQ, 14 days  Control, 14 days | -0,55589 | 4,70723 | 0,16701 | 1 | 0,05 | 0 | -16,56098 | 15,4492 |
| DHQ, 14 days  Control, 28 days | -4,2462 | 4,37895 | 1,37134 | 0,99783 | 0,05 | 0 | -19,13511 | 10,6427 |
| DHQ, 14 days  Control, 42 days | 5,47953 | 4,95422 | 1,56417 | 0,99328 | 0,05 | 0 | -11,36535 | 22,3244 |
| DHQ, 14 days  Control, 56 days | 8,47663 | 5,30317 | 2,26049 | 0,90291 | 0,05 | 0 | -9,55473 | 26,50799 |
| DHQ, 14 days  DHQ, 1 day | -20,29248 | 4,70723 | 6,09656 | 0,00329 | 0,05 | 1 | -36,29757 | -4,28739 |
| DHQ, 14 days  DHQ, 7 days | -4,92537 | 4,70723 | 1,47975 | 0,99578 | 0,05 | 0 | -20,93047 | 11,07972 |
| DHQ, 28 days  Control, 1 day | -6,7956 | 4,37895 | 2,19469 | 0,91905 | 0,05 | 0 | -21,6845 | 8,0933 |
| DHQ, 28 days  Control, 7 days | 3,29111 | 4,56943 | 1,01858 | 0,99986 | 0,05 | 0 | -12,24544 | 18,82766 |
| DHQ, 28 days  Control, 14 days | 4,23329 | 4,56943 | 1,31018 | 0,99855 | 0,05 | 0 | -11,30326 | 19,76983 |
| DHQ, 28 days  Control, 28 days | 0,54297 | 4,23047 | 0,18151 | 1 | 0,05 | 0 | -13,84107 | 14,92702 |
| DHQ, 28 days  Control, 42 days | 10,2687 | 4,82348 | 3,01072 | 0,60495 | 0,05 | 0 | -6,13164 | 26,66904 |
| DHQ, 28 days  Control, 56 days | 13,26581 | 5,18125 | 3,62088 | 0,32487 | 0,05 | 0 | -4,35098 | 30,8826 |
| DHQ, 28 days  DHQ, 1 day | -15,5033 | 4,56943 | 4,79819 | 0,05097 | 0,05 | 0 | -31,03985 | 0,03324 |
| DHQ, 28 days  DHQ, 7 days | -0,1362 | 4,56943 | 0,04215 | 1 | 0,05 | 0 | -15,67275 | 15,40035 |
| DHQ, 28 days  DHQ, 14 days | 4,78918 | 4,37895 | 1,5467 | 0,99388 | 0,05 | 0 | -10,09973 | 19,67808 |
| DHQ, 42 days  Control, 1 day | -16,89017 | 4,95422 | 4,82141 | 0,0488 | 0,05 | 1 | -33,73504 | -0,0453 |
| DHQ, 42 days  Control, 7 days | -6,80346 | 5,12335 | 1,87798 | 0,97193 | 0,05 | 0 | -24,22341 | 10,61649 |
| DHQ, 42 days  Control, 14 days | -5,86128 | 5,12335 | 1,61791 | 0,99113 | 0,05 | 0 | -23,28123 | 11,55866 |
| DHQ, 42 days  Control, 28 days | -9,5516 | 4,82348 | 2,80047 | 0,70432 | 0,05 | 0 | -25,95193 | 6,84874 |
| DHQ, 42 days  Control, 42 days | 0,17413 | 5,35117 | 0,04602 | 1 | 0,05 | 0 | -18,02041 | 18,36867 |
| DHQ, 42 days  Control, 56 days | 3,17124 | 5,67577 | 0,79017 | 0,99999 | 0,05 | 0 | -16,12699 | 22,46946 |
| DHQ, 42 days  DHQ, 1 day | -25,59787 | 5,12335 | 7,06585 | 3,13932E-4 | 0,05 | 1 | -43,01782 | -8,17793 |
| DHQ, 42 days  DHQ, 7 days | -10,23077 | 5,12335 | 2,82403 | 0,69353 | 0,05 | 0 | -27,65072 | 7,18918 |
| DHQ, 42 days  DHQ, 14 days | -5,30539 | 4,95422 | 1,51446 | 0,99487 | 0,05 | 0 | -22,15027 | 11,53948 |
| DHQ, 42 days  DHQ, 28 days | -10,09457 | 4,82348 | 2,95967 | 0,62958 | 0,05 | 0 | -26,49491 | 6,30577 |
| DHQ, 56 days  Control, 1 day | -20,28136 | 5,30317 | 5,40849 | 0,01509 | 0,05 | 1 | -38,31272 | -2,25 |
| DHQ, 56 days  Control, 7 days | -10,19465 | 5,46151 | 2,63982 | 0,77421 | 0,05 | 0 | -28,76437 | 8,37508 |
| DHQ, 56 days  Control, 14 days | -9,25247 | 5,46151 | 2,39585 | 0,86377 | 0,05 | 0 | -27,8222 | 9,31726 |
| DHQ, 56 days  Control, 28 days | -12,94278 | 5,18125 | 3,53271 | 0,36114 | 0,05 | 0 | -30,55957 | 4,674 |
| DHQ, 56 days  Control, 42 days | -3,21705 | 5,67577 | 0,80158 | 0,99999 | 0,05 | 0 | -22,51528 | 16,08117 |
| DHQ, 56 days  Control, 56 days | -0,21995 | 5,98279 | 0,05199 | 1 | 0,05 | 0 | -20,56206 | 20,12217 |
| DHQ, 56 days  DHQ, 1 day | -28,98906 | 5,46151 | 7,50648 | 1,01955E-4 | 0,05 | 1 | -47,55879 | -10,4193 |
| DHQ, 56 days  DHQ, 7 days | -13,62196 | 5,46151 | 3,52729 | 0,36344 | 0,05 | 0 | -32,19168 | 4,94777 |
| DHQ, 56 days  DHQ, 14 days | -8,69658 | 5,30317 | 2,31914 | 0,88693 | 0,05 | 0 | -26,72794 | 9,33478 |
| DHQ, 56 days  DHQ, 28 days | -13,48576 | 5,18125 | 3,68092 | 0,30139 | 0,05 | 0 | -31,10255 | 4,13103 |
| DHQ, 56 days  DHQ, 42 days | -3,39119 | 5,67577 | 0,84497 | 0,99998 | 0,05 | 0 | -22,68941 | 15,90704 |

### Grouping Letters

#### Bonferroni Test

| | Mean | Groups | | |
|---|---|---|---|---|
| DHQ, 1 day | 35,28931 | A | | |
| Control, 1 day | 26,58161 | A | B | |
| DHQ, 7 days | 19,92221 | A | B | C |
| DHQ, 28 days | 19,78601 | A | B | C |
| Control, 28 days | 19,24303 | A | B | C |
| Control, 7 days | 16,4949 | | B | C |
| Control, 14 days | 15,55272 | | B | C |
| DHQ, 14 days | 14,99683 | | B | C |
| DHQ, 42 days | 9,69144 | | B | C |
| Control, 42 days | 9,5173 | | B | C |
| Control, 56 days | 6,5202 | | | C |
| DHQ, 56 days | 6,30025 | | | C |

Means that do not share a letter are significantly different.

#### Scheffe Test

| | Mean | Groups | |
|---|---|---|---|
| DHQ, 1 day | 35,28931 | A | |
| Control, 1 day | 26,58161 | A | B |
| DHQ, 7 days | 19,92221 | A | B |
| DHQ, 28 days | 19,78601 | A | B |
| Control, 28 days | 19,24303 | A | B |
| Control, 7 days | 16,4949 | A | B |
| Control, 14 days | 15,55272 | A | B |
| DHQ, 14 days | 14,99683 | A | B |
| DHQ, 42 days | 9,69144 | | B |
| Control, 42 days | 9,5173 | | B |
| Control, 56 days | 6,5202 | | B |
| DHQ, 56 days | 6,30025 | | B |

Means that do not share a letter are significantly different.

#### Tukey Test

| | Mean | Groups | | |
|---|---|---|---|---|
| DHQ, 1 day | 35,28931 | A | | |
| Control, 1 day | 26,58161 | A | B | |
| DHQ, 7 days | 19,92221 | A | B | C |
| DHQ, 28 days | 19,78601 | A | B | C |
| Control, 28 days | 19,24303 | | B | C |
| Control, 7 days | 16,4949 | | B | C |
| Control, 14 days | 15,55272 | | B | C |
| DHQ, 14 days | 14,99683 | | B | C |
| DHQ, 42 days | 9,69144 | | | C |
| Control, 42 days | 9,5173 | | | C |
| Control, 56 days | 6,5202 | | | C |
| DHQ, 56 days | 6,30025 | | | C |

Means that do not share a letter are significantly different.

Sig equals 1 indicates that the difference of the means is significant at the 0,05 level.
Sig equals 0 indicates that the difference of the means is not significant at the 0,05 level.

### Powers

| | Alpha | Sample Size | Power |
|---|---|---|---|
| Actual Power | 0,05 | 72 | 0,99982 |

## *ANOVAOneWay (28.10.2024 12:47:56)*

### *Notes*

| | |
|---|---|
| Description | Perform One-Way ANOVA |
| User Name | yulia |
| Operation Time | 28.10.2024 12:47:56 |
| Report Status | New Analysis Report |
| Data Filter | No |

### *Input Data*

| | Data | Range |
|---|---|---|
| Control, 7 days | [Book1]JC-1!B"Control, 7 days" | [1*:4*] |
| | [Book1]JC-1!B"Control, 7 days" | [1*:4*] |
| Control, 14 days | [Book1]JC-1!C"Control, 14 days | [1*:4*] |
| Control, 28 days | [Book1]JC-1!D"Control, 28 days | [1*:5*] |
| Control, 42 days | [Book1]JC-1!E"Control, 42 days | [1*:3*] |
| Control, 56 days | [Book1]JC-1!F"Control, 56 days | [1*:3*] |
| DHQ, 1 day | [Book1]JC-1!G"DHQ, 1 day" | [1*:3*] |
| DHQ, 7 days | [Book1]JC-1!H"DHQ, 7 days" | [1*:3*] |
| DHQ, 14 days | [Book1]JC-1!I"DHQ, 14 days" | [1*:3*] |
| DHQ, 28 days | [Book1]JC-1!J"DHQ, 28 days" | [1*:4*] |
| DHQ, 42 days | [Book1]JC-1!K"DHQ, 42 days" | [1*:3*] |
| DHQ, 56 days | [Book1]JC-1!L"DHQ, 56 days" | [1*:3*] |

### *Descriptive Statistics*

| | N Analysis | N Missing | Mean | Standard Deviation | SE of Mean |
|---|---|---|---|---|---|
| Control, 7 days | 4 | 0 | 1,33698 | 0,22736 | 0,11368 |
| | 4 | 0 | 1,33698 | 0,22736 | 0,11368 |
| Control, 14 days | 4 | 0 | 1,2456 | 0,27933 | 0,13967 |
| Control, 28 days | 5 | 0 | 0,69605 | 0,31921 | 0,14275 |
| Control, 42 days | 3 | 0 | 0,82293 | 0,1687 | 0,0974 |
| Control, 56 days | 3 | 0 | 0,33009 | 0,11116 | 0,06418 |
| DHQ, 1 day | 3 | 0 | 0,99191 | 0,4011 | 0,23157 |
| DHQ, 7 days | 3 | 0 | 1,34417 | 0,24789 | 0,14312 |
| DHQ, 14 days | 3 | 0 | 1,61333 | 0,49313 | 0,28471 |
| DHQ, 28 days | 4 | 0 | 0,89324 | 0,24513 | 0,12256 |
| DHQ, 42 days | 3 | 0 | 0,87044 | 0,17692 | 0,10214 |
| DHQ, 56 days | 3 | 0 | 0,29444 | 0,01098 | 0,00634 |

### *ANOVA*

#### *Overall ANOVA*

| | DF | Sum of Squares | Mean Square | F Value | Prob>F |
|---|---|---|---|---|---|
| Model | 11 | 6,12037 | 0,5564 | 7,56123 | 4,9129E-6 |
| Error | 30 | 2,20756 | 0,07359 | | |
| Total | 41 | 8,32793 | | | |

Null Hypothesis: The means of all levels are equal.
Alternative Hypothesis: The means of one or more levels are different.
At the 0.05 level, the population means are significantly different.

#### *Fit Statistics*

| | R-Square | Coeff Var | Root MSE | Data Mean |
|---|---|---|---|---|
| | 0,73492 | 0,27431 | 0,27127 | 0,98889 |

#### *Means Comparisons*

##### *Bonferroni Test*

| | MeanDiff | SEM | t Value | Prob | Alpha | Sig | LCL | UCL |
|---|---|---|---|---|---|---|---|---|
| Control, 7 days Control, 7 days | 0 | 0,19181 | 0 | 1 | 0,05 | 0 | -0,71909 | 0,71909 |
| Control, 14 days Control, 7 days | -0,09139 | 0,19181 | -0,47643 | 1 | 0,05 | 0 | -0,81048 | 0,62771 |
| | -0,09139 | 0,19181 | -0,47643 | 1 | 0,05 | 0 | -0,81048 | 0,62771 |
| Control, 28 days Control, 7 days | -0,64094 | 0,18197 | -3,52218 | 0,09189 | 0,05 | 0 | -1,32313 | 0,04126 |
| | -0,64094 | 0,18197 | -3,52218 | 0,09189 | 0,05 | 0 | -1,32313 | 0,04126 |
| Control, 28 days Control, 14 da | -0,54955 | 0,18197 | -3,01999 | 0,33827 | 0,05 | 0 | -1,23174 | 0,13264 |
| Control, 42 days Control, 7 days | -0,51406 | 0,20718 | -2,48117 | 1 | 0,05 | 0 | -1,29077 | 0,26265 |
| | -0,51406 | 0,20718 | -2,48117 | 1 | 0,05 | 0 | -1,29077 | 0,26265 |
| Control, 42 days Control, 14 da | -0,42267 | 0,20718 | -2,04009 | 1 | 0,05 | 0 | -1,19938 | 0,35404 |
| Control, 42 days Control, 28 da | 0,12688 | 0,19811 | 0,64046 | 1 | 0,05 | 0 | -0,6158 | 0,86955 |
| Control, 56 days Control, 7 days | -1,0069 | 0,20718 | -4,85994 | 0,00228 | 0,05 | 1 | -1,78361 | -0,23019 |
| | -1,0069 | 0,20718 | -4,85994 | 0,00228 | 0,05 | 1 | -1,78361 | -0,23019 |
| Control, 56 days Control, 14 da | -0,91551 | 0,20718 | -4,41885 | 0,00788 | 0,05 | 1 | -1,69222 | -0,1388 |
| Control, 56 days Control, 28 da | -0,36596 | 0,19811 | -1,84731 | 1 | 0,05 | 0 | -1,10864 | 0,37671 |
| Control, 56 days Control, 42 da | -0,49284 | 0,22149 | -2,22513 | 1 | 0,05 | 0 | -1,32318 | 0,3375 |
| DHQ, 1 day Control, 7 days | -0,34508 | 0,20718 | -1,66557 | 1 | 0,05 | 0 | -1,12179 | 0,43163 |
| | -0,34508 | 0,20718 | -1,66557 | 1 | 0,05 | 0 | -1,12179 | 0,43163 |
| DHQ, 1 day Control, 14 days | -0,25369 | 0,20718 | -1,22448 | 1 | 0,05 | 0 | -1,0304 | 0,52302 |
| DHQ, 1 day Control, 28 days | 0,29586 | 0,19811 | 1,49344 | 1 | 0,05 | 0 | -0,44682 | 1,03853 |
| DHQ, 1 day Control, 42 days | 0,16898 | 0,22149 | 0,76293 | 1 | 0,05 | 0 | -0,66136 | 0,99932 |
| DHQ, 1 day Control, 56 days | 0,66182 | 0,22149 | 2,98806 | 0,36657 | 0,05 | 0 | -0,16852 | 1,49216 |
| DHQ, 7 days Control, 7 days | 0,00719 | 0,20718 | 0,0347 | 1 | 0,05 | 0 | -0,76952 | 0,7839 |
| | 0,00719 | 0,20718 | 0,0347 | 1 | 0,05 | 0 | -0,76952 | 0,7839 |
| DHQ, 7 days Control, 14 days | 0,09858 | 0,20718 | 0,47579 | 1 | 0,05 | 0 | -0,67813 | 0,87528 |
| DHQ, 7 days Control, 28 days | 0,64813 | 0,19811 | 3,27163 | 0,17759 | 0,05 | 0 | -0,09455 | 1,3908 |
| DHQ, 7 days Control, 42 days | 0,52125 | 0,22149 | 2,35339 | 1 | 0,05 | 0 | -0,30909 | 1,35158 |
| DHQ, 7 days Control, 56 days | 1,01409 | 0,22149 | 4,57852 | 0,00504 | 0,05 | 1 | 0,18375 | 1,84442 |
| DHQ, 7 days DHQ, 1 day | 0,35227 | 0,22149 | 1,59046 | 1 | 0,05 | 0 | -0,47807 | 1,1826 |
| DHQ, 14 days Control, 7 days | 0,27635 | 0,20718 | 1,33383 | 1 | 0,05 | 0 | -0,50036 | 1,05306 |
| | 0,27635 | 0,20718 | 1,33383 | 1 | 0,05 | 0 | -0,50036 | 1,05306 |
| DHQ, 14 days Control, 14 days | 0,36773 | 0,20718 | 1,77491 | 1 | 0,05 | 0 | -0,40898 | 1,14444 |
| DHQ, 14 days Control, 28 days | 0,91728 | 0,19811 | 4,63028 | 0,00436 | 0,05 | 1 | 0,17461 | 1,65996 |
| DHQ, 14 days Control, 42 days | 0,7904 | 0,22149 | 3,56861 | 0,0812 | 0,05 | 0 | -0,03993 | 1,62074 |
| DHQ, 14 days Control, 56 days | 1,28324 | 0,22149 | 5,79374 | 1,63998 | 0,05 | 1 | 0,45291 | 2,11358 |
| DHQ, 14 days DHQ, 1 day | 0,62142 | 0,22149 | 2,80568 | 0,57624 | 0,05 | 0 | -0,20891 | 1,45176 |
| DHQ, 14 days DHQ, 7 days | 0,26916 | 0,22149 | 1,21522 | 1 | 0,05 | 0 | -0,56118 | 1,09949 |
| DHQ, 28 days Control, 7 days | -0,44375 | 0,19181 | -2,31341 | 1 | 0,05 | 0 | -1,16284 | 0,27535 |
| | -0,44375 | 0,19181 | -2,31341 | 1 | 0,05 | 0 | -1,16284 | 0,27535 |
| DHQ, 28 days Control, 14 days | -0,35236 | 0,19181 | -1,83699 | 1 | 0,05 | 0 | -1,07145 | 0,36673 |
| DHQ, 28 days Control, 28 days | 0,19719 | 0,18197 | 1,08363 | 1 | 0,05 | 0 | -0,485 | 0,87938 |
| DHQ, 28 days Control, 42 days | 0,07031 | 0,20718 | 0,33937 | 1 | 0,05 | 0 | -0,7064 | 0,84702 |
| DHQ, 28 days Control, 56 days | 0,56315 | 0,20718 | 2,71813 | 0,71292 | 0,05 | 0 | -0,21356 | 1,33986 |
| DHQ, 28 days DHQ, 1 day | -0,09867 | 0,20718 | -0,47624 | 1 | 0,05 | 0 | -0,87538 | 0,67804 |
| DHQ, 28 days DHQ, 7 days | -0,45094 | 0,20718 | -2,17651 | 1 | 0,05 | 0 | -1,22765 | 0,32577 |
| DHQ, 28 days DHQ, 14 days | -0,72009 | 0,20718 | -3,47563 | 0,10398 | 0,05 | 0 | -1,4968 | 0,05662 |
| DHQ, 42 days Control, 7 days | -0,46654 | 0,20718 | -2,25184 | 1 | 0,05 | 0 | -1,24325 | 0,31017 |
| | -0,46654 | 0,20718 | -2,25184 | 1 | 0,05 | 0 | -1,24325 | 0,31017 |
| DHQ, 42 days Control, 14 days | -0,37516 | 0,20718 | -1,81076 | 1 | 0,05 | 0 | -1,15187 | 0,40155 |
| DHQ, 42 days Control, 28 days | 0,17439 | 0,19811 | 0,8803 | 1 | 0,05 | 0 | -0,56828 | 0,91707 |
| DHQ, 42 days Control, 42 days | 0,04751 | 0,22149 | 0,21452 | 1 | 0,05 | 0 | -0,78282 | 0,87785 |
| DHQ, 42 days Control, 56 days | 0,54035 | 0,22149 | 2,43965 | 1 | 0,05 | 0 | -0,28998 | 1,37069 |
| DHQ, 42 days DHQ, 1 day | -0,12147 | 0,22149 | -0,54841 | 1 | 0,05 | 0 | -0,9518 | 0,70887 |
| DHQ, 42 days DHQ, 7 days | -0,47373 | 0,22149 | -2,13887 | 1 | 0,05 | 0 | -1,30407 | 0,3566 |
| DHQ, 42 days DHQ, 14 days | -0,74289 | 0,22149 | -3,35409 | 0,14322 | 0,05 | 0 | -1,57323 | 0,08745 |
| DHQ, 42 days DHQ, 28 days | -0,0228 | 0,20718 | -0,11004 | 1 | 0,05 | 0 | -0,79951 | 0,75391 |
| DHQ, 56 days Control, 7 days | -1,04254 | 0,20718 | -5,03197 | 0,0014 | 0,05 | 1 | -1,81925 | -0,26583 |
| | -1,04254 | 0,20718 | -5,03197 | 0,0014 | 0,05 | 1 | -1,81925 | -0,26583 |
| DHQ, 56 days Control, 14 days | -0,95115 | 0,20718 | -4,59088 | 0,00487 | 0,05 | 1 | -1,72786 | -0,17444 |
| DHQ, 56 days Control, 28 days | -0,4016 | 0,19811 | -2,02723 | 1 | 0,05 | 0 | -1,14428 | 0,34107 |
| DHQ, 56 days Control, 42 days | -0,52848 | 0,22149 | -2,38605 | 1 | 0,05 | 0 | -1,35882 | 0,30185 |
| DHQ, 56 days Control, 56 days | -0,03564 | 0,22149 | -0,16092 | 1 | 0,05 | 0 | -0,86598 | 0,7947 |
| DHQ, 56 days DHQ, 1 day | -0,69746 | 0,22149 | -3,14898 | 0,24369 | 0,05 | 0 | -1,5278 | 0,13287 |
| DHQ, 56 days DHQ, 7 days | -1,04973 | 0,22149 | -4,73944 | 0,00321 | 0,05 | 1 | -1,88007 | -0,21939 |
| DHQ, 56 days DHQ, 14 days | -1,31889 | 0,22149 | -5,95466 | 1,04463 | 0,05 | 1 | -2,14922 | -0,48855 |
| DHQ, 56 days DHQ, 28 days | -0,59879 | 0,20718 | -2,89017 | 0,46799 | 0,05 | 0 | -1,3755 | 0,17792 |
| DHQ, 56 days DHQ, 42 days | -0,576 | 0,22149 | -2,60057 | 0,94432 | 0,05 | 0 | -1,40633 | 0,25434 |

##### *Scheffe Test*

| | MeanDiff | SEM | F Value | Prob | Alpha | Sig | LCL | UCL |
|---|---|---|---|---|---|---|---|---|
| Control, 7 days Control, 7 days | 0 | 0,19181 | 0 | 1 | 0,05 | 0 | -0,9275 | 0,9275 |
| Control, 14 days Control, 7 days | -0,09139 | 0,19181 | 0,02063 | 1 | 0,05 | 0 | -1,01889 | 0,83611 |
| | -0,09139 | 0,19181 | 0,02063 | 1 | 0,05 | 0 | -1,01889 | 0,83611 |
| Control, 28 days Control, 7 days | -0,64094 | 0,18197 | 1,1278 | 0,37533 | 0,05 | 0 | -1,52084 | 0,23897 |
| | -0,64094 | 0,18197 | 1,1278 | 0,37533 | 0,05 | 0 | -1,52084 | 0,23897 |
| Control, 28 days Control, 14 da | -0,54955 | 0,18197 | 0,82912 | 0,61324 | 0,05 | 0 | -1,42945 | 0,33035 |
| Control, 42 days Control, 7 days | -0,51406 | 0,20718 | 0,55965 | 0,8454 | 0,05 | 0 | -1,51587 | 0,48776 |
| | -0,51406 | 0,20718 | 0,55965 | 0,8454 | 0,05 | 0 | -1,51587 | 0,48776 |
| Control, 42 days Control, 14 da | -0,42267 | 0,20718 | 0,37836 | 0,95459 | 0,05 | 0 | -1,42449 | 0,57914 |
| Control, 42 days Control, 28 da | 0,12688 | 0,19811 | 0,03729 | 1 | 0,05 | 0 | -0,83104 | 1,0848 |
| Control, 56 days Control, 7 days | -1,0069 | 0,20718 | 2,14718 | 0,04779 | 0,05 | 1 | -2,00871 | -0,00508 |
| | -1,0069 | 0,20718 | 2,14718 | 0,04779 | 0,05 | 1 | -2,00871 | -0,00508 |
| Control, 56 days Control, 14 da | -0,91551 | 0,20718 | 1,77511 | 0,10409 | 0,05 | 0 | -1,91733 | 0,0863 |
| Control, 56 days Control, 28 da | -0,36596 | 0,19811 | 0,31023 | 0,97795 | 0,05 | 0 | -1,32388 | 0,59196 |
| Control, 56 days Control, 42 da | -0,49284 | 0,22149 | 0,45011 | 0,91901 | 0,05 | 0 | -1,56383 | 0,57814 |
| DHQ, 1 day Control, 7 days | -0,34508 | 0,20718 | 0,25219 | 0,99025 | 0,05 | 0 | -1,34689 | 0,65674 |
| | -0,34508 | 0,20718 | 0,25219 | 0,99025 | 0,05 | 0 | -1,34689 | 0,65674 |
| DHQ, 1 day Control, 14 days | -0,25369 | 0,20718 | 0,1363 | 0,99935 | 0,05 | 0 | -1,25551 | 0,74812 |
| DHQ, 1 day Control, 28 days | 0,29586 | 0,19811 | 0,20276 | 0,9961 | 0,05 | 0 | -0,66206 | 1,25378 |
| DHQ, 1 day Control, 42 days | 0,16898 | 0,22149 | 0,05291 | 0,99999 | 0,05 | 0 | -0,90201 | 1,23996 |
| DHQ, 1 day Control, 56 days | 0,66182 | 0,22149 | 0,81168 | 0,62874 | 0,05 | 0 | -0,40916 | 1,73281 |
| DHQ, 7 days Control, 7 days | 0,00719 | 0,20718 | 1,09492E-4 | 1 | 0,05 | 0 | -0,99462 | 1,009 |
| | 0,00719 | 0,20718 | 1,09492E-4 | 1 | 0,05 | 0 | -0,99462 | 1,009 |
| DHQ, 7 days Control, 14 days | 0,09858 | 0,20718 | 0,02058 | 1 | 0,05 | 0 | -0,90324 | 1,10039 |
| DHQ, 7 days Control, 28 days | 0,64813 | 0,19811 | 0,97305 | 0,49056 | 0,05 | 0 | -0,30979 | 1,60604 |
| DHQ, 7 days Control, 42 days | 0,52125 | 0,22149 | 0,50349 | 0,88573 | 0,05 | 0 | -0,54974 | 1,59223 |
| DHQ, 7 days Control, 56 days | 1,01409 | 0,22149 | 1,90571 | 0,07926 | 0,05 | 0 | -0,0569 | 2,08507 |
| DHQ, 7 days DHQ, 1 day | 0,35227 | 0,22149 | 0,22996 | 0,99334 | 0,05 | 0 | -0,71872 | 1,42325 |
| DHQ, 14 days Control, 7 days | 0,27635 | 0,20718 | 0,16174 | 0,99857 | 0,05 | 0 | -0,72547 | 1,27816 |
| | 0,27635 | 0,20718 | 0,16174 | 0,99857 | 0,05 | 0 | -0,72547 | 1,27816 |
| DHQ, 14 days Control, 14 days | 0,36773 | 0,20718 | 0,28639 | 0,98379 | 0,05 | 0 | -0,63408 | 1,36955 |
| DHQ, 14 days Control, 28 days | 0,91728 | 0,19811 | 1,94905 | 0,07238 | 0,05 | 0 | -0,04064 | 1,8752 |
| DHQ, 14 days Control, 42 days | 0,7904 | 0,22149 | 1,15772 | 0,35546 | 0,05 | 0 | -0,28058 | 1,86139 |
| DHQ, 14 days Control, 56 days | 1,28324 | 0,22149 | 3,05158 | 0,00747 | 0,05 | 1 | 0,21226 | 2,35423 |
| DHQ, 14 days DHQ, 1 day | 0,62142 | 0,22149 | 0,71562 | 0,71461 | 0,05 | 0 | -0,44956 | 1,69241 |
| DHQ, 14 days DHQ, 7 days | 0,26916 | 0,22149 | 0,13425 | 0,9994 | 0,05 | 0 | -0,80183 | 1,34014 |
| DHQ, 28 days Control, 7 days | -0,44375 | 0,19181 | 0,48653 | 0,89688 | 0,05 | 0 | -1,37125 | 0,48375 |
| | -0,44375 | 0,19181 | 0,48653 | 0,89688 | 0,05 | 0 | -1,37125 | 0,48375 |
| DHQ, 28 days Control, 14 days | -0,35236 | 0,19181 | 0,30677 | 0,97887 | 0,05 | 0 | -1,27986 | 0,57514 |
| DHQ, 28 days Control, 28 days | 0,19719 | 0,18197 | 0,10675 | 0,9998 | 0,05 | 0 | -0,68271 | 1,07709 |
| DHQ, 28 days Control, 42 days | 0,07031 | 0,20718 | 0,01047 | 1 | 0,05 | 0 | -0,9315 | 1,07213 |
| DHQ, 28 days Control, 56 days | 0,56315 | 0,20718 | 0,67166 | 0,7533 | 0,05 | 0 | -0,43866 | 1,56497 |
| DHQ, 28 days DHQ, 1 day | -0,09867 | 0,20718 | 0,02062 | 1 | 0,05 | 0 | -1,10048 | 0,90315 |
| DHQ, 28 days DHQ, 7 days | -0,45094 | 0,20718 | 0,43065 | 0,92974 | 0,05 | 0 | -1,45275 | 0,55088 |
| DHQ, 28 days DHQ, 14 days | -0,72009 | 0,20718 | 1,09819 | 0,39579 | 0,05 | 0 | -1,72191 | 0,28172 |
| DHQ, 42 days Control, 7 days | -0,46654 | 0,20718 | 0,46098 | 0,91268 | 0,05 | 0 | -1,46836 | 0,53527 |
| | -0,46654 | 0,20718 | 0,46098 | 0,91268 | 0,05 | 0 | -1,46836 | 0,53527 |
| DHQ, 42 days Control, 14 days | -0,37516 | 0,20718 | 0,29808 | 0,98108 | 0,05 | 0 | -1,37697 | 0,62666 |
| DHQ, 42 days Control, 28 days | 0,17439 | 0,19811 | 0,07045 | 0,99997 | 0,05 | 0 | -0,78353 | 1,13231 |
| DHQ, 42 days Control, 42 days | 0,04751 | 0,22149 | 0,00418 | 1 | 0,05 | 0 | -1,02347 | 1,1185 |
| DHQ, 42 days Control, 56 days | 0,54035 | 0,22149 | 0,54108 | 0,85927 | 0,05 | 0 | -0,53063 | 1,61134 |
| DHQ, 42 days DHQ, 1 day | -0,12147 | 0,22149 | 0,02734 | 1 | 0,05 | 0 | -1,19245 | 0,94952 |
| DHQ, 42 days DHQ, 7 days | -0,47373 | 0,22149 | 0,41589 | 0,93736 | 0,05 | 0 | -1,54472 | 0,59725 |
| DHQ, 42 days DHQ, 14 days | -0,74289 | 0,22149 | 1,02272 | 0,45139 | 0,05 | 0 | -1,81388 | 0,32809 |
| DHQ, 42 days DHQ, 28 days | -0,0228 | 0,20718 | 0,0011 | 1 | 0,05 | 0 | -1,02461 | 0,97902 |
| DHQ, 56 days Control, 7 days | -1,04254 | 0,20718 | 2,30188 | 0,03458 | 0,05 | 1 | -2,04435 | -0,04072 |
| | -1,04254 | 0,20718 | 2,30188 | 0,03458 | 0,05 | 1 | -2,04435 | -0,04072 |
| DHQ, 56 days Control, 14 days | -0,95115 | 0,20718 | 1,91602 | 0,07757 | 0,05 | 0 | -1,95297 | 0,05066 |
| DHQ, 56 days Control, 28 days | -0,4016 | 0,19811 | 0,37361 | 0,95655 | 0,05 | 0 | -1,35952 | 0,55631 |
| DHQ, 56 days Control, 42 days | -0,52848 | 0,22149 | 0,51757 | 0,87609 | 0,05 | 0 | -1,59947 | 0,5425 |
| DHQ, 56 days Control, 56 days | -0,03564 | 0,22149 | 0,00235 | 1 | 0,05 | 0 | -1,10663 | 1,03534 |
| DHQ, 56 days DHQ, 1 day | -0,69746 | 0,22149 | 0,90146 | 0,55014 | 0,05 | 0 | -1,76845 | 0,37352 |
| DHQ, 56 days DHQ, 7 days | -1,04973 | 0,22149 | 2,04203 | 0,05957 | 0,05 | 0 | -2,12071 | 0,02126 |
| DHQ, 56 days DHQ, 14 days | -1,31889 | 0,22149 | 3,22345 | 0,00532 | 0,05 | 1 | -2,38987 | -0,2479 |
| DHQ, 56 days DHQ, 28 days | -0,59879 | 0,20718 | 0,75937 | 0,67554 | 0,05 | 0 | -1,60061 | 0,40302 |
| DHQ, 56 days DHQ, 42 days | -0,576 | 0,22149 | 0,61482 | 0,80157 | 0,05 | 0 | -1,64698 | 0,49499 |

##### *Tukey Test*

| | MeanDiff | SEM | q Value | Prob | Alpha | Sig | LCL | UCL |
|---|---|---|---|---|---|---|---|---|
| Control, 7 days Control, 7 days | 0 | 0,19181 | 0 | 1 | 0,05 | 0 | -0,67828 | 0,67828 |
| Control, 14 days Control, 7 days | -0,09139 | 0,19181 | 0,67377 | 1 | 0,05 | 0 | -0,76966 | 0,58689 |
| | -0,09139 | 0,19181 | 0,67377 | 1 | 0,05 | 0 | -0,76966 | 0,58689 |
| Control, 28 days Control, 7 days | -0,64094 | 0,18197 | 4,98112 | 0,05163 | 0,05 | 0 | -1,2844 | 0,00253 |
| | -0,64094 | 0,18197 | 4,98112 | 0,05163 | 0,05 | 0 | -1,2844 | 0,00253 |
| Control, 28 days Control, 14 da | -0,54955 | 0,18197 | 4,2709 | 0,15179 | 0,05 | 0 | -1,19302 | 0,09392 |
| Control, 42 days Control, 7 days | -0,51406 | 0,20718 | 3,5089 | 0,38791 | 0,05 | 0 | -1,24668 | 0,21856 |
| | -0,51406 | 0,20718 | 3,5089 | 0,38791 | 0,05 | 0 | -1,24668 | 0,21856 |
| Control, 42 days Control, 14 da | -0,42267 | 0,20718 | 2,88512 | 0,66478 | 0,05 | 0 | -1,15529 | 0,30995 |
| Control, 42 days Control, 28 da | 0,12688 | 0,19811 | 0,90575 | 0,99994 | 0,05 | 0 | -0,57364 | 0,8274 |
| Control, 56 days Control, 7 days | -1,0069 | 0,20718 | 6,87299 | 0,00173 | 0,05 | 1 | -1,73952 | -0,27428 |
| | -1,0069 | 0,20718 | 6,87299 | 0,00173 | 0,05 | 1 | -1,73952 | -0,27428 |
| Control, 56 days Control, 14 da | -0,91551 | 0,20718 | 6,2492 | 0,00559 | 0,05 | 1 | -1,64813 | -0,18289 |
| Control, 56 days Control, 28 da | -0,36596 | 0,19811 | 2,6125 | 0,78012 | 0,05 | 0 | -1,06648 | 0,33456 |
| Control, 56 days Control, 42 da | -0,49284 | 0,22149 | 3,14681 | 0,5456 | 0,05 | 0 | -1,27605 | 0,29036 |
| DHQ, 1 day Control, 7 days | -0,34508 | 0,20718 | 2,35547 | 0,86963 | 0,05 | 0 | -1,0777 | 0,38754 |
| | -0,34508 | 0,20718 | 2,35547 | 0,86963 | 0,05 | 0 | -1,0777 | 0,38754 |
| DHQ, 1 day Control, 14 days | -0,25369 | 0,20718 | 1,73168 | 0,98241 | 0,05 | 0 | -0,98631 | 0,47893 |
| DHQ, 1 day Control, 28 days | 0,29586 | 0,19811 | 2,11204 | 0,93107 | 0,05 | 0 | -0,40466 | 0,99638 |
| DHQ, 1 day Control, 42 days | 0,16898 | 0,22149 | 1,07894 | 0,9997 | 0,05 | 0 | -0,61423 | 0,95219 |
| DHQ, 1 day Control, 56 days | 0,66182 | 0,22149 | 4,22576 | 0,16162 | 0,05 | 0 | -0,12139 | 1,44503 |
| DHQ, 7 days Control, 7 days | 0,00719 | 0,20718 | 0,04908 | 1 | 0,05 | 0 | -0,72543 | 0,73981 |
| | 0,00719 | 0,20718 | 0,04908 | 1 | 0,05 | 0 | -0,72543 | 0,73981 |
| DHQ, 7 days Control, 14 days | 0,09858 | 0,20718 | 0,67287 | 1 | 0,05 | 0 | -0,63405 | 0,8312 |
| DHQ, 7 days Control, 28 days | 0,64813 | 0,19811 | 4,62678 | 0,09022 | 0,05 | 0 | -0,05239 | 1,34865 |
| DHQ, 7 days Control, 42 days | 0,52125 | 0,22149 | 3,32819 | 0,46428 | 0,05 | 0 | -0,26196 | 1,30445 |
| DHQ, 7 days Control, 56 days | 1,01409 | 0,22149 | 6,475 | 0,00368 | 0,05 | 1 | 0,23088 | 1,79729 |
| DHQ, 7 days DHQ, 1 day | 0,35227 | 0,22149 | 2,24925 | 0,89938 | 0,05 | 0 | -0,43094 | 1,13547 |
| DHQ, 14 days Control, 7 days | 0,27635 | 0,20718 | 1,88632 | 0,96734 | 0,05 | 0 | -0,45627 | 1,00897 |
| | 0,27635 | 0,20718 | 1,88632 | 0,96734 | 0,05 | 0 | -0,45627 | 1,00897 |
| DHQ, 14 days Control, 14 days | 0,36773 | 0,20718 | 2,51011 | 0,81852 | 0,05 | 0 | -0,36489 | 1,10035 |
| DHQ, 14 days Control, 28 days | 0,91728 | 0,19811 | 6,54821 | 0,0032 | 0,05 | 1 | 0,21676 | 1,6178 |
| DHQ, 14 days Control, 42 days | 0,7904 | 0,22149 | 5,04677 | 0,04638 | 0,05 | 1 | 0,0072 | 1,57361 |
| DHQ, 14 days Control, 56 days | 1,28324 | 0,22149 | 8,19358 | 1,36426 | 0,05 | 1 | 0,50004 | 2,06645 |
| DHQ, 14 days DHQ, 1 day | 0,62142 | 0,22149 | 3,96783 | 0,22759 | 0,05 | 0 | -0,16178 | 1,40463 |
| DHQ, 14 days DHQ, 7 days | 0,26916 | 0,22149 | 1,71858 | 0,98338 | 0,05 | 0 | -0,51405 | 1,05236 |
| DHQ, 28 days Control, 7 days | -0,44375 | 0,19181 | 3,27166 | 0,48925 | 0,05 | 0 | -1,12202 | 0,23453 |
| | -0,44375 | 0,19181 | 3,27166 | 0,48925 | 0,05 | 0 | -1,12202 | 0,23453 |
| DHQ, 28 days Control, 14 days | -0,35236 | 0,19181 | 2,59789 | 0,7858 | 0,05 | 0 | -1,03064 | 0,32592 |
| DHQ, 28 days Control, 28 days | 0,19719 | 0,18197 | 1,53248 | 0,99322 | 0,05 | 0 | -0,44628 | 0,84066 |
| DHQ, 28 days Control, 42 days | 0,07031 | 0,20718 | 0,47994 | 1 | 0,05 | 0 | -0,66231 | 0,80293 |
| DHQ, 28 days Control, 56 days | 0,56315 | 0,20718 | 3,84402 | 0,26548 | 0,05 | 0 | -0,16947 | 1,29577 |
| DHQ, 28 days DHQ, 1 day | -0,09867 | 0,20718 | 0,6735 | 1 | 0,05 | 0 | -0,83129 | 0,63395 |
| DHQ, 28 days DHQ, 7 days | -0,45094 | 0,20718 | 3,07805 | 0,57704 | 0,05 | 0 | -1,18356 | 0,28169 |
| DHQ, 28 days DHQ, 14 days | -0,72009 | 0,20718 | 4,91529 | 0,05742 | 0,05 | 0 | -1,45271 | 0,01253 |
| DHQ, 42 days Control, 7 days | -0,46654 | 0,20718 | 3,18459 | 0,52842 | 0,05 | 0 | -1,19917 | 0,26608 |
| | -0,46654 | 0,20718 | 3,18459 | 0,52842 | 0,05 | 0 | -1,19917 | 0,26608 |
| DHQ, 42 days Control, 14 days | -0,37516 | 0,20718 | 2,5608 | 0,79993 | 0,05 | 0 | -1,10778 | 0,35746 |
| DHQ, 42 days Control, 28 days | 0,17439 | 0,19811 | 1,24493 | 0,99886 | 0,05 | 0 | -0,52613 | 0,87491 |
| DHQ, 42 days Control, 42 days | 0,04751 | 0,22149 | 0,30337 | 1 | 0,05 | 0 | -0,73569 | 0,83072 |
| DHQ, 42 days Control, 56 days | 0,54035 | 0,22149 | 3,45019 | 0,41206 | 0,05 | 0 | -0,24285 | 1,32356 |
| DHQ, 42 days DHQ, 1 day | -0,12147 | 0,22149 | 0,77557 | 0,99999 | 0,05 | 0 | -0,90467 | 0,66174 |
| DHQ, 42 days DHQ, 7 days | -0,47373 | 0,22149 | 3,02482 | 0,60142 | 0,05 | 0 | -1,25694 | 0,30947 |
| DHQ, 42 days DHQ, 14 days | -0,74289 | 0,22149 | 4,7434 | 0,07538 | 0,05 | 0 | -1,5261 | 0,04031 |
| DHQ, 42 days DHQ, 28 days | -0,0228 | 0,20718 | 0,15562 | 1 | 0,05 | 0 | -0,75542 | 0,70982 |
| DHQ, 56 days Control, 7 days | -1,04254 | 0,20718 | 7,11628 | 0,00109 | 0,05 | 1 | -1,77516 | -0,30992 |
| | -1,04254 | 0,20718 | 7,11628 | 0,00109 | 0,05 | 1 | -1,77516 | -0,30992 |
| DHQ, 56 days Control, 14 days | -0,95115 | 0,20718 | 6,49249 | 0,00356 | 0,05 | 1 | -1,68378 | -0,21853 |
| DHQ, 56 days Control, 28 days | -0,4016 | 0,19811 | 2,86693 | 0,6729 | 0,05 | 0 | -1,10212 | 0,29892 |
| DHQ, 56 days Control, 42 days | -0,52848 | 0,22149 | 3,37439 | 0,44421 | 0,05 | 0 | -1,31169 | 0,25472 |
| DHQ, 56 days Control, 56 days | -0,03564 | 0,22149 | 0,22758 | 1 | 0,05 | 0 | -0,81885 | 0,74756 |
| DHQ, 56 days DHQ, 1 day | -0,69746 | 0,22149 | 4,45333 | 0,1169 | 0,05 | 0 | -1,48067 | 0,08574 |
| DHQ, 56 days DHQ, 7 days | -1,04973 | 0,22149 | 6,70258 | 0,0024 | 0,05 | 1 | -1,83294 | -0,26652 |
| DHQ, 56 days DHQ, 14 days | -1,31889 | 0,22149 | 8,42116 | 8,78243 | 0,05 | 1 | -2,10209 | -0,53568 |
| DHQ, 56 days DHQ, 28 days | -0,59879 | 0,20718 | 4,08731 | 0,19489 | 0,05 | 0 | -1,33142 | 0,13383 |
| DHQ, 56 days DHQ, 42 days | -0,576 | 0,22149 | 3,67776 | 0,32273 | 0,05 | 0 | -1,3592 | 0,20721 |

#### *Grouping Letters*

##### *Bonferroni Test*

| | Mean | Groups | | |
|---|---|---|---|---|
| DHQ, 14 days | 1,61333 | A | | |
| DHQ, 7 days | 1,34417 | A | B | |
| Control, 7 days | 1,33698 | A | B | |
| | 1,33698 | A | B | |
| Control, 14 days | 1,2456 | A | B | |
| DHQ, 1 day | 0,99191 | A | B | C |
| DHQ, 28 days | 0,89324 | A | B | C |
| DHQ, 42 days | 0,87044 | A | B | C |
| Control, 42 days | 0,82293 | A | B | C |
| Control, 28 days | 0,69605 | | B | C |
| Control, 56 days | 0,33009 | | | C |
| DHQ, 56 days | 0,29444 | | | C |

Means that do not share a letter are significantly different.

##### *Scheffe Test*

| | Mean | Groups | |
|---|---|---|---|
| DHQ, 14 days | 1,61333 | A | |
| DHQ, 7 days | 1,34417 | A | B |
| Control, 7 days | 1,33698 | A | |
| | 1,33698 | A | |
| Control, 14 days | 1,2456 | A | B |
| DHQ, 1 day | 0,99191 | A | B |
| DHQ, 28 days | 0,89324 | A | B |
| DHQ, 42 days | 0,87044 | A | B |
| Control, 42 days | 0,82293 | A | B |
| Control, 28 days | 0,69605 | A | B |
| Control, 56 days | 0,33009 | | B |
| DHQ, 56 days | 0,29444 | | B |

Means that do not share a letter are significantly different.

##### *Tukey Test*

| | Mean | Groups | | |
|---|---|---|---|---|
| DHQ, 14 days | 1,61333 | A | | |
| DHQ, 7 days | 1,34417 | A | B | |
| Control, 7 days | 1,33698 | A | B | |
| | 1,33698 | A | B | |
| Control, 14 days | 1,2456 | A | B | |
| DHQ, 1 day | 0,99191 | A | B | C |
| DHQ, 28 days | 0,89324 | A | B | C |
| DHQ, 42 days | 0,87044 | A | B | C |
| Control, 42 days | 0,82293 | | B | C |
| Control, 28 days | 0,69605 | | B | C |
| Control, 56 days | 0,33009 | | | C |
| DHQ, 56 days | 0,29444 | | | C |

Means that do not share a letter are significantly different.

Sig equals 1 indicates that the difference of the means is significant at the 0,05 level.
Sig equals 0 indicates that the difference of the means is not significant at the 0,05 level.

### *Powers*

| | Alpha | Sample Size | Power |
|---|---|---|---|
| Actual Power | 0,05 | 42 | 0,99999 |

# ANOVAOneWay (28.10.2024 12:41:22)

## Notes

| | |
|---|---|
| Description | Perform One-Way ANOVA |
| User Name | yulia |
| Operation Time | 28.10.2024 12:41:22 |
| Report Status | New Analysis Report |
| Data Filter | No |

## Input Data

| | Data | Range |
|---|---|---|
| Control, 1 day | [Book1]"MitoTracker Green"!A"Control, 1 day" | [1*:4*] |
| Control, 7 days | [Book1]"MitoTracker Green"!B"Control, 7 days" | [1*:4*] |
| Control, 14 days | [Book1]"MitoTracker Green"!C"Control, 14 days" | [1*:4*] |
| Control, 28 days | [Book1]"MitoTracker Green"!D"Control, 28 days" | [1*:5*] |
| Control, 42 days | [Book1]"MitoTracker Green"!E"Control, 42 days" | [1*:3*] |
| Control, 56 days | [Book1]"MitoTracker Green"!F"Control, 56 days" | [1*:3*] |
| DHQ, 1 day | [Book1]"MitoTracker Green"!G"DHQ, 1 day" | [1*:3*] |
| DHQ, 7 days | [Book1]"MitoTracker Green"!H"DHQ, 7 days" | [1*:3*] |
| DHQ, 14 days | [Book1]"MitoTracker Green"!I"DHQ, 14 days" | [1*:3*] |
| DHQ, 28 days | [Book1]"MitoTracker Green"!J"DHQ, 28 days" | [1*:4*] |
| DHQ, 42 days | [Book1]"MitoTracker Green"!K"DHQ, 42 days" | [1*:3*] |
| DHQ, 56 days | [Book1]"MitoTracker Green"!L"DHQ, 56 days" | [1*:3*] |

## Descriptive Statistics

| | N Analysis | N Missing | Mean | Standard Deviation | SE of Mean |
|---|---|---|---|---|---|
| Control, 1 day | 4 | 0 | 1 | 0 | 0 |
| Control, 7 days | 4 | 0 | 0,75284 | 0,15367 | 0,07684 |
| Control, 14 days | 4 | 0 | 0,80575 | 0,32102 | 0,16051 |
| Control, 28 days | 5 | 0 | 0,62457 | 0,25903 | 0,11584 |
| Control, 42 days | 3 | 0 | 0,75046 | 0,33497 | 0,1934 |
| Control, 56 days | 3 | 0 | 0,85375 | 0,23072 | 0,1332 |
| DHQ, 1 day | 3 | 0 | 0,62545 | 0,27128 | 0,15662 |
| DHQ, 7 days | 3 | 0 | 0,43196 | 0,30081 | 0,17367 |
| DHQ, 14 days | 3 | 0 | 0,40671 | 0,17417 | 0,10056 |
| DHQ, 28 days | 4 | 0 | 0,4242 | 0,2321 | 0,11605 |
| DHQ, 42 days | 3 | 0 | 0,24098 | 0,14279 | 0,08244 |
| DHQ, 56 days | 3 | 0 | 0,20314 | 0,06826 | 0,03941 |

## ANOVA

### Overall ANOVA

| | DF | Sum of Squares | Mean Square | F Value | Prob>F |
|---|---|---|---|---|---|
| Model | 11 | 2,34477 | 0,21316 | 4,04781 | 0,00112 |
| Error | 30 | 1,57983 | 0,05266 | | |
| Total | 41 | 3,9246 | | | |

Null Hypothesis: The means of all levels are equal.
Alternative Hypothesis: The means of one or more levels are different.
At the 0.05 level, the population means are significantly different.

### Fit Statistics

| | R-Square | Coeff Var | Root MSE | Data Mean |
|---|---|---|---|---|
| | 0,59746 | 0,37662 | 0,22948 | 0,60932 |

### Means Comparisons

#### Bonferroni Test

| | MeanDiff | SEM | t Value | Prob | Alpha | Sig | LCL | UCL |
|---|---|---|---|---|---|---|---|---|
| Control, 7 days Control, 1 day | -0,24716 | 0,16227 | -1,5232 | 1 | 0,05 | 0 | -0,85549 | 0,36116 |
| Control, 14 days Control, 1 day | -0,19425 | 0,16227 | -1,1971 | 1 | 0,05 | 0 | -0,80257 | 0,41407 |
| Control, 14 days Control, 7 days | 0,05291 | 0,16227 | 0,3261 | 1 | 0,05 | 0 | -0,55541 | 0,66124 |
| Control, 28 days Control, 1 day | -0,37543 | 0,15394 | -2,43883 | 1 | 0,05 | 0 | -0,95254 | 0,20167 |
| Control, 28 days Control, 7 days | -0,12827 | 0,15394 | -0,83323 | 1 | 0,05 | 0 | -0,70537 | 0,44884 |
| Control, 28 days Control, 14 da | -0,18118 | 0,15394 | -1,17697 | 1 | 0,05 | 0 | -0,75829 | 0,39592 |
| Control, 42 days Control, 1 day | -0,24954 | 0,17527 | -1,42377 | 1 | 0,05 | 0 | -0,9066 | 0,40752 |
| Control, 42 days Control, 7 days | -0,00238 | 0,17527 | -0,01355 | 1 | 0,05 | 0 | -0,65944 | 0,65469 |
| Control, 42 days Control, 14 da | -0,05529 | 0,17527 | -0,31546 | 1 | 0,05 | 0 | -0,71235 | 0,60177 |
| Control, 42 days Control, 28 da | 0,12589 | 0,16759 | 0,7512 | 1 | 0,05 | 0 | -0,50238 | 0,75416 |
| Control, 56 days Control, 1 day | -0,14625 | 0,17527 | -0,83446 | 1 | 0,05 | 0 | -0,80332 | 0,51081 |
| Control, 56 days Control, 7 days | 0,10091 | 0,17527 | 0,57575 | 1 | 0,05 | 0 | -0,55615 | 0,75797 |
| Control, 56 days Control, 14 da | 0,048 | 0,17527 | 0,27384 | 1 | 0,05 | 0 | -0,60907 | 0,70506 |
| Control, 56 days Control, 28 da | 0,22918 | 0,16759 | 1,36751 | 1 | 0,05 | 0 | -0,39909 | 0,85745 |
| Control, 56 days Control, 42 da | 0,10329 | 0,18737 | 0,55125 | 1 | 0,05 | 0 | -0,59914 | 0,80572 |
| DHQ, 1 day Control, 1 day | -0,37455 | 0,17527 | -2,13702 | 1 | 0,05 | 0 | -1,03161 | 0,28251 |
| DHQ, 1 day Control, 7 days | -0,12739 | 0,17527 | -0,72681 | 1 | 0,05 | 0 | -0,78445 | 0,52968 |
| DHQ, 1 day Control, 14 days | -0,1803 | 0,17527 | -1,02871 | 1 | 0,05 | 0 | -0,83736 | 0,47676 |
| DHQ, 1 day Control, 28 days | 8,81778E-4 | 0,16759 | 0,00526 | 1 | 0,05 | 0 | -0,62739 | 0,62915 |
| DHQ, 1 day Control, 42 days | -0,12501 | 0,18737 | -0,66719 | 1 | 0,05 | 0 | -0,82744 | 0,57742 |
| DHQ, 1 day Control, 56 days | -0,2283 | 0,18737 | -1,21843 | 1 | 0,05 | 0 | -0,93073 | 0,47413 |
| DHQ, 7 days Control, 1 day | -0,56804 | 0,17527 | -3,241 | 0,19227 | 0,05 | 0 | -1,22511 | 0,08902 |
| DHQ, 7 days Control, 7 days | -0,32088 | 0,17527 | -1,83079 | 1 | 0,05 | 0 | -0,97794 | 0,33618 |
| DHQ, 7 days Control, 14 days | -0,37379 | 0,17527 | -2,1327 | 1 | 0,05 | 0 | -1,03086 | 0,28327 |
| DHQ, 7 days Control, 28 days | -0,19261 | 0,16759 | -1,14931 | 1 | 0,05 | 0 | -0,82088 | 0,43566 |
| DHQ, 7 days Control, 42 days | -0,3185 | 0,18737 | -1,69987 | 1 | 0,05 | 0 | -1,02093 | 0,38393 |
| DHQ, 7 days Control, 56 days | -0,42179 | 0,18737 | -2,25111 | 1 | 0,05 | 0 | -1,12422 | 0,28064 |
| DHQ, 7 days DHQ, 1 day | -0,19349 | 0,18737 | -1,03268 | 1 | 0,05 | 0 | -0,89592 | 0,50894 |
| DHQ, 14 days Control, 1 day | -0,59329 | 0,17527 | -3,38507 | 0,13204 | 0,05 | 0 | -1,25036 | 0,06377 |
| DHQ, 14 days Control, 7 days | -0,34613 | 0,17527 | -1,97486 | 1 | 0,05 | 0 | -1,00319 | 0,31093 |
| DHQ, 14 days Control, 14 days | -0,39904 | 0,17527 | -2,27677 | 1 | 0,05 | 0 | -1,05611 | 0,25802 |
| DHQ, 14 days Control, 28 days | -0,21786 | 0,16759 | -1,29998 | 1 | 0,05 | 0 | -0,84613 | 0,41041 |
| DHQ, 14 days Control, 42 days | -0,34375 | 0,18737 | -1,83463 | 1 | 0,05 | 0 | -1,04618 | 0,35868 |
| DHQ, 14 days Control, 56 days | -0,44704 | 0,18737 | -2,38588 | 1 | 0,05 | 0 | -1,14947 | 0,25539 |
| DHQ, 14 days DHQ, 1 day | -0,21874 | 0,18737 | -1,16745 | 1 | 0,05 | 0 | -0,92117 | 0,48369 |
| DHQ, 14 days DHQ, 7 days | -0,02525 | 0,18737 | -0,13477 | 1 | 0,05 | 0 | -0,72768 | 0,67718 |
| DHQ, 28 days Control, 1 day | -0,5758 | 0,16227 | -3,54849 | 0,08567 | 0,05 | 0 | -1,18412 | 0,03252 |
| DHQ, 28 days Control, 7 days | -0,32864 | 0,16227 | -2,02529 | 1 | 0,05 | 0 | -0,93696 | 0,27968 |
| DHQ, 28 days Control, 14 days | -0,38155 | 0,16227 | -2,35139 | 1 | 0,05 | 0 | -0,98987 | 0,22677 |
| DHQ, 28 days Control, 28 days | -0,20037 | 0,15394 | -1,30161 | 1 | 0,05 | 0 | -0,77747 | 0,37674 |
| DHQ, 28 days Control, 42 days | -0,32626 | 0,17527 | -1,8615 | 1 | 0,05 | 0 | -0,98332 | 0,3308 |
| DHQ, 28 days Control, 56 days | -0,42955 | 0,17527 | -2,45081 | 1 | 0,05 | 0 | -1,08661 | 0,22751 |
| DHQ, 28 days DHQ, 1 day | -0,20125 | 0,17527 | -1,14825 | 1 | 0,05 | 0 | -0,85831 | 0,45581 |
| DHQ, 28 days DHQ, 7 days | -0,00776 | 0,17527 | -0,04427 | 1 | 0,05 | 0 | -0,66482 | 0,6493 |
| DHQ, 28 days DHQ, 14 days | 0,01749 | 0,17527 | 0,09981 | 1 | 0,05 | 0 | -0,63957 | 0,67456 |
| DHQ, 42 days Control, 1 day | -0,75902 | 0,17527 | -4,33065 | 0,01009 | 0,05 | 1 | -1,41609 | -0,1019 |
| DHQ, 42 days Control, 7 days | -0,51186 | 0,17527 | -2,92044 | 0,43409 | 0,05 | 0 | -1,16892 | 0,1452 |
| DHQ, 42 days Control, 14 days | -0,56477 | 0,17527 | -3,22235 | 0,20177 | 0,05 | 0 | -1,22184 | 0,09229 |
| DHQ, 42 days Control, 28 days | -0,38359 | 0,16759 | -2,2889 | 1 | 0,05 | 0 | -1,01186 | 0,24468 |
| DHQ, 42 days Control, 42 days | -0,50948 | 0,18737 | -2,71914 | 0,71118 | 0,05 | 0 | -1,21191 | 0,19294 |
| DHQ, 42 days Control, 56 days | -0,61277 | 0,18737 | -3,27039 | 0,17816 | 0,05 | 0 | -1,3152 | 0,08966 |
| DHQ, 42 days DHQ, 1 day | -0,38447 | 0,18737 | -2,05196 | 1 | 0,05 | 0 | -1,0869 | 0,31796 |
| DHQ, 42 days DHQ, 7 days | -0,19098 | 0,18737 | -1,01928 | 1 | 0,05 | 0 | -0,89341 | 0,51145 |
| DHQ, 42 days DHQ, 14 days | -0,16573 | 0,18737 | -0,88451 | 1 | 0,05 | 0 | -0,86816 | 0,5367 |
| DHQ, 42 days DHQ, 28 days | -0,18322 | 0,17527 | -1,04539 | 1 | 0,05 | 0 | -0,84029 | 0,47384 |
| DHQ, 56 days Control, 1 day | -0,79686 | 0,17527 | -4,54652 | 0,00551 | 0,05 | 1 | -1,45392 | -0,1398 |
| DHQ, 56 days Control, 7 days | -0,54969 | 0,17527 | -3,13631 | 0,25173 | 0,05 | 0 | -1,20676 | 0,10737 |
| DHQ, 56 days Control, 14 days | -0,60261 | 0,17527 | -3,43821 | 0,11479 | 0,05 | 0 | -1,25967 | 0,05445 |
| DHQ, 56 days Control, 28 days | -0,42143 | 0,16759 | -2,51465 | 1 | 0,05 | 0 | -1,0497 | 0,20685 |
| DHQ, 56 days Control, 42 days | -0,54732 | 0,18737 | -2,92107 | 0,43342 | 0,05 | 0 | -1,24975 | 0,15511 |
| DHQ, 56 days Control, 56 days | -0,6506 | 0,18737 | -3,47231 | 0,10489 | 0,05 | 0 | -1,35303 | 0,05182 |
| DHQ, 56 days DHQ, 1 day | -0,42231 | 0,18737 | -2,25388 | 1 | 0,05 | 0 | -1,12474 | 0,28012 |
| DHQ, 56 days DHQ, 7 days | -0,22882 | 0,18737 | -1,2212 | 1 | 0,05 | 0 | -0,93124 | 0,47361 |
| DHQ, 56 days DHQ, 14 days | -0,20356 | 0,18737 | -1,08643 | 1 | 0,05 | 0 | -0,90599 | 0,49886 |
| DHQ, 56 days DHQ, 28 days | -0,22106 | 0,17527 | -1,26125 | 1 | 0,05 | 0 | -0,87812 | 0,43601 |
| DHQ, 56 days DHQ, 42 days | -0,03783 | 0,18737 | -0,20192 | 1 | 0,05 | 0 | -0,74026 | 0,6646 |

#### Scheffe Test

| | MeanDiff | SEM | F Value | Prob | Alpha | Sig | LCL | UCL |
|---|---|---|---|---|---|---|---|---|
| Control, 7 days Control, 1 day | -0,24716 | 0,16227 | 0,21092 | 0,99538 | 0,05 | 0 | -1,03179 | 0,53746 |
| Control, 14 days Control, 1 day | -0,19425 | 0,16227 | 0,13028 | 0,99948 | 0,05 | 0 | -0,97887 | 0,59037 |
| Control, 14 days Control, 7 days | 0,05291 | 0,16227 | 0,00967 | 1 | 0,05 | 0 | -0,73171 | 0,83754 |
| Control, 28 days Control, 1 day | -0,37543 | 0,15394 | 0,54072 | 0,85954 | 0,05 | 0 | -1,11979 | 0,36893 |
| Control, 28 days Control, 7 days | -0,12827 | 0,15394 | 0,06312 | 0,99999 | 0,05 | 0 | -0,87263 | 0,61609 |
| Control, 28 days Control, 14 da | -0,18118 | 0,15394 | 0,12593 | 0,99955 | 0,05 | 0 | -0,92554 | 0,56318 |
| Control, 42 days Control, 1 day | -0,24954 | 0,17527 | 0,18428 | 0,99743 | 0,05 | 0 | -1,09703 | 0,59795 |
| Control, 42 days Control, 7 days | -0,00238 | 0,17527 | 1,67024E-5 | 1 | 0,05 | 0 | -0,84987 | 0,84512 |
| Control, 42 days Control, 14 da | -0,05529 | 0,17527 | 0,00905 | 1 | 0,05 | 0 | -0,90278 | 0,7922 |
| Control, 42 days Control, 28 da | 0,12589 | 0,16759 | 0,0513 | 0,99999 | 0,05 | 0 | -0,68446 | 0,93625 |
| Control, 56 days Control, 1 day | -0,14625 | 0,17527 | 0,0633 | 0,99999 | 0,05 | 0 | -0,99375 | 0,70124 |
| Control, 56 days Control, 7 days | 0,10091 | 0,17527 | 0,03014 | 1 | 0,05 | 0 | -0,74658 | 0,9484 |
| Control, 56 days Control, 14 da | 0,048 | 0,17527 | 0,00682 | 1 | 0,05 | 0 | -0,7995 | 0,89549 |
| Control, 56 days Control, 28 da | 0,22918 | 0,16759 | 0,17001 | 0,99821 | 0,05 | 0 | -0,58118 | 1,03954 |
| Control, 56 days Control, 42 da | 0,10329 | 0,18737 | 0,02762 | 1 | 0,05 | 0 | -0,80272 | 1,00929 |
| DHQ, 1 day Control, 1 day | -0,37455 | 0,17527 | 0,41517 | 0,93771 | 0,05 | 0 | -1,22204 | 0,47294 |
| DHQ, 1 day Control, 7 days | -0,12739 | 0,17527 | 0,04802 | 1 | 0,05 | 0 | -0,97488 | 0,72011 |
| DHQ, 1 day Control, 14 days | -0,1803 | 0,17527 | 0,0962 | 0,99988 | 0,05 | 0 | -1,02779 | 0,66719 |
| DHQ, 1 day Control, 28 days | 8,81778E-4 | 0,16759 | 2,51674E-6 | 1 | 0,05 | 0 | -0,80948 | 0,81124 |
| DHQ, 1 day Control, 42 days | -0,12501 | 0,18737 | 0,04047 | 1 | 0,05 | 0 | -1,03102 | 0,781 |
| DHQ, 1 day Control, 56 days | -0,2283 | 0,18737 | 0,13496 | 0,99938 | 0,05 | 0 | -1,1343 | 0,67771 |
| DHQ, 7 days Control, 1 day | -0,56804 | 0,17527 | 0,95492 | 0,50533 | 0,05 | 0 | -1,41553 | 0,27945 |
| DHQ, 7 days Control, 7 days | -0,32088 | 0,17527 | 0,30471 | 0,97941 | 0,05 | 0 | -1,16837 | 0,52661 |
| DHQ, 7 days Control, 14 days | -0,37379 | 0,17527 | 0,41349 | 0,93855 | 0,05 | 0 | -1,22128 | 0,4737 |
| DHQ, 7 days Control, 28 days | -0,19261 | 0,16759 | 0,12008 | 0,99964 | 0,05 | 0 | -1,00297 | 0,61775 |
| DHQ, 7 days Control, 42 days | -0,3185 | 0,18737 | 0,26269 | 0,98849 | 0,05 | 0 | -1,22451 | 0,5875 |
| DHQ, 7 days Control, 56 days | -0,42179 | 0,18737 | 0,46068 | 0,91285 | 0,05 | 0 | -1,3278 | 0,48422 |
| DHQ, 7 days DHQ, 1 day | -0,19349 | 0,18737 | 0,09695 | 0,99987 | 0,05 | 0 | -1,0995 | 0,71251 |
| DHQ, 14 days Control, 1 day | -0,59329 | 0,17527 | 1,0417 | 0,43695 | 0,05 | 0 | -1,44079 | 0,2542 |
| DHQ, 14 days Control, 7 days | -0,34613 | 0,17527 | 0,35455 | 0,96391 | 0,05 | 0 | -1,19362 | 0,50136 |
| DHQ, 14 days Control, 14 days | -0,39904 | 0,17527 | 0,47124 | 0,90648 | 0,05 | 0 | -1,24654 | 0,44845 |
| DHQ, 14 days Control, 28 days | -0,21786 | 0,16759 | 0,15363 | 0,99887 | 0,05 | 0 | -1,02822 | 0,5925 |
| DHQ, 14 days Control, 42 days | -0,34375 | 0,18737 | 0,30599 | 0,97908 | 0,05 | 0 | -1,24976 | 0,56225 |
| DHQ, 14 days Control, 56 days | -0,44704 | 0,18737 | 0,51749 | 0,87615 | 0,05 | 0 | -1,35305 | 0,45897 |
| DHQ, 14 days DHQ, 1 day | -0,21874 | 0,18737 | 0,1239 | 0,99959 | 0,05 | 0 | -1,12475 | 0,68726 |
| DHQ, 14 days DHQ, 7 days | -0,02525 | 0,18737 | 0,00165 | 1 | 0,05 | 0 | -0,93126 | 0,88076 |
| DHQ, 28 days Control, 1 day | -0,5758 | 0,16227 | 1,14471 | 0,364 | 0,05 | 0 | -1,36043 | 0,20882 |
| DHQ, 28 days Control, 7 days | -0,32864 | 0,16227 | 0,37289 | 0,95684 | 0,05 | 0 | -1,11326 | 0,45599 |
| DHQ, 28 days Control, 14 days | -0,38155 | 0,16227 | 0,50264 | 0,88631 | 0,05 | 0 | -1,16618 | 0,40307 |
| DHQ, 28 days Control, 28 days | -0,20037 | 0,15394 | 0,15402 | 0,99886 | 0,05 | 0 | -0,94473 | 0,54399 |
| DHQ, 28 days Control, 42 days | -0,32626 | 0,17527 | 0,31502 | 0,97664 | 0,05 | 0 | -1,17375 | 0,52123 |
| DHQ, 28 days Control, 56 days | -0,42955 | 0,17527 | 0,54604 | 0,85562 | 0,05 | 0 | -1,27704 | 0,41794 |
| DHQ, 28 days DHQ, 1 day | -0,20125 | 0,17527 | 0,11986 | 0,99965 | 0,05 | 0 | -1,04874 | 0,64624 |
| DHQ, 28 days DHQ, 7 days | -0,00776 | 0,17527 | 1,78137E-4 | 1 | 0,05 | 0 | -0,85525 | 0,83973 |
| DHQ, 28 days DHQ, 14 days | 0,01749 | 0,17527 | 9,05555E-4 | 1 | 0,05 | 0 | -0,83 | 0,86498 |
| DHQ, 42 days Control, 1 day | -0,75902 | 0,17527 | 1,70496 | 0,1204 | 0,05 | 0 | -1,60652 | 0,08847 |
| DHQ, 42 days Control, 7 days | -0,51186 | 0,17527 | 0,77536 | 0,66121 | 0,05 | 0 | -1,35935 | 0,33563 |
| DHQ, 42 days Control, 14 days | -0,56477 | 0,17527 | 0,94396 | 0,51437 | 0,05 | 0 | -1,41227 | 0,28272 |
| DHQ, 42 days Control, 28 days | -0,38359 | 0,16759 | 0,47628 | 0,90337 | 0,05 | 0 | -1,19395 | 0,42676 |
| DHQ, 42 days Control, 42 days | -0,50948 | 0,18737 | 0,67216 | 0,75286 | 0,05 | 0 | -1,41549 | 0,39652 |
| DHQ, 42 days Control, 56 days | -0,61277 | 0,18737 | 0,97231 | 0,49116 | 0,05 | 0 | -1,51878 | 0,29324 |
| DHQ, 42 days DHQ, 1 day | -0,38447 | 0,18737 | 0,38278 | 0,95272 | 0,05 | 0 | -1,29048 | 0,52153 |
| DHQ, 42 days DHQ, 7 days | -0,19098 | 0,18737 | 0,09445 | 0,99989 | 0,05 | 0 | -1,09699 | 0,71503 |
| DHQ, 42 days DHQ, 14 days | -0,16573 | 0,18737 | 0,07112 | 0,99997 | 0,05 | 0 | -1,07174 | 0,74028 |
| DHQ, 42 days DHQ, 28 days | -0,18322 | 0,17527 | 0,09935 | 0,99986 | 0,05 | 0 | -1,03071 | 0,66427 |
| DHQ, 56 days Control, 1 day | -0,79686 | 0,17527 | 1,87917 | 0,08378 | 0,05 | 0 | -1,64435 | 0,05063 |
| DHQ, 56 days Control, 7 days | -0,54969 | 0,17527 | 0,89422 | 0,55635 | 0,05 | 0 | -1,39719 | 0,2978 |
| DHQ, 56 days Control, 14 days | -0,60261 | 0,17527 | 1,07466 | 0,41259 | 0,05 | 0 | -1,4501 | 0,24488 |
| DHQ, 56 days Control, 28 days | -0,42143 | 0,16759 | 0,57486 | 0,83368 | 0,05 | 0 | -1,23178 | 0,38893 |
| DHQ, 56 days Control, 42 days | -0,54732 | 0,18737 | 0,77569 | 0,66091 | 0,05 | 0 | -1,45332 | 0,35869 |
| DHQ, 56 days Control, 56 days | -0,6506 | 0,18737 | 1,09609 | 0,39726 | 0,05 | 0 | -1,55661 | 0,2554 |
| DHQ, 56 days DHQ, 1 day | -0,42231 | 0,18737 | 0,46182 | 0,91218 | 0,05 | 0 | -1,32831 | 0,4837 |
| DHQ, 56 days DHQ, 7 days | -0,22882 | 0,18737 | 0,13558 | 0,99937 | 0,05 | 0 | -1,13482 | 0,67719 |
| DHQ, 56 days DHQ, 14 days | -0,20356 | 0,18737 | 0,1073 | 0,99979 | 0,05 | 0 | -1,10957 | 0,70244 |
| DHQ, 56 days DHQ, 28 days | -0,22106 | 0,17527 | 0,14461 | 0,99915 | 0,05 | 0 | -1,06855 | 0,62643 |
| DHQ, 56 days DHQ, 42 days | -0,03783 | 0,18737 | 0,00371 | 1 | 0,05 | 0 | -0,94384 | 0,86817 |

#### Tukey Test

| | MeanDiff | SEM | q Value | Prob | Alpha | Sig | LCL | UCL |
|---|---|---|---|---|---|---|---|---|
| Control, 7 days Control, 1 day | -0,24716 | 0,16227 | 2,15413 | 0,92215 | 0,05 | 0 | -0,82096 | 0,32663 |
| Control, 14 days Control, 1 day | -0,19425 | 0,16227 | 1,69296 | 0,98516 | 0,05 | 0 | -0,76804 | 0,37954 |
| Control, 14 days Control, 7 days | 0,05291 | 0,16227 | 0,46117 | 1 | 0,05 | 0 | -0,52088 | 0,62671 |
| Control, 28 days Control, 1 day | -0,37543 | 0,15394 | 3,44903 | 0,41254 | 0,05 | 0 | -0,91978 | 0,16891 |
| Control, 28 days Control, 7 days | -0,12827 | 0,15394 | 1,17837 | 0,99931 | 0,05 | 0 | -0,67261 | 0,41608 |
| Control, 28 days Control, 14 da | -0,18118 | 0,15394 | 1,66449 | 0,98697 | 0,05 | 0 | -0,72553 | 0,36316 |
| Control, 42 days Control, 1 day | -0,24954 | 0,17527 | 2,01351 | 0,94921 | 0,05 | 0 | -0,86931 | 0,37023 |
| Control, 42 days Control, 7 days | -0,00238 | 0,17527 | 0,01917 | 1 | 0,05 | 0 | -0,62214 | 0,61739 |
| Control, 42 days Control, 14 da | -0,05529 | 0,17527 | 0,44613 | 1 | 0,05 | 0 | -0,67506 | 0,56448 |
| Control, 42 days Control, 28 da | 0,12589 | 0,16759 | 1,06236 | 0,99974 | 0,05 | 0 | -0,46672 | 0,7185 |
| Control, 56 days Control, 1 day | -0,14625 | 0,17527 | 1,1801 | 0,9993 | 0,05 | 0 | -0,76602 | 0,47351 |
| Control, 56 days Control, 7 days | 0,10091 | 0,17527 | 0,81424 | 0,99998 | 0,05 | 0 | -0,51886 | 0,72068 |
| Control, 56 days Control, 14 da | 0,048 | 0,17527 | 0,38727 | 1 | 0,05 | 0 | -0,57177 | 0,66776 |
| Control, 56 days Control, 28 da | 0,22918 | 0,16759 | 1,93395 | 0,96121 | 0,05 | 0 | -0,36343 | 0,82179 |
| Control, 56 days Control, 42 da | 0,10329 | 0,18737 | 0,77958 | 0,99999 | 0,05 | 0 | -0,55927 | 0,76584 |
| DHQ, 1 day Control, 1 day | -0,37455 | 0,17527 | 3,0222 | 0,60261 | 0,05 | 0 | -0,99432 | 0,24522 |
| DHQ, 1 day Control, 7 days | -0,12739 | 0,17527 | 1,02786 | 0,99981 | 0,05 | 0 | -0,74715 | 0,49238 |
| DHQ, 1 day Control, 14 days | -0,1803 | 0,17527 | 1,45482 | 0,99559 | 0,05 | 0 | -0,80007 | 0,43947 |
| DHQ, 1 day Control, 28 days | 8,81778E-4 | 0,16759 | 0,00744 | 1 | 0,05 | 0 | -0,59173 | 0,59349 |
| DHQ, 1 day Control, 42 days | -0,12501 | 0,18737 | 0,94354 | 0,99992 | 0,05 | 0 | -0,78757 | 0,53755 |
| DHQ, 1 day Control, 56 days | -0,2283 | 0,18737 | 1,72312 | 0,98305 | 0,05 | 0 | -0,89085 | 0,43426 |
| DHQ, 7 days Control, 1 day | -0,56804 | 0,17527 | 4,58347 | 0,09634 | 0,05 | 0 | -1,18781 | 0,05172 |
| DHQ, 7 days Control, 7 days | -0,32088 | 0,17527 | 2,58913 | 0,78918 | 0,05 | 0 | -0,94064 | 0,29889 |
| DHQ, 7 days Control, 14 days | -0,37379 | 0,17527 | 3,01609 | 0,60541 | 0,05 | 0 | -0,99356 | 0,24597 |
| DHQ, 7 days Control, 28 days | -0,19261 | 0,16759 | 1,62537 | 0,98917 | 0,05 | 0 | -0,78522 | 0,4 |
| DHQ, 7 days Control, 42 days | -0,3185 | 0,18737 | 2,40397 | 0,85456 | 0,05 | 0 | -0,98106 | 0,34405 |
| DHQ, 7 days Control, 56 days | -0,42179 | 0,18737 | 3,18355 | 0,52889 | 0,05 | 0 | -1,08435 | 0,24077 |
| DHQ, 7 days DHQ, 1 day | -0,19349 | 0,18737 | 1,46043 | 0,99544 | 0,05 | 0 | -0,85605 | 0,46907 |
| DHQ, 14 days Control, 1 day | -0,59329 | 0,17527 | 4,78721 | 0,07038 | 0,05 | 0 | -1,21306 | 0,02647 |
| DHQ, 14 days Control, 7 days | -0,34613 | 0,17527 | 2,79287 | 0,70546 | 0,05 | 0 | -0,9659 | 0,27364 |
| DHQ, 14 days Control, 14 days | -0,39904 | 0,17527 | 3,21984 | 0,51248 | 0,05 | 0 | -1,01881 | 0,22072 |
| DHQ, 14 days Control, 28 days | -0,21786 | 0,16759 | 1,83845 | 0,97276 | 0,05 | 0 | -0,81047 | 0,37475 |
| DHQ, 14 days Control, 42 days | -0,34375 | 0,18737 | 2,59456 | 0,78708 | 0,05 | 0 | -1,00631 | 0,3188 |
| DHQ, 14 days Control, 56 days | -0,44704 | 0,18737 | 3,37414 | 0,44431 | 0,05 | 0 | -1,1096 | 0,21552 |
| DHQ, 14 days DHQ, 1 day | -0,21874 | 0,18737 | 1,65102 | 0,98776 | 0,05 | 0 | -0,8813 | 0,44381 |
| DHQ, 14 days DHQ, 7 days | -0,02525 | 0,18737 | 0,19059 | 1 | 0,05 | 0 | -0,68781 | 0,63731 |
| DHQ, 28 days Control, 1 day | -0,5758 | 0,16227 | 5,01833 | 0,04859 | 0,05 | 1 | -1,14959 | -0,0020 |
| DHQ, 28 days Control, 7 days | -0,32864 | 0,16227 | 2,86419 | 0,67412 | 0,05 | 0 | -0,90243 | 0,24515 |
| DHQ, 28 days Control, 14 days | -0,38155 | 0,16227 | 3,32537 | 0,46551 | 0,05 | 0 | -0,95534 | 0,19224 |
| DHQ, 28 days Control, 28 days | -0,20037 | 0,15394 | 1,84075 | 0,97251 | 0,05 | 0 | -0,74472 | 0,34398 |
| DHQ, 28 days Control, 42 days | -0,32626 | 0,17527 | 2,63256 | 0,77222 | 0,05 | 0 | -0,94603 | 0,2935 |
| DHQ, 28 days Control, 56 days | -0,42955 | 0,17527 | 3,46596 | 0,4055 | 0,05 | 0 | -1,04931 | 0,19022 |
| DHQ, 28 days DHQ, 1 day | -0,20125 | 0,17527 | 1,62387 | 0,98924 | 0,05 | 0 | -0,82102 | 0,41852 |
| DHQ, 28 days DHQ, 7 days | -0,00776 | 0,17527 | 0,0626 | 1 | 0,05 | 0 | -0,62752 | 0,61201 |
| DHQ, 28 days DHQ, 14 days | 0,01749 | 0,17527 | 0,14115 | 1 | 0,05 | 0 | -0,60227 | 0,63726 |
| DHQ, 42 days Control, 1 day | -0,75902 | 0,17527 | 6,12447 | 0,00704 | 0,05 | 1 | -1,37879 | -0,1392 |
| DHQ, 42 days Control, 7 days | -0,51186 | 0,17527 | 4,13013 | 0,18408 | 0,05 | 0 | -1,13163 | 0,10791 |
| DHQ, 42 days Control, 14 days | -0,56477 | 0,17527 | 4,55709 | 0,10023 | 0,05 | 0 | -1,18454 | 0,05499 |
| DHQ, 42 days Control, 28 days | -0,38359 | 0,16759 | 3,23699 | 0,50476 | 0,05 | 0 | -0,9762 | 0,20902 |
| DHQ, 42 days Control, 42 days | -0,50948 | 0,18737 | 3,84545 | 0,26502 | 0,05 | 0 | -1,17204 | 0,15307 |
| DHQ, 42 days Control, 56 days | -0,61277 | 0,18737 | 4,62503 | 0,09046 | 0,05 | 0 | -1,27533 | 0,04979 |
| DHQ, 42 days DHQ, 1 day | -0,38447 | 0,18737 | 2,90191 | 0,65725 | 0,05 | 0 | -1,04703 | 0,27808 |
| DHQ, 42 days DHQ, 7 days | -0,19098 | 0,18737 | 1,44148 | 0,99591 | 0,05 | 0 | -0,85354 | 0,47158 |
| DHQ, 42 days DHQ, 14 days | -0,16573 | 0,18737 | 1,25089 | 0,99881 | 0,05 | 0 | -0,82829 | 0,49683 |
| DHQ, 42 days DHQ, 28 days | -0,18322 | 0,17527 | 1,4784 | 0,99495 | 0,05 | 0 | -0,80299 | 0,43654 |
| DHQ, 56 days Control, 1 day | -0,79686 | 0,17527 | 6,42975 | 0,004 | 0,05 | 1 | -1,41662 | -0,1770 |
| DHQ, 56 days Control, 7 days | -0,54969 | 0,17527 | 4,43541 | 0,12 | 0,05 | 0 | -1,16946 | 0,07007 |
| DHQ, 56 days Control, 14 days | -0,60261 | 0,17527 | 4,86237 | 0,06249 | 0,05 | 0 | -1,22237 | 0,01716 |
| DHQ, 56 days Control, 28 days | -0,42143 | 0,16759 | 3,55626 | 0,36897 | 0,05 | 0 | -1,01404 | 0,17118 |
| DHQ, 56 days Control, 42 days | -0,54732 | 0,18737 | 4,13101 | 0,18387 | 0,05 | 0 | -1,20988 | 0,11524 |
| DHQ, 56 days Control, 56 days | -0,6506 | 0,18737 | 4,91059 | 0,05786 | 0,05 | 0 | -1,31316 | 0,01195 |
| DHQ, 56 days DHQ, 1 day | -0,42231 | 0,18737 | 3,18747 | 0,52711 | 0,05 | 0 | -1,08487 | 0,24025 |
| DHQ, 56 days DHQ, 7 days | -0,22882 | 0,18737 | 1,72704 | 0,98276 | 0,05 | 0 | -0,89137 | 0,43374 |
| DHQ, 56 days DHQ, 14 days | -0,20356 | 0,18737 | 1,53645 | 0,99308 | 0,05 | 0 | -0,86612 | 0,45899 |
| DHQ, 56 days DHQ, 28 days | -0,22106 | 0,17527 | 1,78368 | 0,9781 | 0,05 | 0 | -0,84082 | 0,39871 |
| DHQ, 56 days DHQ, 42 days | -0,03783 | 0,18737 | 0,28556 | 1 | 0,05 | 0 | -0,70039 | 0,62472 |

### Grouping Letters

#### Bonferroni Test

| | Mean | Groups | |
|---|---|---|---|
| Control, 1 day | 1 | A | |
| Control, 56 days | 0,85375 | A | B |
| Control, 14 days | 0,80575 | A | B |
| Control, 7 days | 0,75284 | A | B |
| Control, 42 days | 0,75046 | A | B |
| DHQ, 1 day | 0,62545 | A | B |
| Control, 28 days | 0,62457 | A | B |
| DHQ, 7 days | 0,43196 | A | B |
| DHQ, 28 days | 0,4242 | A | B |
| DHQ, 14 days | 0,40671 | A | B |
| DHQ, 42 days | 0,24098 | | B |
| DHQ, 56 days | 0,20314 | | B |

Means that do not share a letter are significantly different.

#### Scheffe Test

| | Mean | Groups |
|---|---|---|
| Control, 1 day | 1 | A |
| Control, 56 days | 0,85375 | A |
| Control, 14 days | 0,80575 | A |
| Control, 7 days | 0,75284 | A |
| Control, 42 days | 0,75046 | A |
| DHQ, 1 day | 0,62545 | A |
| Control, 28 days | 0,62457 | A |
| DHQ, 7 days | 0,43196 | A |
| DHQ, 28 days | 0,4242 | A |
| DHQ, 14 days | 0,40671 | A |
| DHQ, 42 days | 0,24098 | A |
| DHQ, 56 days | 0,20314 | A |

Means that do not share a letter are significantly different.

#### Tukey Test

| | Mean | Groups | |
|---|---|---|---|
| Control, 1 day | 1 | A | |
| Control, 56 days | 0,85375 | A | B |
| Control, 14 days | 0,80575 | A | B |
| Control, 7 days | 0,75284 | A | B |
| Control, 42 days | 0,75046 | A | B |
| DHQ, 1 day | 0,62545 | A | B |
| Control, 28 days | 0,62457 | A | B |
| DHQ, 7 days | 0,43196 | A | B |
| DHQ, 28 days | 0,4242 | | B |
| DHQ, 14 days | 0,40671 | A | B |
| DHQ, 42 days | 0,24098 | | B |
| DHQ, 56 days | 0,20314 | | B |

Means that do not share a letter are significantly different.

Sig equals 1 indicates that the difference of the means is significant at the 0,05 level.
Sig equals 0 indicates that the difference of the means is not significant at the 0,05 level.

## Powers

| | Alpha | Sample Size | Power |
|---|---|---|---|
| Actual Power | 0,05 | 42 | 0,9887 |

*ANOVAOneWay (28.10.2024 12:44:25)*

*Notes*

| | |
|---|---|
| Description | Perform One-Way ANOVA |
| User Name | yulia |
| Operation Time | 28.10.2024 12:44:25 |
| Report Status | New Analysis Report |
| Data Filter | No |

*Input Data*

| | Data | Range |
|---|---|---|
| Control, 1 day | [Book1]"MitoTracker Red"!A"Control, 1 day" | [1*:4*] |
| Control, 7 days | [Book1]"MitoTracker Red"!B"Control, 7 days" | [1*:4*] |
| Control, 14 days | [Book1]"MitoTracker Red"!C"Control, 14 days" | [1*:4*] |
| Control, 28 days | [Book1]"MitoTracker Red"!D"Control, 28 days" | [1*:5*] |
| Control, 42 days | [Book1]"MitoTracker Red"!E"Control, 42 days" | [1*:3*] |
| Control, 56 days | [Book1]"MitoTracker Red"!F"Control, 56 days" | [1*:3*] |
| DHQ, 1 day | [Book1]"MitoTracker Red"!G"DHQ, 1 day" | [1*:3*] |
| DHQ, 7 days | [Book1]"MitoTracker Red"!H"DHQ, 7 days" | [1*:3*] |
| DHQ, 14 days | [Book1]"MitoTracker Red"!I"DHQ, 14 days" | [1*:3*] |
| DHQ, 28 days | [Book1]"MitoTracker Red"!J"DHQ, 28 days" | [1*:4*] |
| DHQ, 42 days | [Book1]"MitoTracker Red"!K"DHQ, 42 days" | [1*:3*] |
| DHQ, 56 days | [Book1]"MitoTracker Red"!L"DHQ, 56 days" | [1*:3*] |

*Descriptive Statistics*

| | N Analysis | N Missing | Mean | Standard Deviation | SE of Mean |
|---|---|---|---|---|---|
| Control, 1 day | 4 | 0 | 1 | 0 | 0 |
| Control, 7 days | 4 | 0 | 0,72321 | 0,21156 | 0,10578 |
| Control, 14 days | 4 | 0 | 0,78618 | 0,34769 | 0,17385 |
| Control, 28 days | 5 | 0 | 0,87282 | 0,30109 | 0,13465 |
| Control, 42 days | 3 | 0 | 1,16037 | 0,35901 | 0,20728 |
| Control, 56 days | 3 | 0 | 1,10693 | 0,29958 | 0,17297 |
| DHQ, 1 day | 3 | 0 | 0,76594 | 0,31339 | 0,18093 |
| DHQ, 7 days | 3 | 0 | 0,62981 | 0,43131 | 0,24902 |
| DHQ, 14 days | 3 | 0 | 0,61273 | 0,05169 | 0,02985 |
| DHQ, 28 days | 4 | 0 | 1,11388 | 0,70592 | 0,35296 |
| DHQ, 42 days | 3 | 0 | 0,41333 | 0,27637 | 0,15956 |
| DHQ, 56 days | 3 | 0 | 0,22106 | 0,16024 | 0,09251 |

*ANOVA*

*Overall ANOVA*

| | DF | Sum of Squares | Mean Square | F Value | Prob>F |
|---|---|---|---|---|---|
| Model | 11 | 2,92682 | 0,26607 | 2,23607 | 0,03967 |
| Error | 30 | 3,56977 | 0,11899 | | |
| Total | 41 | 6,49659 | | | |

Null Hypothesis: The means of all levels are equal.
Alternative Hypothesis: The means of one or more levels are different.
At the 0.05 level, the population means are significantly different.

*Fit Statistics*

| | R-Square | Coeff Var | Root MSE | Data Mean |
|---|---|---|---|---|
| | 0,45052 | 0,43135 | 0,34495 | 0,79971 |

*Means Comparisons*

*Bonferroni Test*

| | MeanDiff | SEM | t Value | Prob | Alpha | Sig | LCL | UCL |
|---|---|---|---|---|---|---|---|---|
| Control, 7 days  Control, 1 day | -0,27679 | 0,24392 | -1,13477 | 1 | 0,05 | 0 | -1,19122 | 0,63764 |
| Control, 14 days  Control, 1 day | -0,21382 | 0,24392 | -0,87661 | 1 | 0,05 | 0 | -1,12825 | 0,7006 |
| Control, 14 days  Control, 7 days | 0,06297 | 0,24392 | 0,25815 | 1 | 0,05 | 0 | -0,85146 | 0,9774 |
| Control, 28 days  Control, 1 day | -0,12718 | 0,2314 | -0,54963 | 1 | 0,05 | 0 | -0,99469 | 0,74032 |
| Control, 28 days  Control, 7 days | 0,14961 | 0,2314 | 0,64653 | 1 | 0,05 | 0 | -0,71789 | 1,01711 |
| Control, 28 days  Control, 14 da | 0,08664 | 0,2314 | 0,37441 | 1 | 0,05 | 0 | -0,78086 | 0,95414 |
| Control, 42 days  Control, 1 day | 0,16037 | 0,26346 | 0,60872 | 1 | 0,05 | 0 | -0,82732 | 1,14807 |
| Control, 42 days  Control, 7 days | 0,43716 | 0,26346 | 1,65931 | 1 | 0,05 | 0 | -0,55053 | 1,42486 |
| Control, 42 days  Control, 14 da | 0,3742 | 0,26346 | 1,4203 | 1 | 0,05 | 0 | -0,6135 | 1,36189 |
| Control, 42 days  Control, 28 da | 0,28756 | 0,25192 | 1,14147 | 1 | 0,05 | 0 | -0,65686 | 1,23197 |
| Control, 56 days  Control, 1 day | 0,10693 | 0,26346 | 0,40585 | 1 | 0,05 | 0 | -0,88077 | 1,09462 |
| Control, 56 days  Control, 7 days | 0,38372 | 0,26346 | 1,45645 | 1 | 0,05 | 0 | -0,60398 | 1,37141 |
| Control, 56 days  Control, 14 da | 0,32075 | 0,26346 | 1,21744 | 1 | 0,05 | 0 | -0,66694 | 1,30844 |
| Control, 56 days  Control, 28 da | 0,23411 | 0,25192 | 0,92932 | 1 | 0,05 | 0 | -0,7103 | 1,17853 |
| Control, 56 days  Control, 42 da | -0,05345 | 0,28165 | -0,18976 | 1 | 0,05 | 0 | -1,10933 | 1,00244 |
| DHQ, 1 day  Control, 1 day | -0,23406 | 0,26346 | -0,88838 | 1 | 0,05 | 0 | -1,22175 | 0,75364 |
| DHQ, 1 day  Control, 7 days | 0,04274 | 0,26346 | 0,16221 | 1 | 0,05 | 0 | -0,94496 | 1,03043 |
| DHQ, 1 day  Control, 14 days | -0,02023 | 0,26346 | -0,0768 | 1 | 0,05 | 0 | -1,00793 | 0,96746 |
| DHQ, 1 day  Control, 28 days | -0,10687 | 0,25192 | -0,42423 | 1 | 0,05 | 0 | -1,05129 | 0,83754 |
| DHQ, 1 day  Control, 42 days | -0,39443 | 0,28165 | -1,40041 | 1 | 0,05 | 0 | -1,45032 | 0,66146 |
| DHQ, 1 day  Control, 56 days | -0,34098 | 0,28165 | -1,21065 | 1 | 0,05 | 0 | -1,39687 | 0,71491 |
| DHQ, 7 days  Control, 1 day | -0,37019 | 0,26346 | -1,4051 | 1 | 0,05 | 0 | -1,35788 | 0,6175 |
| DHQ, 7 days  Control, 7 days | -0,0934 | 0,26346 | -0,35451 | 1 | 0,05 | 0 | -1,08109 | 0,89429 |
| DHQ, 7 days  Control, 14 days | -0,15637 | 0,26346 | -0,59351 | 1 | 0,05 | 0 | -1,14406 | 0,83133 |
| DHQ, 7 days  Control, 28 days | -0,24301 | 0,25192 | -0,96463 | 1 | 0,05 | 0 | -1,18742 | 0,70141 |
| DHQ, 7 days  Control, 42 days | -0,53056 | 0,28165 | -1,88375 | 1 | 0,05 | 0 | -1,58645 | 0,52532 |
| DHQ, 7 days  Control, 56 days | -0,47712 | 0,28165 | -1,69399 | 1 | 0,05 | 0 | -1,53301 | 0,57877 |
| DHQ, 7 days  DHQ, 1 day | -0,13614 | 0,28165 | -0,48334 | 1 | 0,05 | 0 | -1,19202 | 0,91975 |
| DHQ, 14 days  Control, 1 day | -0,38727 | 0,26346 | -1,46994 | 1 | 0,05 | 0 | -1,37497 | 0,60042 |
| DHQ, 14 days  Control, 7 days | -0,11048 | 0,26346 | -0,41934 | 1 | 0,05 | 0 | -1,09817 | 0,87721 |
| DHQ, 14 days  Control, 14 days | -0,17345 | 0,26346 | -0,65835 | 1 | 0,05 | 0 | -1,16114 | 0,81424 |
| DHQ, 14 days  Control, 28 days | -0,26009 | 0,25192 | -1,03243 | 1 | 0,05 | 0 | -1,2045 | 0,68433 |
| DHQ, 14 days  Control, 42 days | -0,54765 | 0,28165 | -1,9444 | 1 | 0,05 | 0 | -1,60353 | 0,50824 |
| DHQ, 14 days  Control, 56 days | -0,4942 | 0,28165 | -1,75464 | 1 | 0,05 | 0 | -1,55009 | 0,56169 |
| DHQ, 14 days  DHQ, 1 day | -0,15322 | 0,28165 | -0,54399 | 1 | 0,05 | 0 | -1,20911 | 0,90267 |
| DHQ, 14 days  DHQ, 7 days | -0,01708 | 0,28165 | -0,06065 | 1 | 0,05 | 0 | -1,07297 | 1,03881 |
| DHQ, 28 days  Control, 1 day | 0,11388 | 0,24392 | 0,4669 | 1 | 0,05 | 0 | -0,80054 | 1,02831 |
| DHQ, 28 days  Control, 7 days | 0,39068 | 0,24392 | 1,60167 | 1 | 0,05 | 0 | -0,52375 | 1,3051 |
| DHQ, 28 days  Control, 14 days | 0,32771 | 0,24392 | 1,34351 | 1 | 0,05 | 0 | -0,58672 | 1,24213 |
| DHQ, 28 days  Control, 28 days | 0,24107 | 0,2314 | 1,04178 | 1 | 0,05 | 0 | -0,62643 | 1,10857 |
| DHQ, 28 days  Control, 42 days | -0,04649 | 0,26346 | -0,17645 | 1 | 0,05 | 0 | -1,03418 | 0,9412 |
| DHQ, 28 days  Control, 56 days | 0,00696 | 0,26346 | 0,02641 | 1 | 0,05 | 0 | -0,98074 | 0,99465 |
| DHQ, 28 days  DHQ, 1 day | 0,34794 | 0,26346 | 1,32064 | 1 | 0,05 | 0 | -0,63975 | 1,33563 |
| DHQ, 28 days  DHQ, 7 days | 0,48408 | 0,26346 | 1,83736 | 1 | 0,05 | 0 | -0,50362 | 1,47177 |
| DHQ, 28 days  DHQ, 14 days | 0,50116 | 0,26346 | 1,9022 | 1 | 0,05 | 0 | -0,48654 | 1,48885 |
| DHQ, 42 days  Control, 1 day | -0,58667 | 0,26346 | -2,22676 | 1 | 0,05 | 0 | -1,57436 | 0,40103 |
| DHQ, 42 days  Control, 7 days | -0,30987 | 0,26346 | -1,17616 | 1 | 0,05 | 0 | -1,29757 | 0,67782 |
| DHQ, 42 days  Control, 14 days | -0,37284 | 0,26346 | -1,41517 | 1 | 0,05 | 0 | -1,36054 | 0,61485 |
| DHQ, 42 days  Control, 28 days | -0,45948 | 0,25192 | -1,82393 | 1 | 0,05 | 0 | -1,4039 | 0,48493 |
| DHQ, 42 days  Control, 42 days | -0,74704 | 0,28165 | -2,65234 | 0,834 | 0,05 | 0 | -1,80293 | 0,30885 |
| DHQ, 42 days  Control, 56 days | -0,69359 | 0,28165 | -2,46258 | 1 | 0,05 | 0 | -1,74948 | 0,3623 |
| DHQ, 42 days  DHQ, 1 day | -0,35261 | 0,28165 | -1,25193 | 1 | 0,05 | 0 | -1,4085 | 0,70328 |
| DHQ, 42 days  DHQ, 7 days | -0,21648 | 0,28165 | -0,76859 | 1 | 0,05 | 0 | -1,27236 | 0,83941 |
| DHQ, 42 days  DHQ, 14 days | -0,19939 | 0,28165 | -0,70794 | 1 | 0,05 | 0 | -1,25528 | 0,85649 |
| DHQ, 42 days  DHQ, 28 days | -0,70055 | 0,26346 | -2,65902 | 0,821 | 0,05 | 0 | -1,68824 | 0,28714 |
| DHQ, 56 days  Control, 1 day | -0,77894 | 0,26346 | -2,95657 | 0,396 | 0,05 | 0 | -1,76664 | 0,20875 |
| DHQ, 56 days  Control, 7 days | -0,50215 | 0,26346 | -1,90598 | 1 | 0,05 | 0 | -1,48985 | 0,48554 |
| DHQ, 56 days  Control, 14 days | -0,56512 | 0,26346 | -2,14499 | 1 | 0,05 | 0 | -1,55282 | 0,42257 |
| DHQ, 56 days  Control, 28 days | -0,65176 | 0,25192 | -2,5872 | 0,974 | 0,05 | 0 | -1,59618 | 0,29265 |
| DHQ, 56 days  Control, 42 days | -0,93932 | 0,28165 | -3,33502 | 0,150 | 0,05 | 0 | -1,99521 | 0,11657 |
| DHQ, 56 days  Control, 56 days | -0,88587 | 0,28165 | -3,14526 | 0,246 | 0,05 | 0 | -1,94176 | 0,17002 |
| DHQ, 56 days  DHQ, 1 day | -0,54489 | 0,28165 | -1,93462 | 1 | 0,05 | 0 | -1,60078 | 0,511 |
| DHQ, 56 days  DHQ, 7 days | -0,40875 | 0,28165 | -1,45127 | 1 | 0,05 | 0 | -1,46464 | 0,64713 |
| DHQ, 56 days  DHQ, 14 days | -0,39167 | 0,28165 | -1,39062 | 1 | 0,05 | 0 | -1,44756 | 0,66422 |
| DHQ, 56 days  DHQ, 28 days | -0,89283 | 0,26346 | -3,38884 | 0,130 | 0,05 | 0 | -1,88052 | 0,09486 |
| DHQ, 56 days  DHQ, 42 days | -0,19228 | 0,28165 | -0,68268 | 1 | 0,05 | 0 | -1,24817 | 0,86361 |

*Scheffe Test*

| | MeanDiff | SEM | F Value | Prob | Alpha | Sig | LCL | UCL |
|---|---|---|---|---|---|---|---|---|
| Control, 7 days  Control, 1 day | -0,27679 | 0,24392 | 0,11706 | 0,99968 | 0,05 | 0 | -1,45624 | 0,90265 |
| Control, 14 days  Control, 1 day | -0,21382 | 0,24392 | 0,06986 | 0,99998 | 0,05 | 0 | -1,39327 | 0,96562 |
| Control, 14 days  Control, 7 days | 0,06297 | 0,24392 | 0,00606 | 1 | 0,05 | 0 | -1,11648 | 1,24241 |
| Control, 28 days  Control, 1 day | -0,12718 | 0,2314 | 0,02746 | 1 | 0,05 | 0 | -1,2461 | 0,99174 |
| Control, 28 days  Control, 7 days | 0,14961 | 0,2314 | 0,038 | 1 | 0,05 | 0 | -0,96931 | 1,26853 |
| Control, 28 days  Control, 14 da | 0,08664 | 0,2314 | 0,01274 | 1 | 0,05 | 0 | -1,03228 | 1,20556 |
| Control, 42 days  Control, 1 day | 0,16037 | 0,26346 | 0,03368 | 1 | 0,05 | 0 | -1,11357 | 1,43432 |
| Control, 42 days  Control, 7 days | 0,43716 | 0,26346 | 0,2503 | 0,99054 | 0,05 | 0 | -0,83678 | 1,71111 |
| Control, 42 days  Control, 14 da | 0,3742 | 0,26346 | 0,18339 | 0,99749 | 0,05 | 0 | -0,89975 | 1,64814 |
| Control, 42 days  Control, 28 da | 0,28756 | 0,25192 | 0,11845 | 0,99967 | 0,05 | 0 | -0,93057 | 1,50568 |
| Control, 56 days  Control, 1 day | 0,10693 | 0,26346 | 0,01497 | 1 | 0,05 | 0 | -1,16702 | 1,38087 |
| Control, 56 days  Control, 7 days | 0,38372 | 0,26346 | 0,19284 | 0,99687 | 0,05 | 0 | -0,89023 | 1,65766 |
| Control, 56 days  Control, 14 da | 0,32075 | 0,26346 | 0,13474 | 0,99939 | 0,05 | 0 | -0,9532 | 1,5947 |
| Control, 56 days  Control, 28 da | 0,23411 | 0,25192 | 0,07851 | 0,99996 | 0,05 | 0 | -0,98401 | 1,45224 |
| Control, 56 days  Control, 42 da | -0,05345 | 0,28165 | 0,00327 | 1 | 0,05 | 0 | -1,41535 | 1,30846 |
| DHQ, 1 day  Control, 1 day | -0,23406 | 0,26346 | 0,07175 | 0,99997 | 0,05 | 0 | -1,508 | 1,03989 |
| DHQ, 1 day  Control, 7 days | 0,04274 | 0,26346 | 0,00239 | 1 | 0,05 | 0 | -1,23121 | 1,31668 |
| DHQ, 1 day  Control, 14 days | -0,02023 | 0,26346 | 5,36137E-4 | 1 | 0,05 | 0 | -1,29418 | 1,25371 |
| DHQ, 1 day  Control, 28 days | -0,10687 | 0,25192 | 0,01636 | 1 | 0,05 | 0 | -1,325 | 1,11125 |
| DHQ, 1 day  Control, 42 days | -0,39443 | 0,28165 | 0,17829 | 0,99778 | 0,05 | 0 | -1,75633 | 0,96748 |
| DHQ, 1 day  Control, 56 days | -0,34098 | 0,28165 | 0,13324 | 0,99942 | 0,05 | 0 | -1,70289 | 1,02092 |
| DHQ, 7 days  Control, 1 day | -0,37019 | 0,26346 | 0,17948 | 0,99772 | 0,05 | 0 | -1,64414 | 0,90376 |
| DHQ, 7 days  Control, 7 days | -0,0934 | 0,26346 | 0,01143 | 1 | 0,05 | 0 | -1,36734 | 1,18055 |
| DHQ, 7 days  Control, 14 days | -0,15637 | 0,26346 | 0,03202 | 1 | 0,05 | 0 | -1,43031 | 1,11758 |
| DHQ, 7 days  Control, 28 days | -0,24301 | 0,25192 | 0,08459 | 0,99994 | 0,05 | 0 | -1,46113 | 0,97512 |
| DHQ, 7 days  Control, 42 days | -0,53056 | 0,28165 | 0,32259 | 0,97446 | 0,05 | 0 | -1,89247 | 0,83134 |
| DHQ, 7 days  Control, 56 days | -0,47712 | 0,28165 | 0,26087 | 0,98881 | 0,05 | 0 | -1,83902 | 0,88479 |
| DHQ, 7 days  DHQ, 1 day | -0,13614 | 0,28165 | 0,02124 | 1 | 0,05 | 0 | -1,49804 | 1,22577 |
| DHQ, 14 days  Control, 1 day | -0,38727 | 0,26346 | 0,19643 | 0,9966 | 0,05 | 0 | -1,66122 | 0,88667 |
| DHQ, 14 days  Control, 7 days | -0,11048 | 0,26346 | 0,01599 | 1 | 0,05 | 0 | -1,38443 | 1,16346 |
| DHQ, 14 days  Control, 14 days | -0,17345 | 0,26346 | 0,0394 | 1 | 0,05 | 0 | -1,4474 | 1,1005 |
| DHQ, 14 days  Control, 28 days | -0,26009 | 0,25192 | 0,0969 | 0,99987 | 0,05 | 0 | -1,47821 | 0,95804 |
| DHQ, 14 days  Control, 42 days | -0,54765 | 0,28165 | 0,3437 | 0,96774 | 0,05 | 0 | -1,90955 | 0,81426 |
| DHQ, 14 days  Control, 56 days | -0,4942 | 0,28165 | 0,27989 | 0,98519 | 0,05 | 0 | -1,8561 | 0,86771 |
| DHQ, 14 days  DHQ, 1 day | -0,15322 | 0,28165 | 0,0269 | 1 | 0,05 | 0 | -1,51512 | 1,20869 |
| DHQ, 14 days  DHQ, 7 days | -0,01708 | 0,28165 | 3,34377E-4 | 1 | 0,05 | 0 | -1,37899 | 1,34482 |
| DHQ, 28 days  Control, 1 day | 0,11388 | 0,24392 | 0,01982 | 1 | 0,05 | 0 | -1,06556 | 1,29333 |
| DHQ, 28 days  Control, 7 days | 0,39068 | 0,24392 | 0,23321 | 0,99293 | 0,05 | 0 | -0,78877 | 1,57012 |
| DHQ, 28 days  Control, 14 days | 0,32771 | 0,24392 | 0,16409 | 0,99847 | 0,05 | 0 | -0,85174 | 1,50715 |
| DHQ, 28 days  Control, 28 days | 0,24107 | 0,2314 | 0,09866 | 0,99986 | 0,05 | 0 | -0,87785 | 1,35999 |
| DHQ, 28 days  Control, 42 days | -0,04649 | 0,26346 | 0,00283 | 1 | 0,05 | 0 | -1,32043 | 1,22746 |
| DHQ, 28 days  Control, 56 days | 0,00696 | 0,26346 | 6,34008E-5 | 1 | 0,05 | 0 | -1,26699 | 1,2809 |
| DHQ, 28 days  DHQ, 1 day | 0,34794 | 0,26346 | 0,15855 | 0,99869 | 0,05 | 0 | -0,92601 | 1,62189 |
| DHQ, 28 days  DHQ, 7 days | 0,48408 | 0,26346 | 0,3069 | 0,97884 | 0,05 | 0 | -0,78987 | 1,75802 |
| DHQ, 28 days  DHQ, 14 days | 0,50116 | 0,26346 | 0,32894 | 0,97254 | 0,05 | 0 | -0,77279 | 1,7751 |
| DHQ, 42 days  Control, 1 day | -0,58667 | 0,26346 | 0,45077 | 0,91863 | 0,05 | 0 | -1,86061 | 0,68728 |
| DHQ, 42 days  Control, 7 days | -0,30987 | 0,26346 | 0,12576 | 0,99956 | 0,05 | 0 | -1,58382 | 0,96407 |
| DHQ, 42 days  Control, 14 days | -0,37284 | 0,26346 | 0,18206 | 0,99757 | 0,05 | 0 | -1,64679 | 0,9011 |
| DHQ, 42 days  Control, 28 days | -0,45948 | 0,25192 | 0,30243 | 0,97999 | 0,05 | 0 | -1,67761 | 0,75864 |
| DHQ, 42 days  Control, 42 days | -0,74704 | 0,28165 | 0,63954 | 0,78089 | 0,05 | 0 | -2,10894 | 0,61487 |
| DHQ, 42 days  Control, 56 days | -0,69359 | 0,28165 | 0,5513 | 0,8517 | 0,05 | 0 | -2,0555 | 0,66831 |
| DHQ, 42 days  DHQ, 1 day | -0,35261 | 0,28165 | 0,14249 | 0,9992 | 0,05 | 0 | -1,71452 | 1,00929 |
| DHQ, 42 days  DHQ, 7 days | -0,21648 | 0,28165 | 0,0537 | 0,99999 | 0,05 | 0 | -1,57838 | 1,14543 |
| DHQ, 42 days  DHQ, 14 days | -0,19939 | 0,28165 | 0,04556 | 1 | 0,05 | 0 | -1,5613 | 1,16251 |
| DHQ, 42 days  DHQ, 28 days | -0,70055 | 0,26346 | 0,64276 | 0,77815 | 0,05 | 0 | -1,9745 | 0,5734 |
| DHQ, 56 days  Control, 1 day | -0,77894 | 0,26346 | 0,79467 | 0,64393 | 0,05 | 0 | -2,05289 | 0,495 |
| DHQ, 56 days  Control, 7 days | -0,50215 | 0,26346 | 0,33025 | 0,97213 | 0,05 | 0 | -1,7761 | 0,77179 |
| DHQ, 56 days  Control, 14 days | -0,56512 | 0,26346 | 0,41827 | 0,93616 | 0,05 | 0 | -1,83907 | 0,70882 |
| DHQ, 56 days  Control, 28 days | -0,65176 | 0,25192 | 0,60851 | 0,80675 | 0,05 | 0 | -1,86989 | 0,56636 |
| DHQ, 56 days  Control, 42 days | -0,93932 | 0,28165 | 1,01113 | 0,46036 | 0,05 | 0 | -2,30122 | 0,42259 |
| DHQ, 56 days  Control, 56 days | -0,88587 | 0,28165 | 0,89933 | 0,55196 | 0,05 | 0 | -2,24778 | 0,47603 |
| DHQ, 56 days  DHQ, 1 day | -0,54489 | 0,28165 | 0,34025 | 0,96891 | 0,05 | 0 | -1,9068 | 0,81702 |
| DHQ, 56 days  DHQ, 7 days | -0,40875 | 0,28165 | 0,19147 | 0,99696 | 0,05 | 0 | -1,77066 | 0,95315 |
| DHQ, 56 days  DHQ, 14 days | -0,39167 | 0,28165 | 0,1758 | 0,99792 | 0,05 | 0 | -1,75358 | 0,97023 |
| DHQ, 56 days  DHQ, 28 days | -0,89283 | 0,26346 | 1,04402 | 0,43521 | 0,05 | 0 | -2,16678 | 0,38112 |
| DHQ, 56 days  DHQ, 42 days | -0,19228 | 0,28165 | 0,04237 | 1 | 0,05 | 0 | -1,55418 | 1,16963 |

*Tukey Test*

| | MeanDiff | SEM | q Value | Prob | Alpha | Sig | LCL | UCL |
|---|---|---|---|---|---|---|---|---|
| Control, 7 days  Control, 1 day | -0,27679 | 0,24392 | 1,60481 | 0,9902 | 0,05 | 0 | -1,13931 | 0,58573 |
| Control, 14 days  Control, 1 day | -0,21382 | 0,24392 | 1,23972 | 0,9989 | 0,05 | 0 | -1,07634 | 0,6487 |
| Control, 14 days  Control, 7 days | 0,06297 | 0,24392 | 0,36509 | 1 | 0,05 | 0 | -0,79955 | 0,92549 |
| Control, 28 days  Control, 1 day | -0,12718 | 0,2314 | 0,77729 | 0,99999 | 0,05 | 0 | -0,94544 | 0,69108 |
| Control, 28 days  Control, 7 days | 0,14961 | 0,2314 | 0,91433 | 0,99994 | 0,05 | 0 | -0,66865 | 0,96787 |
| Control, 28 days  Control, 14 da | 0,08664 | 0,2314 | 0,52949 | 1 | 0,05 | 0 | -0,73162 | 0,9049 |
| Control, 42 days  Control, 1 day | 0,16037 | 0,26346 | 0,86085 | 0,99997 | 0,05 | 0 | -0,77126 | 1,092 |
| Control, 42 days  Control, 7 days | 0,43716 | 0,26346 | 2,34662 | 0,87228 | 0,05 | 0 | -0,49446 | 1,36879 |
| Control, 42 days  Control, 14 da | 0,3742 | 0,26346 | 2,00861 | 0,95002 | 0,05 | 0 | -0,55743 | 1,30583 |
| Control, 42 days  Control, 28 da | 0,28756 | 0,25192 | 1,61429 | 0,98973 | 0,05 | 0 | -0,60325 | 1,17837 |
| Control, 56 days  Control, 1 day | 0,10693 | 0,26346 | 0,57396 | 1 | 0,05 | 0 | -0,8247 | 1,03856 |
| Control, 56 days  Control, 7 days | 0,38372 | 0,26346 | 2,05973 | 0,94117 | 0,05 | 0 | -0,54791 | 1,31535 |
| Control, 56 days  Control, 14 da | 0,32075 | 0,26346 | 1,72172 | 0,98315 | 0,05 | 0 | -0,61088 | 1,25238 |
| Control, 56 days  Control, 28 da | 0,23411 | 0,25192 | 1,31425 | 0,99815 | 0,05 | 0 | -0,6567 | 1,12492 |
| Control, 56 days  Control, 42 da | -0,05345 | 0,28165 | 0,26836 | 1 | 0,05 | 0 | -1,0494 | 0,94251 |
| DHQ, 1 day  Control, 1 day | -0,23406 | 0,26346 | 1,25636 | 0,99876 | 0,05 | 0 | -1,16568 | 0,69757 |
| DHQ, 1 day  Control, 7 days | 0,04274 | 0,26346 | 0,2294 | 1 | 0,05 | 0 | -0,88889 | 0,97437 |
| DHQ, 1 day  Control, 14 days | -0,02023 | 0,26346 | 0,1086 | 1 | 0,05 | 0 | -0,95186 | 0,9114 |
| DHQ, 1 day  Control, 28 days | -0,10687 | 0,25192 | 0,59995 | 1 | 0,05 | 0 | -0,99768 | 0,78394 |
| DHQ, 1 day  Control, 42 days | -0,39443 | 0,28165 | 1,98048 | 0,95447 | 0,05 | 0 | -1,39038 | 0,60153 |
| DHQ, 1 day  Control, 56 days | -0,34098 | 0,28165 | 1,71211 | 0,98385 | 0,05 | 0 | -1,33694 | 0,65497 |
| DHQ, 7 days  Control, 1 day | -0,37019 | 0,26346 | 1,98711 | 0,95345 | 0,05 | 0 | -1,30182 | 0,56144 |
| DHQ, 7 days  Control, 7 days | -0,0934 | 0,26346 | 0,50135 | 1 | 0,05 | 0 | -1,02503 | 0,83823 |
| DHQ, 7 days  Control, 14 days | -0,15637 | 0,26346 | 0,83935 | 0,99997 | 0,05 | 0 | -1,088 | 0,77526 |
| DHQ, 7 days  Control, 28 days | -0,24301 | 0,25192 | 1,36419 | 0,99745 | 0,05 | 0 | -1,13381 | 0,6478 |
| DHQ, 7 days  Control, 42 days | -0,53056 | 0,28165 | 2,66403 | 0,75961 | 0,05 | 0 | -1,52652 | 0,46539 |
| DHQ, 7 days  Control, 56 days | -0,47712 | 0,28165 | 2,39567 | 0,8572 | 0,05 | 0 | -1,47307 | 0,51884 |
| DHQ, 7 days  DHQ, 1 day | -0,13614 | 0,28165 | 0,68355 | 1 | 0,05 | 0 | -1,13209 | 0,85982 |
| DHQ, 14 days  Control, 1 day | -0,38727 | 0,26346 | 2,0788 | 0,93761 | 0,05 | 0 | -1,3189 | 0,54436 |
| DHQ, 14 days  Control, 7 days | -0,11048 | 0,26346 | 0,59304 | 1 | 0,05 | 0 | -1,04211 | 0,82115 |
| DHQ, 14 days  Control, 14 days | -0,17345 | 0,26346 | 0,93104 | 0,99993 | 0,05 | 0 | -1,10508 | 0,75818 |
| DHQ, 14 days  Control, 28 days | -0,26009 | 0,25192 | 1,46008 | 0,99545 | 0,05 | 0 | -1,1509 | 0,63072 |
| DHQ, 14 days  Control, 42 days | -0,54765 | 0,28165 | 2,7498 | 0,72397 | 0,05 | 0 | -1,5436 | 0,44831 |
| DHQ, 14 days  Control, 56 days | -0,4942 | 0,28165 | 2,48144 | 0,82865 | 0,05 | 0 | -1,49015 | 0,50175 |
| DHQ, 14 days  DHQ, 1 day | -0,15322 | 0,28165 | 0,76932 | 0,99999 | 0,05 | 0 | -1,14917 | 0,84274 |
| DHQ, 14 days  DHQ, 7 days | -0,01708 | 0,28165 | 0,08577 | 1 | 0,05 | 0 | -1,01304 | 0,97887 |
| DHQ, 28 days  Control, 1 day | 0,11388 | 0,24392 | 0,66029 | 1 | 0,05 | 0 | -0,74864 | 0,97641 |
| DHQ, 28 days  Control, 7 days | 0,39068 | 0,24392 | 2,2651 | 0,89523 | 0,05 | 0 | -0,47185 | 1,2532 |
| DHQ, 28 days  Control, 14 days | 0,32771 | 0,24392 | 1,90001 | 0,96566 | 0,05 | 0 | -0,53481 | 1,19023 |
| DHQ, 28 days  Control, 28 days | 0,24107 | 0,2314 | 1,4733 | 0,9951 | 0,05 | 0 | -0,57719 | 1,05933 |
| DHQ, 28 days  Control, 42 days | -0,04649 | 0,26346 | 0,24954 | 1 | 0,05 | 0 | -0,97812 | 0,88514 |
| DHQ, 28 days  Control, 56 days | 0,00696 | 0,26346 | 0,03735 | 1 | 0,05 | 0 | -0,92467 | 0,93859 |
| DHQ, 28 days  DHQ, 1 day | 0,34794 | 0,26346 | 1,86767 | 0,96954 | 0,05 | 0 | -0,58369 | 1,27957 |
| DHQ, 28 days  DHQ, 7 days | 0,48408 | 0,26346 | 2,59842 | 0,78559 | 0,05 | 0 | -0,44755 | 1,4157 |
| DHQ, 28 days  DHQ, 14 days | 0,50116 | 0,26346 | 2,69011 | 0,74896 | 0,05 | 0 | -0,43047 | 1,43279 |
| DHQ, 42 days  Control, 1 day | -0,58667 | 0,26346 | 3,14911 | 0,54456 | 0,05 | 0 | -1,5183 | 0,34496 |
| DHQ, 42 days  Control, 7 days | -0,30987 | 0,26346 | 1,66335 | 0,98704 | 0,05 | 0 | -1,2415 | 0,62176 |
| DHQ, 42 days  Control, 14 days | -0,37284 | 0,26346 | 2,00135 | 0,9512 | 0,05 | 0 | -1,30447 | 0,55879 |
| DHQ, 42 days  Control, 28 days | -0,45948 | 0,25192 | 2,57943 | 0,79288 | 0,05 | 0 | -1,35029 | 0,43133 |
| DHQ, 42 days  Control, 42 days | -0,74704 | 0,28165 | 3,75098 | 0,29663 | 0,05 | 0 | -1,74299 | 0,24891 |
| DHQ, 42 days  Control, 56 days | -0,69359 | 0,28165 | 3,48262 | 0,39864 | 0,05 | 0 | -1,68955 | 0,30236 |
| DHQ, 42 days  DHQ, 1 day | -0,35261 | 0,28165 | 1,7705 | 0,97926 | 0,05 | 0 | -1,34856 | 0,64334 |
| DHQ, 42 days  DHQ, 7 days | -0,21648 | 0,28165 | 1,08695 | 0,99967 | 0,05 | 0 | -1,21243 | 0,77948 |
| DHQ, 42 days  DHQ, 14 days | -0,19939 | 0,28165 | 1,00118 | 0,99985 | 0,05 | 0 | -1,19535 | 0,79656 |
| DHQ, 42 days  DHQ, 28 days | -0,70055 | 0,26346 | 3,76042 | 0,29337 | 0,05 | 0 | -1,63218 | 0,23108 |
| DHQ, 56 days  Control, 1 day | -0,77894 | 0,26346 | 4,18123 | 0,17179 | 0,05 | 0 | -1,71057 | 0,15268 |
| DHQ, 56 days  Control, 7 days | -0,50215 | 0,26346 | 2,69546 | 0,74675 | 0,05 | 0 | -1,43378 | 0,42948 |
| DHQ, 56 days  Control, 14 days | -0,56512 | 0,26346 | 3,03347 | 0,59746 | 0,05 | 0 | -1,49675 | 0,36651 |
| DHQ, 56 days  Control, 28 days | -0,65176 | 0,25192 | 3,65885 | 0,32969 | 0,05 | 0 | -1,54257 | 0,23905 |
| DHQ, 56 days  Control, 42 days | -0,93932 | 0,28165 | 4,71644 | 0,07861 | 0,05 | 0 | -1,93527 | 0,05664 |
| DHQ, 56 days  Control, 56 days | -0,88587 | 0,28165 | 4,44807 | 0,1178 | 0,05 | 0 | -1,88183 | 0,11008 |
| DHQ, 56 days  DHQ, 1 day | -0,54489 | 0,28165 | 2,73596 | 0,72983 | 0,05 | 0 | -1,54084 | 0,45106 |
| DHQ, 56 days  DHQ, 7 days | -0,40875 | 0,28165 | 2,05241 | 0,9425 | 0,05 | 0 | -1,40471 | 0,5872 |
| DHQ, 56 days  DHQ, 14 days | -0,39167 | 0,28165 | 1,96664 | 0,95656 | 0,05 | 0 | -1,38763 | 0,60428 |
| DHQ, 56 days  DHQ, 28 days | -0,89283 | 0,26346 | 4,79254 | 0,0698 | 0,05 | 0 | -1,82446 | 0,0388 |
| DHQ, 56 days  DHQ, 42 days | -0,19228 | 0,28165 | 0,96546 | 0,99989 | 0,05 | 0 | -1,18823 | 0,80367 |

*Grouping Letters*

*Bonferroni Test*

| | Mean | Groups |
|---|---|---|
| Control, 42 days | 1,16037 | A |
| DHQ, 28 days | 1,11388 | A |
| Control, 56 days | 1,10693 | A |
| Control, 1 day | 1 | A |
| Control, 28 days | 0,87282 | A |
| Control, 14 days | 0,78618 | A |
| DHQ, 1 day | 0,76594 | A |
| Control, 7 days | 0,72321 | A |
| DHQ, 7 days | 0,62981 | A |
| DHQ, 14 days | 0,61273 | A |
| DHQ, 42 days | 0,41333 | A |
| DHQ, 56 days | 0,22106 | A |

Means that do not share a letter are significantly different.

*Scheffe Test*

| | Mean | Groups |
|---|---|---|
| Control, 42 days | 1,16037 | A |
| DHQ, 28 days | 1,11388 | A |
| Control, 56 days | 1,10693 | A |
| Control, 1 day | 1 | A |
| Control, 28 days | 0,87282 | A |
| Control, 14 days | 0,78618 | A |
| DHQ, 1 day | 0,76594 | A |
| Control, 7 days | 0,72321 | A |
| DHQ, 7 days | 0,62981 | A |
| DHQ, 14 days | 0,61273 | A |
| DHQ, 42 days | 0,41333 | A |
| DHQ, 56 days | 0,22106 | A |

Means that do not share a letter are significantly different.

*Tukey Test*

| | Mean | Groups |
|---|---|---|
| Control, 42 days | 1,16037 | A |
| DHQ, 28 days | 1,11388 | A |
| Control, 56 days | 1,10693 | A |
| Control, 1 day | 1 | A |
| Control, 28 days | 0,87282 | A |
| Control, 14 days | 0,78618 | A |
| DHQ, 1 day | 0,76594 | A |
| Control, 7 days | 0,72321 | A |
| DHQ, 7 days | 0,62981 | A |
| DHQ, 14 days | 0,61273 | A |
| DHQ, 42 days | 0,41333 | A |
| DHQ, 56 days | 0,22106 | A |

Means that do not share a letter are significantly different.

Sig equals 1 indicates that the difference of the means is significant at the 0,05 level.
Sig equals 0 indicates that the difference of the means is not significant at the 0,05 level.

*Powers*

| | Alpha | Sample Size | Power |
|---|---|---|---|
| Actual Power | 0,05 | 42 | 0,83853 |

*ANOVAOneWay (28.10.2024 12:53:36)*

*Notes*

| | |
|---|---|
| Description | Perform One-Way ANOVA |
| User Name | yulia |
| Operation Time | 28.10.2024 12:53:36 |
| Report Status | New Analysis Report |
| Data Filter | No |

*Input Data*

| | Data | Range |
|---|---|---|
| Control, 1 day, P1 | [Book1]"Subpopulations, % of cells" !A"Control, 1 day, P1" | [1*:3*] |
| Control, 7 days, P1 | [Book1]"Subpopulations, % of cells" !B"Control, 7 days, P1" | [1*:3*] |
| Control, 14 days, P1 | [Book1]"Subpopulations, % of cells" !C"Control, 14 days, P1" | [1*:3*] |
| Control, 28 days, P1 | [Book1]"Subpopulations, % of cells" !D"Control, 28 days, P1" | [1*:3*] |
| Control, 42 days, P1 | [Book1]"Subpopulations, % of cells" !E"Control, 42 days, P1" | [1*:3*] |
| Control, 56 days, P1 | [Book1]"Subpopulations, % of cells" !F"Control, 56 days, P1" | [1*:3*] |
| DHQ, 1 day, P1 | [Book1]"Subpopulations, % of cells" !G"DHQ, 1 day, P1" | [1*:3*] |
| DHQ, 7 days, P1 | [Book1]"Subpopulations, % of cells" !H"DHQ, 7 days, P1" | [1*:3*] |
| DHQ, 14 days, P1 | [Book1]"Subpopulations, % of cells" !I"DHQ, 14 days, P1" | [1*:3*] |
| DHQ, 28 days, P1 | [Book1]"Subpopulations, % of cells" !J"DHQ, 28 days, P1" | [1*:3*] |
| DHQ, 42 days, P1 | [Book1]"Subpopulations, % of cells" !K"DHQ, 42 days, P1" | [1*:3*] |
| DHQ, 56 days, P1 | [Book1]"Subpopulations, % of cells" !L"DHQ, 56 days, P1" | [1*:3*] |
| Control, 1 day, P2 | [Book1]"Subpopulations, % of cells" !M"Control, 1 day, P2" | [1*:3*] |
| Control, 7 days, P2 | [Book1]"Subpopulations, % of cells" !N"Control, 7 days, P2" | [1*:3*] |
| Control, 14 days, P2 | [Book1]"Subpopulations, % of cells" !O"Control, 14 days, P2" | [1*:3*] |
| Control, 28 days, P2 | [Book1]"Subpopulations, % of cells" !P"Control, 28 days, P2" | [1*:3*] |
| Control, 42 days, P2 | [Book1]"Subpopulations, % of cells" !Q"Control, 42 days, P2" | [1*:3*] |
| Control, 56 days, P2 | [Book1]"Subpopulations, % of cells" !R"Control, 56 days, P2" | [1*:3*] |
| DHQ, 1 day, P2 | [Book1]"Subpopulations, % of cells" !S"DHQ, 1 day, P2" | [1*:3*] |
| DHQ, 7 days, P2 | [Book1]"Subpopulations, % of cells" !T"DHQ, 7 days, P2" | [1*:3*] |
| DHQ, 14 days, P2 | [Book1]"Subpopulations, % of cells" !U"DHQ, 14 days, P2" | [1*:3*] |
| DHQ, 28 days, P2 | [Book1]"Subpopulations, % of cells" !V"DHQ, 28 days, P2" | [1*:3*] |
| DHQ, 42 days, P2 | [Book1]"Subpopulations, % of cells" !W"DHQ, 42 days, P2" | [1*:3*] |
| DHQ, 56 days, P2 | [Book1]"Subpopulations, % of cells" !X"DHQ, 56 days, P2" | [1*:3*] |

*Descriptive Statistics*

| | N Analysis | N Missing | Mean | Standard Deviation | SE of Mean |
|---|---|---|---|---|---|
| Control, 1 day, P1 | 3 | 0 | 0,27199 | 0,02576 | 0,01487 |
| Control, 7 days, P1 | 3 | 0 | 0,37249 | 0,08022 | 0,04631 |
| Control, 14 days, P1 | 3 | 0 | 0,30031 | 0,08471 | 0,04891 |
| Control, 28 days, P1 | 3 | 0 | 0,45363 | 0,06564 | 0,0379 |
| Control, 42 days, P1 | 3 | 0 | 0,51142 | 0,04353 | 0,02513 |
| Control, 56 days, P1 | 3 | 0 | 0,71377 | 0,10173 | 0,05874 |
| DHQ, 1 day, P1 | 3 | 0 | 0,33905 | 0,03485 | 0,02012 |
| DHQ, 7 days, P1 | 3 | 0 | 0,2176 | 0,0109 | 0,00629 |
| DHQ, 14 days, P1 | 3 | 0 | 0,23545 | 5,5E-4 | 3,17543E-4 |
| DHQ, 28 days, P1 | 3 | 0 | 0,46445 | 0,05455 | 0,03149 |
| DHQ, 42 days, P1 | 3 | 0 | 0,68625 | 0,15415 | 0,089 |
| DHQ, 56 days, P1 | 3 | 0 | 0,87855 | 0,08185 | 0,04726 |
| Control, 1 day, P2 | 3 | 0 | 0,71757 | 0,03293 | 0,01901 |
| Control, 7 days, P2 | 3 | 0 | 0,5896 | 0,078 | 0,04503 |
| Control, 14 days, P2 | 3 | 0 | 0,66443 | 0,09363 | 0,05406 |
| Control, 28 days, P2 | 3 | 0 | 0,52131 | 0,06119 | 0,03533 |
| Control, 42 days, P2 | 3 | 0 | 0,4683 | 0,03895 | 0,02249 |
| Control, 56 days, P2 | 3 | 0 | 0,26094 | 0,09914 | 0,05724 |
| DHQ, 1 day, P2 | 3 | 0 | 0,63725 | 0,03905 | 0,02255 |
| DHQ, 7 days, P2 | 3 | 0 | 0,6959 | 0,0142 | 0,0082 |
| DHQ, 14 days, P2 | 3 | 0 | 0,7108 | 0,0215 | 0,01241 |
| DHQ, 28 days, P2 | 3 | 0 | 0,5008 | 0,0523 | 0,0302 |
| DHQ, 42 days, P2 | 3 | 0 | 0,24555 | 0,09975 | 0,05759 |
| DHQ, 56 days, P2 | 3 | 0 | 0,09135 | 0,06865 | 0,03964 |

*ANOVA*

*Overall ANOVA*

| | DF | Sum of Squares | Mean Square | F Value | Prob>F |
|---|---|---|---|---|---|
| Model | 23 | 2,93073 | 0,12742 | 26,31551 | 6,71156E-20 |
| Error | 48 | 0,23242 | 0,00484 | | |
| Total | 71 | 3,16315 | | | |

Null Hypothesis: The means of all levels are equal.
Alternative Hypothesis: The means of one or more levels are different.
At the 0.05 level, the population means are significantly different.

*Fit Statistics*

| R-Square | Coeff Var | Root MSE | Data Mean |
|---|---|---|---|
| 0,92652 | 0,14461 | 0,06959 | 0,4812 |

*Means Comparisons*

*Bonferroni Test*

| | MeanDiff | SEM | t Value | Prob | Alpha | Sig | LCL | UCL |
|---|---|---|---|---|---|---|---|---|
| Control, 7 days, P1 Control, 1 day, P1 | 0,1005 | 0,05682 | 1,76886 | 1 | 0,05 | 0 | -0,13008 | 0,33108 |
| Control, 14 days, P1 Control, 1 day, P1 | 0,02832 | 0,05682 | 0,49849 | 1 | 0,05 | 0 | -0,20226 | 0,2589 |
| Control, 14 days, P1 Control, 7 days, P1 | -0,07218 | 0,05682 | -1,27037 | 1 | 0,05 | 0 | -0,30276 | 0,1584 |
| Control, 28 days, P1 Control, 1 day, P1 | 0,18164 | 0,05682 | 3,19705 | 0,67833 | 0,05 | 0 | -0,04894 | 0,41223 |
| Control, 28 days, P1 Control, 7 days, P1 | 0,08114 | 0,05682 | 1,42819 | 1 | 0,05 | 0 | -0,14944 | 0,31173 |
| Control, 28 days, P1 Control, 14 days, P1 | 0,15332 | 0,05682 | 2,69856 | 1 | 0,05 | 0 | -0,07726 | 0,3839 |
| Control, 42 days, P1 Control, 1 day, P1 | 0,23943 | 0,05682 | 4,21417 | 0,03034 | 0,05 | 1 | 0,00885 | 0,47001 |
| Control, 42 days, P1 Control, 7 days, P1 | 0,13893 | 0,05682 | 2,44531 | 1 | 0,05 | 0 | -0,09165 | 0,36951 |
| Control, 42 days, P1 Control, 14 days, P1 | 0,21111 | 0,05682 | 3,71568 | 0,14593 | 0,05 | 0 | -0,01947 | 0,44169 |
| Control, 42 days, P1 Control, 28 days, P1 | 0,05779 | 0,05682 | 1,01712 | 1 | 0,05 | 0 | -0,17279 | 0,28837 |
| Control, 56 days, P1 Control, 1 day, P1 | 0,44178 | 0,05682 | 7,77555 | 1,32926E-7 | 0,05 | 1 | 0,2112 | 0,67236 |
| Control, 56 days, P1 Control, 7 days, P1 | 0,34128 | 0,05682 | 6,00669 | 6,75928E-5 | 0,05 | 1 | 0,1107 | 0,57186 |
| Control, 56 days, P1 Control, 14 days, P1 | 0,41346 | 0,05682 | 7,27706 | 7,64135E-7 | 0,05 | 1 | 0,18287 | 0,64404 |
| Control, 56 days, P1 Control, 28 days, P1 | 0,26013 | 0,05682 | 4,5785 | 0,00919 | 0,05 | 1 | 0,02955 | 0,49071 |
| Control, 56 days, P1 Control, 42 days, P1 | 0,20234 | 0,05682 | 3,56138 | 0,23315 | 0,05 | 0 | -0,02824 | 0,43293 |
| DHQ, 1 day, P1 Control, 1 day, P1 | 0,06706 | 0,05682 | 1,18032 | 1 | 0,05 | 0 | -0,16352 | 0,29764 |
| DHQ, 1 day, P1 Control, 7 days, P1 | -0,03344 | 0,05682 | -0,58854 | 1 | 0,05 | 0 | -0,26402 | 0,19714 |
| DHQ, 1 day, P1 Control, 14 days, P1 | 0,03874 | 0,05682 | 0,68183 | 1 | 0,05 | 0 | -0,19184 | 0,26932 |
| DHQ, 1 day, P1 Control, 28 days, P1 | -0,11458 | 0,05682 | -2,01674 | 1 | 0,05 | 0 | -0,34516 | 0,116 |
| DHQ, 1 day, P1 Control, 42 days, P1 | -0,17237 | 0,05682 | -3,03385 | 1 | 0,05 | 0 | -0,40295 | 0,05821 |
| DHQ, 1 day, P1 Control, 56 days, P1 | -0,37472 | 0,05682 | -6,59524 | 8,47857E-6 | 0,05 | 1 | -0,6053 | -0,14414 |
| DHQ, 7 days, P1 Control, 1 day, P1 | -0,05439 | 0,05682 | -0,95728 | 1 | 0,05 | 0 | -0,28497 | 0,17619 |
| DHQ, 7 days, P1 Control, 7 days, P1 | -0,15489 | 0,05682 | -2,72614 | 1 | 0,05 | 0 | -0,38547 | 0,07569 |
| DHQ, 7 days, P1 Control, 14 days, P1 | -0,08271 | 0,05682 | -1,45577 | 1 | 0,05 | 0 | -0,31329 | 0,14787 |
| DHQ, 7 days, P1 Control, 28 days, P1 | -0,23603 | 0,05682 | -4,15433 | 0,03679 | 0,05 | 1 | -0,46661 | -0,00545 |
| DHQ, 7 days, P1 Control, 42 days, P1 | -0,29382 | 0,05682 | -5,17145 | 0,00124 | 0,05 | 1 | -0,5244 | -0,06324 |
| DHQ, 7 days, P1 Control, 56 days, P1 | -0,49617 | 0,05682 | -8,73283 | 4,85638E-9 | 0,05 | 1 | -0,72675 | -0,26559 |
| DHQ, 7 days, P1 DHQ, 1 day, P1 | -0,12145 | 0,05682 | -2,13759 | 1 | 0,05 | 0 | -0,35203 | 0,10913 |
| DHQ, 14 days, P1 Control, 1 day, P1 | -0,03654 | 0,05682 | -0,64311 | 1 | 0,05 | 0 | -0,26712 | 0,19404 |
| DHQ, 14 days, P1 Control, 7 days, P1 | -0,13704 | 0,05682 | -2,41197 | 1 | 0,05 | 0 | -0,36762 | 0,09354 |
| DHQ, 14 days, P1 Control, 14 days, P1 | -0,06486 | 0,05682 | -1,14159 | 1 | 0,05 | 0 | -0,29544 | 0,16572 |
| DHQ, 14 days, P1 Control, 28 days, P1 | -0,21818 | 0,05682 | -3,84016 | 0,09935 | 0,05 | 0 | -0,44876 | 0,0124 |
| DHQ, 14 days, P1 Control, 42 days, P1 | -0,27597 | 0,05682 | -4,85728 | 0,00361 | 0,05 | 1 | -0,50655 | -0,04539 |
| DHQ, 14 days, P1 Control, 56 days, P1 | -0,47832 | 0,05682 | -8,41866 | 1,42654E-8 | 0,05 | 1 | -0,7089 | -0,24774 |
| DHQ, 14 days, P1 DHQ, 1 day, P1 | -0,1036 | 0,05682 | -1,82342 | 1 | 0,05 | 0 | -0,33418 | 0,12698 |
| DHQ, 14 days, P1 DHQ, 7 days, P1 | 0,01785 | 0,05682 | 0,31417 | 1 | 0,05 | 0 | -0,21273 | 0,24843 |
| DHQ, 28 days, P1 Control, 1 day, P1 | 0,19246 | 0,05682 | 3,38743 | 0,39097 | 0,05 | 0 | -0,03812 | 0,42304 |
| DHQ, 28 days, P1 Control, 7 days, P1 | 0,09196 | 0,05682 | 1,61857 | 1 | 0,05 | 0 | -0,13862 | 0,32254 |
| DHQ, 28 days, P1 Control, 14 days, P1 | 0,16414 | 0,05682 | 2,88894 | 1 | 0,05 | 0 | -0,06644 | 0,39472 |
| DHQ, 28 days, P1 Control, 28 days, P1 | 0,01082 | 0,05682 | 0,19038 | 1 | 0,05 | 0 | -0,21976 | 0,2414 |
| DHQ, 28 days, P1 Control, 42 days, P1 | -0,04697 | 0,05682 | -0,82674 | 1 | 0,05 | 0 | -0,27755 | 0,18361 |
| DHQ, 28 days, P1 Control, 56 days, P1 | -0,24932 | 0,05682 | -4,38812 | 0,01723 | 0,05 | 1 | -0,4799 | -0,01874 |
| DHQ, 28 days, P1 DHQ, 1 day, P1 | 0,1254 | 0,05682 | 2,20711 | 1 | 0,05 | 0 | -0,10518 | 0,35598 |
| DHQ, 28 days, P1 DHQ, 7 days, P1 | 0,24685 | 0,05682 | 4,34471 | 0,01986 | 0,05 | 1 | 0,01627 | 0,47743 |
| DHQ, 28 days, P1 DHQ, 14 days, P1 | 0,229 | 0,05682 | 4,03054 | 0,05462 | 0,05 | 0 | -0,00158 | 0,45958 |
| DHQ, 42 days, P1 Control, 1 day, P1 | 0,41426 | 0,05682 | 7,29124 | 7,26931E-7 | 0,05 | 1 | 0,18368 | 0,64484 |
| DHQ, 42 days, P1 Control, 7 days, P1 | 0,31376 | 0,05682 | 5,52238 | 3,68004E-4 | 0,05 | 1 | 0,08318 | 0,54434 |
| DHQ, 42 days, P1 Control, 14 days, P1 | 0,38594 | 0,05682 | 6,79276 | 4,21917E-6 | 0,05 | 1 | 0,15536 | 0,61652 |
| DHQ, 42 days, P1 Control, 28 days, P1 | 0,23262 | 0,05682 | 4,09419 | 0,0446 | 0,05 | 1 | 0,00204 | 0,4632 |
| DHQ, 42 days, P1 Control, 42 days, P1 | 0,17483 | 0,05682 | 3,07707 | 0,95184 | 0,05 | 0 | -0,05575 | 0,40541 |
| DHQ, 42 days, P1 Control, 56 days, P1 | -0,02752 | 0,05682 | -0,48431 | 1 | 0,05 | 0 | -0,2581 | 0,20306 |
| DHQ, 42 days, P1 DHQ, 1 day, P1 | 0,3472 | 0,05682 | 6,11093 | 4,68384E-5 | 0,05 | 1 | 0,11662 | 0,57778 |
| DHQ, 42 days, P1 DHQ, 7 days, P1 | 0,46865 | 0,05682 | 8,24852 | 2,56645E-8 | 0,05 | 1 | 0,23807 | 0,69923 |
| DHQ, 42 days, P1 DHQ, 14 days, P1 | 0,4508 | 0,05682 | 7,93435 | 7,63831E-8 | 0,05 | 1 | 0,22022 | 0,68138 |
| DHQ, 42 days, P1 DHQ, 28 days, P1 | 0,2218 | 0,05682 | 3,90381 | 0,08145 | 0,05 | 0 | -0,00878 | 0,45238 |
| DHQ, 56 days, P1 Control, 1 day, P1 | 0,60656 | 0,05682 | 10,67584 | 7,86742E-1 | 0,05 | 1 | 0,37598 | 0,83714 |
| DHQ, 56 days, P1 Control, 7 days, P1 | 0,50606 | 0,05682 | 8,90698 | 2,6835E-9 | 0,05 | 1 | 0,27548 | 0,73664 |
| DHQ, 56 days, P1 Control, 14 days, P1 | 0,57824 | 0,05682 | 10,17735 | 3,91923E-1 | 0,05 | 1 | 0,34766 | 0,80882 |
| DHQ, 56 days, P1 Control, 28 days, P1 | 0,42492 | 0,05682 | 7,47879 | 3,75942E-7 | 0,05 | 1 | 0,19434 | 0,6555 |
| DHQ, 56 days, P1 Control, 42 days, P1 | 0,36713 | 0,05682 | 6,46167 | 1,35911E-5 | 0,05 | 1 | 0,13655 | 0,59771 |
| DHQ, 56 days, P1 Control, 56 days, P1 | 0,16478 | 0,05682 | 2,90029 | 1 | 0,05 | 0 | -0,0658 | 0,39536 |
| DHQ, 56 days, P1 DHQ, 1 day, P1 | 0,5395 | 0,05682 | 9,49552 | 3,70148E-1 | 0,05 | 1 | 0,30892 | 0,77008 |
| DHQ, 56 days, P1 DHQ, 7 days, P1 | 0,66095 | 0,05682 | 11,63312 | 3,9362E-13 | 0,05 | 1 | 0,43037 | 0,89153 |
| DHQ, 56 days, P1 DHQ, 14 days, P1 | 0,6431 | 0,05682 | 11,31894 | 1,03833E-1 | 0,05 | 1 | 0,41252 | 0,87368 |
| DHQ, 56 days, P1 DHQ, 28 days, P1 | 0,4141 | 0,05682 | 7,28841 | 7,34223E-7 | 0,05 | 1 | 0,18352 | 0,64468 |
| DHQ, 56 days, P1 DHQ, 42 days, P1 | 0,1923 | 0,05682 | 3,38459 | 0,39424 | 0,05 | 0 | -0,03828 | 0,42288 |
| Control, 1 day, P2 Control, 1 day, P1 | 0,44558 | 0,05682 | 7,84244 | 1,05239E-7 | 0,05 | 1 | 0,215 | 0,67616 |
| Control, 1 day, P2 Control, 7 days, P1 | 0,34508 | 0,05682 | 6,07358 | 5,34212E-5 | 0,05 | 1 | 0,1145 | 0,57566 |
| Control, 1 day, P2 Control, 14 days, P1 | 0,41726 | 0,05682 | 7,34395 | 6,03875E-7 | 0,05 | 1 | 0,18667 | 0,64784 |
| Control, 1 day, P2 Control, 28 days, P1 | 0,26393 | 0,05682 | 4,64538 | 0,00736 | 0,05 | 1 | 0,03335 | 0,49451 |
| Control, 1 day, P2 Control, 42 days, P1 | 0,20614 | 0,05682 | 3,62827 | 0,19051 | 0,05 | 0 | -0,02444 | 0,43673 |
| Control, 1 day, P2 Control, 56 days, P1 | 0,0038 | 0,05682 | 0,06688 | 1 | 0,05 | 0 | -0,22678 | 0,23438 |
| Control, 1 day, P2 DHQ, 1 day, P1 | 0,37852 | 0,05682 | 6,66212 | 6,69402E-6 | 0,05 | 1 | 0,14794 | 0,6091 |
| Control, 1 day, P2 DHQ, 7 days, P1 | 0,49997 | 0,05682 | 8,79971 | 3,86562E-9 | 0,05 | 1 | 0,26939 | 0,73055 |
| Control, 1 day, P2 DHQ, 14 days, P1 | 0,48212 | 0,05682 | 8,48554 | 1,13325E-8 | 0,05 | 1 | 0,25154 | 0,7127 |
| Control, 1 day, P2 DHQ, 28 days, P1 | 0,25312 | 0,05682 | 4,455 | 0,01383 | 0,05 | 1 | 0,02254 | 0,4837 |
| Control, 1 day, P2 DHQ, 42 days, P1 | 0,03132 | 0,05682 | 0,55119 | 1 | 0,05 | 0 | -0,19926 | 0,2619 |
| Control, 1 day, P2 DHQ, 56 days, P1 | -0,16098 | 0,05682 | -2,8334 | 1 | 0,05 | 0 | -0,39156 | 0,0696 |
| Control, 7 days, P2 Control, 1 day, P1 | 0,31761 | 0,05682 | 5,59015 | 2,90674E-4 | 0,05 | 1 | 0,08703 | 0,54819 |
| Control, 7 days, P2 Control, 7 days, P1 | 0,21711 | 0,05682 | 3,82129 | 0,10535 | 0,05 | 0 | -0,01347 | 0,44769 |
| Control, 7 days, P2 Control, 14 days, P1 | 0,28929 | 0,05682 | 5,09166 | 0,00163 | 0,05 | 1 | 0,05871 | 0,51987 |
| Control, 7 days, P2 Control, 28 days, P1 | 0,13597 | 0,05682 | 2,39309 | 1 | 0,05 | 0 | -0,09461 | 0,36655 |
| Control, 7 days, P2 Control, 42 days, P1 | 0,07818 | 0,05682 | 1,37598 | 1 | 0,05 | 0 | -0,1524 | 0,30876 |
| Control, 7 days, P2 Control, 56 days, P1 | -0,12417 | 0,05682 | -2,18541 | 1 | 0,05 | 0 | -0,35475 | 0,10641 |
| Control, 7 days, P2 DHQ, 1 day, P1 | 0,25055 | 0,05682 | 4,40983 | 0,01605 | 0,05 | 1 | 0,01997 | 0,48113 |
| Control, 7 days, P2 DHQ, 7 days, P1 | 0,372 | 0,05682 | 6,54742 | 1,00391E-5 | 0,05 | 1 | 0,14142 | 0,60258 |
| Control, 7 days, P2 DHQ, 14 days, P1 | 0,35415 | 0,05682 | 6,23325 | 3,04361E-5 | 0,05 | 1 | 0,12357 | 0,58473 |
| Control, 7 days, P2 DHQ, 28 days, P1 | 0,12515 | 0,05682 | 2,20271 | 1 | 0,05 | 0 | -0,10543 | 0,35573 |
| Control, 7 days, P2 DHQ, 42 days, P1 | -0,09665 | 0,05682 | -1,7011 | 1 | 0,05 | 0 | -0,32723 | 0,13393 |
| Control, 7 days, P2 DHQ, 56 days, P1 | -0,28895 | 0,05682 | -5,08569 | 0,00166 | 0,05 | 1 | -0,51953 | -0,05837 |
| Control, 7 days, P2 Control, 1 day, P2 | -0,12797 | 0,05682 | -2,25229 | 1 | 0,05 | 0 | -0,35855 | 0,10261 |
| Control, 14 days, P2 Control, 1 day, P1 | 0,39244 | 0,05682 | 6,90726 | 2,81566E-6 | 0,05 | 1 | 0,16186 | 0,62303 |
| Control, 14 days, P2 Control, 7 days, P1 | 0,29194 | 0,05682 | 5,1384 | 0,00139 | 0,05 | 1 | 0,06136 | 0,52253 |
| Control, 14 days, P2 Control, 14 days, P1 | 0,36412 | 0,05682 | 6,40877 | 1,63829E-5 | 0,05 | 1 | 0,13354 | 0,5947 |
| Control, 14 days, P2 Control, 28 days, P1 | 0,2108 | 0,05682 | 3,71021 | 0,1484 | 0,05 | 0 | -0,01978 | 0,44138 |
| Control, 14 days, P2 Control, 42 days, P1 | 0,15301 | 0,05682 | 2,69309 | 1 | 0,05 | 0 | -0,07757 | 0,38359 |
| Control, 14 days, P2 Control, 56 days, P1 | -0,04933 | 0,05682 | -0,8683 | 1 | 0,05 | 0 | -0,27991 | 0,18125 |
| Control, 14 days, P2 DHQ, 1 day, P1 | 0,32538 | 0,05682 | 5,72694 | 1,80307E-4 | 0,05 | 1 | 0,0948 | 0,55596 |
| Control, 14 days, P2 DHQ, 7 days, P1 | 0,44683 | 0,05682 | 7,86453 | 9,74298E-8 | 0,05 | 1 | 0,21625 | 0,67741 |
| Control, 14 days, P2 DHQ, 14 days, P1 | 0,42898 | 0,05682 | 7,55036 | 2,92431E-7 | 0,05 | 1 | 0,1984 | 0,65956 |
| Control, 14 days, P2 DHQ, 28 days, P1 | 0,19998 | 0,05682 | 3,51983 | 0,2641 | 0,05 | 0 | -0,0306 | 0,43056 |
| Control, 14 days, P2 DHQ, 42 days, P1 | -0,02182 | 0,05682 | -0,38399 | 1 | 0,05 | 0 | -0,2524 | 0,20876 |
| Control, 14 days, P2 DHQ, 56 days, P1 | -0,21412 | 0,05682 | -3,76858 | 0,12402 | 0,05 | 0 | -0,4447 | 0,01646 |
| Control, 14 days, P2 Control, 1 day, P2 | -0,05313 | 0,05682 | -0,93518 | 1 | 0,05 | 0 | -0,28371 | 0,17745 |
| Control, 14 days, P2 Control, 7 days, P2 | 0,07483 | 0,05682 | 1,31711 | 1 | 0,05 | 0 | -0,15575 | 0,30541 |
| Control, 28 days, P2 Control, 1 day, P1 | 0,24932 | 0,05682 | 4,38822 | 0,01723 | 0,05 | 1 | 0,01874 | 0,4799 |
| Control, 28 days, P2 Control, 7 days, P1 | 0,14882 | 0,05682 | 2,61936 | 1 | 0,05 | 0 | -0,08176 | 0,3794 |
| Control, 28 days, P2 Control, 14 days, P1 | 0,221 | 0,05682 | 3,88973 | 0,08512 | 0,05 | 0 | -0,00958 | 0,45158 |
| Control, 28 days, P2 Control, 28 days, P1 | 0,06768 | 0,05682 | 1,19117 | 1 | 0,05 | 0 | -0,1629 | 0,29826 |
| Control, 28 days, P2 Control, 42 days, P1 | 0,00989 | 0,05682 | 0,17405 | 1 | 0,05 | 0 | -0,22069 | 0,24047 |
| Control, 28 days, P2 Control, 56 days, P1 | -0,19246 | 0,05682 | -3,38733 | 0,39109 | 0,05 | 0 | -0,42304 | 0,03813 |
| Control, 28 days, P2 DHQ, 1 day, P1 | 0,18226 | 0,05682 | 3,2079 | 0,65764 | 0,05 | 0 | -0,04832 | 0,41284 |
| Control, 28 days, P2 DHQ, 7 days, P1 | 0,30371 | 0,05682 | 5,3455 | 6,79582E-4 | 0,05 | 1 | 0,07313 | 0,53429 |
| Control, 28 days, P2 DHQ, 14 days, P1 | 0,28586 | 0,05682 | 5,03133 | 0,002 | 0,05 | 1 | 0,05528 | 0,51644 |
| Control, 28 days, P2 DHQ, 28 days, P1 | 0,05686 | 0,05682 | 1,00079 | 1 | 0,05 | 0 | -0,17372 | 0,28744 |
| Control, 28 days, P2 DHQ, 42 days, P1 | -0,16494 | 0,05682 | -2,90302 | 1 | 0,05 | 0 | -0,39552 | 0,06564 |
| Control, 28 days, P2 DHQ, 56 days, P1 | -0,35724 | 0,05682 | -6,28762 | 2,5125E-5 | 0,05 | 1 | -0,58782 | -0,12666 |
| Control, 28 days, P2 Control, 1 day, P2 | -0,19626 | 0,05682 | -3,45422 | 0,32107 | 0,05 | 0 | -0,42684 | 0,03433 |
| Control, 28 days, P2 Control, 7 days, P2 | -0,06829 | 0,05682 | -1,20193 | 1 | 0,05 | 0 | -0,29887 | 0,16229 |
| Control, 28 days, P2 Control, 14 days, P2 | -0,14312 | 0,05682 | -2,51904 | 1 | 0,05 | 0 | -0,3737 | 0,08746 |
| Control, 42 days, P2 Control, 1 day, P1 | 0,19631 | 0,05682 | 3,45519 | 0,32014 | 0,05 | 0 | -0,03427 | 0,42689 |
| Control, 42 days, P2 Control, 7 days, P1 | 0,09581 | 0,05682 | 1,68633 | 1 | 0,05 | 0 | -0,13477 | 0,32639 |
| Control, 42 days, P2 Control, 14 days, P1 | 0,16799 | 0,05682 | 2,9567 | 1 | 0,05 | 0 | -0,06259 | 0,39857 |
| Control, 42 days, P2 Control, 28 days, P1 | 0,01467 | 0,05682 | 0,25814 | 1 | 0,05 | 0 | -0,21591 | 0,24525 |
| Control, 42 days, P2 Control, 42 days, P1 | -0,04312 | 0,05682 | -0,75898 | 1 | 0,05 | 0 | -0,2737 | 0,18746 |
| Control, 42 days, P2 Control, 56 days, P1 | -0,24547 | 0,05682 | -4,32036 | 0,0215 | 0,05 | 1 | -0,47605 | -0,01489 |
| Control, 42 days, P2 DHQ, 1 day, P1 | 0,12925 | 0,05682 | 2,27488 | 1 | 0,05 | 0 | -0,10133 | 0,35983 |
| Control, 42 days, P2 DHQ, 7 days, P1 | 0,2507 | 0,05682 | 4,41247 | 0,01591 | 0,05 | 1 | 0,02012 | 0,48128 |
| Control, 42 days, P2 DHQ, 14 days, P1 | 0,23285 | 0,05682 | 4,0983 | 0,04402 | 0,05 | 1 | 0,00227 | 0,46343 |
| Control, 42 days, P2 DHQ, 28 days, P1 | 0,00385 | 0,05682 | 0,06776 | 1 | 0,05 | 0 | -0,22673 | 0,23443 |
| Control, 42 days, P2 DHQ, 42 days, P1 | -0,21795 | 0,05682 | -3,83605 | 0,10063 | 0,05 | 0 | -0,44853 | 0,01263 |
| Control, 42 days, P2 DHQ, 56 days, P1 | -0,41025 | 0,05682 | -7,22065 | 9,32101E-7 | 0,05 | 1 | -0,64083 | -0,17967 |
| Control, 42 days, P2 Control, 1 day, P2 | -0,24927 | 0,05682 | -4,38724 | 0,01728 | 0,05 | 1 | -0,47985 | -0,01869 |
| Control, 42 days, P2 Control, 7 days, P2 | -0,1213 | 0,05682 | -2,13495 | 1 | 0,05 | 0 | -0,35188 | 0,10928 |
| Control, 42 days, P2 Control, 14 days, P2 | -0,19613 | 0,05682 | -3,45206 | 0,32312 | 0,05 | 0 | -0,42671 | 0,03445 |
| Control, 42 days, P2 Control, 28 days, P2 | -0,05301 | 0,05682 | -0,93303 | 1 | 0,05 | 0 | -0,28359 | 0,17757 |
| Control, 56 days, P2 Control, 1 day, P1 | -0,01104 | 0,05682 | -0,19439 | 1 | 0,05 | 0 | -0,24163 | 0,21954 |
| Control, 56 days, P2 Control, 7 days, P1 | -0,11154 | 0,05682 | -1,96325 | 1 | 0,05 | 0 | -0,34213 | 0,11904 |
| Control, 56 days, P2 Control, 14 days, P1 | -0,03937 | 0,05682 | -0,69288 | 1 | 0,05 | 0 | -0,26995 | 0,19121 |
| Control, 56 days, P2 Control, 28 days, P1 | -0,19269 | 0,05682 | -3,39144 | 0,3864 | 0,05 | 0 | -0,42327 | 0,03789 |
| Control, 56 days, P2 Control, 42 days, P1 | -0,25048 | 0,05682 | -4,40856 | 0,01612 | 0,05 | 1 | -0,48106 | -0,0199 |
| Control, 56 days, P2 Control, 56 days, P1 | -0,45282 | 0,05682 | -7,96994 | 6,74795E-8 | 0,05 | 1 | -0,6834 | -0,22224 |
| Control, 56 days, P2 DHQ, 1 day, P1 | -0,07811 | 0,05682 | -1,3747 | 1 | 0,05 | 0 | -0,30869 | 0,15248 |
| Control, 56 days, P2 DHQ, 7 days, P1 | 0,04334 | 0,05682 | 0,76289 | 1 | 0,05 | 0 | -0,18724 | 0,27393 |
| Control, 56 days, P2 DHQ, 14 days, P1 | 0,02549 | 0,05682 | 0,44872 | 1 | 0,05 | 0 | -0,20509 | 0,25608 |
| Control, 56 days, P2 DHQ, 28 days, P1 | -0,20351 | 0,05682 | -3,58182 | 0,21924 | 0,05 | 0 | -0,43409 | 0,02708 |
| Control, 56 days, P2 DHQ, 42 days, P1 | -0,42531 | 0,05682 | -7,48563 | 3,67015E-7 | 0,05 | 1 | -0,65589 | -0,19472 |
| Control, 56 days, P2 DHQ, 56 days, P1 | -0,61761 | 0,05682 | -10,8702 | 4,242E-12 | 0,05 | 1 | -0,84819 | -0,38702 |
| Control, 56 days, P2 Control, 1 day, P2 | -0,45662 | 0,05682 | -8,03682 | 5,34733E-8 | 0,05 | 1 | -0,6872 | -0,22604 |
| Control, 56 days, P2 Control, 7 days, P2 | -0,32866 | 0,05682 | -5,78453 | 1,47397E-4 | 0,05 | 1 | -0,55924 | -0,09807 |
| Control, 56 days, P2 Control, 14 days, P2 | -0,40349 | 0,05682 | -7,10165 | 1,41785E-6 | 0,05 | 1 | -0,63407 | -0,17291 |
| Control, 56 days, P2 Control, 28 days, P2 | -0,26037 | 0,05682 | -4,58261 | 0,00907 | 0,05 | 1 | -0,49095 | -0,02979 |
| Control, 56 days, P2 Control, 42 days, P2 | -0,20736 | 0,05682 | -3,64958 | 0,17857 | 0,05 | 0 | -0,43794 | 0,02323 |
| DHQ, 1 day, P2 Control, 1 day, P1 | 0,36526 | 0,05682 | 6,42881 | 1,52633E-5 | 0,05 | 1 | 0,13468 | 0,59584 |
| [illegible] | [illegible] | [illegible] | [illegible] | [illegible] | [illegible] | [illegible] | [illegible] | [illegible] |

[illegible]

## ANOVAOneWay (28.10.2024 12:37:37)

### Notes

| | |
|---|---|
| Description | Perform One-Way ANOVA |
| User Name | yulia |
| Operation Time | 28.10.2024 12:37:37 |
| Report Status | New Analysis Report |
| Data Filter | No |

### Input Data

| | Data | Range |
|---|---|---|
| Control, 1 day | [Book1]"% inhibition of respiration"! A"Control, 1 day" | [1*:7*] |
| Control, 7 days | [Book1]"% inhibition of respiration"! B"Control, 7 days" | [1*:6*] |
| Control, 14 days | [Book1]"% inhibition of respiration"! C"Control, 14 days" | [1*:6*] |
| Control, 28 days | [Book1]"% inhibition of respiration"! D"Control, 28 days" | [1*:8*] |
| Control, 42 days | [Book1]"% inhibition of respiration"! E"Control, 42 days" | [1*:5*] |
| Control, 56 days | [Book1]"% inhibition of respiration"! F"Control, 56 days" | [1*:4*] |
| DHQ, 1 day | [Book1]"% inhibition of respiration"! G"DHQ, 1 day" | [1*:6*] |
| DHQ, 7 days | [Book1]"% inhibition of respiration"! H"DHQ, 7 days" | [1*:6*] |
| DHQ, 14 days | [Book1]"% inhibition of respiration"!I "DHQ, 14 days" | [1*:7*] |
| DHQ, 28 days | [Book1]"% inhibition of respiration"!J "DHQ, 28 days" | [1*:8*] |
| DHQ, 42 days | [Book1]"% inhibition of respiration"! K"DHQ, 42 days" | [1*:5*] |
| DHQ, 56 days | [Book1]"% inhibition of respiration"! L"DHQ, 56 days" | [1*:4*] |

### Descriptive Statistics

| | N Analysis | N Missing | Mean | Standard Deviation | SE of Mean |
|---|---|---|---|---|---|
| Control, 1 day | 7 | 0 | 39,98467 | 8,5647 | 3,23715 |
| Control, 7 days | 6 | 0 | 12,94466 | 10,93711 | 4,46505 |
| Control, 14 days | 6 | 0 | 14,09797 | 9,53033 | 3,89074 |
| Control, 28 days | 8 | 0 | 26,88037 | 12,76577 | 4,51338 |
| Control, 42 days | 5 | 0 | 31,42821 | 5,27173 | 2,35759 |
| Control, 56 days | 4 | 0 | 13,74486 | 17,00858 | 8,50429 |
| DHQ, 1 day | 6 | 0 | 43,63224 | 7,48808 | 3,057 |
| DHQ, 7 days | 6 | 0 | 27,57032 | 11,65942 | 4,75994 |
| DHQ, 14 days | 7 | 0 | 24,58737 | 12,23922 | 4,62599 |
| DHQ, 28 days | 8 | 0 | 28,27625 | 11,86616 | 4,19532 |
| DHQ, 42 days | 5 | 0 | 14,07202 | 9,80236 | 4,38375 |
| DHQ, 56 days | 4 | 0 | 23,61355 | 21,44705 | 10,72353 |

### ANOVA

#### Overall ANOVA

| | DF | Sum of Squares | Mean Square | F Value | Prob>F |
|---|---|---|---|---|---|
| Model | 11 | 6663,52049 | 605,77459 | 4,42121 | 7,48789E-5 |
| Error | 60 | 8220,92642 | 137,01544 | | |
| Total | 71 | 14884,44691 | | | |

Null Hypothesis: The means of all levels are equal.
Alternative Hypothesis: The means of one or more levels are different.
At the 0.05 level, the population means are significantly different.

#### Fit Statistics

| | R-Square | Coeff Var | Root MSE | Data Mean |
|---|---|---|---|---|
| | 0,44768 | 0,45319 | 11,70536 | 25,82866 |

### Means Comparisons

#### Bonferroni Test

| | | MeanDiff | SEM | t Value | Prob | Alpha | Sig | LCL | UCL |
|---|---|---|---|---|---|---|---|---|---|
| Control, 7 days | Control, 1 day | -27,04001 | 6,51226 | -4,15217 | 0,00698 | 0,05 | 1 | -50,15464 | -3,92538 |
| Control, 14 days | Control, 1 day | -25,8867 | 6,51226 | -3,97507 | 0,01264 | 0,05 | 1 | -49,00133 | -2,77207 |
| Control, 14 days | Control, 7 days | 1,15331 | 6,75809 | 0,17066 | 1 | 0,05 | 0 | -22,83388 | 25,1405 |
| Control, 28 days | Control, 1 day | -13,10431 | 6,0581 | -2,16311 | 1 | 0,05 | 0 | -34,60694 | 8,39832 |
| Control, 28 days | Control, 7 days | 13,9357 | 6,32162 | 2,20445 | 1 | 0,05 | 0 | -8,50226 | 36,37366 |
| Control, 28 days | Control, 14 da | 12,7824 | 6,32162 | 2,02201 | 1 | 0,05 | 0 | -9,65556 | 35,22035 |
| Control, 42 days | Control, 1 day | -8,55646 | 6,85396 | -1,2484 | 1 | 0,05 | 0 | -32,88391 | 15,77099 |
| Control, 42 days | Control, 7 days | 18,48355 | 7,08795 | 2,60774 | 0,75795 | 0,05 | 0 | -6,67443 | 43,64152 |
| Control, 42 days | Control, 14 da | 17,33024 | 7,08795 | 2,44503 | 1 | 0,05 | 0 | -7,82773 | 42,48822 |
| Control, 42 days | Control, 28 da | 4,54785 | 6,67308 | 0,68152 | 1 | 0,05 | 0 | -19,1376 | 28,2333 |
| Control, 56 days | Control, 1 day | -26,23982 | 7,33672 | -3,5765 | 0,04592 | 0,05 | 1 | -52,28079 | -0,19884 |
| Control, 56 days | Control, 7 days | 0,80019 | 7,55578 | 0,1059 | 1 | 0,05 | 0 | -26,0183 | 27,61868 |
| Control, 56 days | Control, 14 da | -0,35312 | 7,55578 | -0,04673 | 1 | 0,05 | 0 | -27,17161 | 26,46538 |
| Control, 56 days | Control, 28 da | -13,13551 | 7,16804 | -1,83251 | 1 | 0,05 | 0 | -38,57777 | 12,30674 |
| Control, 56 days | Control, 42 da | -17,68336 | 7,85219 | -2,25203 | 1 | 0,05 | 0 | -45,55395 | 10,18724 |
| DHQ, 1 day | Control, 1 day | 3,64757 | 6,51226 | 0,56011 | 1 | 0,05 | 0 | -19,46706 | 26,7622 |
| DHQ, 1 day | Control, 7 days | 30,68758 | 6,75809 | 4,54086 | 0,00182 | 0,05 | 1 | 6,70039 | 54,67477 |
| DHQ, 1 day | Control, 14 days | 29,53427 | 6,75809 | 4,37021 | 0,0033 | 0,05 | 1 | 5,54708 | 53,52146 |
| DHQ, 1 day | Control, 28 days | 16,75188 | 6,32162 | 2,64994 | 0,67833 | 0,05 | 0 | -5,68608 | 39,18984 |
| DHQ, 1 day | Control, 42 days | 12,20403 | 7,08795 | 1,7218 | 1 | 0,05 | 0 | -12,95394 | 37,36201 |
| DHQ, 1 day | Control, 56 days | 29,88739 | 7,55578 | 3,95557 | 0,01349 | 0,05 | 1 | 3,0689 | 56,70588 |
| DHQ, 7 days | Control, 1 day | -12,41435 | 6,51226 | -1,9063 | 1 | 0,05 | 0 | -35,52898 | 10,70028 |
| DHQ, 7 days | Control, 7 days | 14,62566 | 6,75809 | 2,16417 | 1 | 0,05 | 0 | -9,36153 | 38,61285 |
| DHQ, 7 days | Control, 14 days | 13,47235 | 6,75809 | 1,99351 | 1 | 0,05 | 0 | -10,51484 | 37,45954 |
| DHQ, 7 days | Control, 28 days | 0,68996 | 6,32162 | 0,10914 | 1 | 0,05 | 0 | -21,748 | 23,12792 |
| DHQ, 7 days | Control, 42 days | -3,85789 | 7,08795 | -0,54429 | 1 | 0,05 | 0 | -29,01586 | 21,30009 |
| DHQ, 7 days | Control, 56 days | 13,82547 | 7,55578 | 1,82979 | 1 | 0,05 | 0 | -12,99302 | 40,64396 |
| DHQ, 7 days | DHQ, 1 day | -16,06192 | 6,75809 | -2,37669 | 1 | 0,05 | 0 | -40,04911 | 7,92527 |
| DHQ, 14 days | Control, 1 day | -15,3973 | 6,25678 | -2,4609 | 1 | 0,05 | 0 | -37,60512 | 6,81052 |
| DHQ, 14 days | Control, 7 days | 11,64271 | 6,51226 | 1,78781 | 1 | 0,05 | 0 | -11,47192 | 34,75734 |
| DHQ, 14 days | Control, 14 days | 10,4894 | 6,51226 | 1,61072 | 1 | 0,05 | 0 | -12,62523 | 33,60404 |
| DHQ, 14 days | Control, 28 days | -2,29299 | 6,0581 | -0,3785 | 1 | 0,05 | 0 | -23,79562 | 19,20964 |
| DHQ, 14 days | Control, 42 days | -6,84084 | 6,85396 | -0,99809 | 1 | 0,05 | 0 | -31,16829 | 17,48661 |
| DHQ, 14 days | Control, 56 days | 10,84252 | 7,33672 | 1,47784 | 1 | 0,05 | 0 | -15,19846 | 36,8835 |
| DHQ, 14 days | DHQ, 1 day | -19,04487 | 6,51226 | -2,92446 | 0,32096 | 0,05 | 0 | -42,1595 | 4,06976 |
| DHQ, 14 days | DHQ, 7 days | -2,98295 | 6,51226 | -0,45805 | 1 | 0,05 | 0 | -26,09758 | 20,13168 |
| DHQ, 28 days | Control, 1 day | -11,70843 | 6,0581 | -1,93269 | 1 | 0,05 | 0 | -33,21106 | 9,7942 |
| DHQ, 28 days | Control, 7 days | 15,33158 | 6,32162 | 2,42526 | 1 | 0,05 | 0 | -7,10638 | 37,76954 |
| DHQ, 28 days | Control, 14 days | 14,17827 | 6,32162 | 2,24282 | 1 | 0,05 | 0 | -8,25969 | 36,61623 |
| DHQ, 28 days | Control, 28 days | 1,39588 | 5,85268 | 0,2385 | 1 | 0,05 | 0 | -19,37764 | 22,16939 |
| DHQ, 28 days | Control, 42 days | -3,15197 | 6,67308 | -0,47234 | 1 | 0,05 | 0 | -26,83742 | 20,53348 |
| DHQ, 28 days | Control, 56 days | 14,53139 | 7,16804 | 2,02725 | 1 | 0,05 | 0 | -10,91087 | 39,97364 |
| DHQ, 28 days | DHQ, 1 day | -15,356 | 6,32162 | -2,42913 | 1 | 0,05 | 0 | -37,79396 | 7,08196 |
| DHQ, 28 days | DHQ, 7 days | 0,70592 | 6,32162 | 0,11167 | 1 | 0,05 | 0 | -21,73204 | 23,14388 |
| DHQ, 28 days | DHQ, 14 days | 3,68887 | 6,0581 | 0,60892 | 1 | 0,05 | 0 | -17,81376 | 25,1915 |
| DHQ, 42 days | Control, 1 day | -25,91265 | 6,85396 | -3,78068 | 0,02392 | 0,05 | 1 | -50,2401 | -1,5852 |
| DHQ, 42 days | Control, 7 days | 1,12736 | 7,08795 | 0,15905 | 1 | 0,05 | 0 | -24,03061 | 26,28533 |
| DHQ, 42 days | Control, 14 days | -0,02595 | 7,08795 | -0,00366 | 1 | 0,05 | 0 | -25,18392 | 25,13203 |
| DHQ, 42 days | Control, 28 days | -12,80834 | 6,67308 | -1,9194 | 1 | 0,05 | 0 | -36,49379 | 10,87711 |
| DHQ, 42 days | Control, 42 days | -17,35619 | 7,40312 | -2,34444 | 1 | 0,05 | 0 | -43,63284 | 8,92046 |
| DHQ, 42 days | Control, 56 days | 0,32717 | 7,85219 | 0,04167 | 1 | 0,05 | 0 | -27,54343 | 28,19776 |
| DHQ, 42 days | DHQ, 1 day | -29,56022 | 7,08795 | -4,17049 | 0,00656 | 0,05 | 1 | -54,7182 | -4,40225 |
| DHQ, 42 days | DHQ, 7 days | -13,4983 | 7,08795 | -1,9044 | 1 | 0,05 | 0 | -38,65628 | 11,65967 |
| DHQ, 42 days | DHQ, 14 days | -10,51535 | 6,85396 | -1,5342 | 1 | 0,05 | 0 | -34,8428 | 13,8121 |
| DHQ, 42 days | DHQ, 28 days | -14,20422 | 6,67308 | -2,12859 | 1 | 0,05 | 0 | -37,88967 | 9,48123 |
| DHQ, 56 days | Control, 1 day | -16,37112 | 7,33672 | -2,23139 | 1 | 0,05 | 0 | -42,4121 | 9,66986 |
| DHQ, 56 days | Control, 7 days | 10,66889 | 7,55578 | 1,41202 | 1 | 0,05 | 0 | -16,1496 | 37,48738 |
| DHQ, 56 days | Control, 14 days | 9,51558 | 7,55578 | 1,25938 | 1 | 0,05 | 0 | -17,30291 | 36,33407 |
| DHQ, 56 days | Control, 28 days | -3,26682 | 7,16804 | -0,45575 | 1 | 0,05 | 0 | -28,70907 | 22,17544 |
| DHQ, 56 days | Control, 42 days | -7,81466 | 7,85219 | -0,99522 | 1 | 0,05 | 0 | -35,68526 | 20,05593 |
| DHQ, 56 days | Control, 56 days | 9,8687 | 8,27694 | 1,19231 | 1 | 0,05 | 0 | -19,50949 | 39,24688 |
| DHQ, 56 days | DHQ, 1 day | -20,01869 | 7,55578 | -2,64946 | 0,67919 | 0,05 | 0 | -46,83718 | 6,7998 |
| DHQ, 56 days | DHQ, 7 days | -3,95677 | 7,55578 | -0,52368 | 1 | 0,05 | 0 | -30,77526 | 22,86172 |
| DHQ, 56 days | DHQ, 14 days | -0,97382 | 7,33672 | -0,13273 | 1 | 0,05 | 0 | -27,0148 | 25,06715 |
| DHQ, 56 days | DHQ, 28 days | -4,66269 | 7,16804 | -0,65048 | 1 | 0,05 | 0 | -30,10495 | 20,77956 |
| DHQ, 56 days | DHQ, 42 days | 9,54153 | 7,85219 | 1,21514 | 1 | 0,05 | 0 | -18,32907 | 37,41212 |

#### Scheffe Test

| | | MeanDiff | SEM | F Value | Prob | Alpha | Sig | LCL | UCL |
|---|---|---|---|---|---|---|---|---|---|
| Control, 7 days | Control, 1 day | -27,04001 | 6,51226 | 1,56732 | 0,13216 | 0,05 | 0 | -57,21809 | 3,13807 |
| Control, 14 days | Control, 1 day | -25,8867 | 6,51226 | 1,43647 | 0,18067 | 0,05 | 0 | -56,06478 | 4,29138 |
| Control, 14 days | Control, 7 days | 1,15331 | 6,75809 | 0,00265 | 1 | 0,05 | 0 | -30,16397 | 32,47058 |
| Control, 28 days | Control, 1 day | -13,10431 | 6,0581 | 0,42537 | 0,93886 | 0,05 | 0 | -41,17778 | 14,96917 |
| Control, 28 days | Control, 7 days | 13,9357 | 6,32162 | 0,44178 | 0,93043 | 0,05 | 0 | -15,35892 | 43,23033 |
| Control, 28 days | Control, 14 da | 12,7824 | 6,32162 | 0,37169 | 0,96218 | 0,05 | 0 | -16,51223 | 42,07702 |
| Control, 42 days | Control, 1 day | -8,55646 | 6,85396 | 0,14168 | 0,99939 | 0,05 | 0 | -40,31797 | 23,20505 |
| Control, 42 days | Control, 7 days | 18,48355 | 7,08795 | 0,61821 | 0,80613 | 0,05 | 0 | -14,36228 | 51,32938 |
| Control, 42 days | Control, 14 da | 17,33024 | 7,08795 | 0,54347 | 0,86554 | 0,05 | 0 | -15,51559 | 50,17608 |
| Control, 42 days | Control, 28 da | 4,54785 | 6,67308 | 0,04222 | 1 | 0,05 | 0 | -26,37548 | 35,47118 |
| Control, 56 days | Control, 1 day | -26,23982 | 7,33672 | 1,16285 | 0,33176 | 0,05 | 0 | -60,23848 | 7,75885 |
| Control, 56 days | Control, 7 days | 0,80019 | 7,55578 | 0,00102 | 1 | 0,05 | 0 | -34,21358 | 35,81397 |
| Control, 56 days | Control, 14 da | -0,35312 | 7,55578 | 1,98556E-4 | 1 | 0,05 | 0 | -35,36689 | 34,66066 |
| Control, 56 days | Control, 28 da | -13,13551 | 7,16804 | 0,30528 | 0,98218 | 0,05 | 0 | -46,3525 | 20,08147 |
| Control, 56 days | Control, 42 da | -17,68336 | 7,85219 | 0,46106 | 0,91977 | 0,05 | 0 | -54,07074 | 18,70403 |
| DHQ, 1 day | Control, 1 day | 3,64757 | 6,51226 | 0,02852 | 1 | 0,05 | 0 | -26,53051 | 33,82565 |
| DHQ, 1 day | Control, 7 days | 30,68758 | 6,75809 | 1,8745 | 0,06113 | 0,05 | 0 | -0,62969 | 62,00485 |
| DHQ, 1 day | Control, 14 days | 29,53427 | 6,75809 | 1,73625 | 0,08695 | 0,05 | 0 | -1,783 | 60,85155 |
| DHQ, 1 day | Control, 28 days | 16,75188 | 6,32162 | 0,63838 | 0,78881 | 0,05 | 0 | -12,54275 | 46,0465 |
| DHQ, 1 day | Control, 42 days | 12,20403 | 7,08795 | 0,26951 | 0,98925 | 0,05 | 0 | -20,6418 | 45,04987 |
| DHQ, 1 day | Control, 56 days | 29,88739 | 7,55578 | 1,42241 | 0,18671 | 0,05 | 0 | -5,12639 | 64,90116 |
| DHQ, 7 days | Control, 1 day | -12,41435 | 6,51226 | 0,33036 | 0,97574 | 0,05 | 0 | -42,59243 | 17,76373 |
| DHQ, 7 days | Control, 7 days | 14,62566 | 6,75809 | 0,42578 | 0,93866 | 0,05 | 0 | -16,69161 | 45,94293 |
| DHQ, 7 days | Control, 14 days | 13,47235 | 6,75809 | 0,36128 | 0,96595 | 0,05 | 0 | -17,84492 | 44,78963 |
| DHQ, 7 days | Control, 28 days | 0,68996 | 6,32162 | 0,00108 | 1 | 0,05 | 0 | -28,60467 | 29,98458 |
| DHQ, 7 days | Control, 42 days | -3,85789 | 7,08795 | 0,02693 | 1 | 0,05 | 0 | -36,70372 | 28,98795 |
| DHQ, 7 days | Control, 56 days | 13,82547 | 7,55578 | 0,30437 | 0,98239 | 0,05 | 0 | -21,18831 | 48,83924 |
| DHQ, 7 days | DHQ, 1 day | -16,06192 | 6,75809 | 0,51352 | 0,88679 | 0,05 | 0 | -47,37919 | 15,25535 |
| DHQ, 14 days | Control, 1 day | -15,3973 | 6,25678 | 0,55055 | 0,86029 | 0,05 | 0 | -44,39146 | 13,59686 |
| DHQ, 14 days | Control, 7 days | 11,64271 | 6,51226 | 0,29057 | 0,98538 | 0,05 | 0 | -18,53537 | 41,82079 |
| DHQ, 14 days | Control, 14 days | 10,4894 | 6,51226 | 0,23586 | 0,99387 | 0,05 | 0 | -19,68868 | 40,66748 |
| DHQ, 14 days | Control, 28 days | -2,29299 | 6,0581 | 0,01302 | 1 | 0,05 | 0 | -30,36647 | 25,78048 |
| DHQ, 14 days | Control, 42 days | -6,84084 | 6,85396 | 0,09056 | 0,99993 | 0,05 | 0 | -38,60235 | 24,92067 |
| DHQ, 14 days | Control, 56 days | 10,84252 | 7,33672 | 0,19855 | 0,99712 | 0,05 | 0 | -23,15615 | 44,84119 |
| DHQ, 14 days | DHQ, 1 day | -19,04487 | 6,51226 | 0,7775 | 0,66076 | 0,05 | 0 | -49,22295 | 11,13321 |
| DHQ, 14 days | DHQ, 7 days | -2,98295 | 6,51226 | 0,01907 | 1 | 0,05 | 0 | -33,16103 | 27,19513 |
| DHQ, 28 days | Control, 1 day | -11,70843 | 6,0581 | 0,33957 | 0,97304 | 0,05 | 0 | -39,7819 | 16,36505 |
| DHQ, 28 days | Control, 7 days | 15,33158 | 6,32162 | 0,53472 | 0,87192 | 0,05 | 0 | -13,96305 | 44,62621 |
| DHQ, 28 days | Control, 14 days | 14,17827 | 6,32162 | 0,4573 | 0,92192 | 0,05 | 0 | -15,11635 | 43,4729 |
| DHQ, 28 days | Control, 28 days | 1,39588 | 5,85268 | 0,00517 | 1 | 0,05 | 0 | -25,72568 | 28,51743 |
| DHQ, 28 days | Control, 42 days | -3,15197 | 6,67308 | 0,02028 | 1 | 0,05 | 0 | -34,0753 | 27,77136 |
| DHQ, 28 days | Control, 56 days | 14,53139 | 7,16804 | 0,37361 | 0,96146 | 0,05 | 0 | -18,6856 | 47,74837 |
| DHQ, 28 days | DHQ, 1 day | -15,356 | 6,32162 | 0,53642 | 0,87069 | 0,05 | 0 | -44,65063 | 13,93863 |
| DHQ, 28 days | DHQ, 7 days | 0,70592 | 6,32162 | 0,00113 | 1 | 0,05 | 0 | -28,58871 | 30,00055 |
| DHQ, 28 days | DHQ, 14 days | 3,68887 | 6,0581 | 0,03371 | 1 | 0,05 | 0 | -24,38461 | 31,76235 |
| DHQ, 42 days | Control, 1 day | -25,91265 | 6,85396 | 1,29942 | 0,24719 | 0,05 | 0 | -57,67416 | 5,84886 |
| DHQ, 42 days | Control, 7 days | 1,12736 | 7,08795 | 0,0023 | 1 | 0,05 | 0 | -31,71847 | 33,97319 |
| DHQ, 42 days | Control, 14 days | -0,02595 | 7,08795 | 1,21843E-6 | 1 | 0,05 | 0 | -32,87178 | 32,81989 |
| DHQ, 42 days | Control, 28 days | -12,80834 | 6,67308 | 0,33492 | 0,97442 | 0,05 | 0 | -43,73167 | 18,11499 |
| DHQ, 42 days | Control, 42 days | -17,35619 | 7,40312 | 0,49967 | 0,89603 | 0,05 | 0 | -51,66254 | 16,95017 |
| DHQ, 42 days | Control, 56 days | 0,32717 | 7,85219 | 1,57821E-4 | 1 | 0,05 | 0 | -36,06022 | 36,71455 |
| DHQ, 42 days | DHQ, 1 day | -29,56022 | 7,08795 | 1,58118 | 0,12777 | 0,05 | 0 | -62,40605 | 3,28561 |
| DHQ, 42 days | DHQ, 7 days | -13,4983 | 7,08795 | 0,3297 | 0,97592 | 0,05 | 0 | -46,34414 | 19,34753 |
| DHQ, 42 days | DHQ, 14 days | -10,51535 | 6,85396 | 0,21398 | 0,99598 | 0,05 | 0 | -42,27686 | 21,24616 |
| DHQ, 42 days | DHQ, 28 days | -14,20422 | 6,67308 | 0,4119 | 0,94533 | 0,05 | 0 | -45,12755 | 16,71911 |
| DHQ, 56 days | Control, 1 day | -16,37112 | 7,33672 | 0,45265 | 0,92452 | 0,05 | 0 | -50,36979 | 17,62755 |
| DHQ, 56 days | Control, 7 days | 10,66889 | 7,55578 | 0,18125 | 0,99809 | 0,05 | 0 | -24,34489 | 45,68266 |
| DHQ, 56 days | Control, 14 days | 9,51558 | 7,55578 | 0,14418 | 0,99934 | 0,05 | 0 | -25,4982 | 44,52936 |
| DHQ, 56 days | Control, 28 days | -3,26682 | 7,16804 | 0,01888 | 1 | 0,05 | 0 | -36,4838 | 29,95017 |
| DHQ, 56 days | Control, 42 days | -7,81466 | 7,85219 | 0,09004 | 0,99993 | 0,05 | 0 | -44,20205 | 28,57272 |
| DHQ, 56 days | Control, 56 days | 9,8687 | 8,27694 | 0,12924 | 0,99961 | 0,05 | 0 | -28,48698 | 48,22437 |
| DHQ, 56 days | DHQ, 1 day | -20,01869 | 7,55578 | 0,63815 | 0,78901 | 0,05 | 0 | -55,03247 | 14,99508 |
| DHQ, 56 days | DHQ, 7 days | -3,95677 | 7,55578 | 0,02493 | 1 | 0,05 | 0 | -38,97055 | 31,057 |
| DHQ, 56 days | DHQ, 14 days | -0,97382 | 7,33672 | 0,0016 | 1 | 0,05 | 0 | -34,97249 | 33,02484 |
| DHQ, 56 days | DHQ, 28 days | -4,66269 | 7,16804 | 0,03847 | 1 | 0,05 | 0 | -37,87968 | 28,55429 |
| DHQ, 56 days | DHQ, 42 days | 9,54153 | 7,85219 | 0,13423 | 0,99953 | 0,05 | 0 | -26,84586 | 45,92891 |

#### Tukey Test

| | | MeanDiff | SEM | q Value | Prob | Alpha | Sig | LCL | UCL |
|---|---|---|---|---|---|---|---|---|---|
| Control, 7 days | Control, 1 day | -27,04001 | 6,51226 | 5,87205 | 0,00549 | 0,05 | 1 | -49,18239 | -4,89763 |
| Control, 14 days | Control, 1 day | -25,8867 | 6,51226 | 5,6216 | 0,00956 | 0,05 | 1 | -48,02908 | -3,74432 |
| Control, 14 days | Control, 7 days | 1,15331 | 6,75809 | 0,24134 | 1 | 0,05 | 0 | -21,82493 | 24,13154 |
| Control, 28 days | Control, 1 day | -13,10431 | 6,0581 | 3,05909 | 0,58148 | 0,05 | 0 | -33,70249 | 7,49388 |
| Control, 28 days | Control, 7 days | 13,9357 | 6,32162 | 3,11757 | 0,55305 | 0,05 | 0 | -7,55847 | 35,42988 |
| Control, 28 days | Control, 14 da | 12,7824 | 6,32162 | 2,85956 | 0,67706 | 0,05 | 0 | -8,71178 | 34,27657 |
| Control, 42 days | Control, 1 day | -8,55646 | 6,85396 | 1,7655 | 0,98232 | 0,05 | 0 | -31,86064 | 14,74773 |
| Control, 42 days | Control, 7 days | 18,48355 | 7,08795 | 3,68791 | 0,29872 | 0,05 | 0 | -5,61623 | 42,58333 |
| Control, 42 days | Control, 14 da | 17,33024 | 7,08795 | 3,45779 | 0,39351 | 0,05 | 0 | -6,76954 | 41,43002 |
| Control, 42 days | Control, 28 da | 4,54785 | 6,67308 | 0,96382 | 0,99992 | 0,05 | 0 | -18,14134 | 27,23704 |
| Control, 56 days | Control, 1 day | -26,23982 | 7,33672 | 5,05794 | 0,0309 | 0,05 | 1 | -51,18546 | -1,29418 |
| Control, 56 days | Control, 7 days | 0,80019 | 7,55578 | 0,14977 | 1 | 0,05 | 0 | -24,89026 | 26,49064 |
| Control, 56 days | Control, 14 da | -0,35312 | 7,55578 | 0,06609 | 1 | 0,05 | 0 | -26,04356 | 25,33733 |
| Control, 56 days | Control, 28 da | -13,13551 | 7,16804 | 2,59156 | 0,79369 | 0,05 | 0 | -37,50761 | 11,23659 |
| Control, 56 days | Control, 42 da | -17,68336 | 7,85219 | 3,18485 | 0,52046 | 0,05 | 0 | -44,38165 | 9,01494 |
| DHQ, 1 day | Control, 1 day | 3,64757 | 6,51226 | 0,79211 | 0,99999 | 0,05 | 0 | -18,49481 | 25,78995 |
| DHQ, 1 day | Control, 7 days | 30,68758 | 6,75809 | 6,42175 | 0,00153 | 0,05 | 1 | 7,70934 | 53,66582 |
| DHQ, 1 day | Control, 14 days | 29,53427 | 6,75809 | 6,18041 | 0,0027 | 0,05 | 1 | 6,55604 | 52,51251 |
| DHQ, 1 day | Control, 28 days | 16,75188 | 6,32162 | 3,74757 | 0,27655 | 0,05 | 0 | -4,74229 | 38,24605 |
| DHQ, 1 day | Control, 42 days | 12,20403 | 7,08795 | 2,43499 | 0,85099 | 0,05 | 0 | -11,89575 | 36,30381 |
| DHQ, 1 day | Control, 56 days | 29,88739 | 7,55578 | 5,59402 | 0,01015 | 0,05 | 1 | 4,19694 | 55,57784 |
| DHQ, 7 days | Control, 1 day | -12,41435 | 6,51226 | 2,69592 | 0,75062 | 0,05 | 0 | -34,55673 | 9,72803 |
| DHQ, 7 days | Control, 7 days | 14,62566 | 6,75809 | 3,0606 | 0,58075 | 0,05 | 0 | -8,35258 | 37,6039 |
| DHQ, 7 days | Control, 14 days | 13,47235 | 6,75809 | 2,81925 | 0,69573 | 0,05 | 0 | -9,50588 | 36,45059 |
| DHQ, 7 days | Control, 28 days | 0,68996 | 6,32162 | 0,15435 | 1 | 0,05 | 0 | -20,80421 | 22,18413 |
| DHQ, 7 days | Control, 42 days | -3,85789 | 7,08795 | 0,76974 | 0,99999 | 0,05 | 0 | -27,95767 | 20,24189 |
| DHQ, 7 days | Control, 56 days | 13,82547 | 7,55578 | 2,58771 | 0,79521 | 0,05 | 0 | -11,86498 | 39,51592 |
| DHQ, 7 days | DHQ, 1 day | -16,06192 | 6,75809 | 3,36115 | 0,43708 | 0,05 | 0 | -39,04016 | 6,91632 |
| DHQ, 14 days | Control, 1 day | -15,3973 | 6,25678 | 3,48024 | 0,38367 | 0,05 | 0 | -36,67101 | 5,87642 |
| DHQ, 14 days | Control, 7 days | 11,64271 | 6,51226 | 2,52835 | 0,81796 | 0,05 | 0 | -10,49967 | 33,78509 |
| DHQ, 14 days | Control, 14 days | 10,4894 | 6,51226 | 2,2779 | 0,89833 | 0,05 | 0 | -11,65298 | 32,63179 |
| DHQ, 14 days | Control, 28 days | -2,29299 | 6,0581 | 0,53528 | 1 | 0,05 | 0 | -22,89118 | 18,30519 |
| DHQ, 14 days | Control, 42 days | -6,84084 | 6,85396 | 1,41151 | 0,9972 | 0,05 | 0 | -30,14502 | 16,46335 |
| DHQ, 14 days | Control, 56 days | 10,84252 | 7,33672 | 2,08998 | 0,9409 | 0,05 | 0 | -14,10312 | 35,78816 |
| DHQ, 14 days | DHQ, 1 day | -19,04487 | 6,51226 | 4,13582 | 0,15871 | 0,05 | 0 | -41,18725 | 3,09751 |
| DHQ, 14 days | DHQ, 7 days | -2,98295 | 6,51226 | 0,64778 | 1 | 0,05 | 0 | -25,12533 | 19,15943 |
| DHQ, 28 days | Control, 1 day | -11,70843 | 6,0581 | 2,73324 | 0,73442 | 0,05 | 0 | -32,30661 | 8,88976 |
| DHQ, 28 days | Control, 7 days | 15,33158 | 6,32162 | 3,42984 | 0,40591 | 0,05 | 0 | -6,16259 | 36,82575 |
| DHQ, 28 days | Control, 14 days | 14,17827 | 6,32162 | 3,17183 | 0,52675 | 0,05 | 0 | -7,3159 | 35,67245 |
| DHQ, 28 days | Control, 28 days | 1,39588 | 5,85268 | 0,33729 | 1 | 0,05 | 0 | -18,50386 | 21,29561 |
| DHQ, 28 days | Control, 42 days | -3,15197 | 6,67308 | 0,66799 | 1 | 0,05 | 0 | -25,84116 | 19,53722 |
| DHQ, 28 days | Control, 56 days | 14,53139 | 7,16804 | 2,86696 | 0,6736 | 0,05 | 0 | -9,84071 | 38,90349 |
| DHQ, 28 days | DHQ, 1 day | -15,356 | 6,32162 | 3,4353 | 0,40348 | 0,05 | 0 | -36,85017 | 6,13817 |
| DHQ, 28 days | DHQ, 7 days | 0,70592 | 6,32162 | 0,15792 | 1 | 0,05 | 0 | -20,78825 | 22,20009 |
| DHQ, 28 days | DHQ, 14 days | 3,68887 | 6,0581 | 0,86114 | 0,99997 | 0,05 | 0 | -16,90931 | 24,28705 |
| DHQ, 42 days | Control, 1 day | -25,91265 | 6,85396 | 5,3467 | 0,01718 | 0,05 | 1 | -49,21683 | -2,60846 |
| DHQ, 42 days | Control, 7 days | 1,12736 | 7,08795 | 0,22493 | 1 | 0,05 | 0 | -22,97242 | 25,22714 |
| DHQ, 42 days | Control, 14 days | -0,02595 | 7,08795 | 0,00518 | 1 | 0,05 | 0 | -24,12573 | 24,07383 |
| DHQ, 42 days | Control, 28 days | -12,80834 | 6,67308 | 2,71445 | 0,74262 | 0,05 | 0 | -35,49753 | 9,88085 |
| DHQ, 42 days | Control, 42 days | -17,35619 | 7,40312 | 3,31554 | 0,45824 | 0,05 | 0 | -42,52759 | 7,81521 |
| DHQ, 42 days | Control, 56 days | 0,32717 | 7,85219 | 0,05892 | 1 | 0,05 | 0 | -26,37113 | 27,02546 |
| DHQ, 42 days | DHQ, 1 day | -29,56022 | 7,08795 | 5,89797 | 0,00517 | 0,05 | 1 | -53,66 | -5,46044 |
| DHQ, 42 days | DHQ, 7 days | -13,4983 | 7,08795 | 2,69323 | 0,75177 | 0,05 | 0 | -37,59808 | 10,60148 |
| DHQ, 42 days | DHQ, 14 days | -10,51535 | 6,85396 | 2,16969 | 0,92469 | 0,05 | 0 | -33,81954 | 12,78883 |
| DHQ, 42 days | DHQ, 28 days | -14,20422 | 6,67308 | 3,01027 | 0,60517 | 0,05 | 0 | -36,89341 | 8,48497 |
| DHQ, 56 days | Control, 1 day | -16,37112 | 7,33672 | 3,15567 | 0,53457 | 0,05 | 0 | -41,31676 | 8,57452 |
| DHQ, 56 days | Control, 7 days | 10,66889 | 7,55578 | 1,99689 | 0,95657 | 0,05 | 0 | -15,02156 | 36,35934 |
| DHQ, 56 days | Control, 14 days | 9,51558 | 7,55578 | 1,78103 | 0,9811 | 0,05 | 0 | -16,17487 | 35,20603 |
| DHQ, 56 days | Control, 28 days | -3,26682 | 7,16804 | 0,64452 | 1 | 0,05 | 0 | -27,63892 | 21,10528 |
| DHQ, 56 days | Control, 42 days | -7,81466 | 7,85219 | 1,40745 | 0,99727 | 0,05 | 0 | -34,51296 | 18,88364 |
| DHQ, 56 days | Control, 56 days | 9,8687 | 8,27694 | 1,68618 | 0,98764 | 0,05 | 0 | -18,27378 | 38,01117 |
| DHQ, 56 days | DHQ, 1 day | -20,01869 | 7,55578 | 3,7469 | 0,27679 | 0,05 | 0 | -45,70914 | 5,67176 |
| DHQ, 56 days | DHQ, 7 days | -3,95677 | 7,55578 | 0,74059 | 0,99999 | 0,05 | 0 | -29,64722 | 21,73368 |
| DHQ, 56 days | DHQ, 14 days | -0,97382 | 7,33672 | 0,18771 | 1 | 0,05 | 0 | -25,91946 | 23,97182 |
| DHQ, 56 days | DHQ, 28 days | -4,66269 | 7,16804 | 0,91992 | 0,99995 | 0,05 | 0 | -29,03479 | 19,70941 |
| DHQ, 56 days | DHQ, 42 days | 9,54153 | 7,85219 | 1,71847 | 0,98565 | 0,05 | 0 | -17,15677 | 36,23983 |

### Grouping Letters

#### Bonferroni Test

| | Mean | Groups | |
|---|---|---|---|
| DHQ, 1 day | 43,63224 | A | |
| Control, 1 day | 39,98467 | A | |
| Control, 42 days | 31,42821 | A | B |
| DHQ, 28 days | 28,27625 | A | B |
| DHQ, 7 days | 27,57032 | A | B |
| Control, 28 days | 26,88037 | A | B |
| DHQ, 14 days | 24,58737 | A | B |
| DHQ, 56 days | 23,61355 | A | B |
| Control, 14 days | 14,09797 | | B |
| DHQ, 42 days | 14,07202 | | B |
| Control, 56 days | 13,74486 | | B |
| Control, 7 days | 12,94466 | | B |

Means that do not share a letter are significantly different.

#### Scheffe Test

| | Mean | Groups |
|---|---|---|
| DHQ, 1 day | 43,63224 | A |
| Control, 1 day | 39,98467 | A |
| Control, 42 days | 31,42821 | A |
| DHQ, 28 days | 28,27625 | A |
| DHQ, 7 days | 27,57032 | A |
| Control, 28 days | 26,88037 | A |
| DHQ, 14 days | 24,58737 | A |
| DHQ, 56 days | 23,61355 | A |
| Control, 14 days | 14,09797 | A |
| DHQ, 42 days | 14,07202 | A |
| Control, 56 days | 13,74486 | A |
| Control, 7 days | 12,94466 | A |

Means that do not share a letter are significantly different.

#### Tukey Test

| | Mean | Groups | |
|---|---|---|---|
| DHQ, 1 day | 43,63224 | A | |
| Control, 1 day | 39,98467 | A | |
| Control, 42 days | 31,42821 | A | B |
| DHQ, 28 days | 28,27625 | A | B |
| DHQ, 7 days | 27,57032 | A | B |
| Control, 28 days | 26,88037 | A | B |
| DHQ, 14 days | 24,58737 | A | B |
| DHQ, 56 days | 23,61355 | A | B |
| Control, 14 days | 14,09797 | | B |
| DHQ, 42 days | 14,07202 | | B |
| Control, 56 days | 13,74486 | | B |
| Control, 7 days | 12,94466 | | B |

Means that do not share a letter are significantly different.

Sig equals 1 indicates that the difference of the means is significant at the 0,05 level.
Sig equals 0 indicates that the difference of the means is not significant at the 0,05 level.

### Powers

| | Alpha | Sample Size | Power |
|---|---|---|---|
| Actual Power | 0,05 | 72 | 0,99835 |
